# Supplementary figures and images for: Metformin alleviates stress-induced cellular senescence of aging human adipose stromal cells and the ensuing adipocyte dysfunction (part 1 of 2)
Source: eLife. 2021 Sep 21;10:e62635. doi: 10.7554/eLife.62635 (PMC8526089; doi:10.7554/eLife.62635)

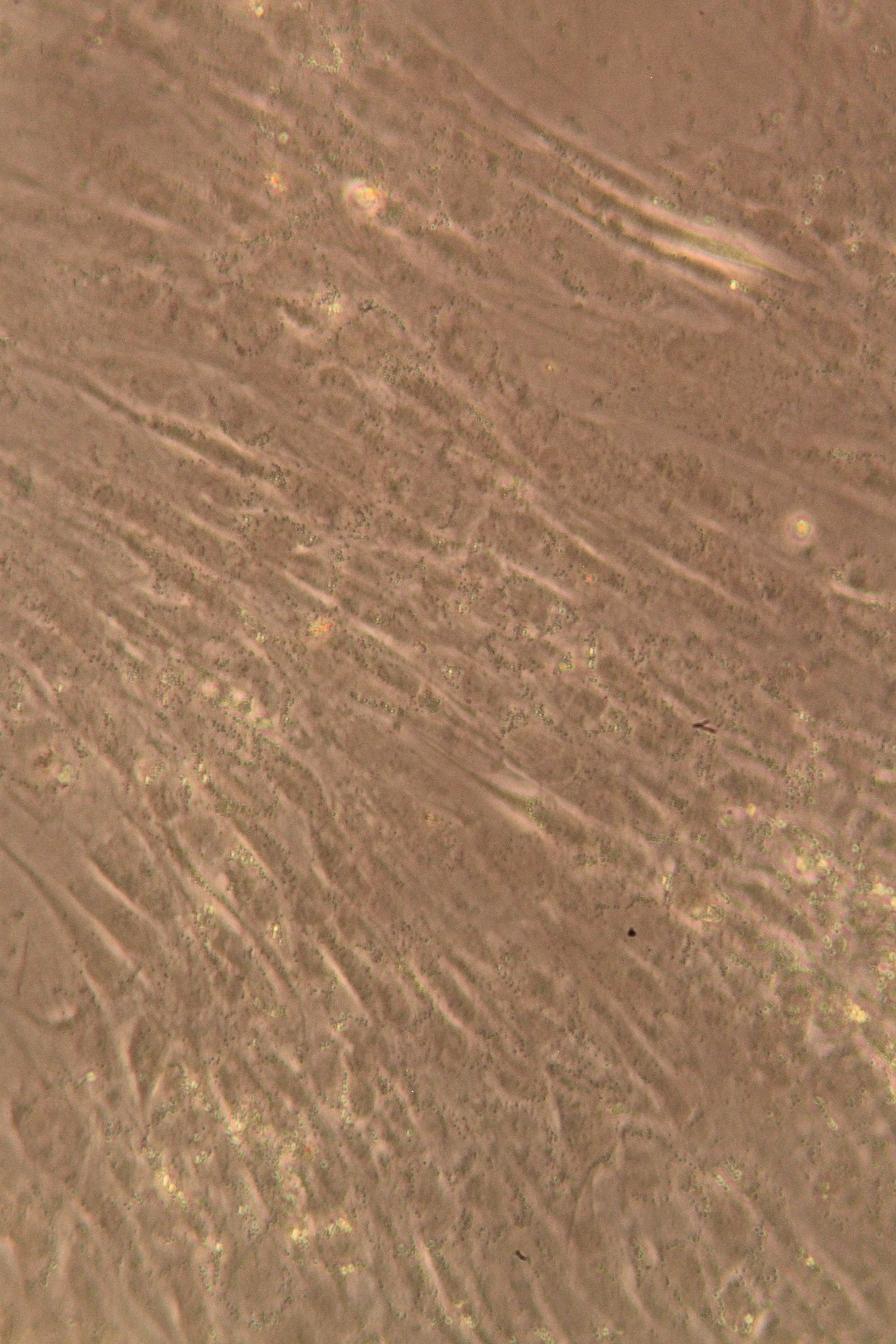

Supplement: Figure 1—source data 1. [file elife-62635-fig1-data1.zip › beta galactosidase P3/beta galactosidase P3- Young ASCs/image 1 .jpg]

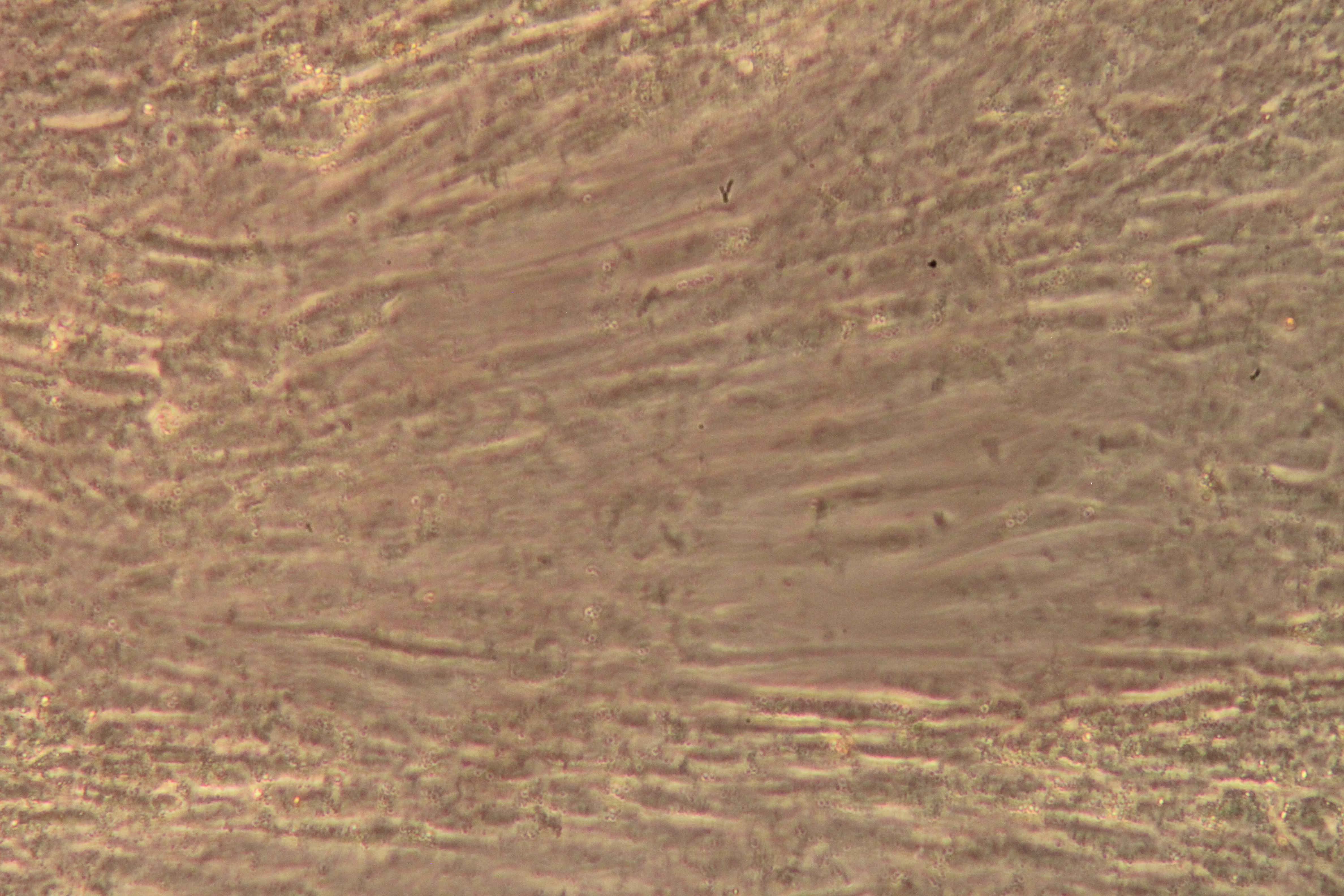

Supplement: Figure 1—source data 1. [file elife-62635-fig1-data1.zip › beta galactosidase P3/beta galactosidase P3- Young ASCs/image 8.JPG]

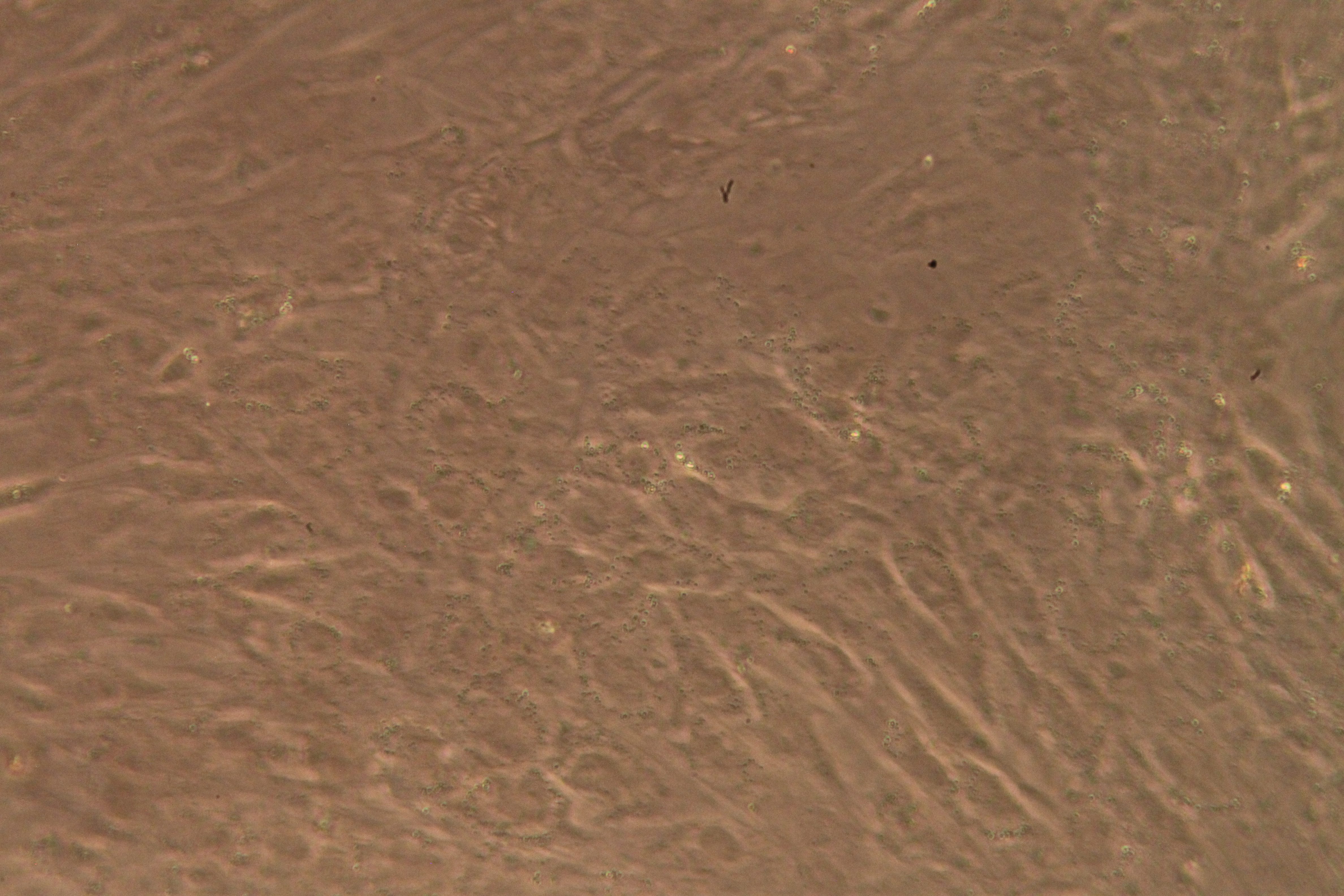

Supplement: Figure 1—source data 1. [file elife-62635-fig1-data1.zip › beta galactosidase P3/beta galactosidase P3- Young ASCs/image 7.JPG]

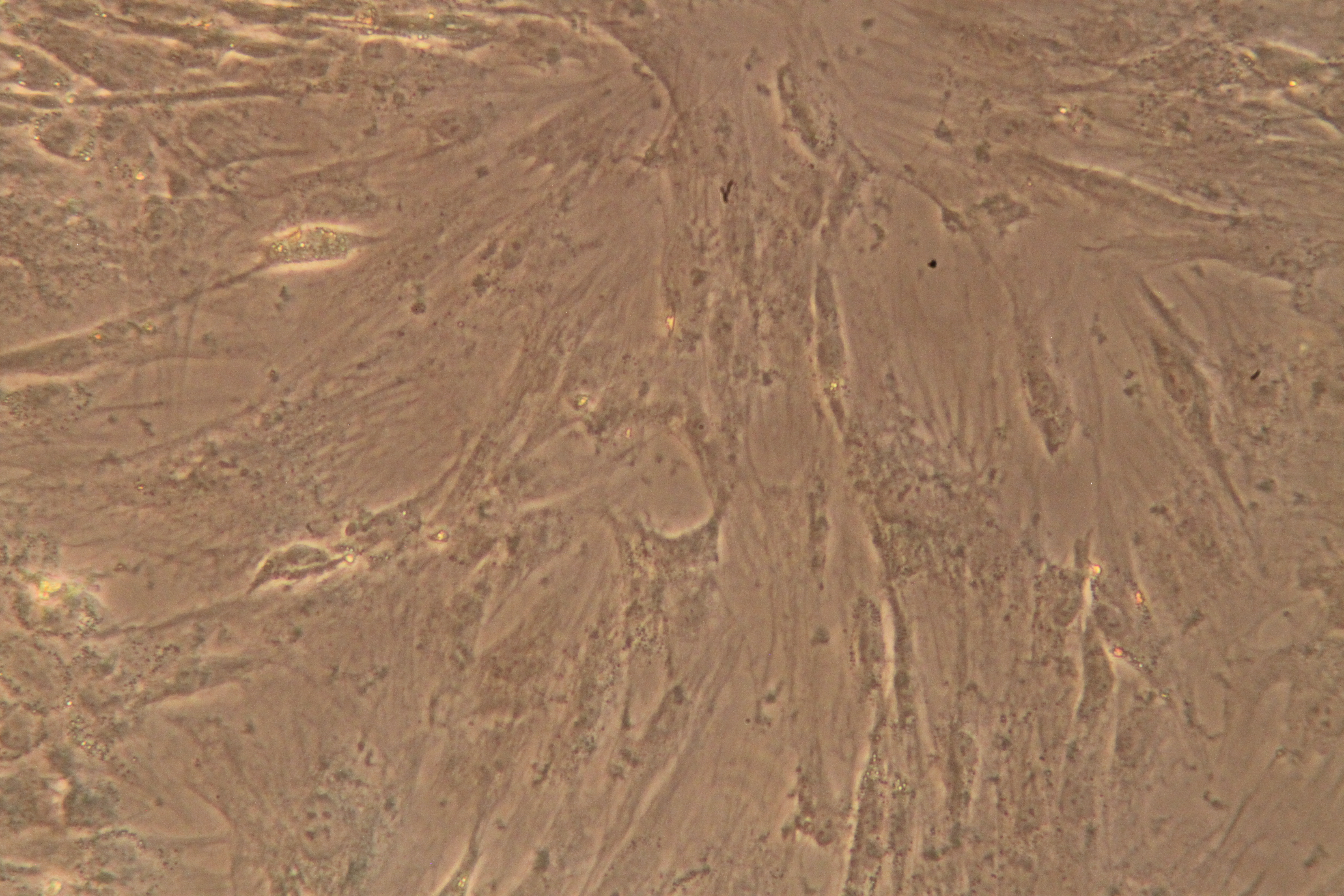

Supplement: Figure 1—source data 1. [file elife-62635-fig1-data1.zip › beta galactosidase P3/beta galactosidase P3- Young ASCs/image 6.JPG]

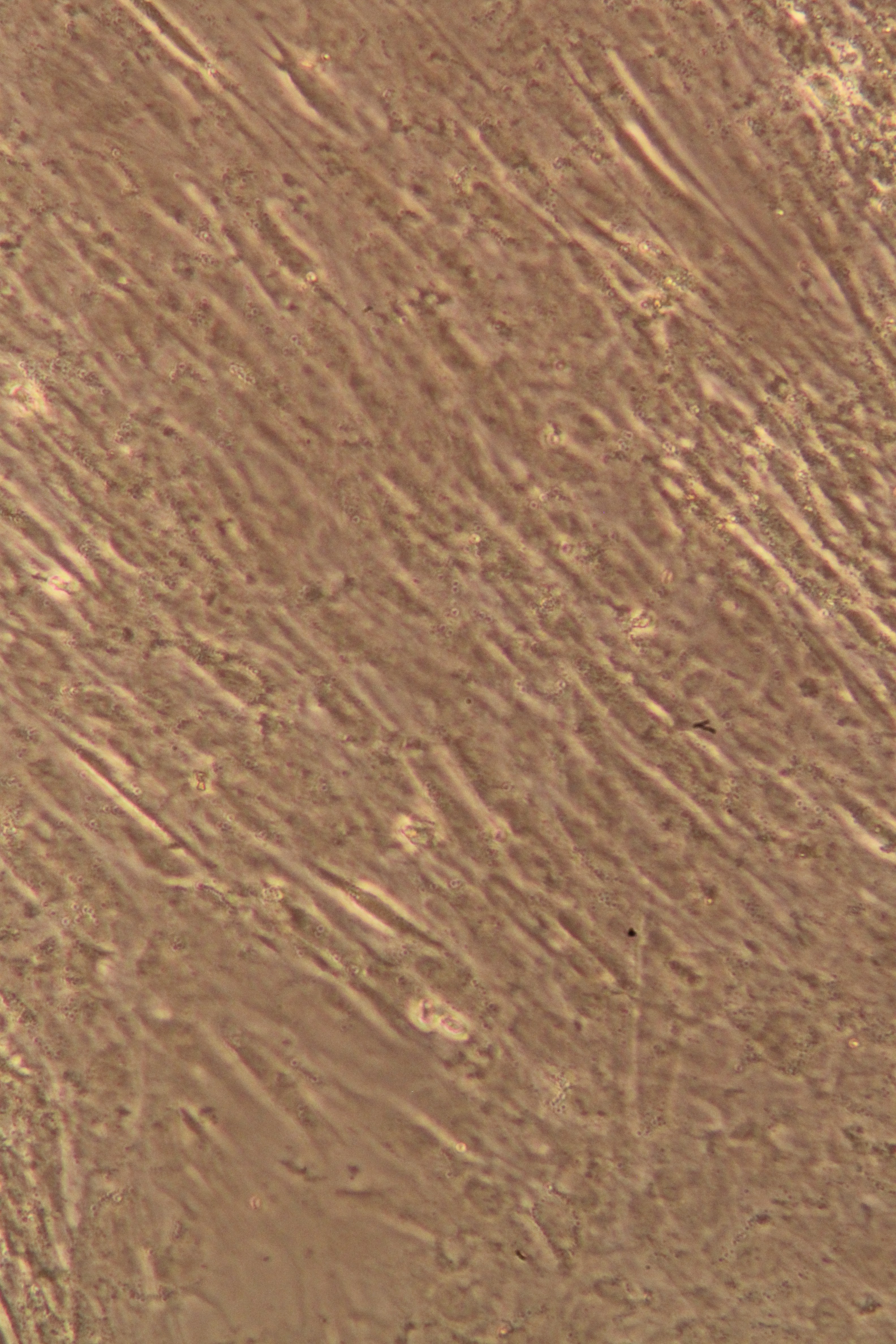

Supplement: Figure 1—source data 1. [file elife-62635-fig1-data1.zip › beta galactosidase P3/beta galactosidase P3- Young ASCs/image 3 .jpg]

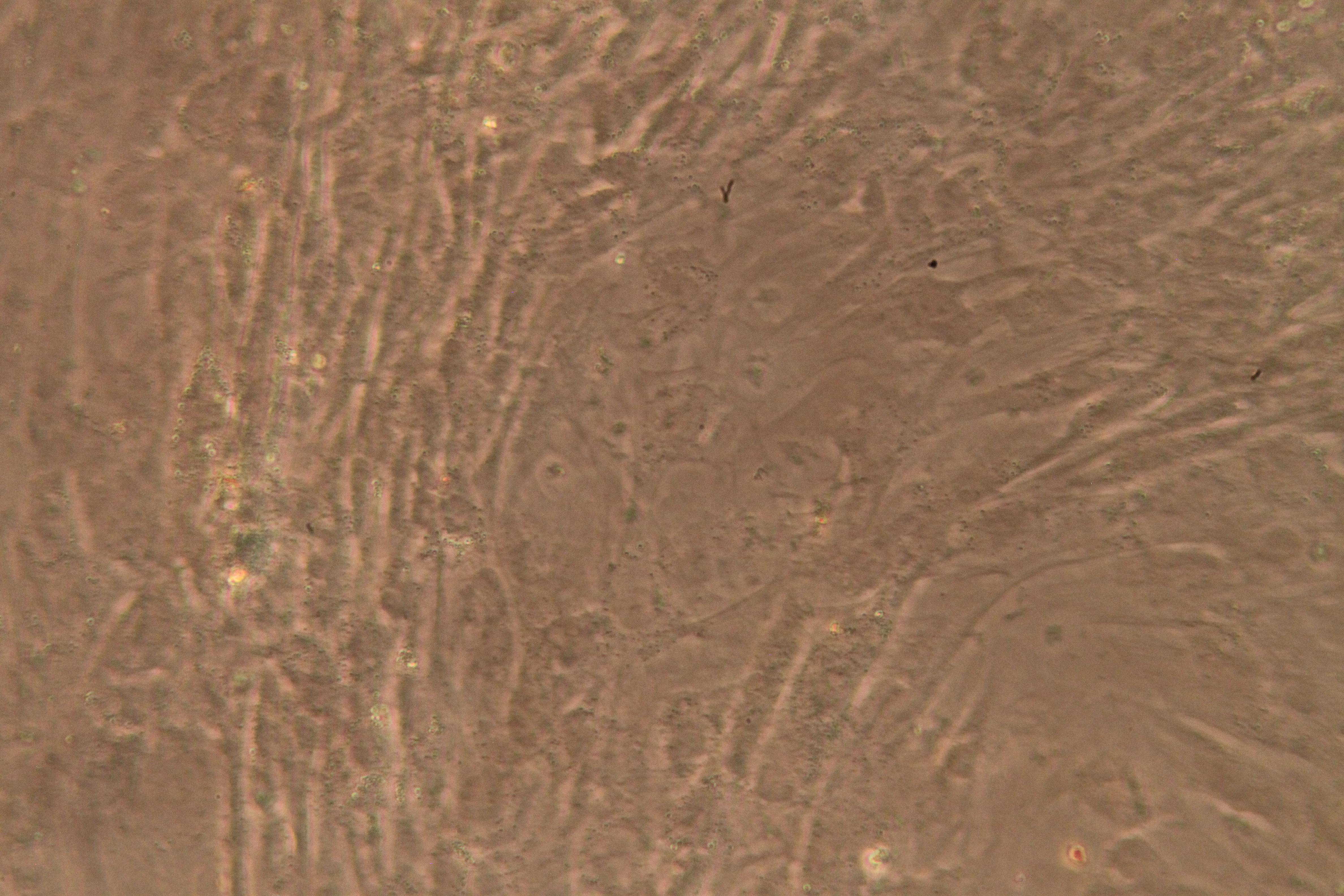

Supplement: Figure 1—source data 1. [file elife-62635-fig1-data1.zip › beta galactosidase P3/beta galactosidase P3- Young ASCs/image 4.JPG]

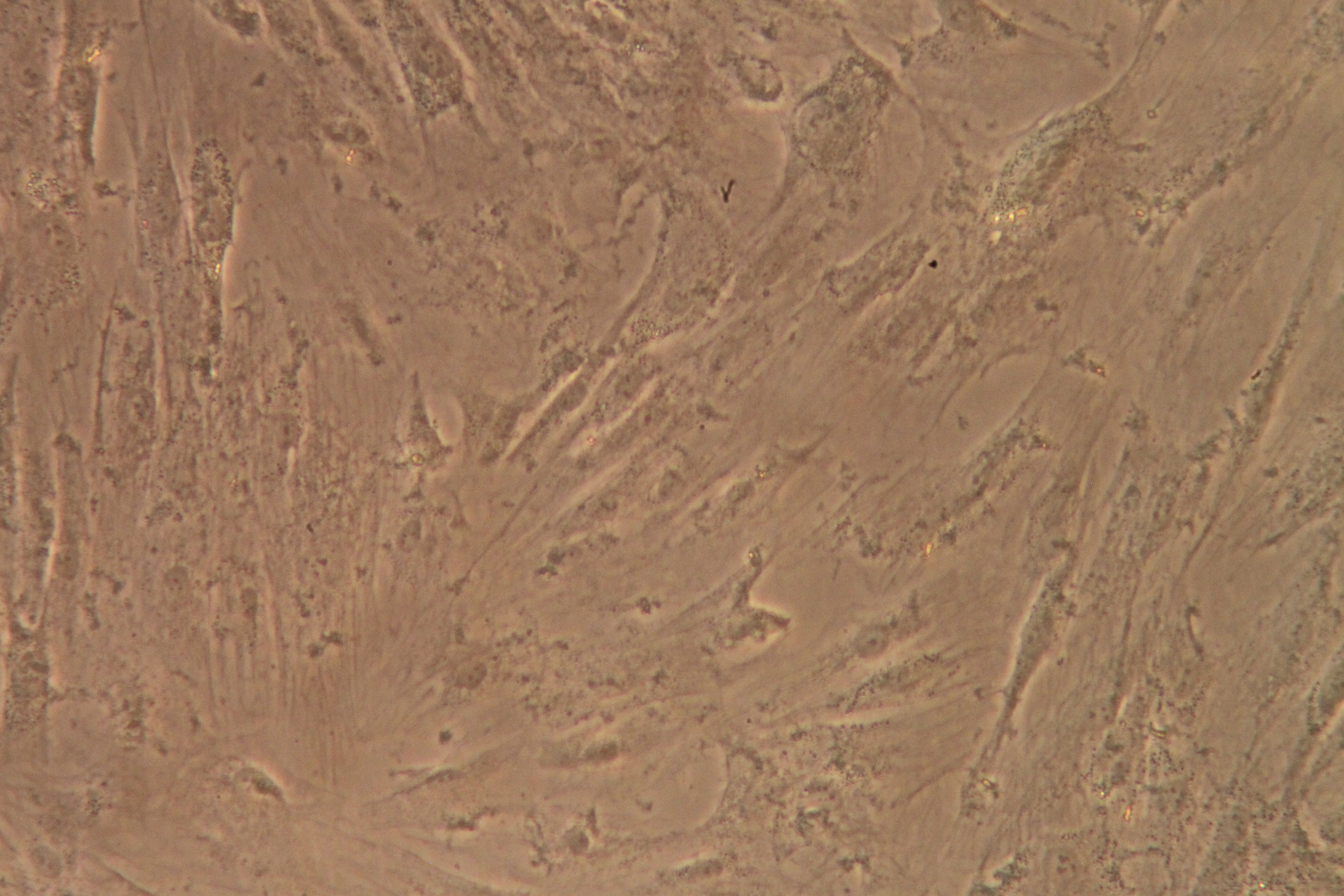

Supplement: Figure 1—source data 1. [file elife-62635-fig1-data1.zip › beta galactosidase P3/beta galactosidase P3- Young ASCs/image 5.JPG]

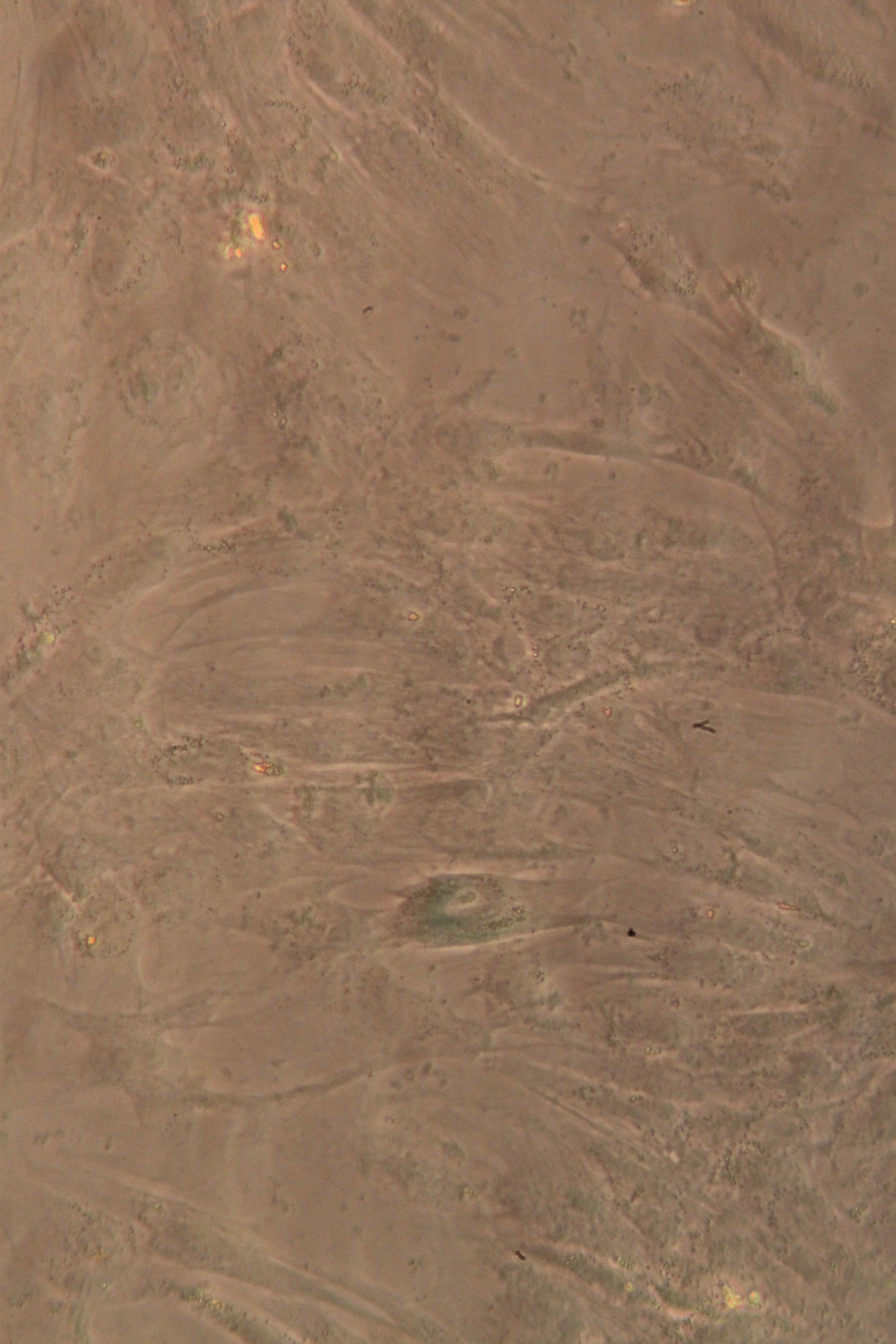

Supplement: Figure 1—source data 1. [file elife-62635-fig1-data1.zip › beta galactosidase P3/beta galactosidase P3- Young ASCs/image 2 .jpg]

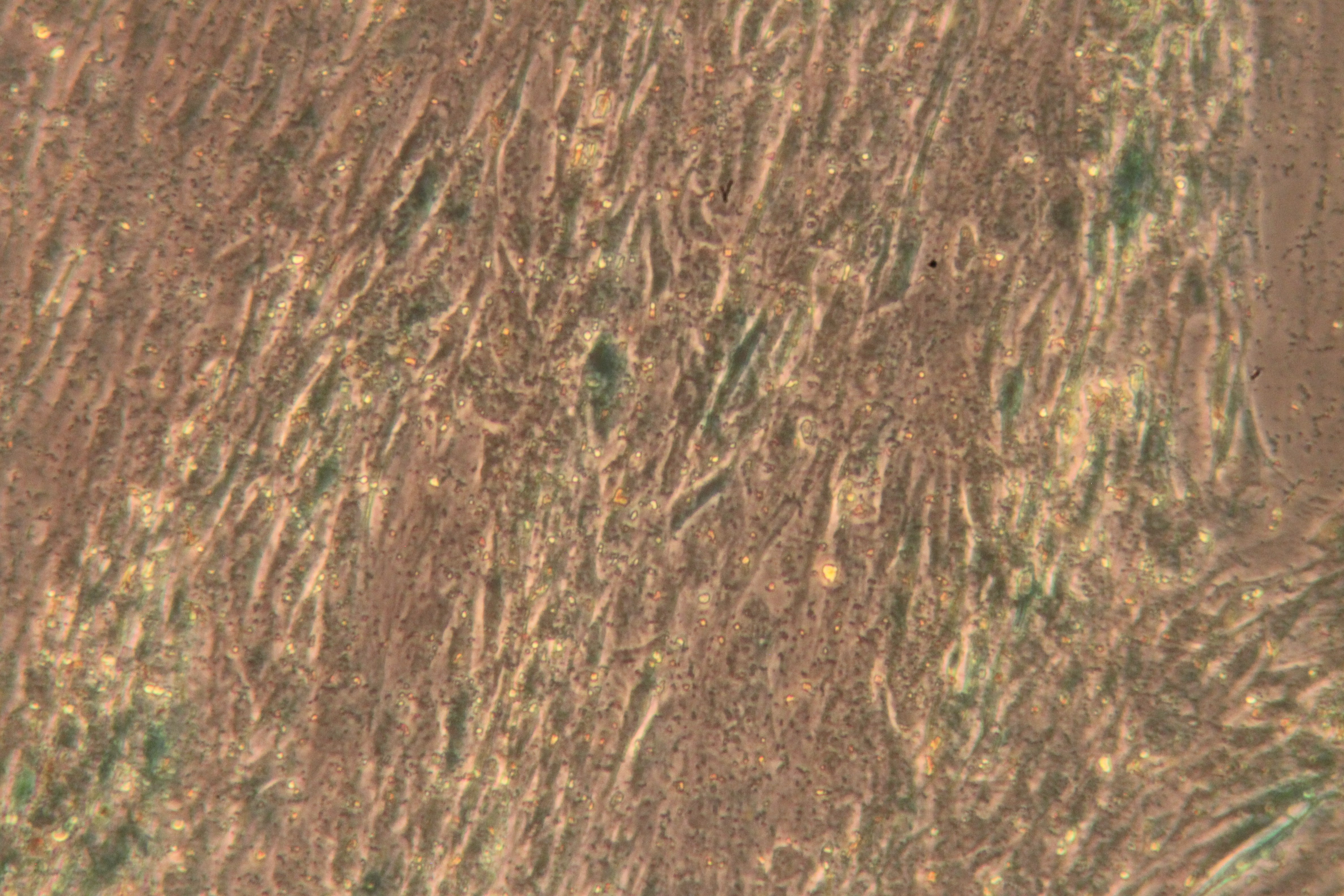

Supplement: Figure 1—source data 1. [file elife-62635-fig1-data1.zip › beta galactosidase P3/beta galactosidase P3- Aged ASCs/image 1 .jpg]

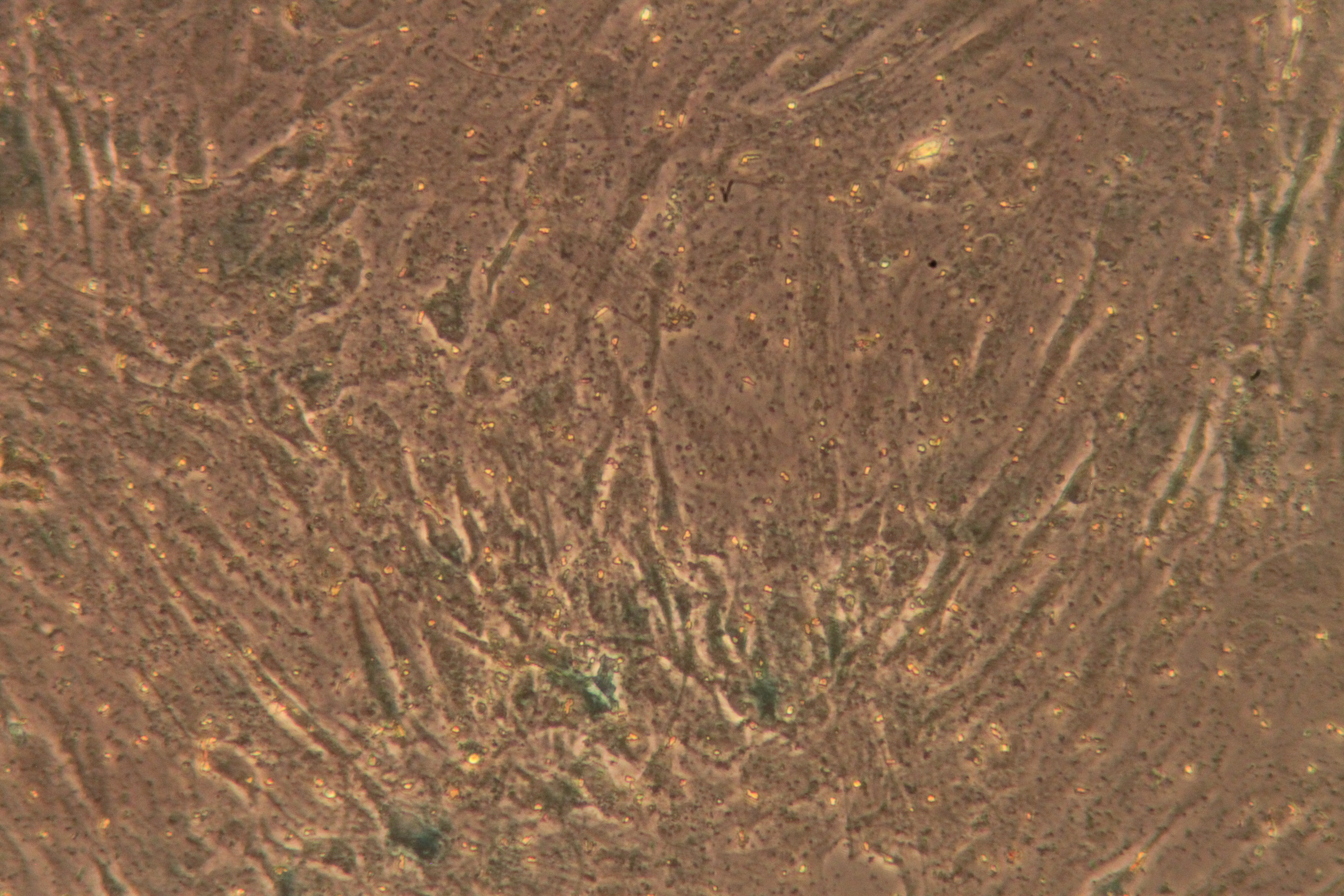

Supplement: Figure 1—source data 1. [file elife-62635-fig1-data1.zip › beta galactosidase P3/beta galactosidase P3- Aged ASCs/image 8.JPG]

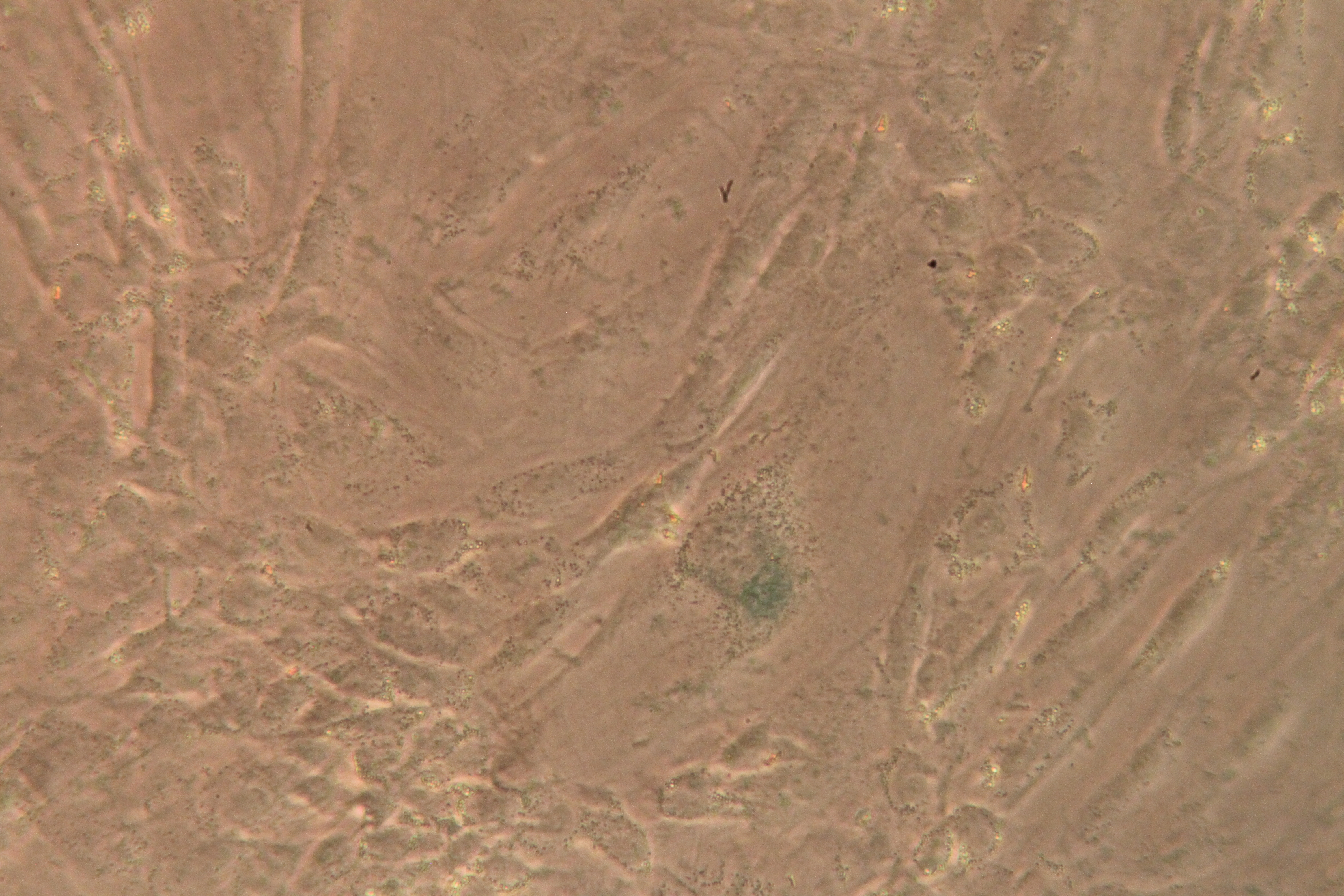

Supplement: Figure 1—source data 1. [file elife-62635-fig1-data1.zip › beta galactosidase P3/beta galactosidase P3- Aged ASCs/image 7.JPG]

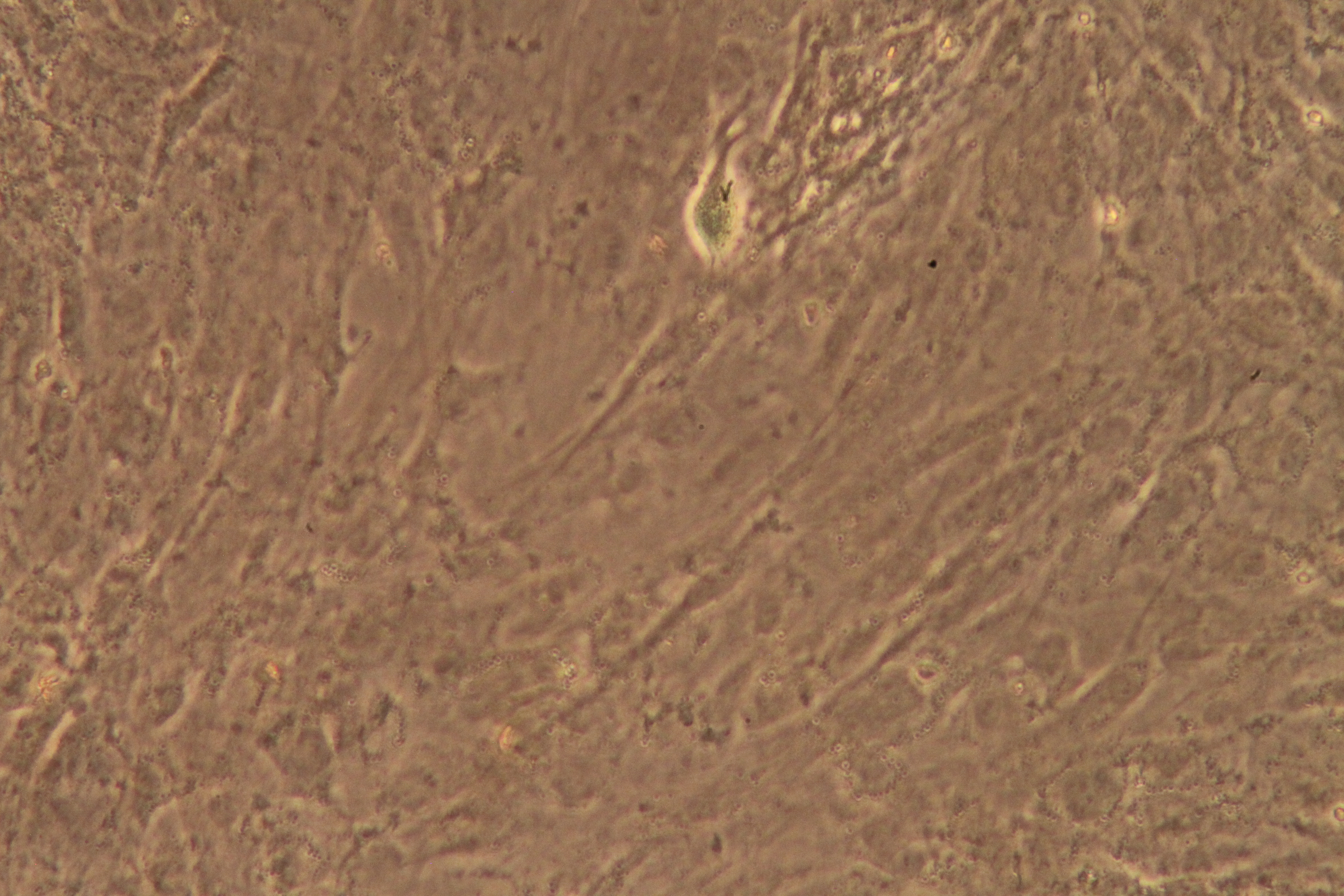

Supplement: Figure 1—source data 1. [file elife-62635-fig1-data1.zip › beta galactosidase P3/beta galactosidase P3- Aged ASCs/image 6.JPG]

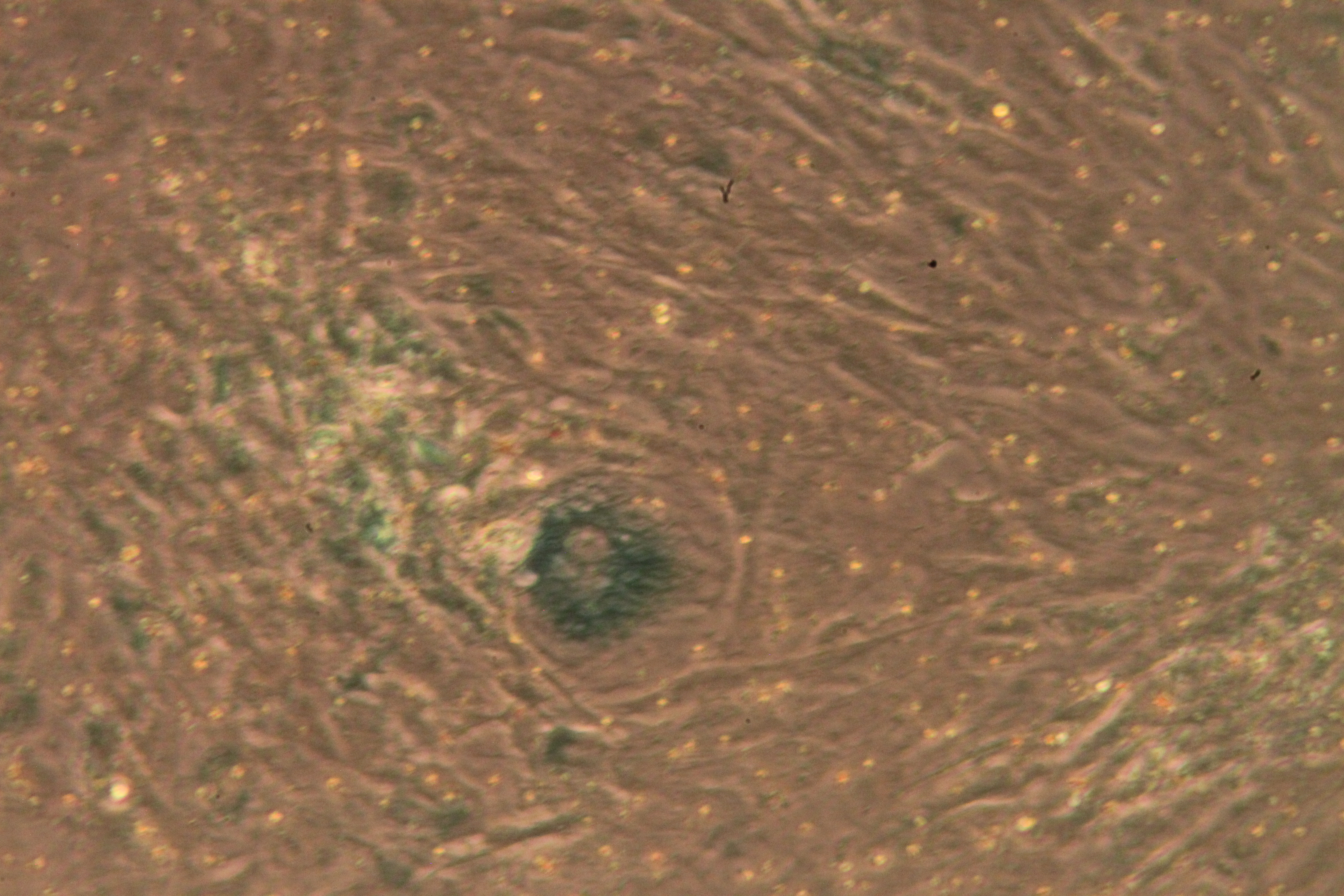

Supplement: Figure 1—source data 1. [file elife-62635-fig1-data1.zip › beta galactosidase P3/beta galactosidase P3- Aged ASCs/image 4.JPG]

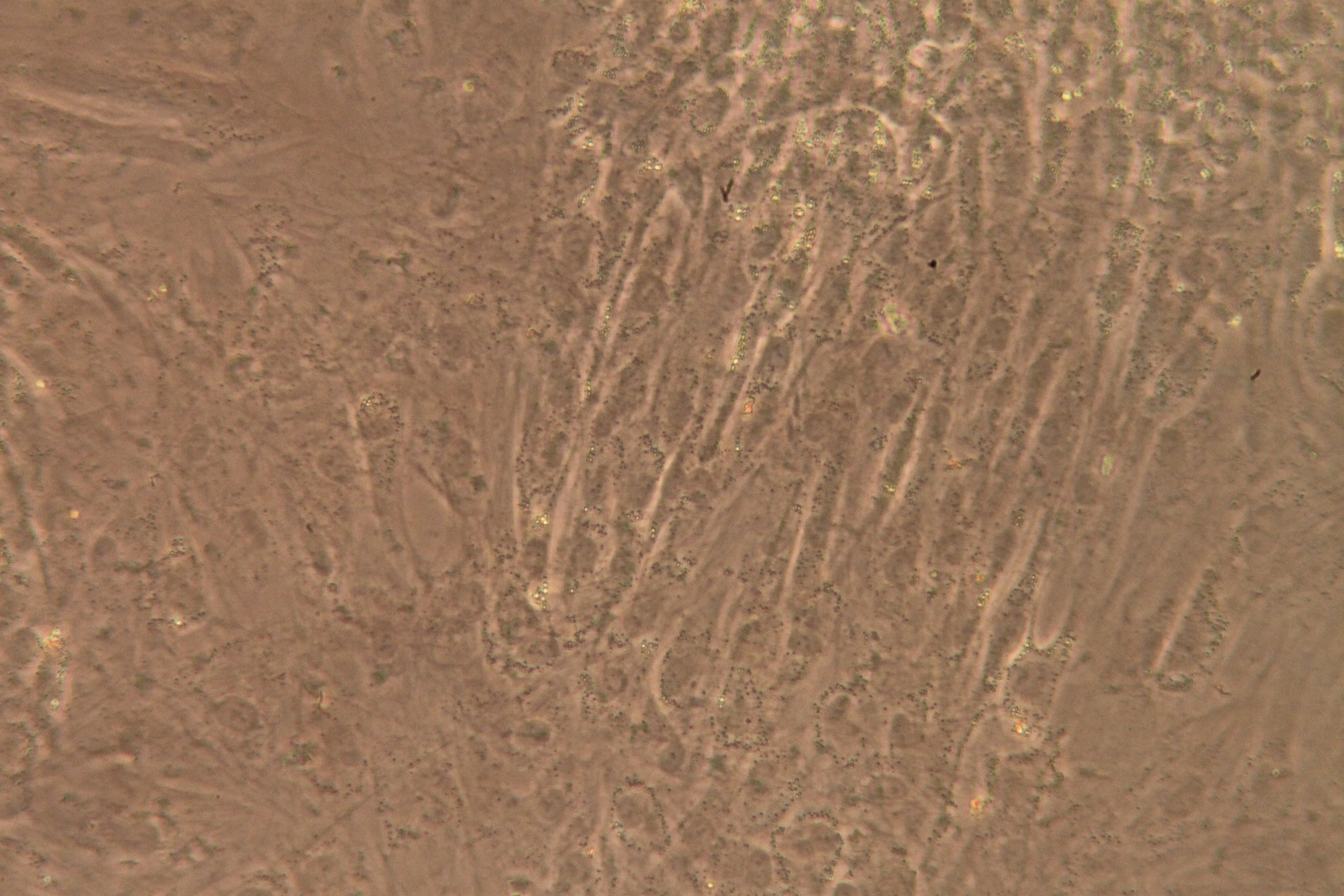

Supplement: Figure 1—source data 1. [file elife-62635-fig1-data1.zip › beta galactosidase P3/beta galactosidase P3- Aged ASCs/image 5.JPG]

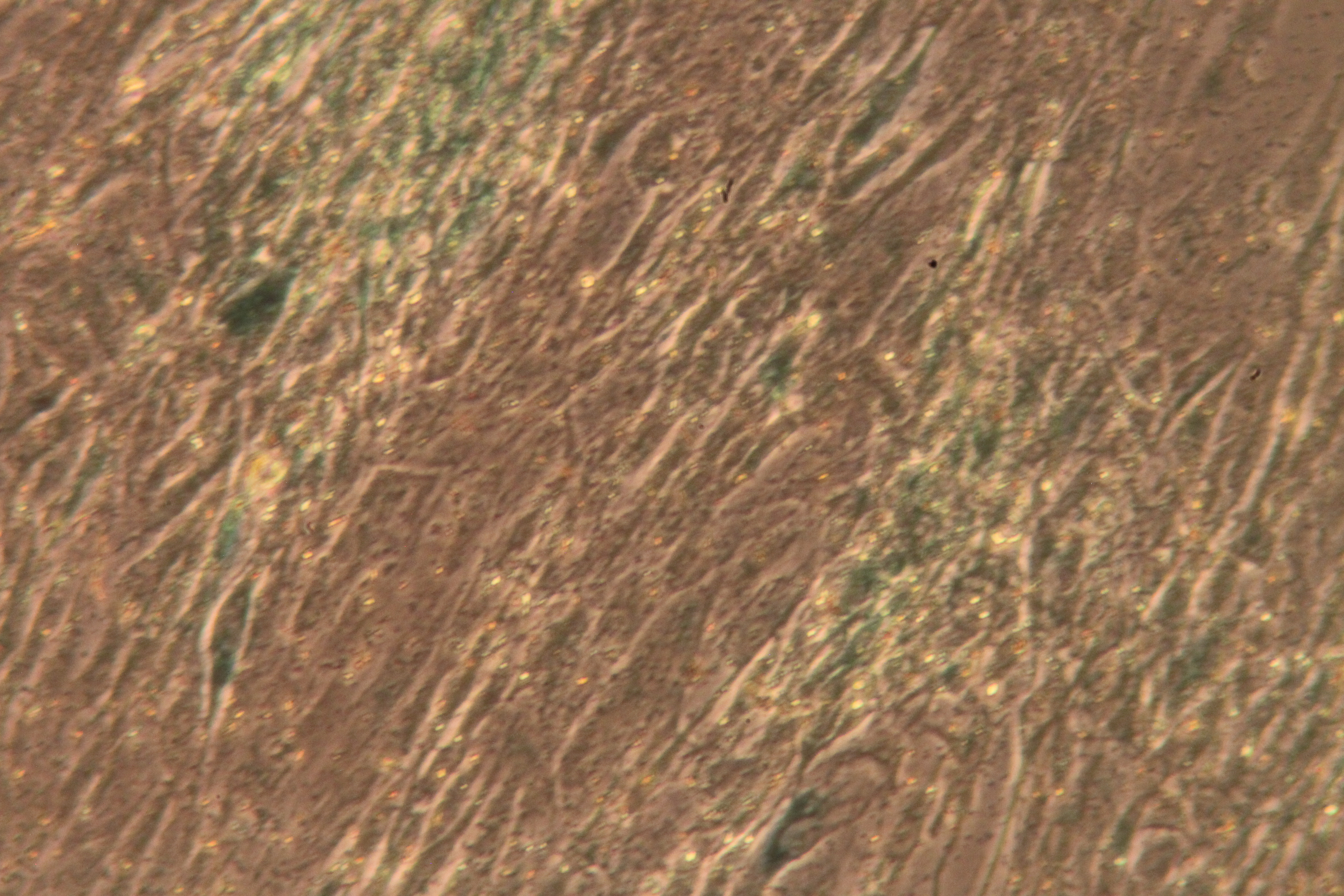

Supplement: Figure 1—source data 1. [file elife-62635-fig1-data1.zip › beta galactosidase P3/beta galactosidase P3- Aged ASCs/image 2.JPG]

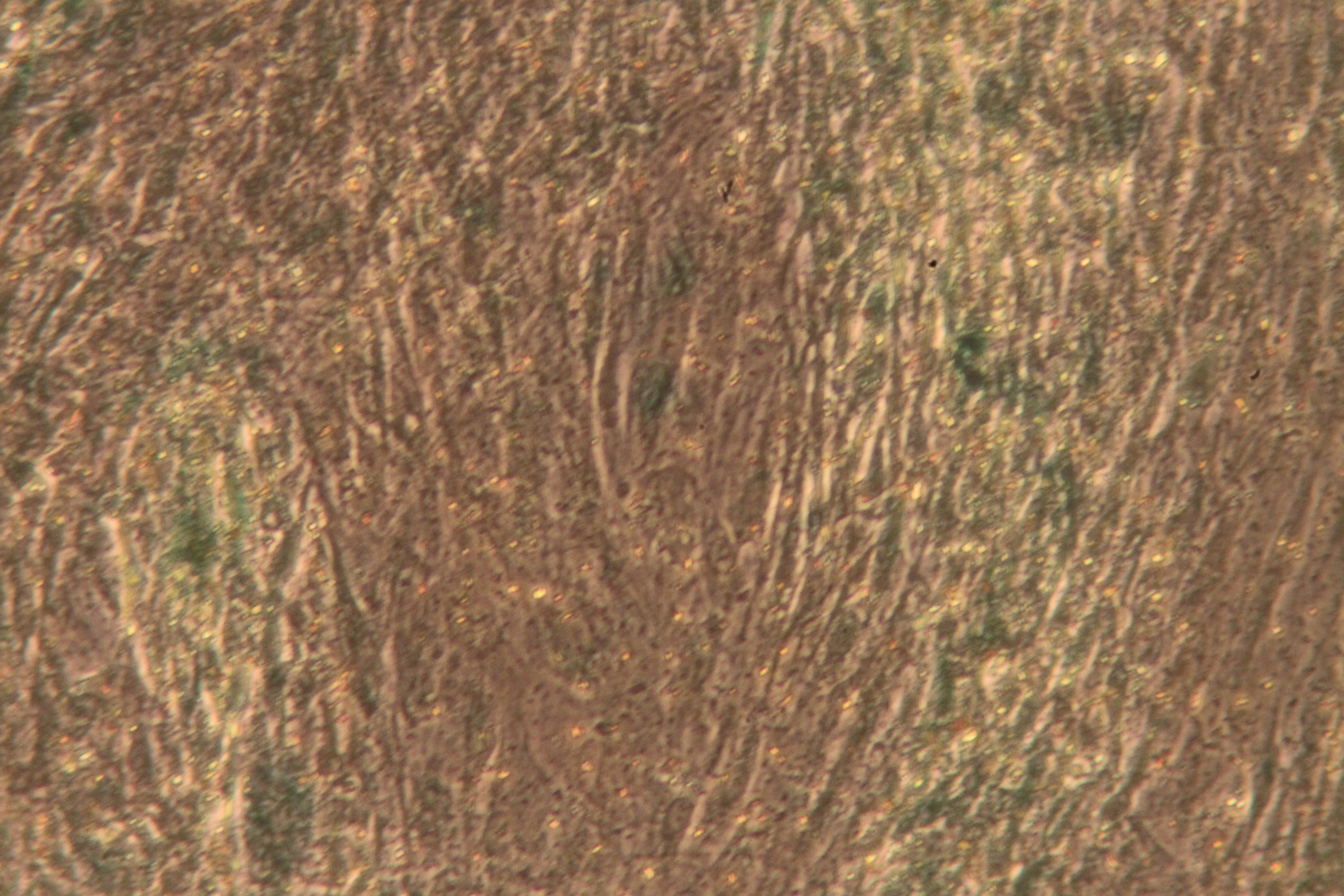

Supplement: Figure 1—source data 1. [file elife-62635-fig1-data1.zip › beta galactosidase P3/beta galactosidase P3- Aged ASCs/image 3.JPG]

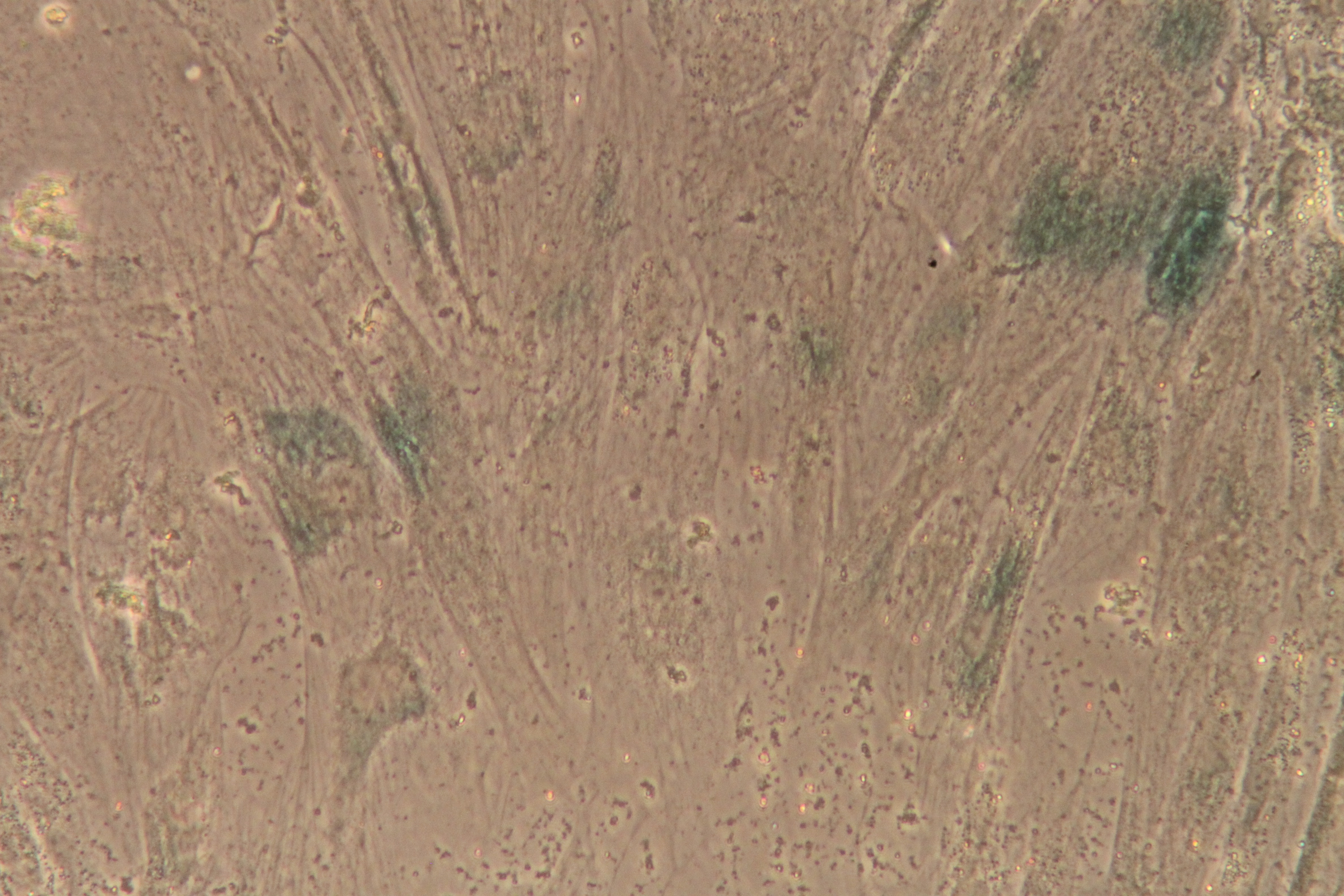

Supplement: Figure 1—source data 2. [file elife-62635-fig1-data2.zip › beta galactosidase P7/beta galactosidase P7- Aged ASCs/image 1 .jpg]

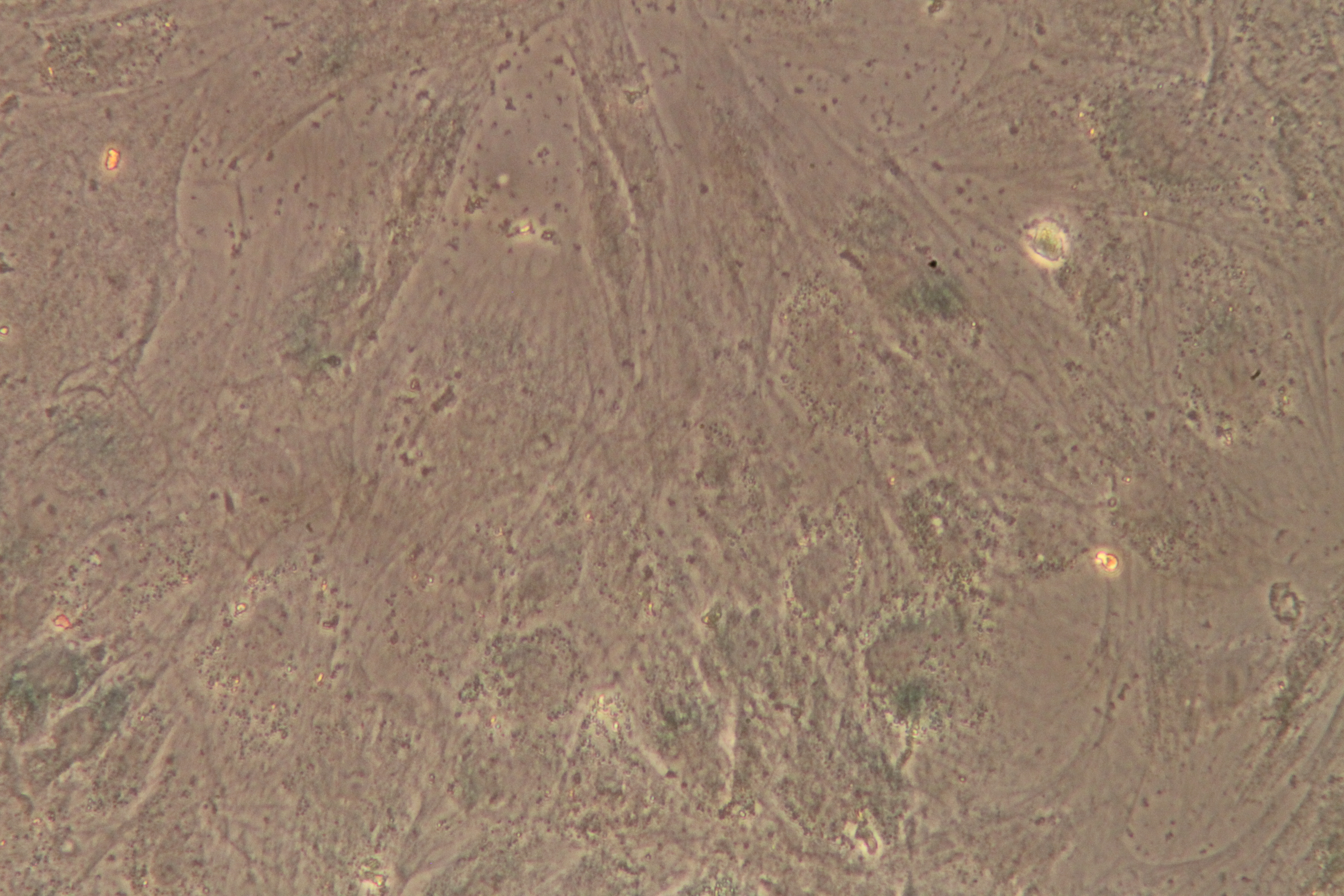

Supplement: Figure 1—source data 2. [file elife-62635-fig1-data2.zip › beta galactosidase P7/beta galactosidase P7- Aged ASCs/image 8.JPG]

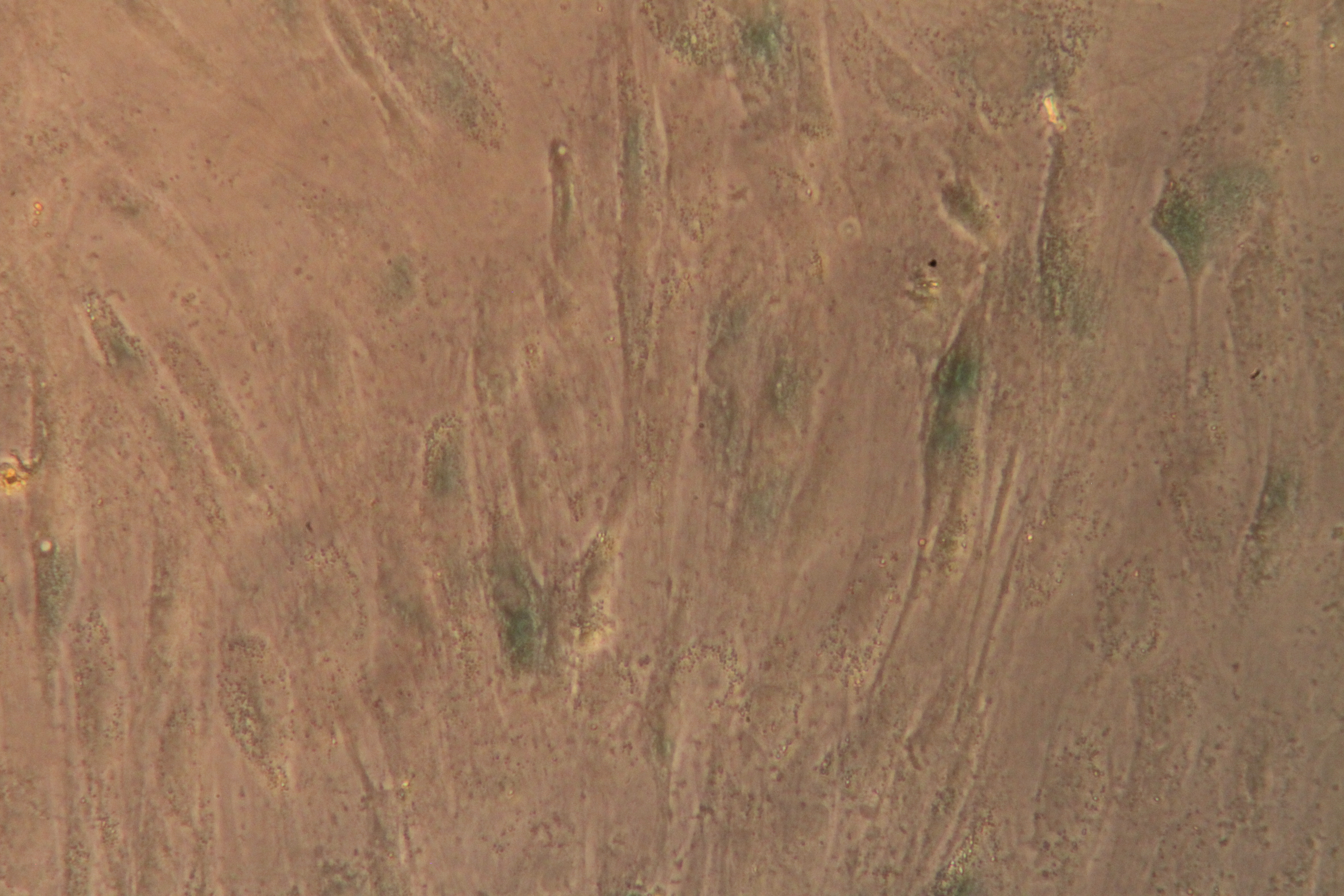

Supplement: Figure 1—source data 2. [file elife-62635-fig1-data2.zip › beta galactosidase P7/beta galactosidase P7- Aged ASCs/image 7.JPG]

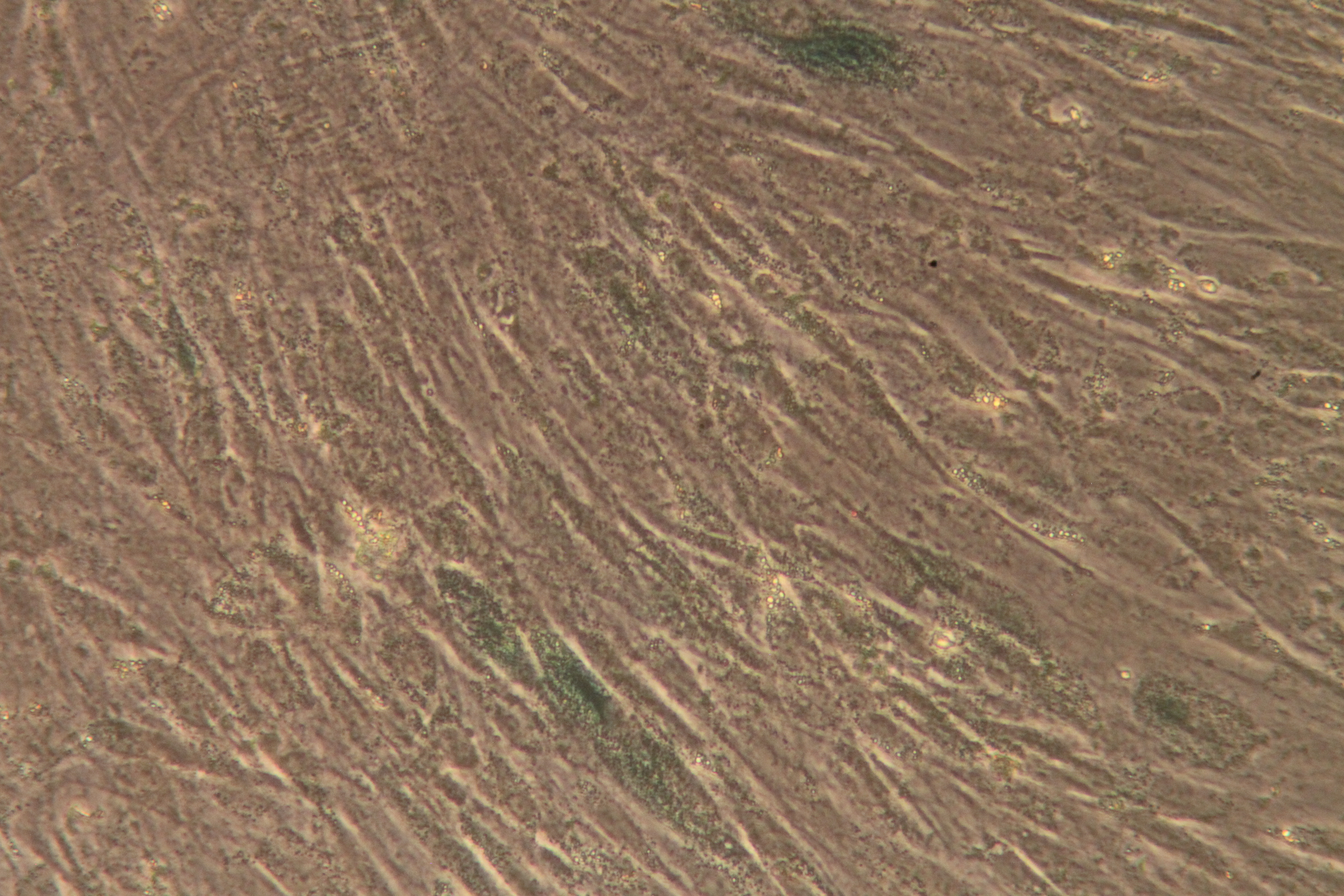

Supplement: Figure 1—source data 2. [file elife-62635-fig1-data2.zip › beta galactosidase P7/beta galactosidase P7- Aged ASCs/image 6.JPG]

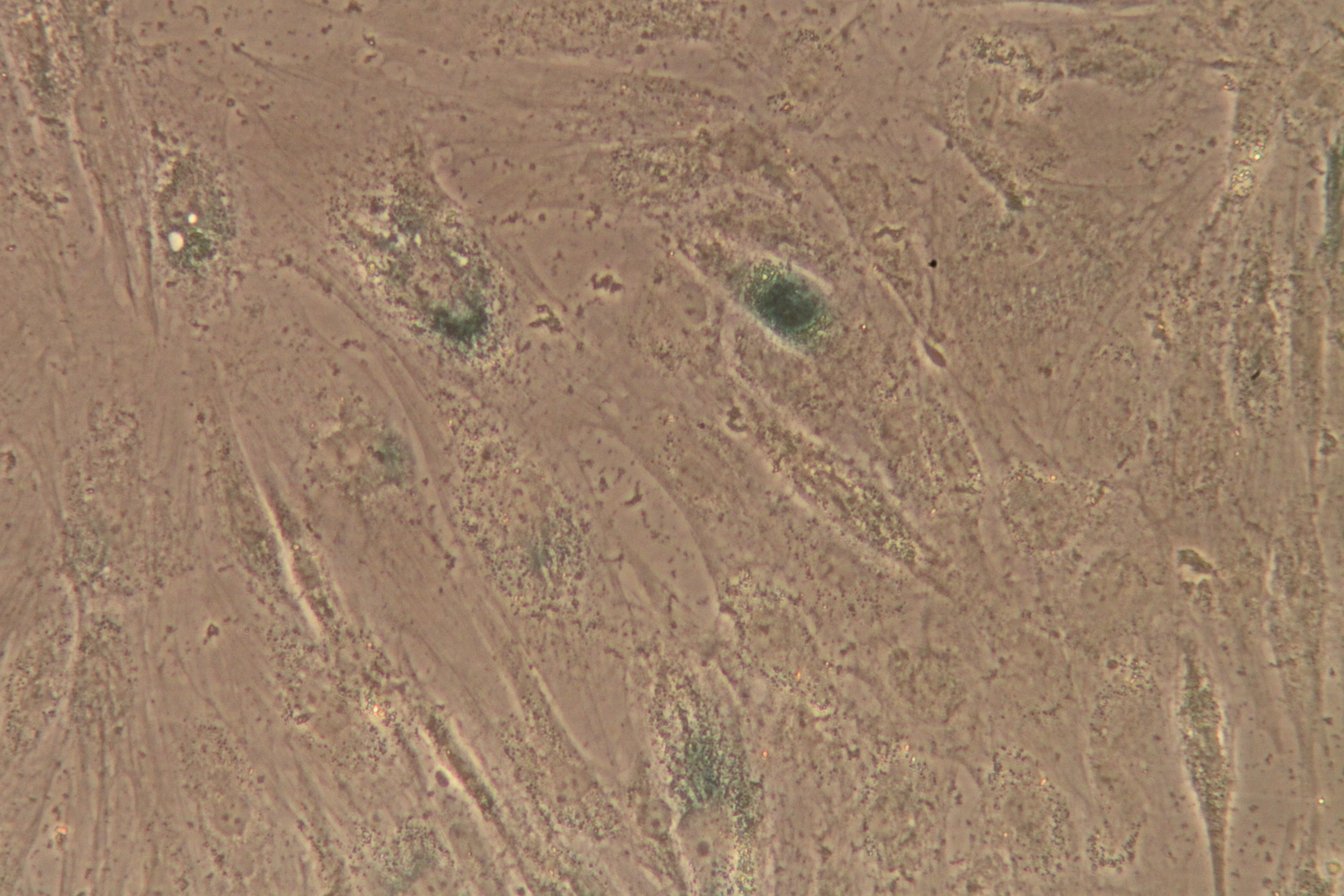

Supplement: Figure 1—source data 2. [file elife-62635-fig1-data2.zip › beta galactosidase P7/beta galactosidase P7- Aged ASCs/image 4.JPG]

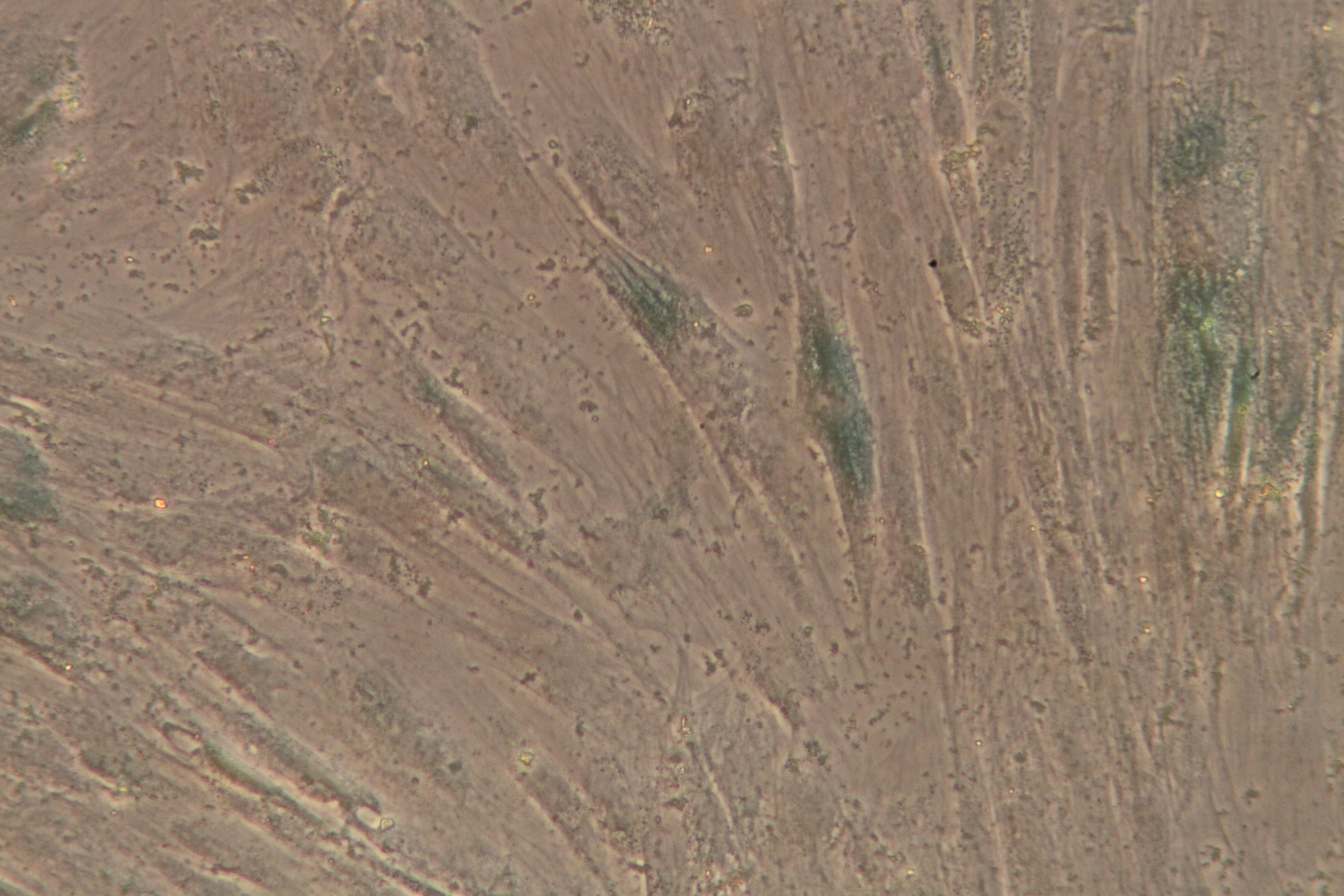

Supplement: Figure 1—source data 2. [file elife-62635-fig1-data2.zip › beta galactosidase P7/beta galactosidase P7- Aged ASCs/image 5.JPG]

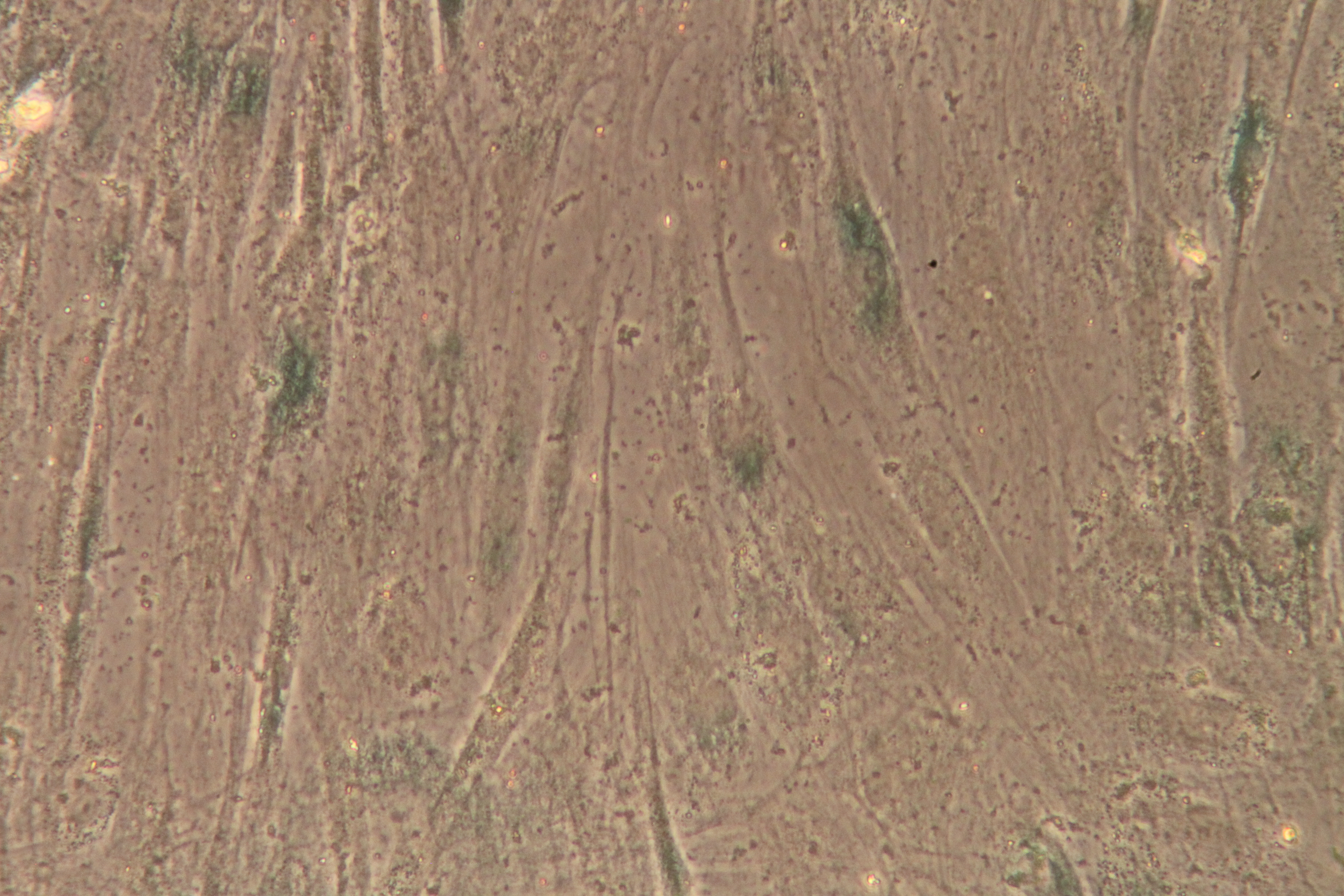

Supplement: Figure 1—source data 2. [file elife-62635-fig1-data2.zip › beta galactosidase P7/beta galactosidase P7- Aged ASCs/image 2.JPG]

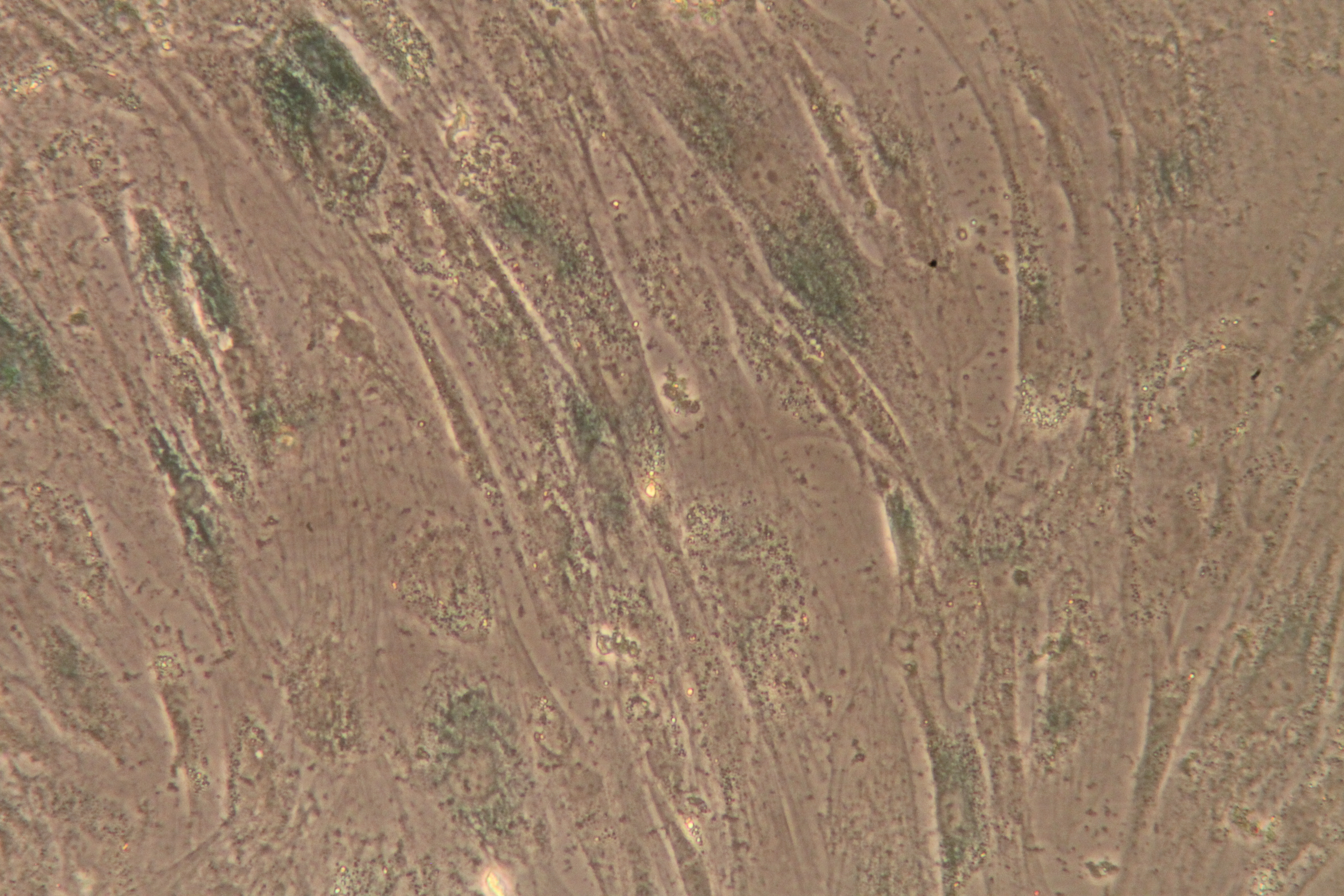

Supplement: Figure 1—source data 2. [file elife-62635-fig1-data2.zip › beta galactosidase P7/beta galactosidase P7- Aged ASCs/image 3.JPG]

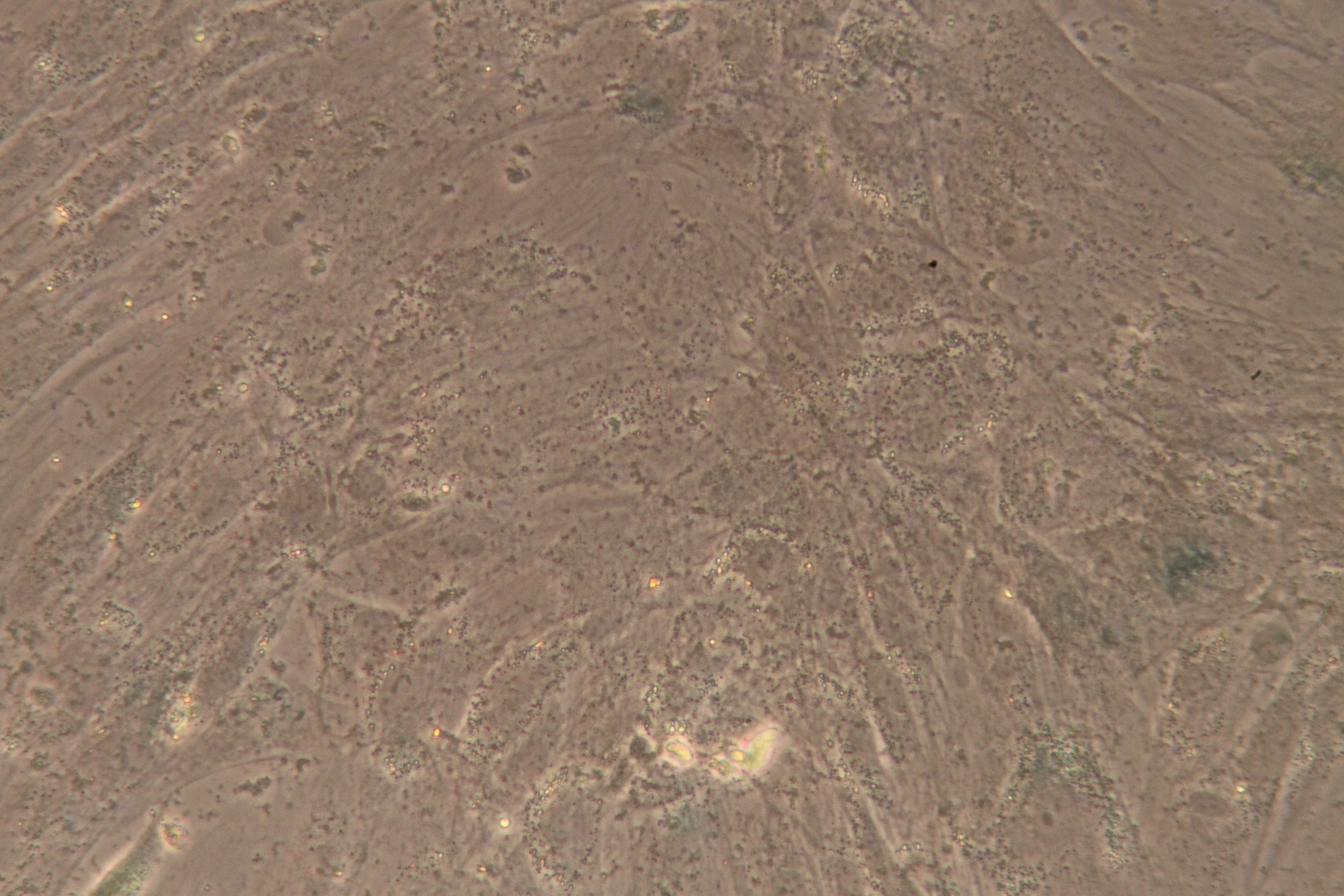

Supplement: Figure 1—source data 2. [file elife-62635-fig1-data2.zip › beta galactosidase P7/beta galactosidase P7-Young ASCs/image 1 .jpg]

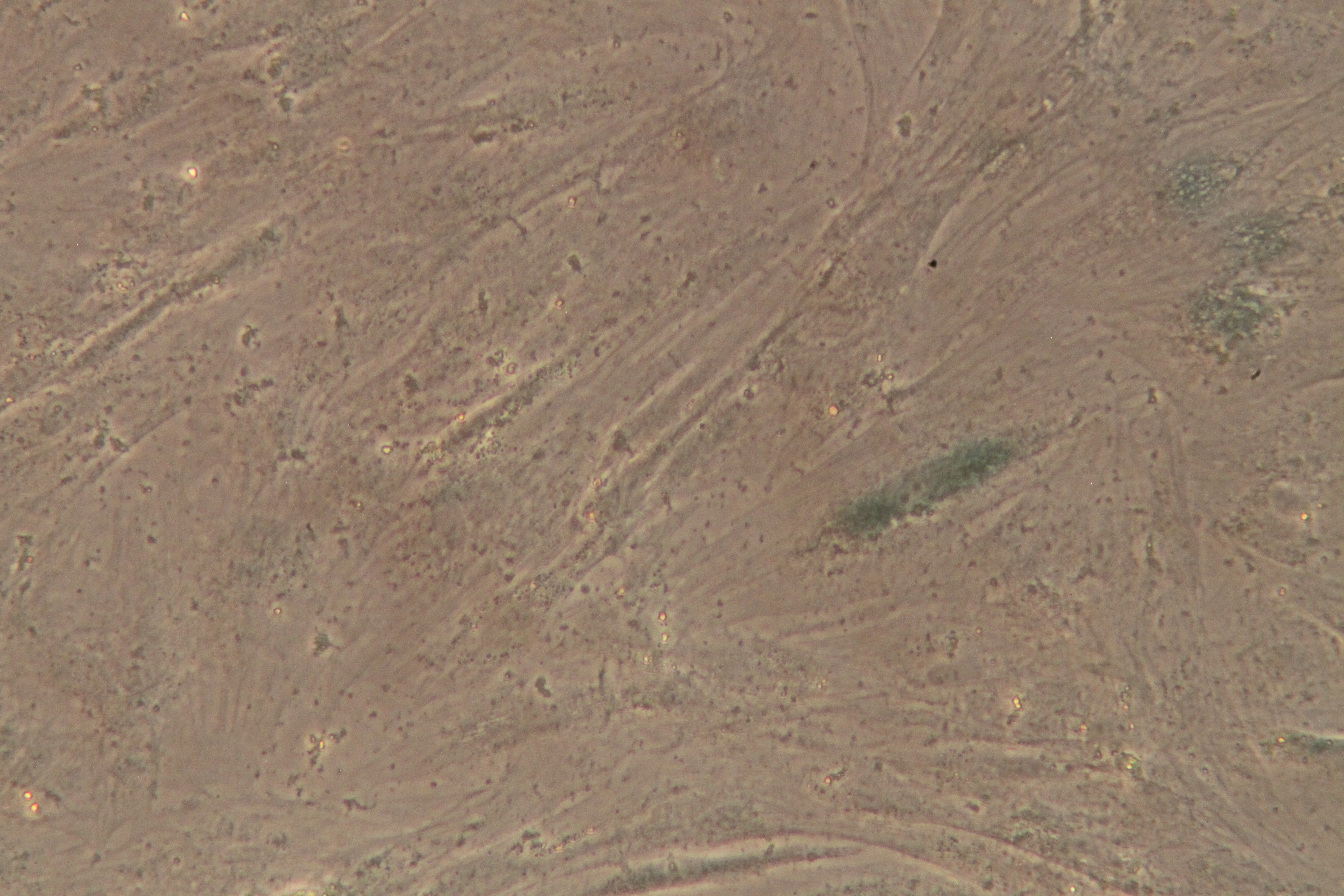

Supplement: Figure 1—source data 2. [file elife-62635-fig1-data2.zip › beta galactosidase P7/beta galactosidase P7-Young ASCs/image 8.JPG]

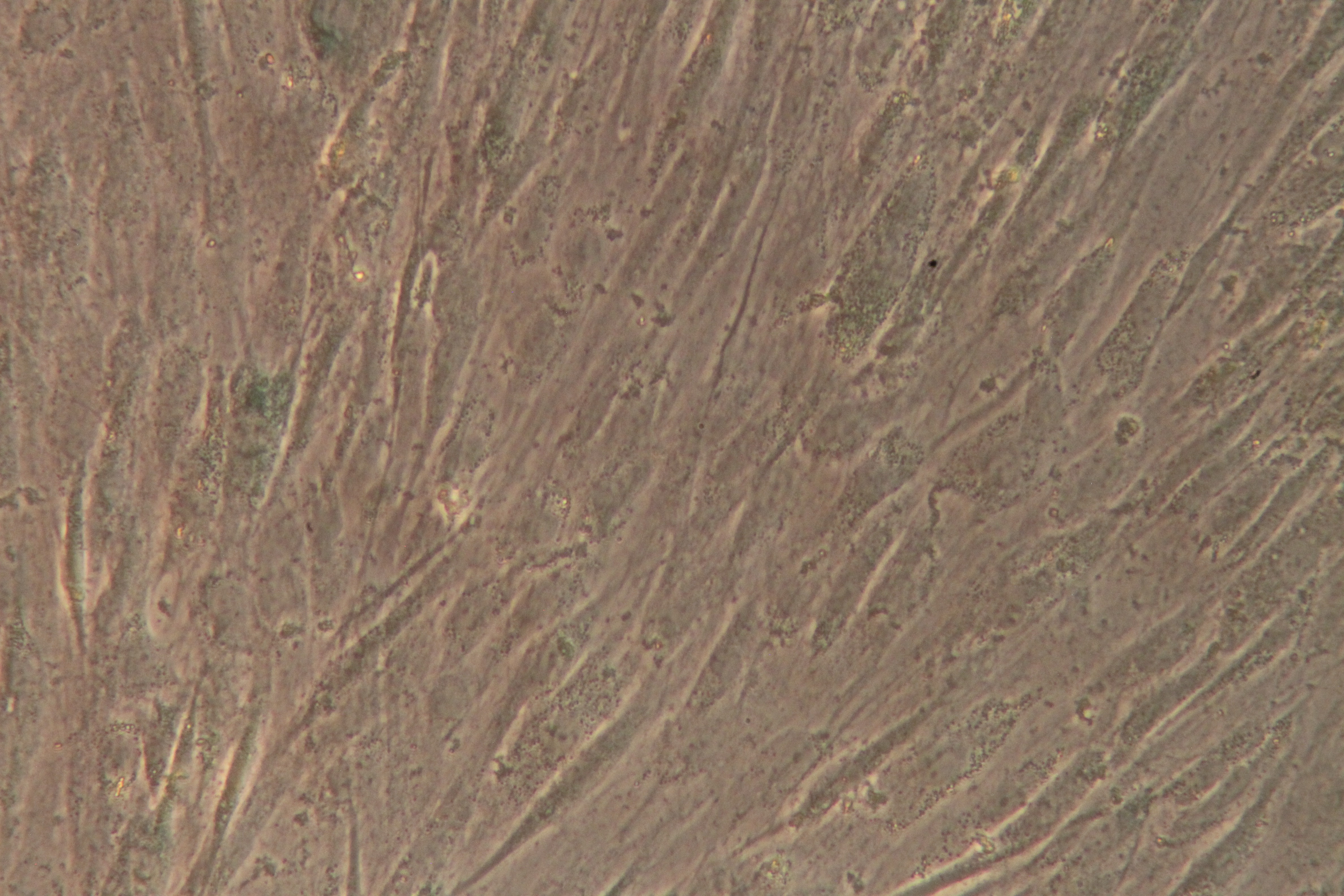

Supplement: Figure 1—source data 2. [file elife-62635-fig1-data2.zip › beta galactosidase P7/beta galactosidase P7-Young ASCs/image 7.jpg]

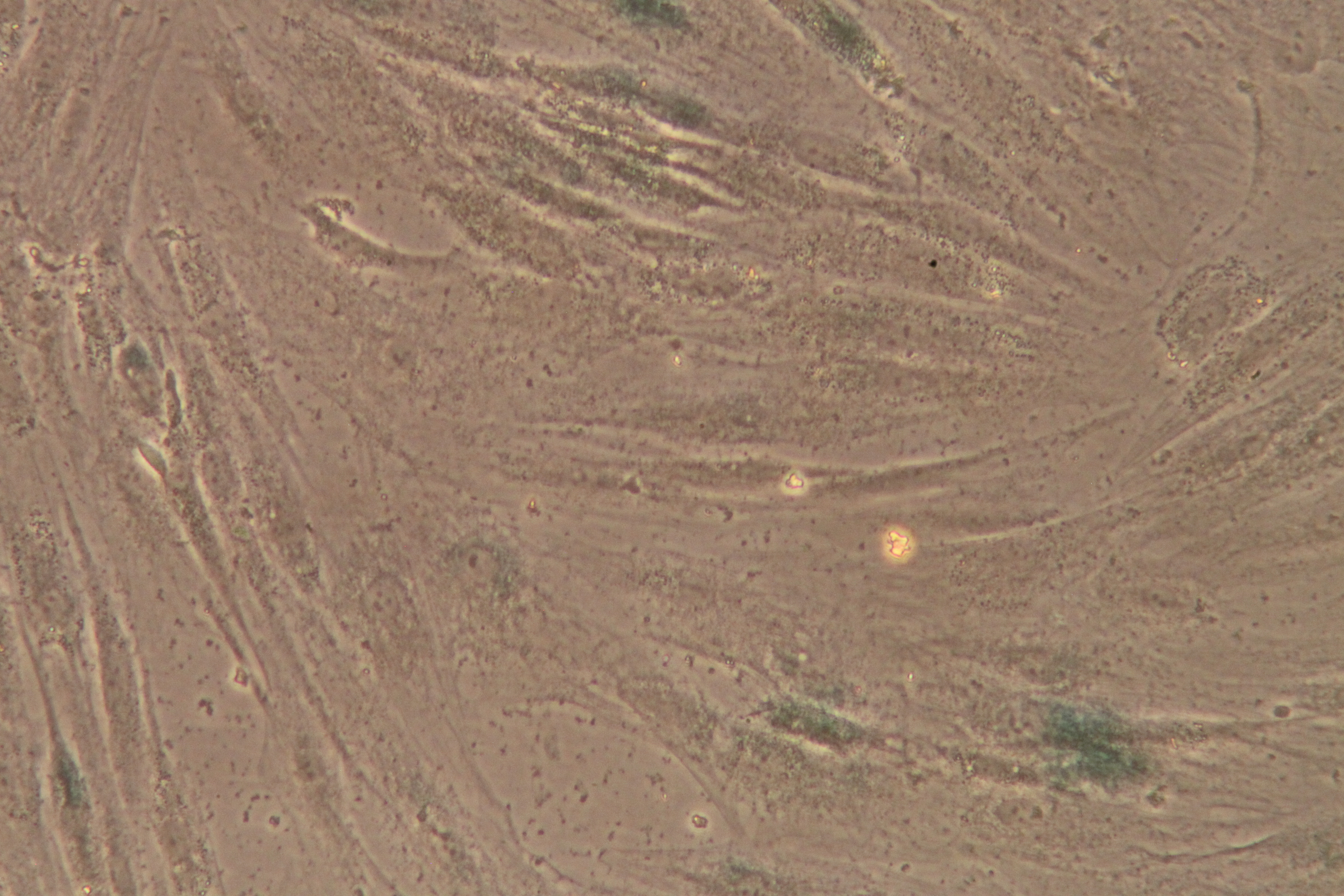

Supplement: Figure 1—source data 2. [file elife-62635-fig1-data2.zip › beta galactosidase P7/beta galactosidase P7-Young ASCs/image 6.JPG]

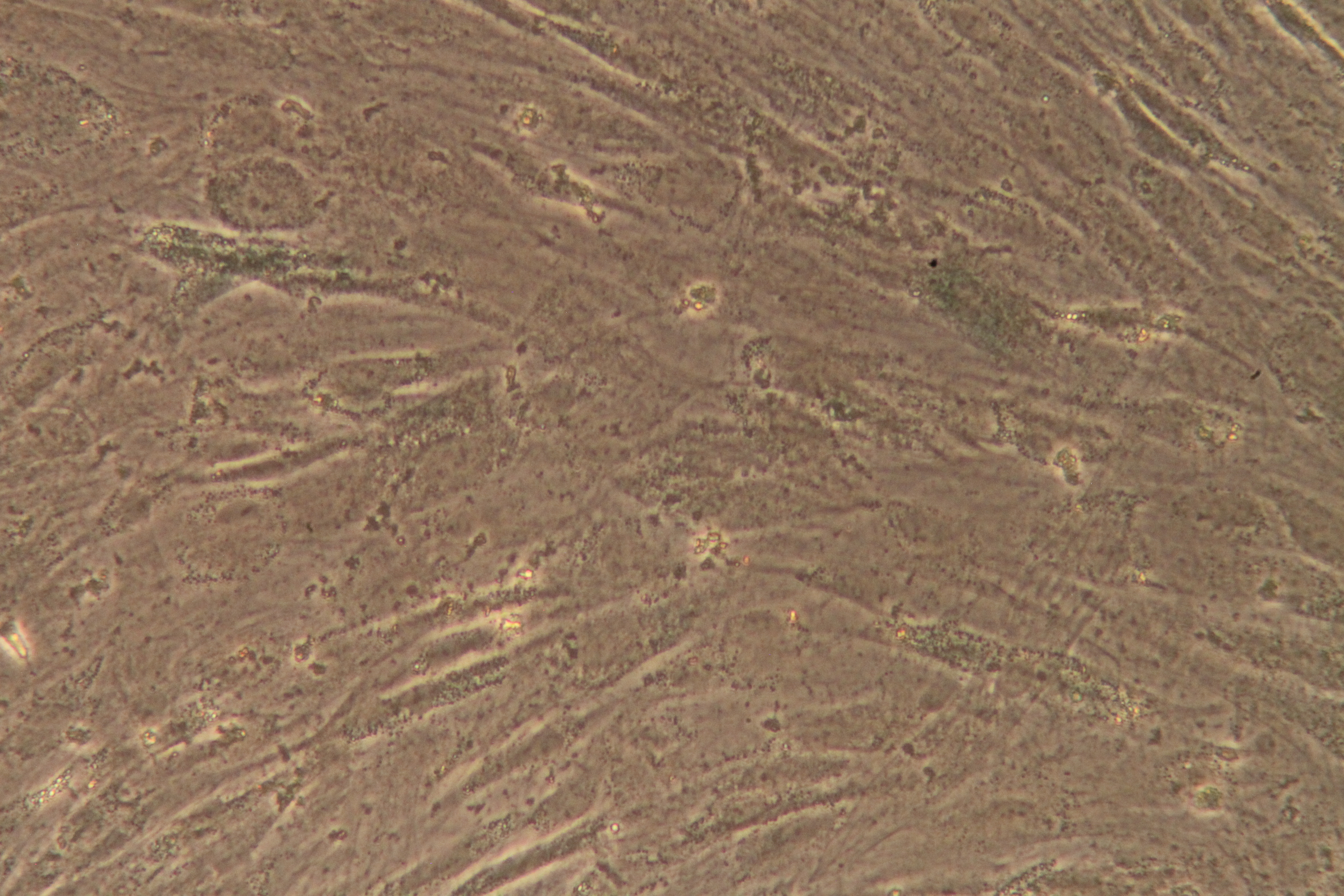

Supplement: Figure 1—source data 2. [file elife-62635-fig1-data2.zip › beta galactosidase P7/beta galactosidase P7-Young ASCs/image 4.JPG]

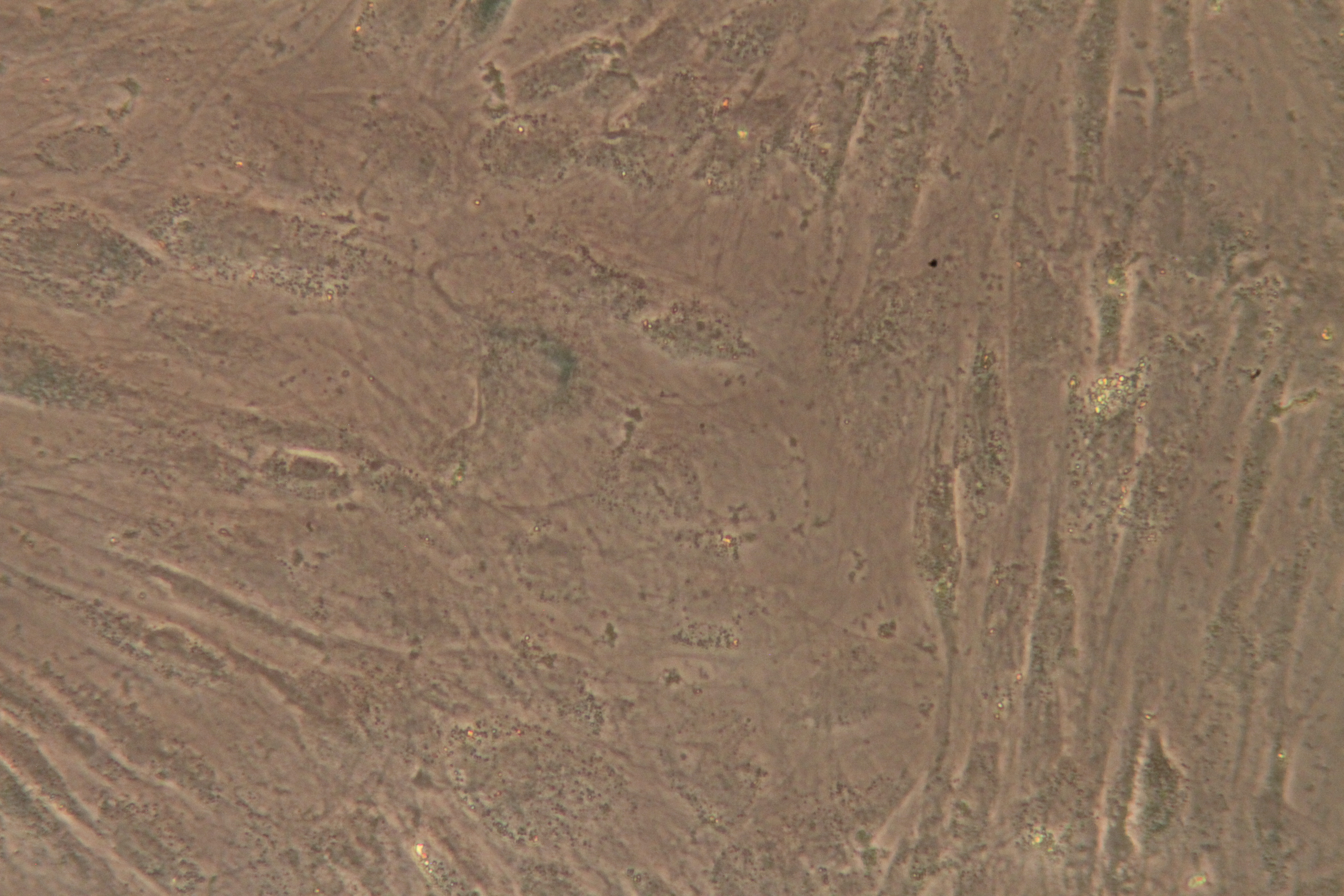

Supplement: Figure 1—source data 2. [file elife-62635-fig1-data2.zip › beta galactosidase P7/beta galactosidase P7-Young ASCs/image 5.JPG]

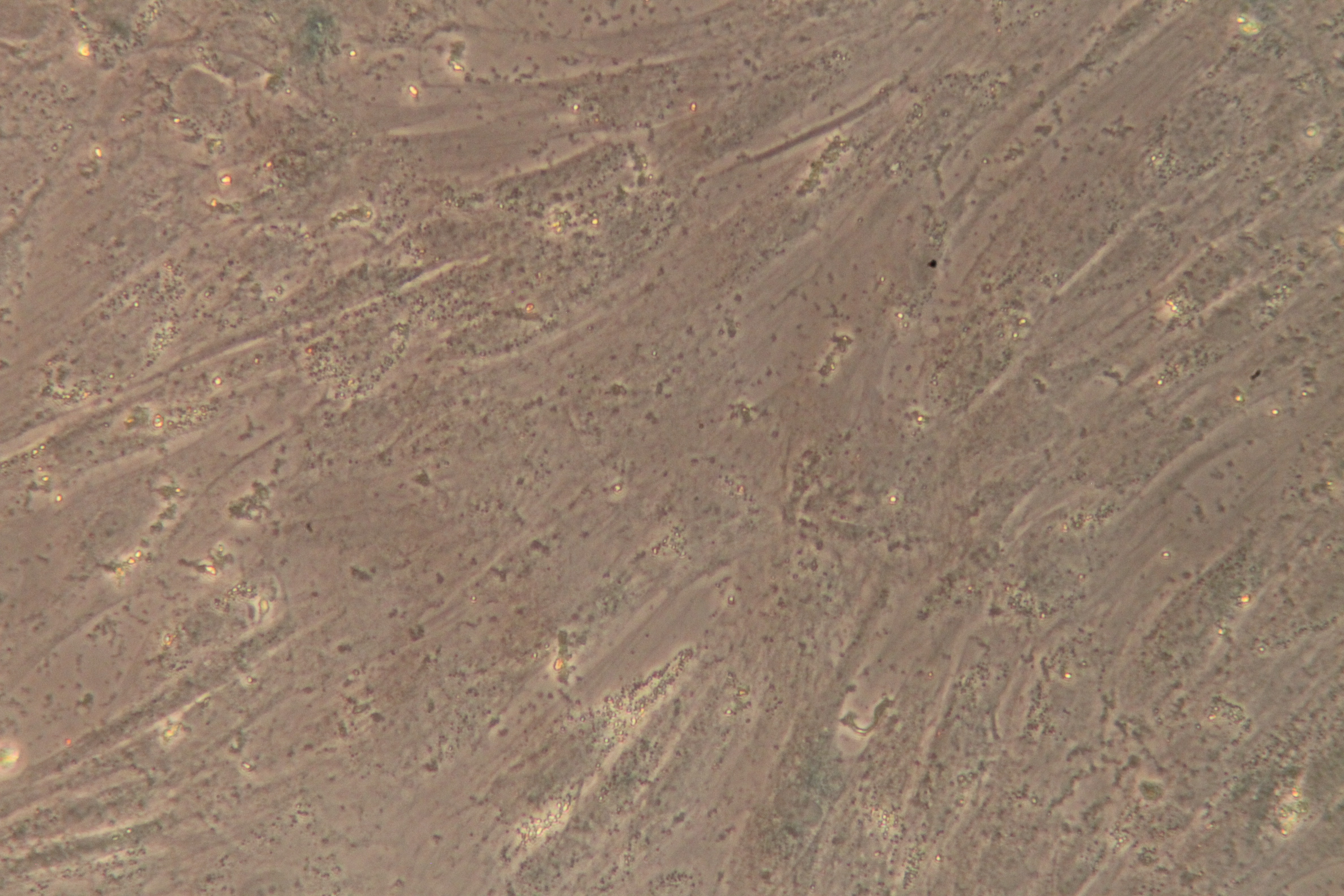

Supplement: Figure 1—source data 2. [file elife-62635-fig1-data2.zip › beta galactosidase P7/beta galactosidase P7-Young ASCs/image 2 .jpg]

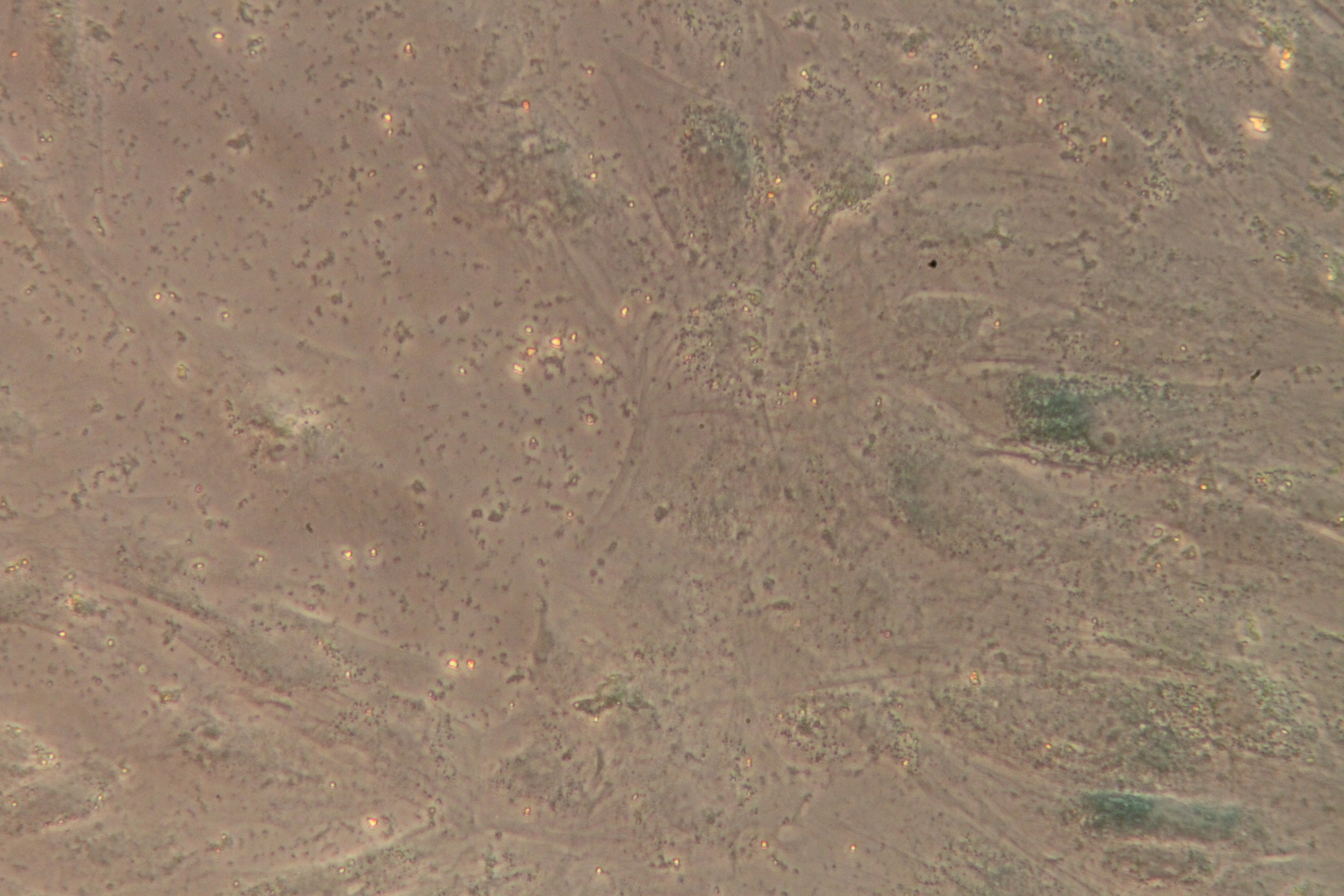

Supplement: Figure 1—source data 2. [file elife-62635-fig1-data2.zip › beta galactosidase P7/beta galactosidase P7-Young ASCs/image 3.JPG]

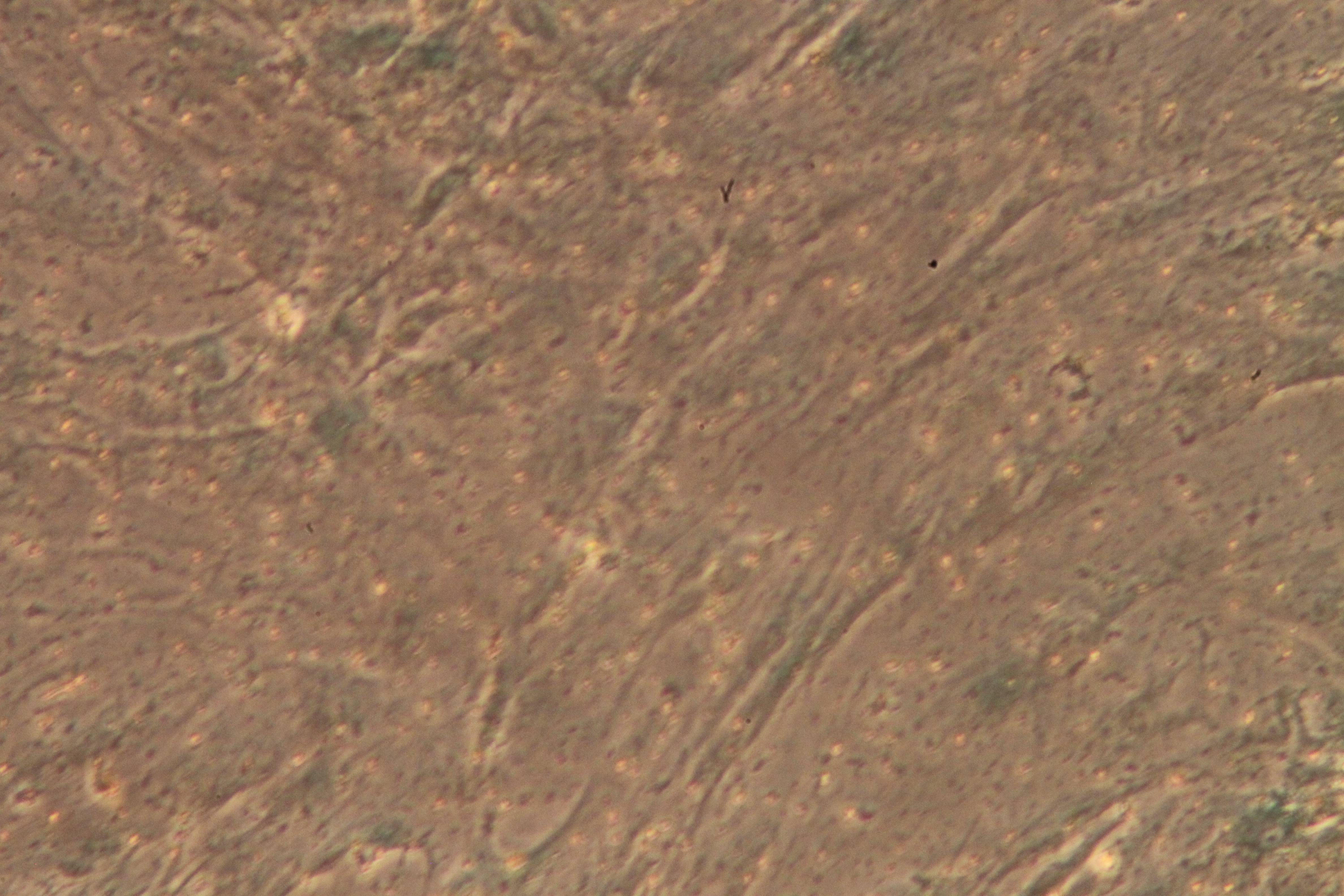

Supplement: Figure 1—source data 3. [file elife-62635-fig1-data3.zip › beta galastosidase P11/beta galactosidase P11-Aged ASCs/image 1 .jpg]

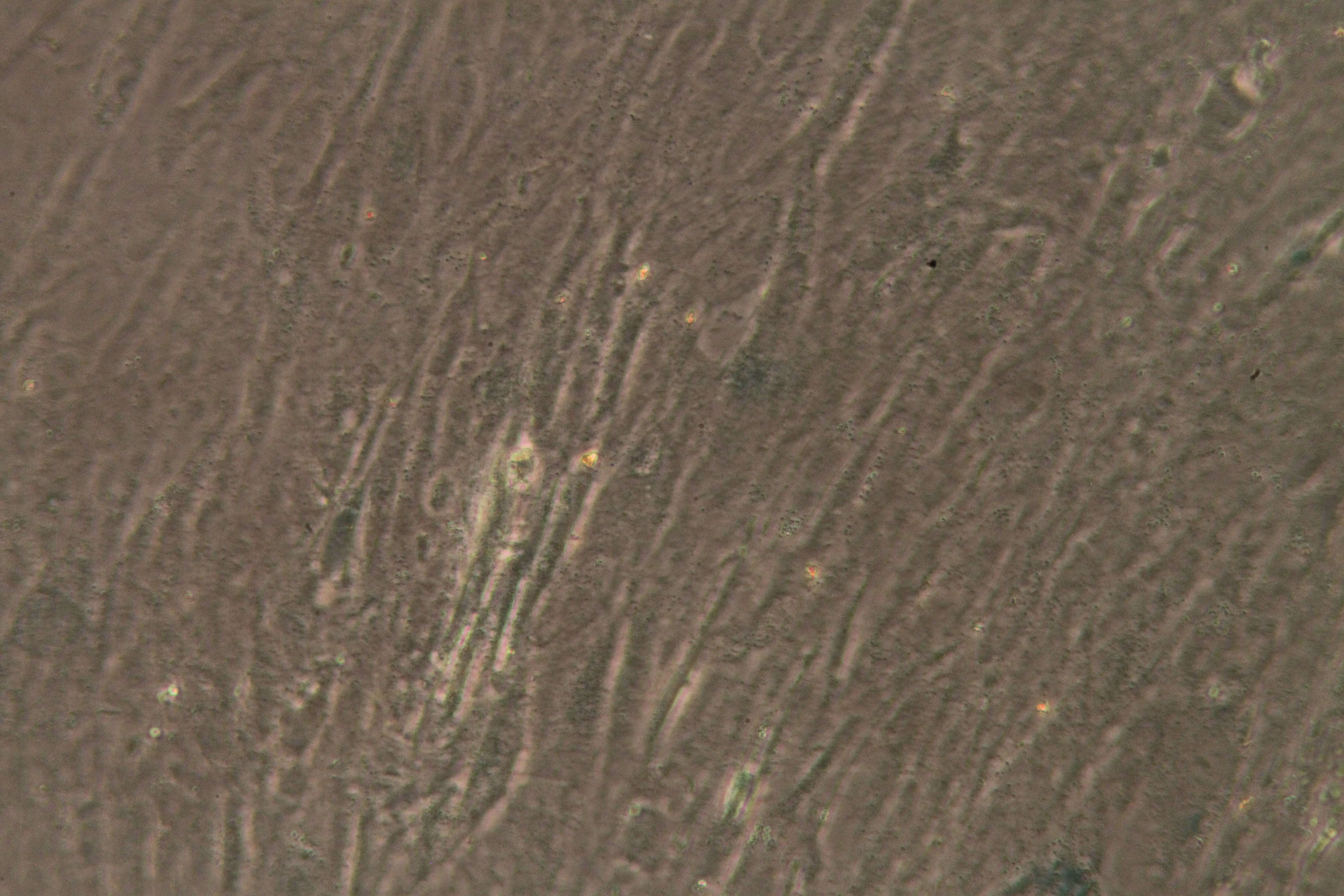

Supplement: Figure 1—source data 3. [file elife-62635-fig1-data3.zip › beta galastosidase P11/beta galactosidase P11-Aged ASCs/image 8.JPG]

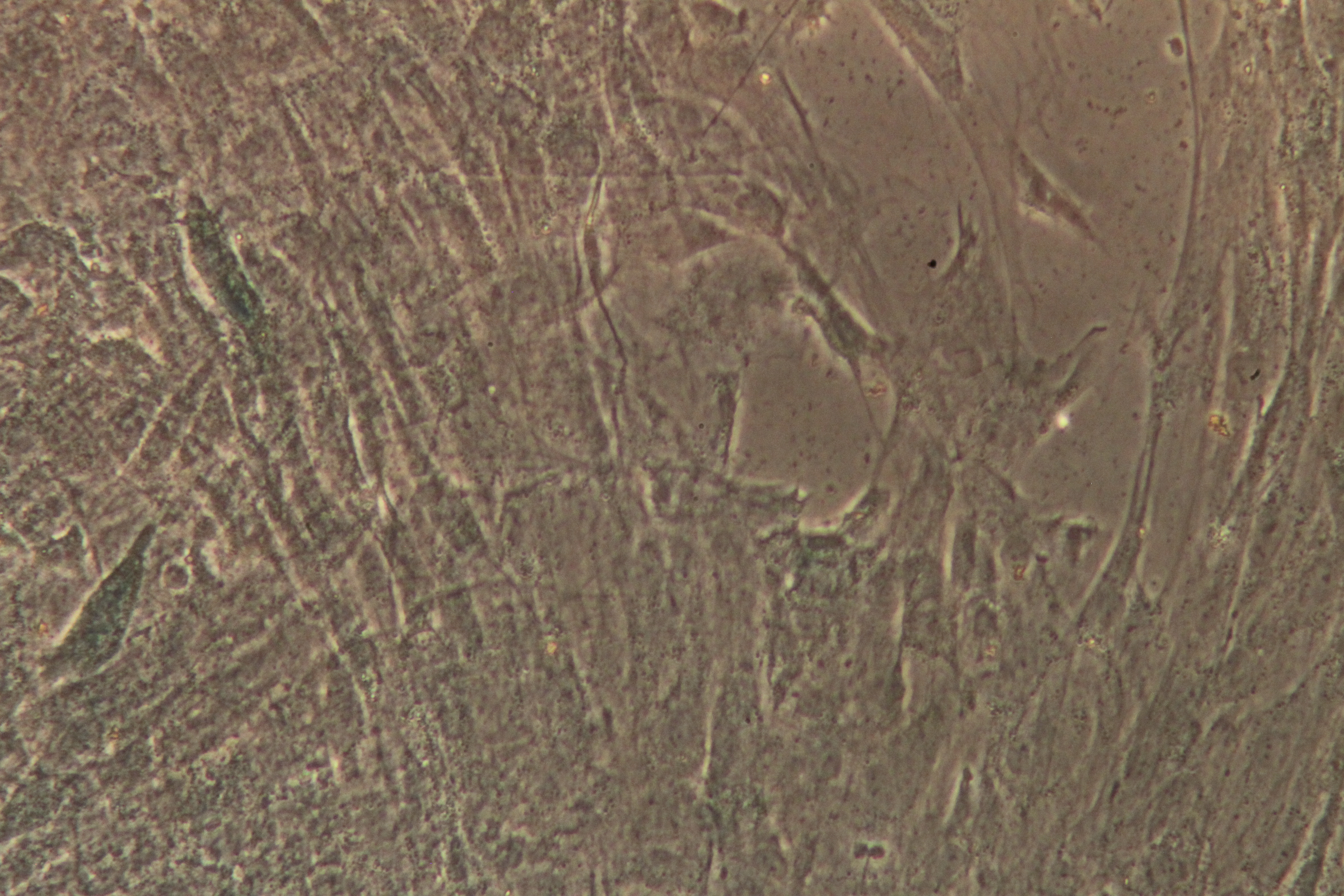

Supplement: Figure 1—source data 3. [file elife-62635-fig1-data3.zip › beta galastosidase P11/beta galactosidase P11-Aged ASCs/image 7.JPG]

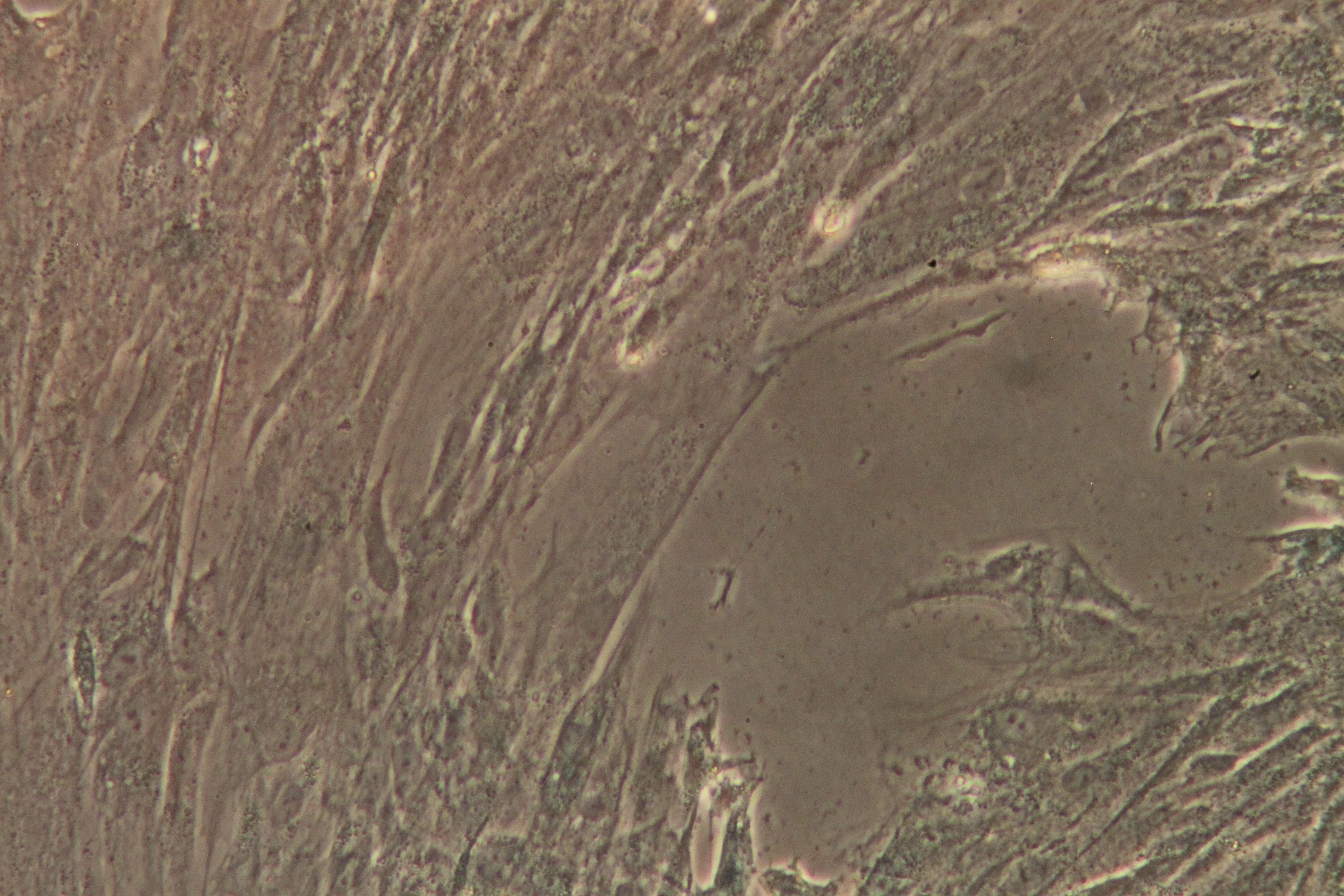

Supplement: Figure 1—source data 3. [file elife-62635-fig1-data3.zip › beta galastosidase P11/beta galactosidase P11-Aged ASCs/image 6.JPG]

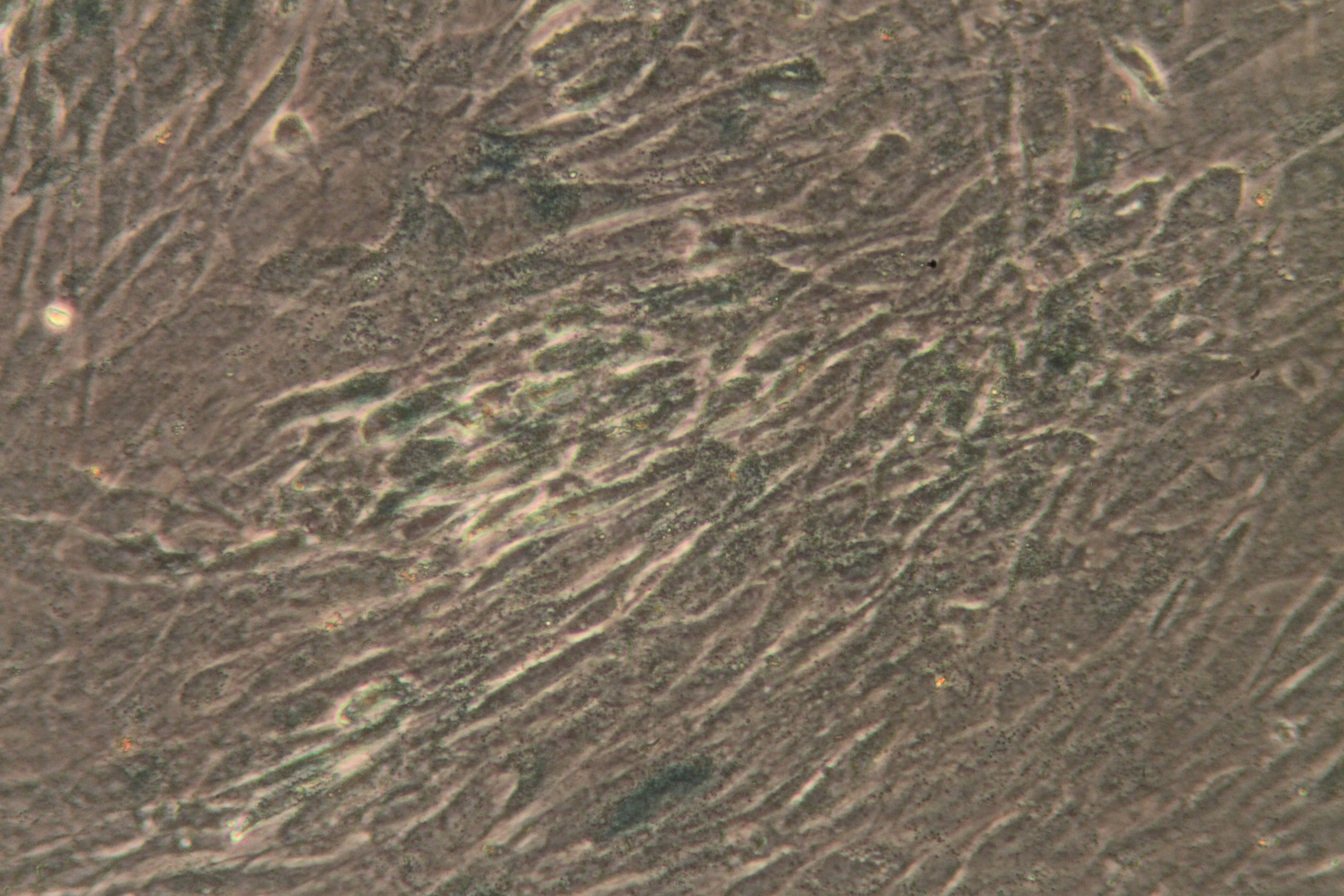

Supplement: Figure 1—source data 3. [file elife-62635-fig1-data3.zip › beta galastosidase P11/beta galactosidase P11-Aged ASCs/image 4.JPG]

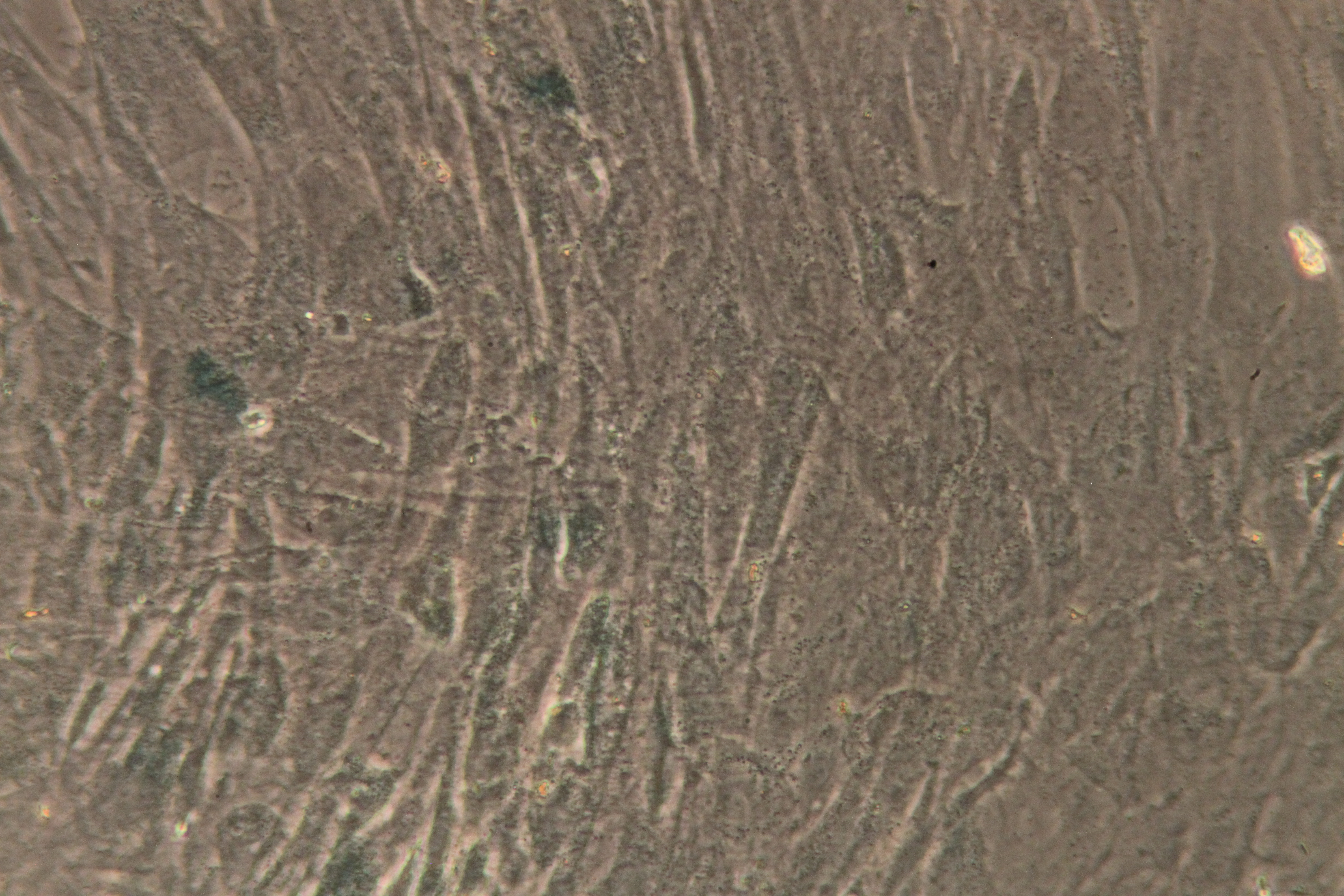

Supplement: Figure 1—source data 3. [file elife-62635-fig1-data3.zip › beta galastosidase P11/beta galactosidase P11-Aged ASCs/image 5.JPG]

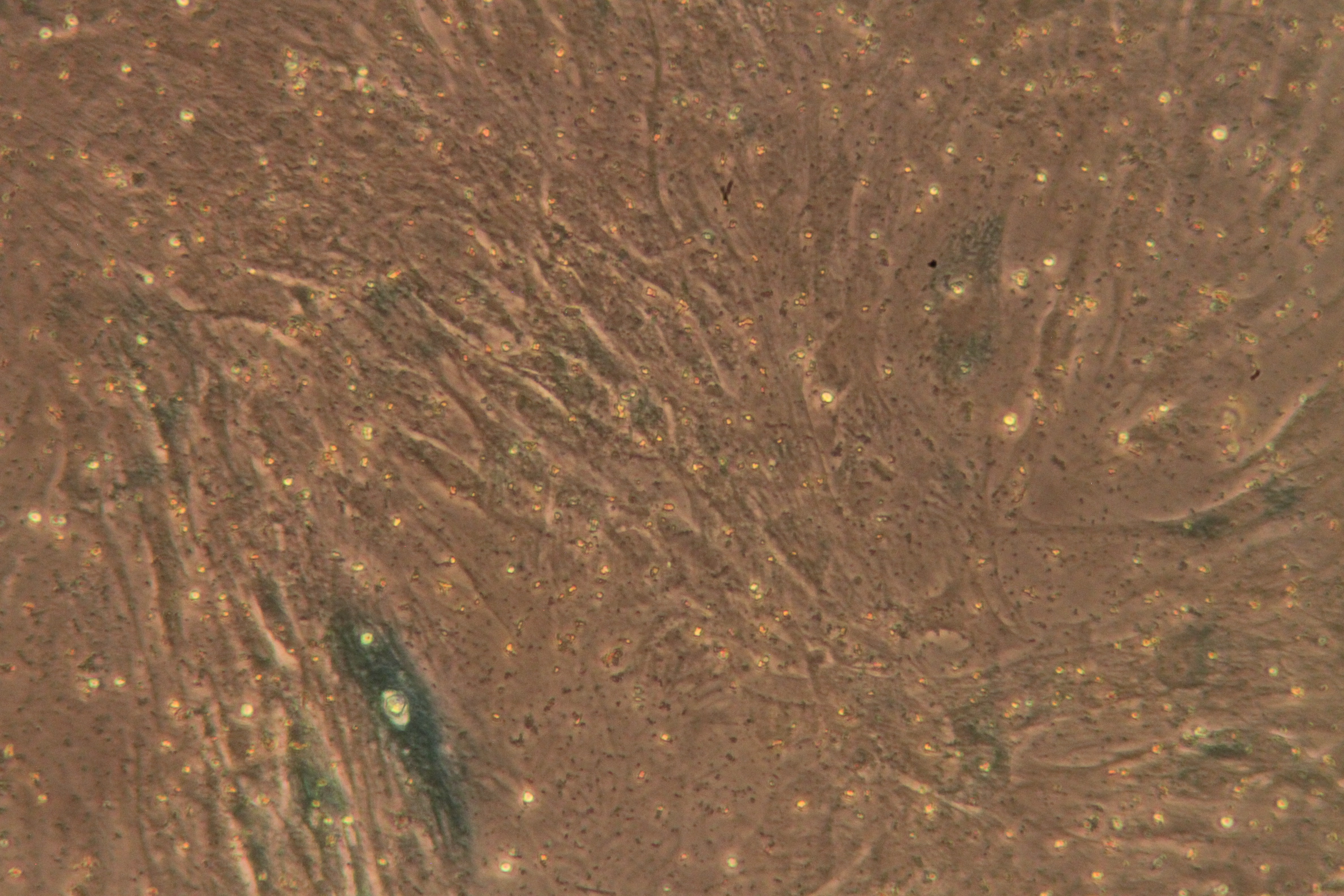

Supplement: Figure 1—source data 3. [file elife-62635-fig1-data3.zip › beta galastosidase P11/beta galactosidase P11-Aged ASCs/image 2 .jpg]

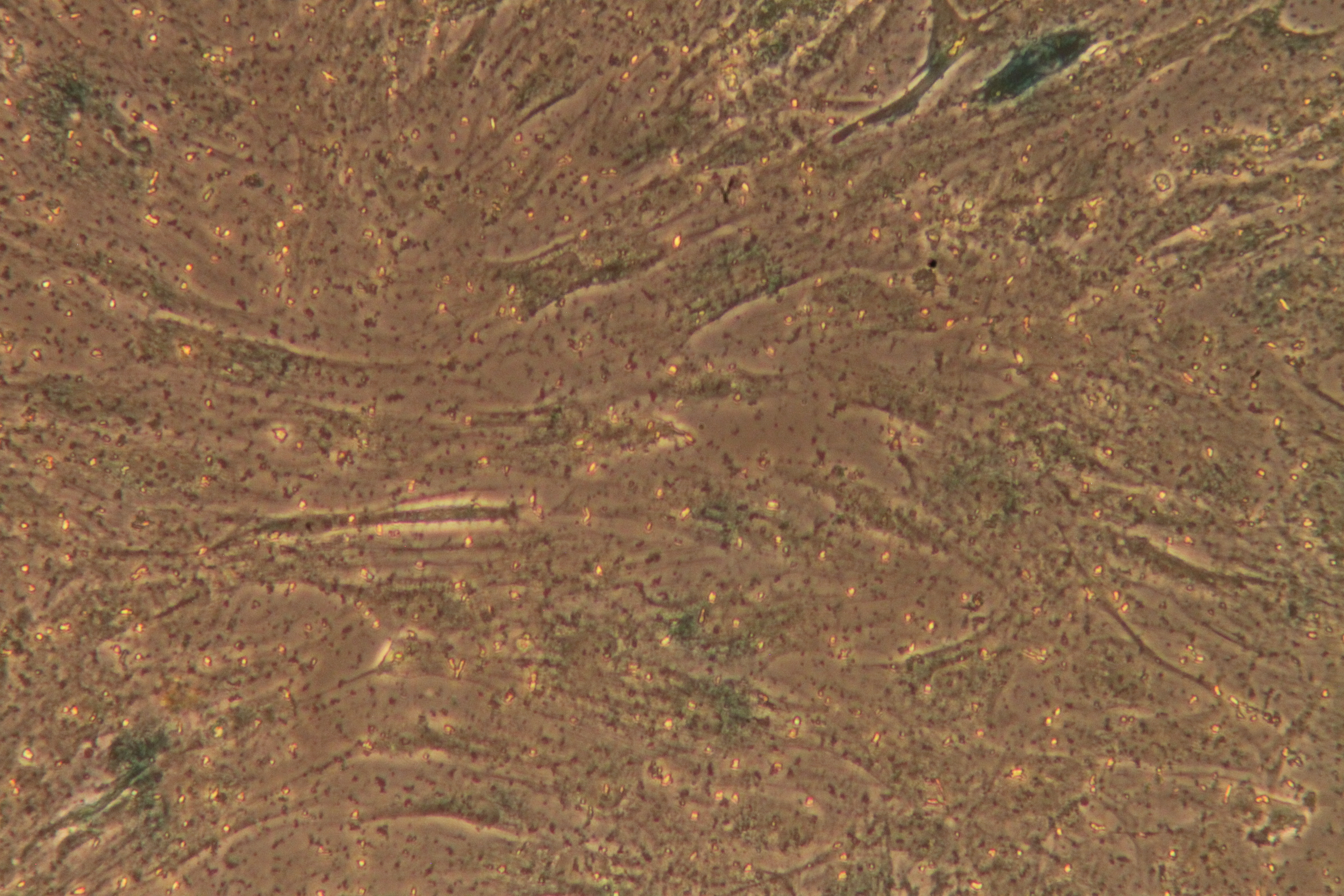

Supplement: Figure 1—source data 3. [file elife-62635-fig1-data3.zip › beta galastosidase P11/beta galactosidase P11-Aged ASCs/image 3.JPG]

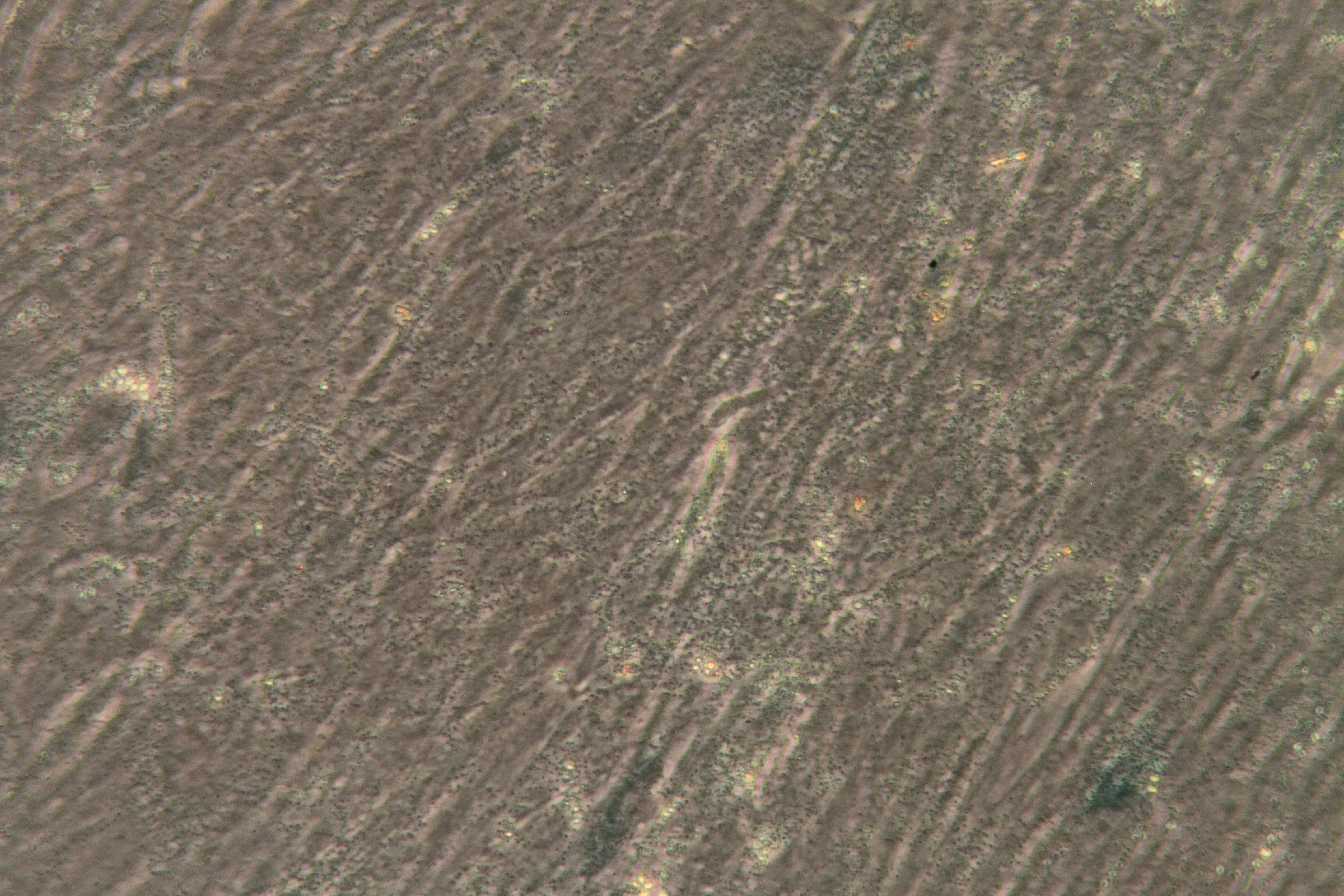

Supplement: Figure 1—source data 3. [file elife-62635-fig1-data3.zip › beta galastosidase P11/beta galactosidase P11-Young ASCs/image 1 .jpg]

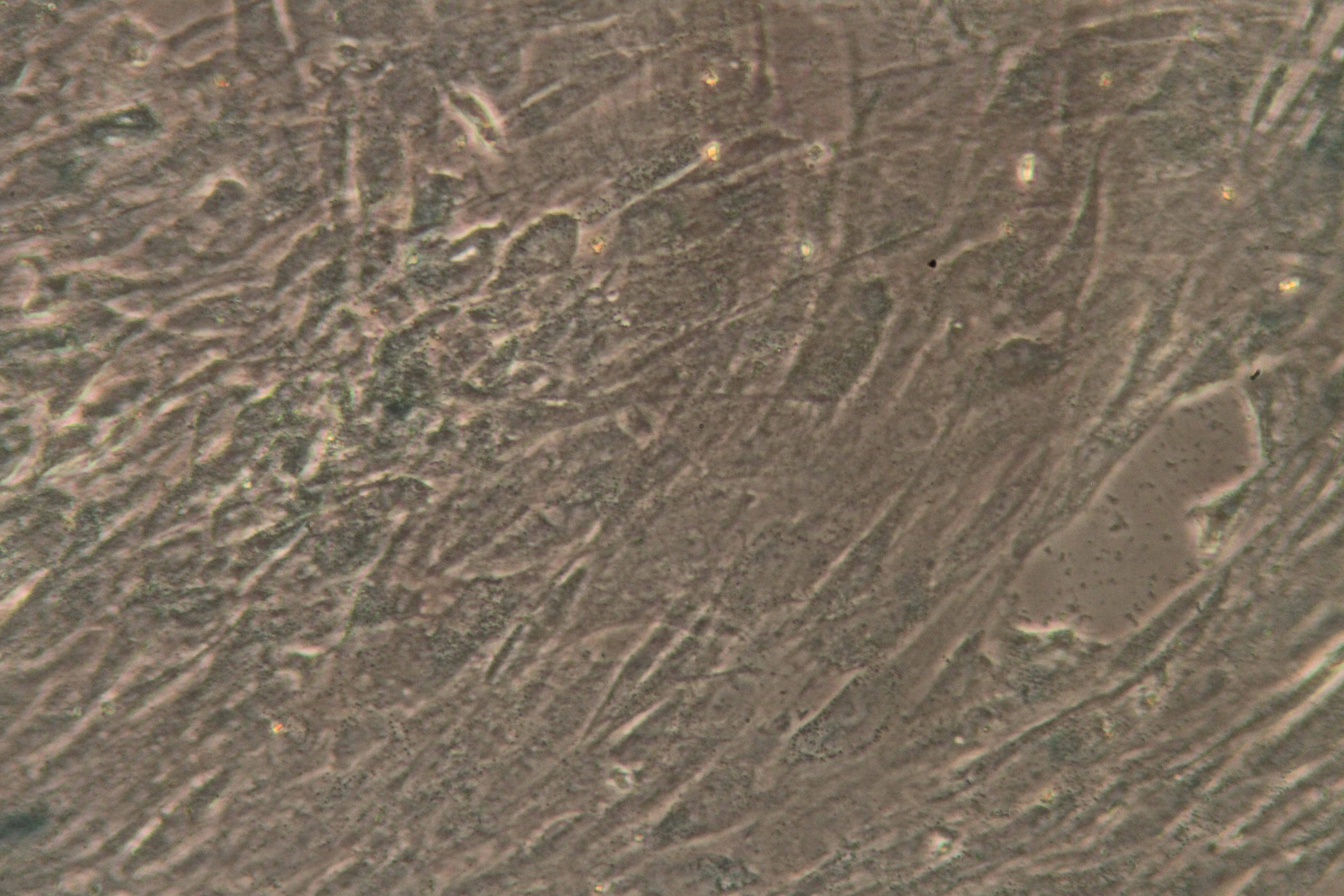

Supplement: Figure 1—source data 3. [file elife-62635-fig1-data3.zip › beta galastosidase P11/beta galactosidase P11-Young ASCs/image 8.JPG]

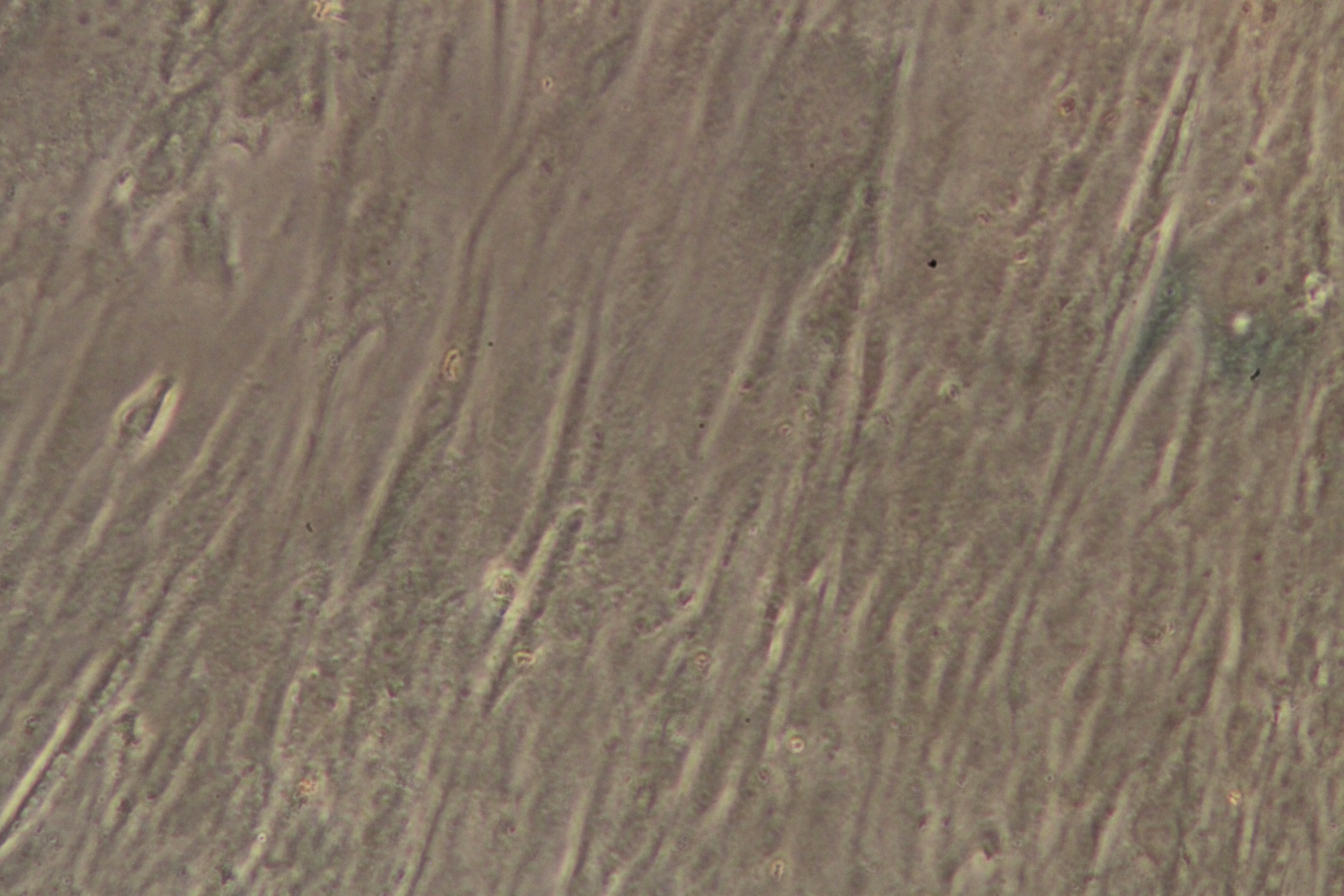

Supplement: Figure 1—source data 3. [file elife-62635-fig1-data3.zip › beta galastosidase P11/beta galactosidase P11-Young ASCs/image 7.JPG]

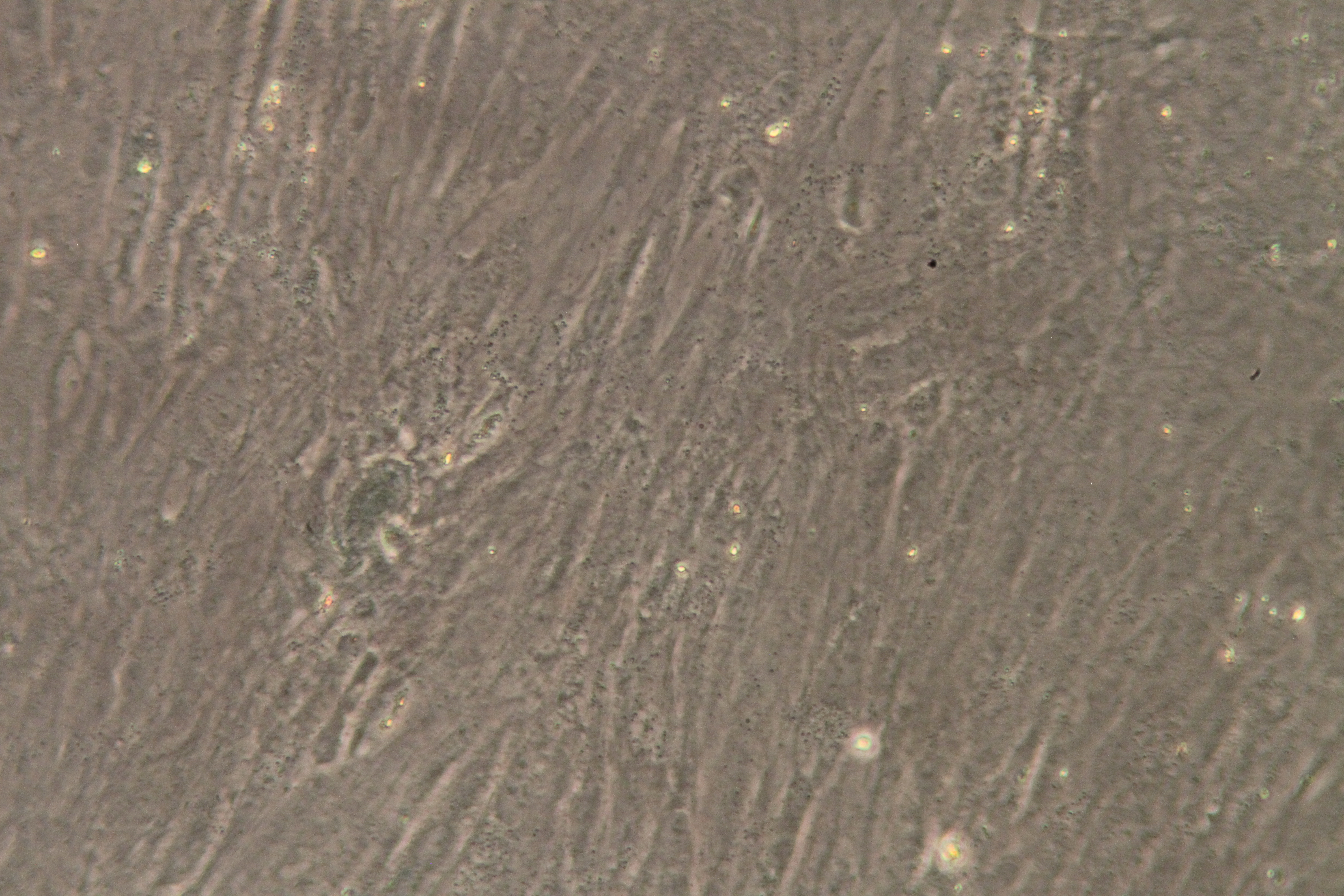

Supplement: Figure 1—source data 3. [file elife-62635-fig1-data3.zip › beta galastosidase P11/beta galactosidase P11-Young ASCs/image 6.JPG]

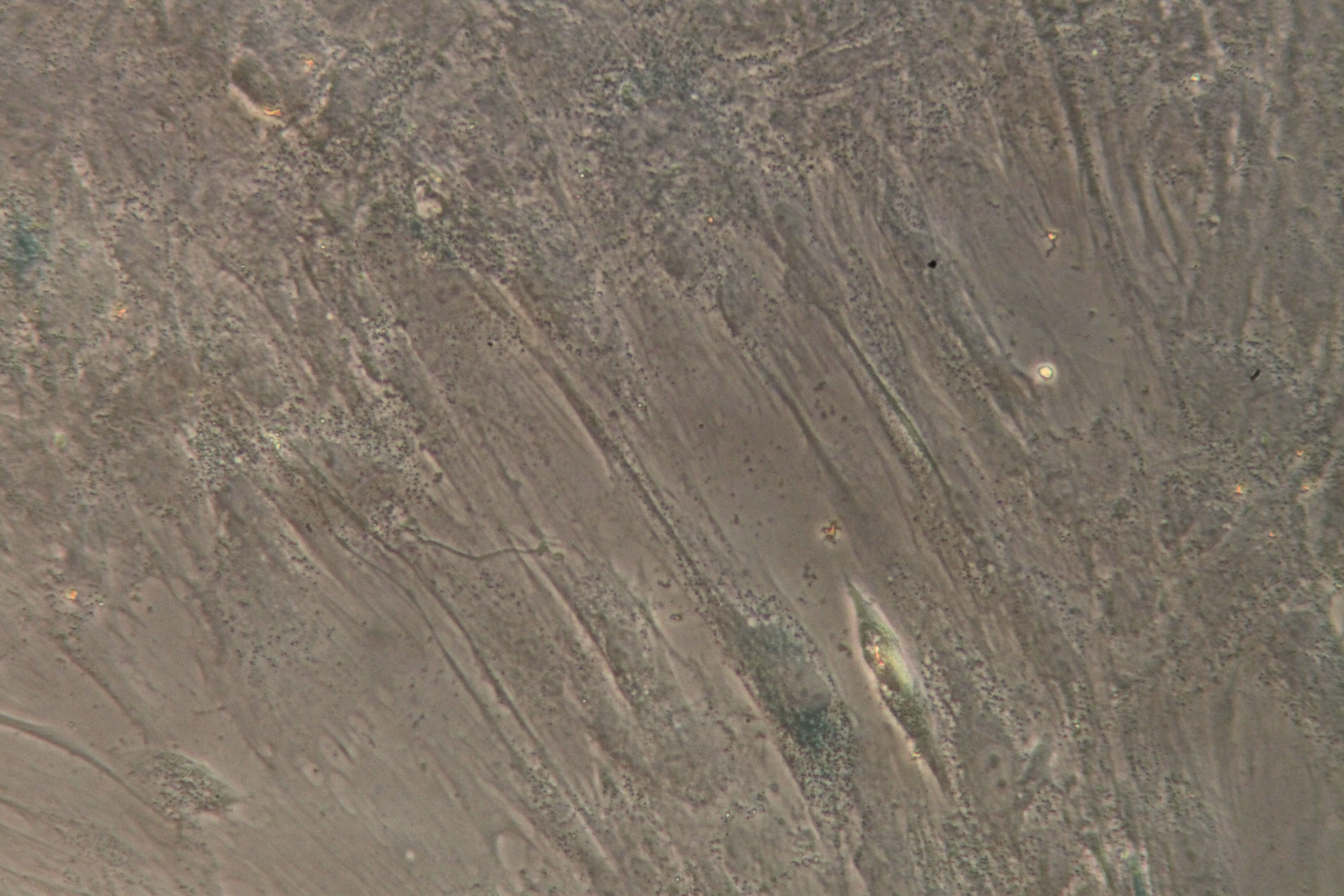

Supplement: Figure 1—source data 3. [file elife-62635-fig1-data3.zip › beta galastosidase P11/beta galactosidase P11-Young ASCs/image 3 .jpg]

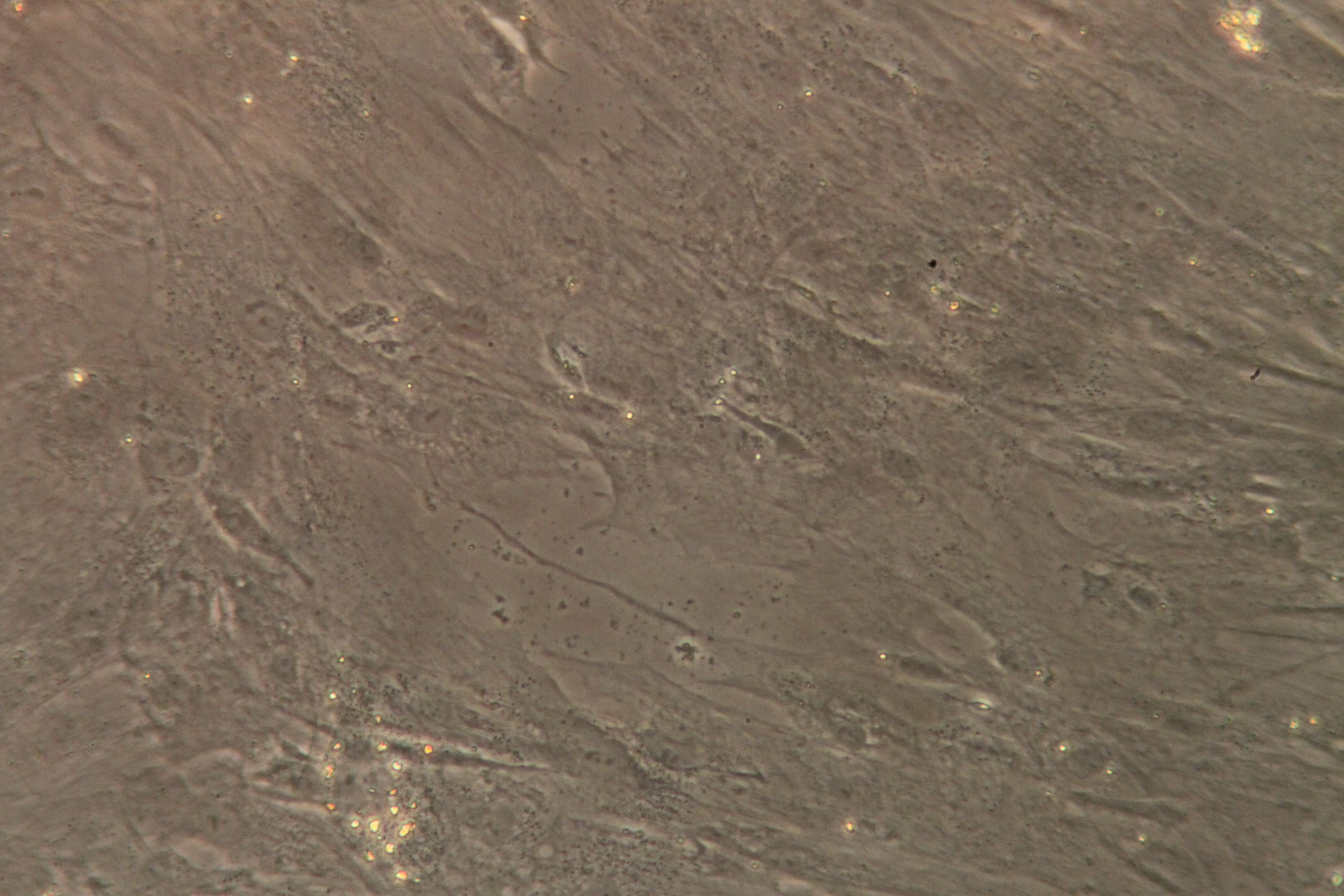

Supplement: Figure 1—source data 3. [file elife-62635-fig1-data3.zip › beta galastosidase P11/beta galactosidase P11-Young ASCs/image 4.JPG]

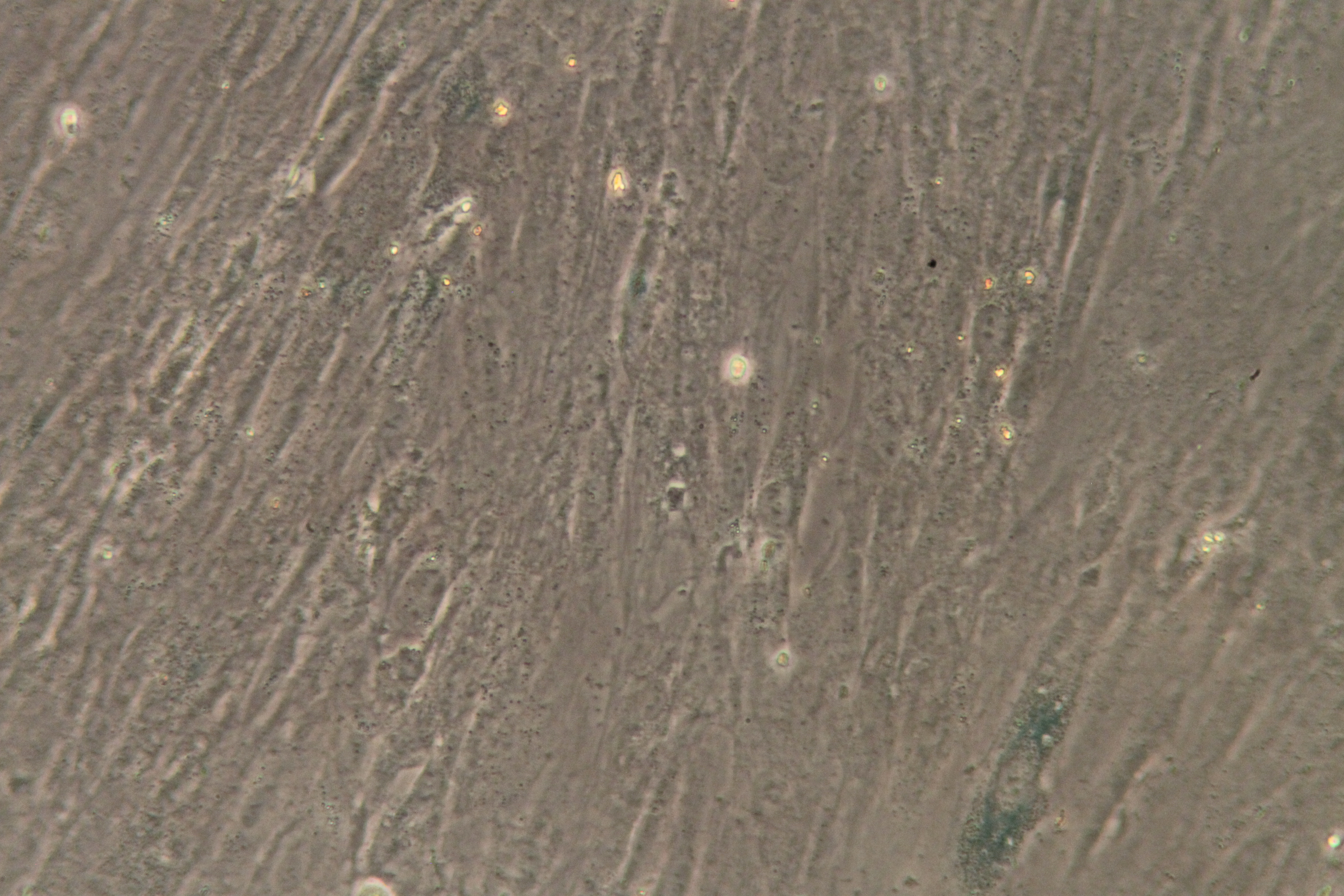

Supplement: Figure 1—source data 3. [file elife-62635-fig1-data3.zip › beta galastosidase P11/beta galactosidase P11-Young ASCs/image 5.JPG]

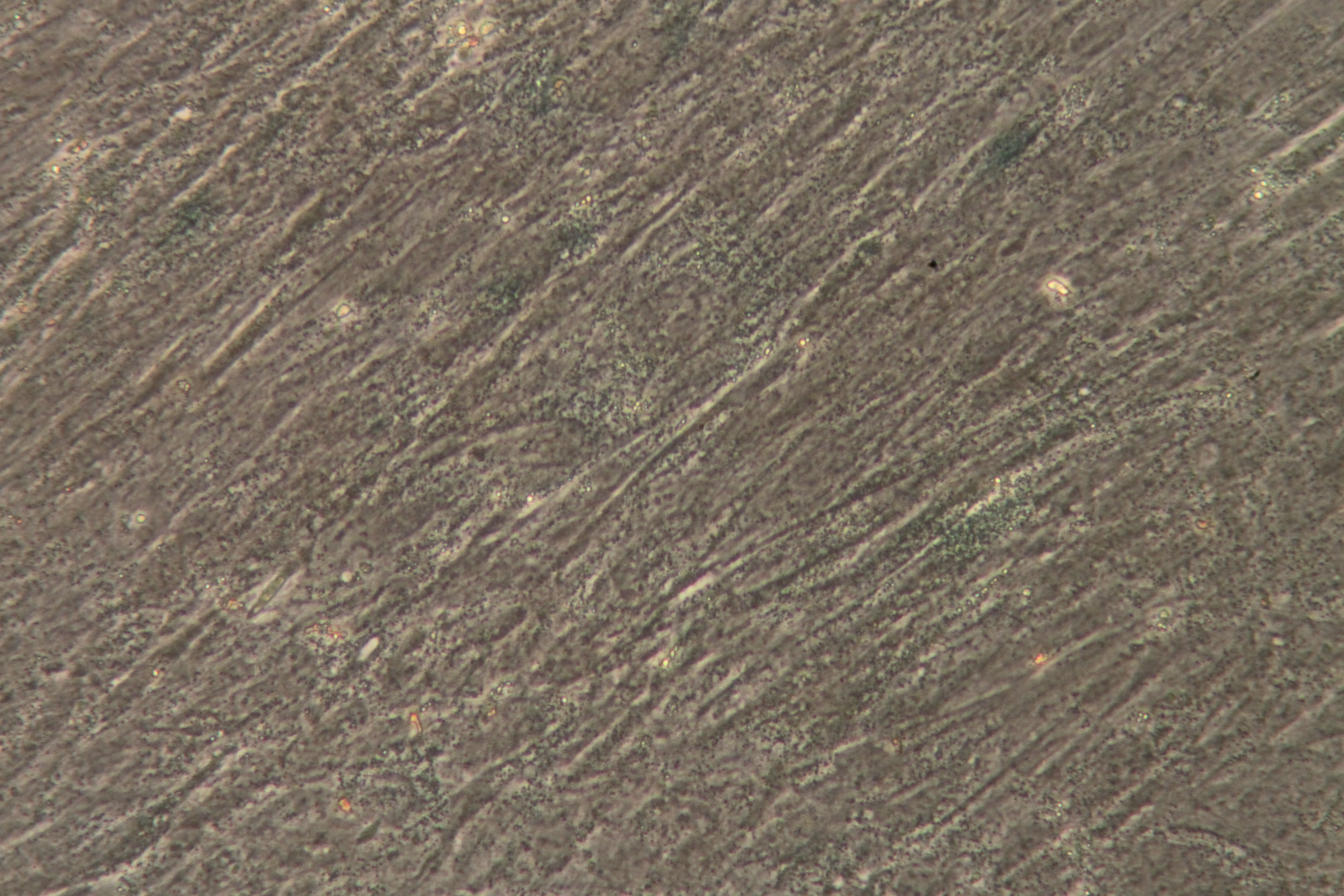

Supplement: Figure 1—source data 3. [file elife-62635-fig1-data3.zip › beta galastosidase P11/beta galactosidase P11-Young ASCs/image 2 .jpg]

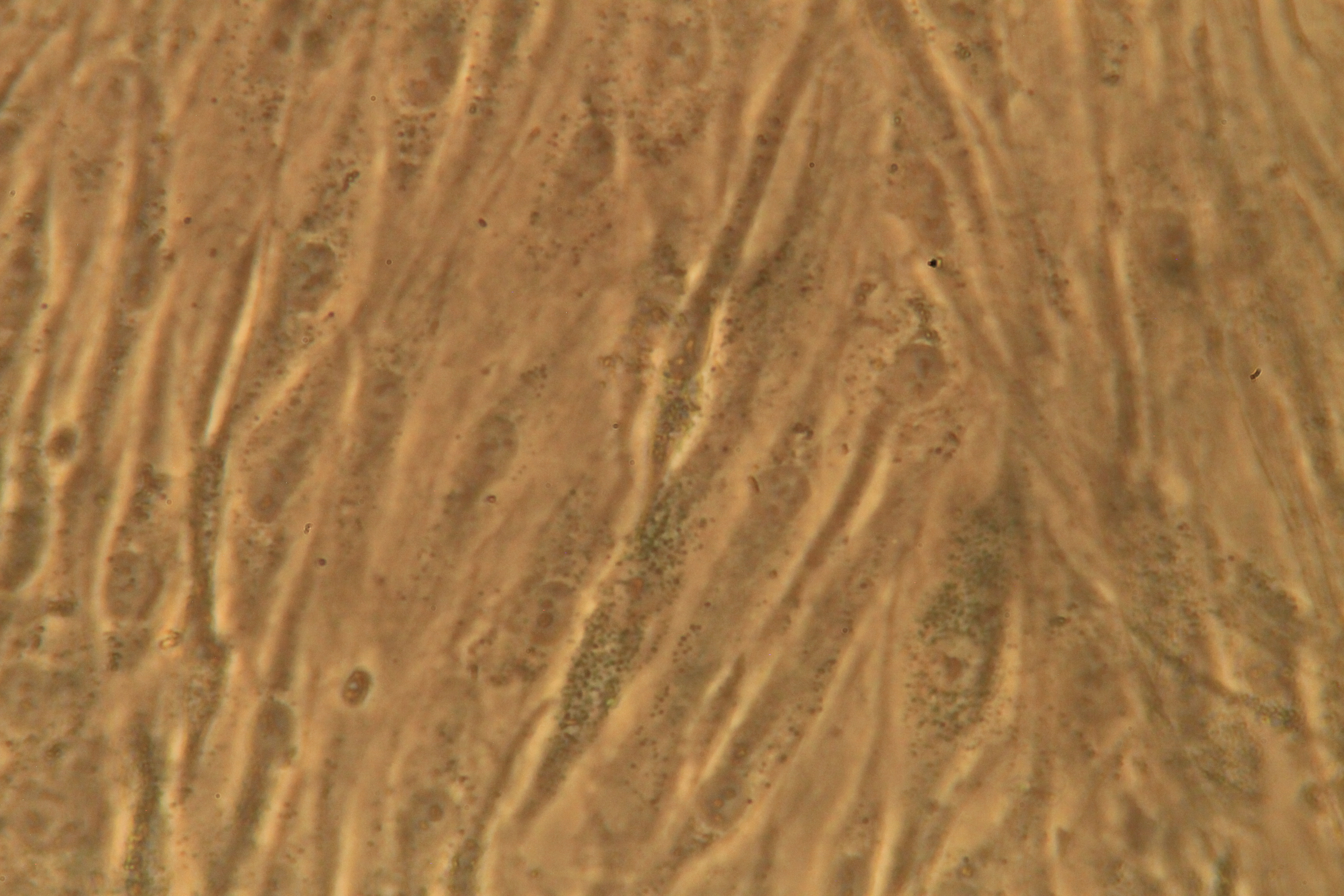

Supplement: Figure 5—source data 1. [file elife-62635-fig5-data1.zip › Figure 5-source data 1/beta galactosidase DRV/image 1.JPG]

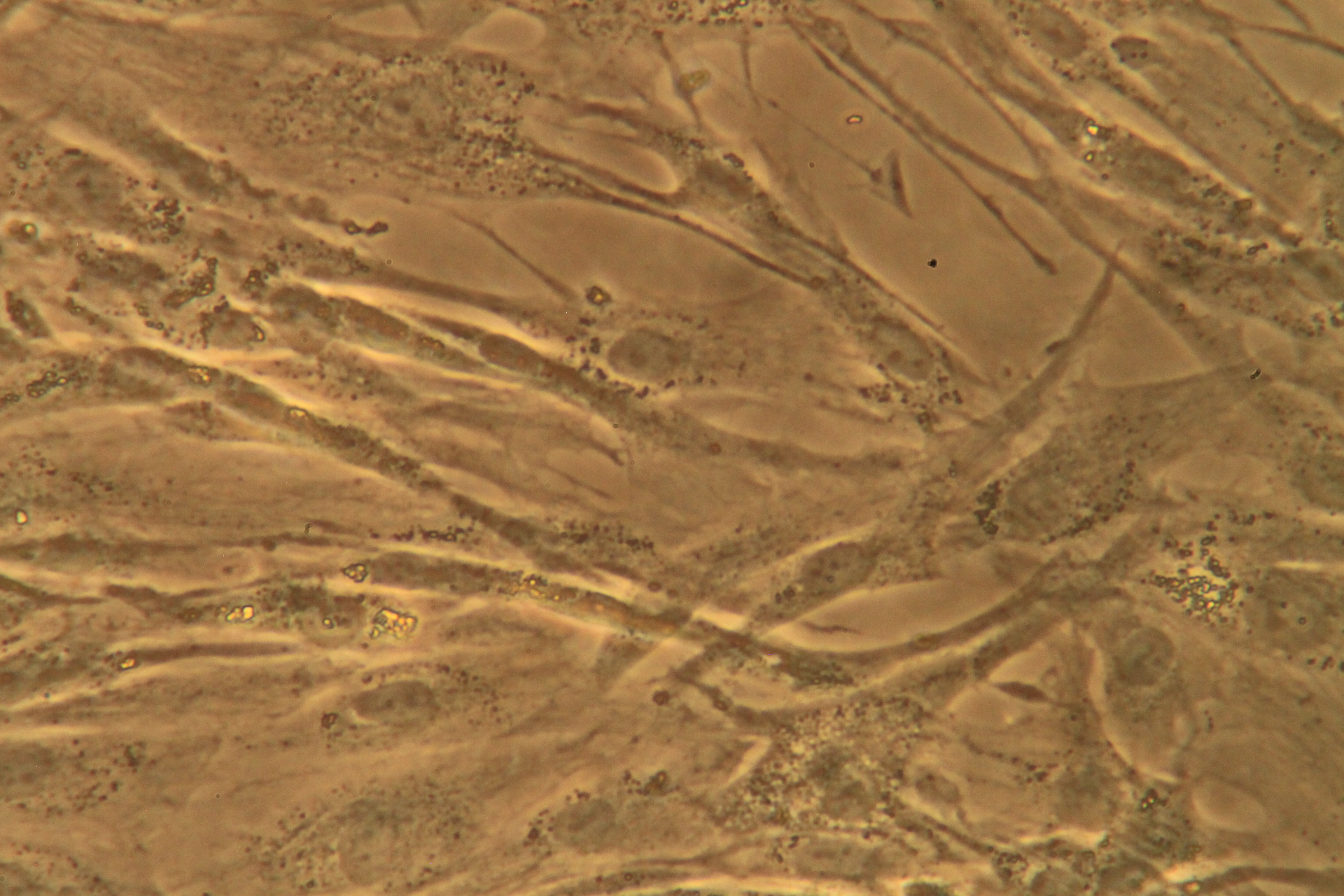

Supplement: Figure 5—source data 1. [file elife-62635-fig5-data1.zip › Figure 5-source data 1/beta galactosidase DRV/image 2.JPG]

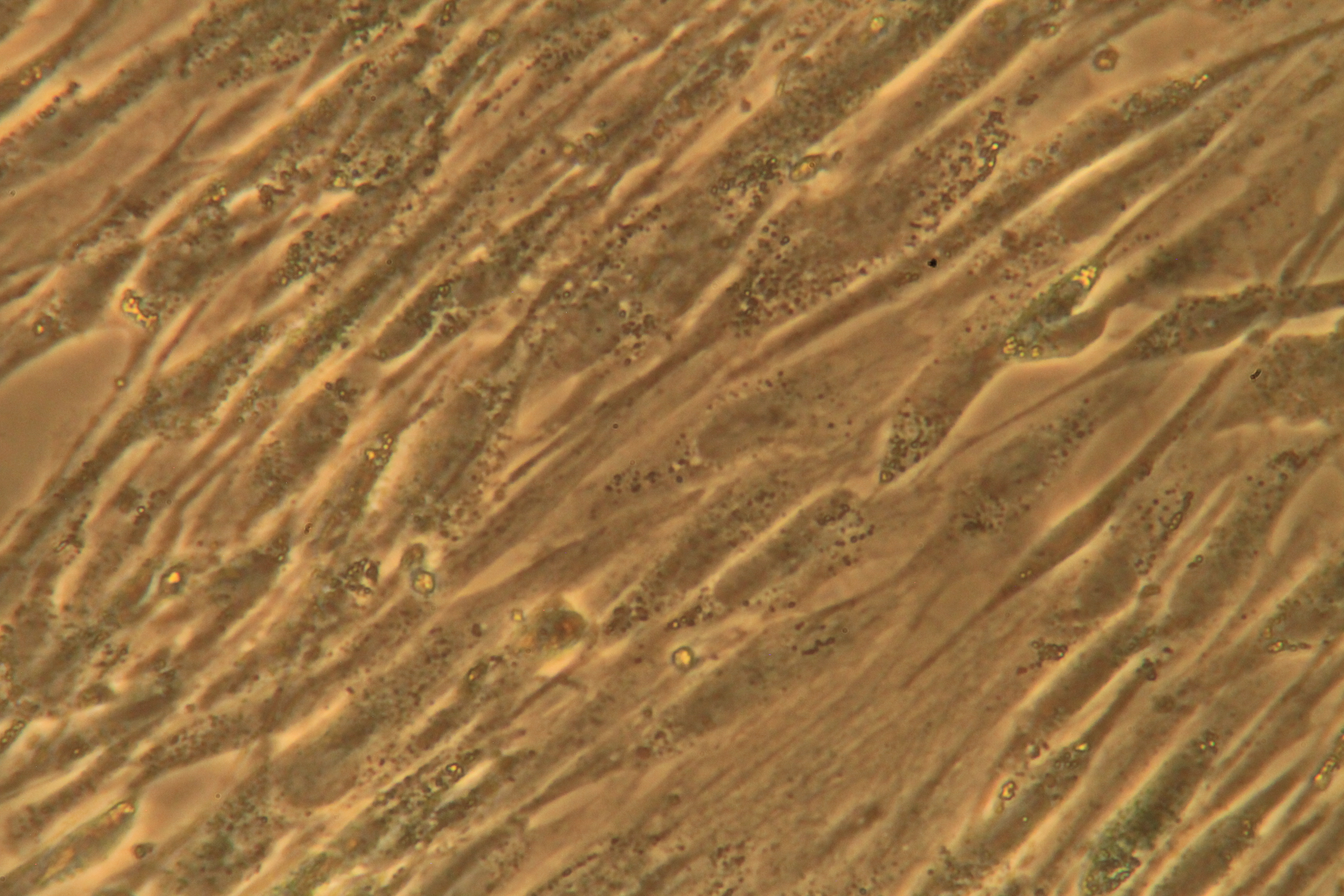

Supplement: Figure 5—source data 1. [file elife-62635-fig5-data1.zip › Figure 5-source data 1/beta galactosidase DRV/image 3.JPG]

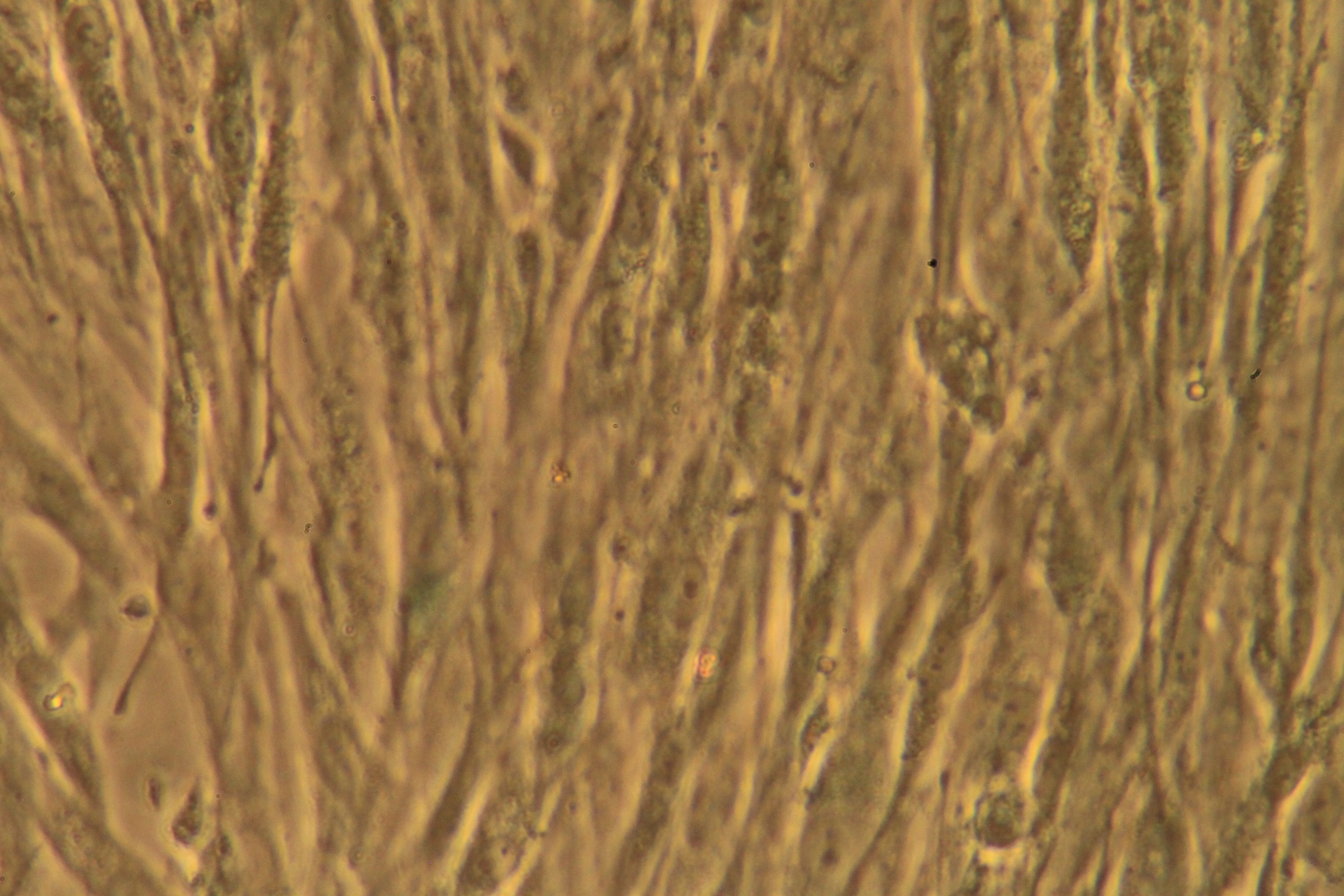

Supplement: Figure 5—source data 1. [file elife-62635-fig5-data1.zip › Figure 5-source data 1/beta galactosidase DMSO/image 1.JPG]

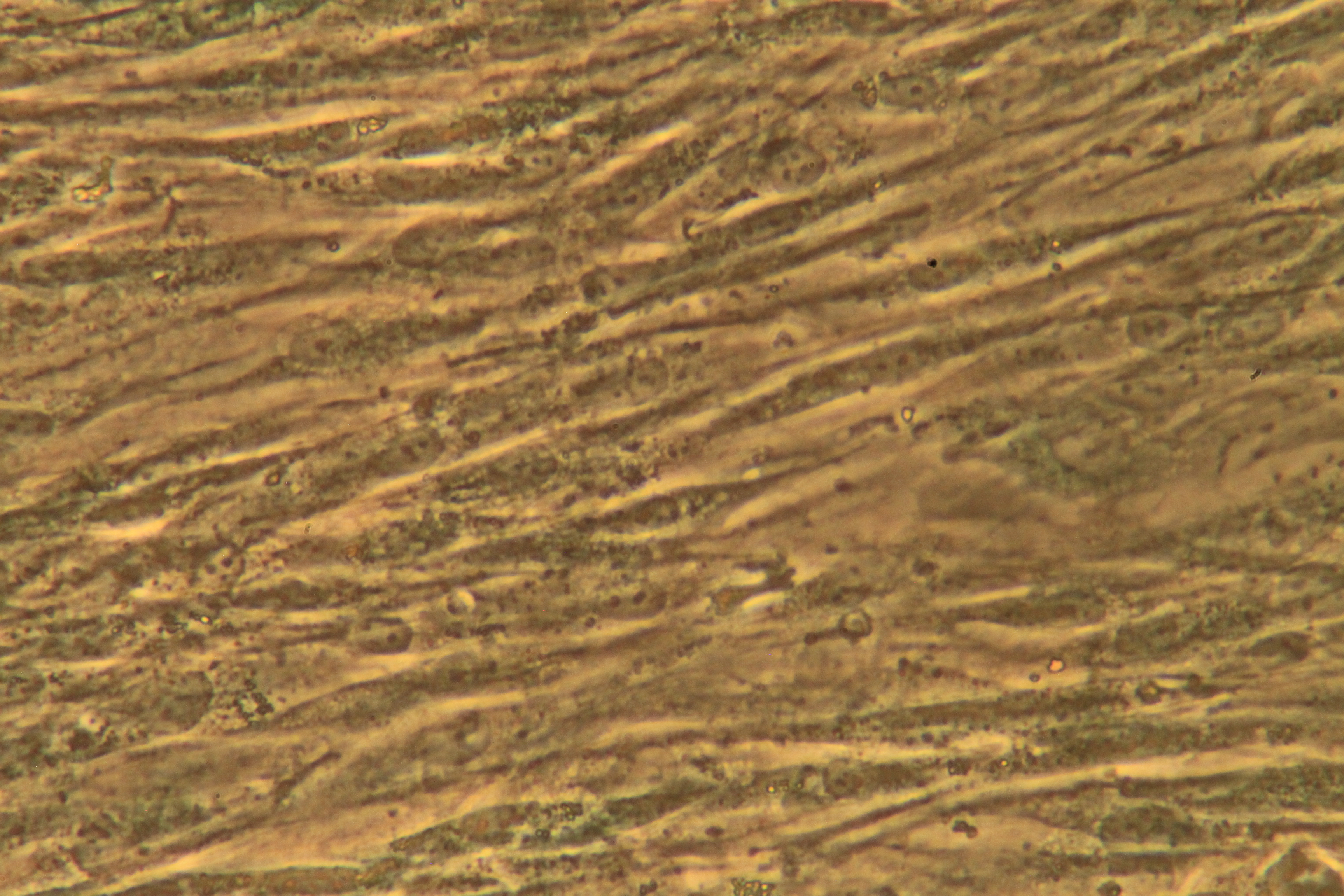

Supplement: Figure 5—source data 1. [file elife-62635-fig5-data1.zip › Figure 5-source data 1/beta galactosidase DMSO/image 2.JPG]

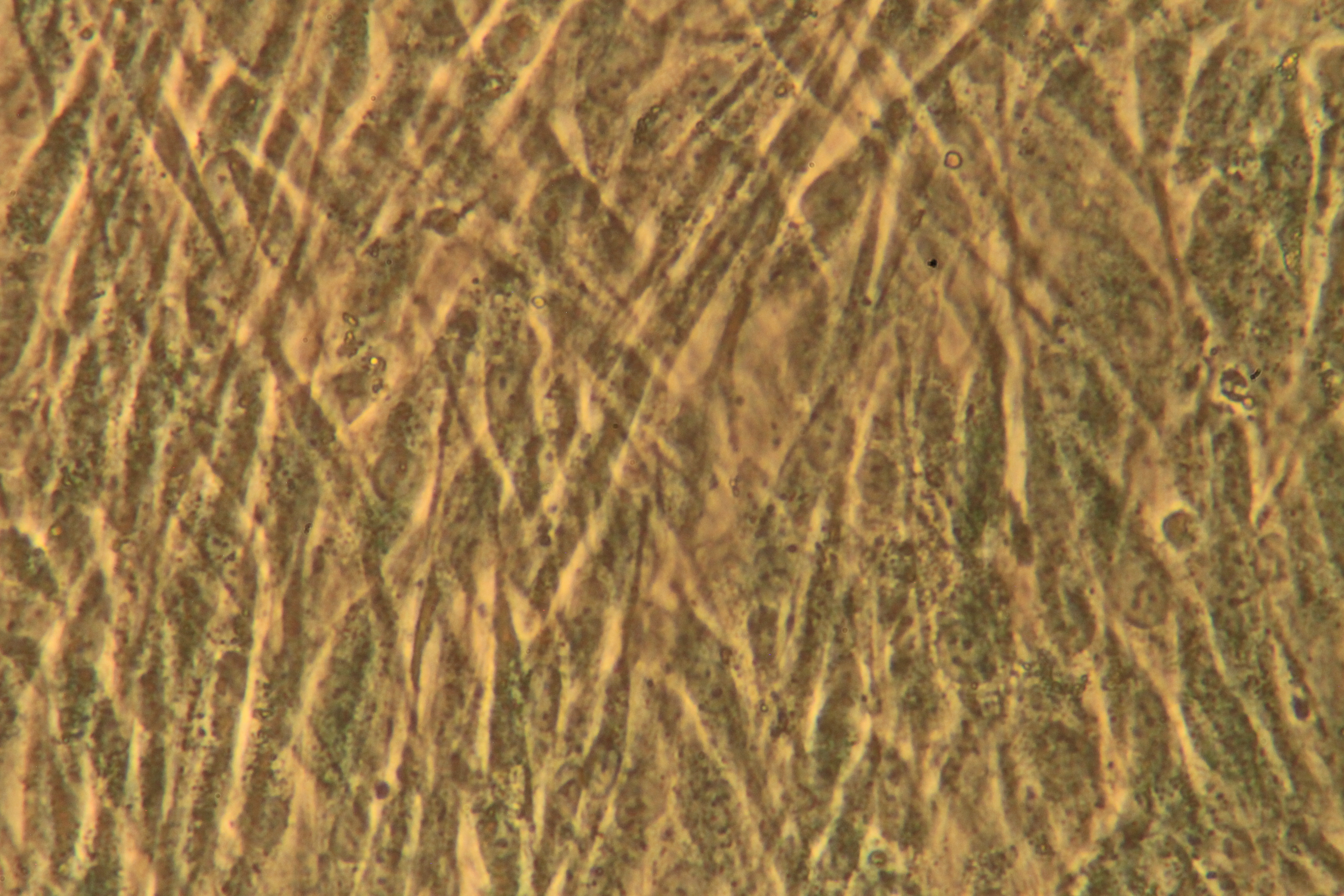

Supplement: Figure 5—source data 1. [file elife-62635-fig5-data1.zip › Figure 5-source data 1/beta galactosidase DMSO/image 3.JPG]

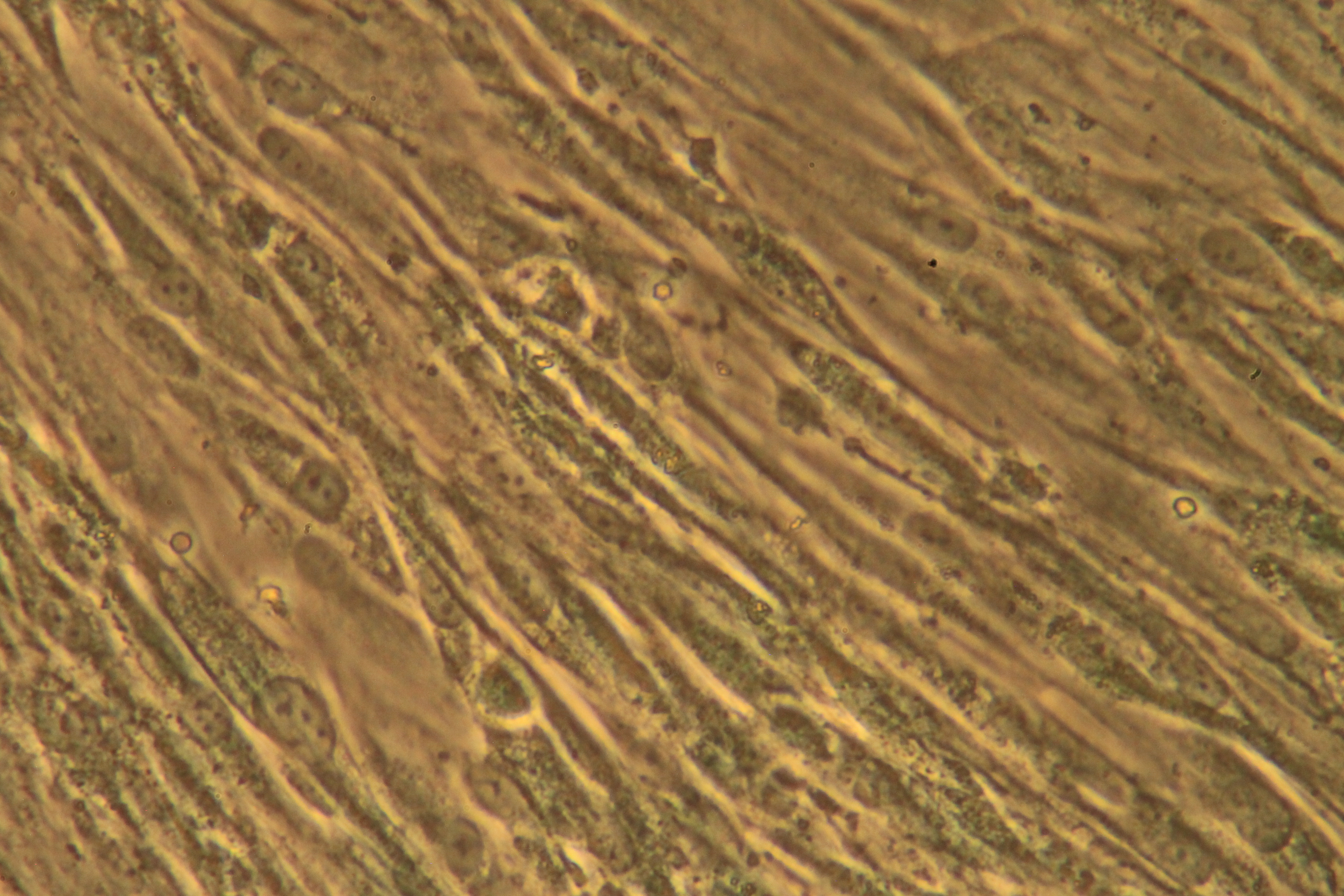

Supplement: Figure 5—source data 1. [file elife-62635-fig5-data1.zip › Figure 5-source data 1/beta galactosidase LPV/image 1.JPG]

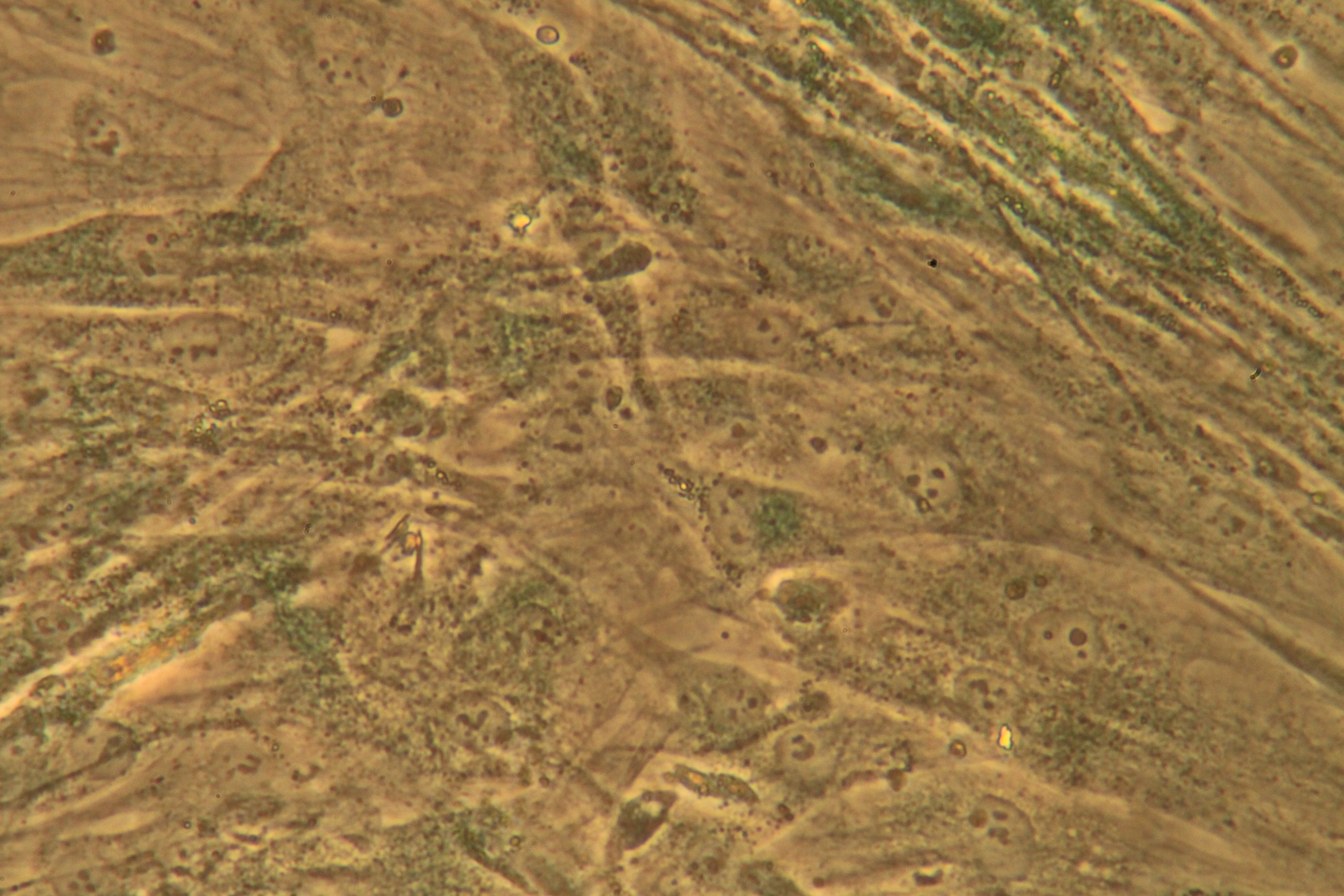

Supplement: Figure 5—source data 1. [file elife-62635-fig5-data1.zip › Figure 5-source data 1/beta galactosidase LPV/image 2.JPG]

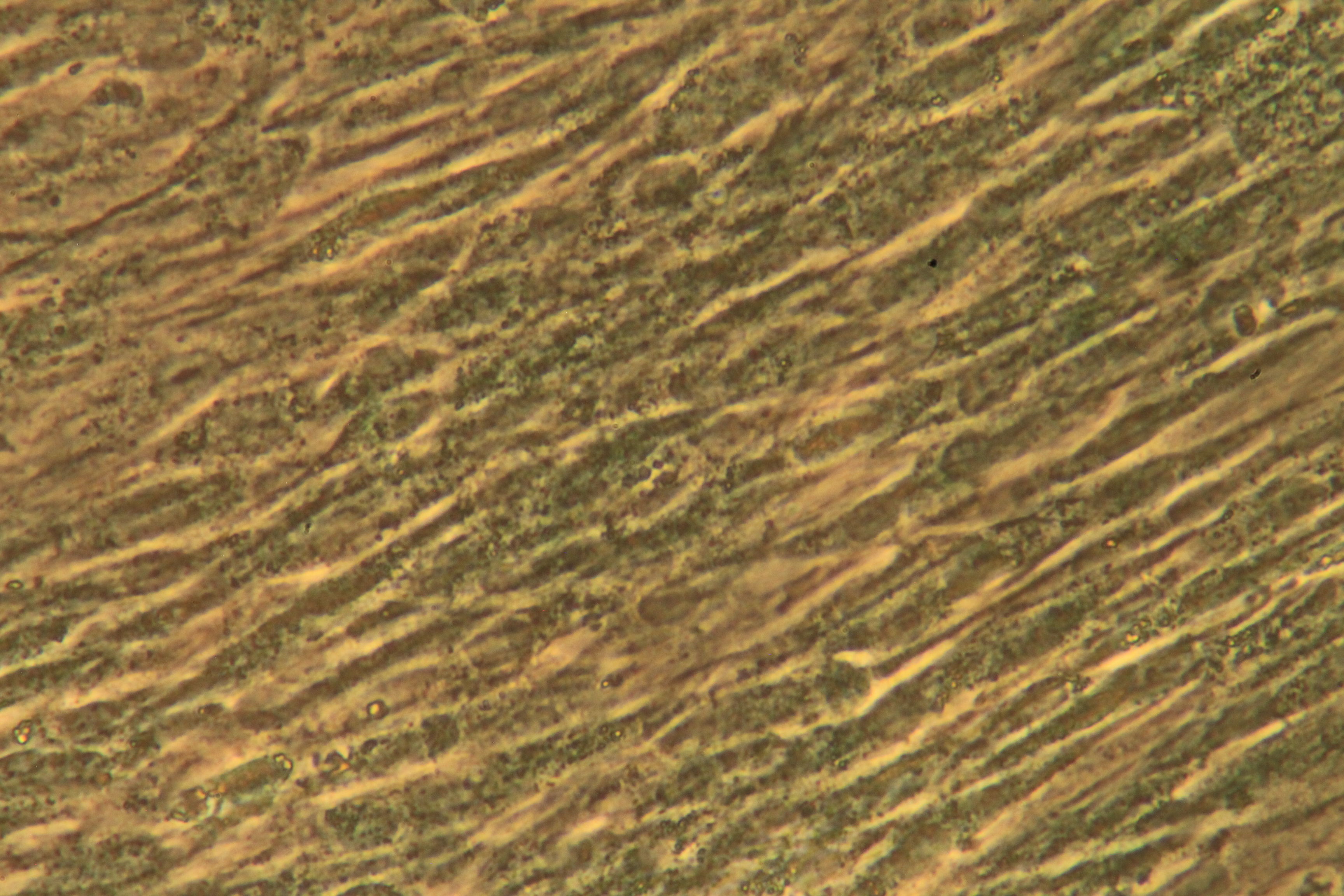

Supplement: Figure 5—source data 1. [file elife-62635-fig5-data1.zip › Figure 5-source data 1/beta galactosidase LPV/image 3.JPG]

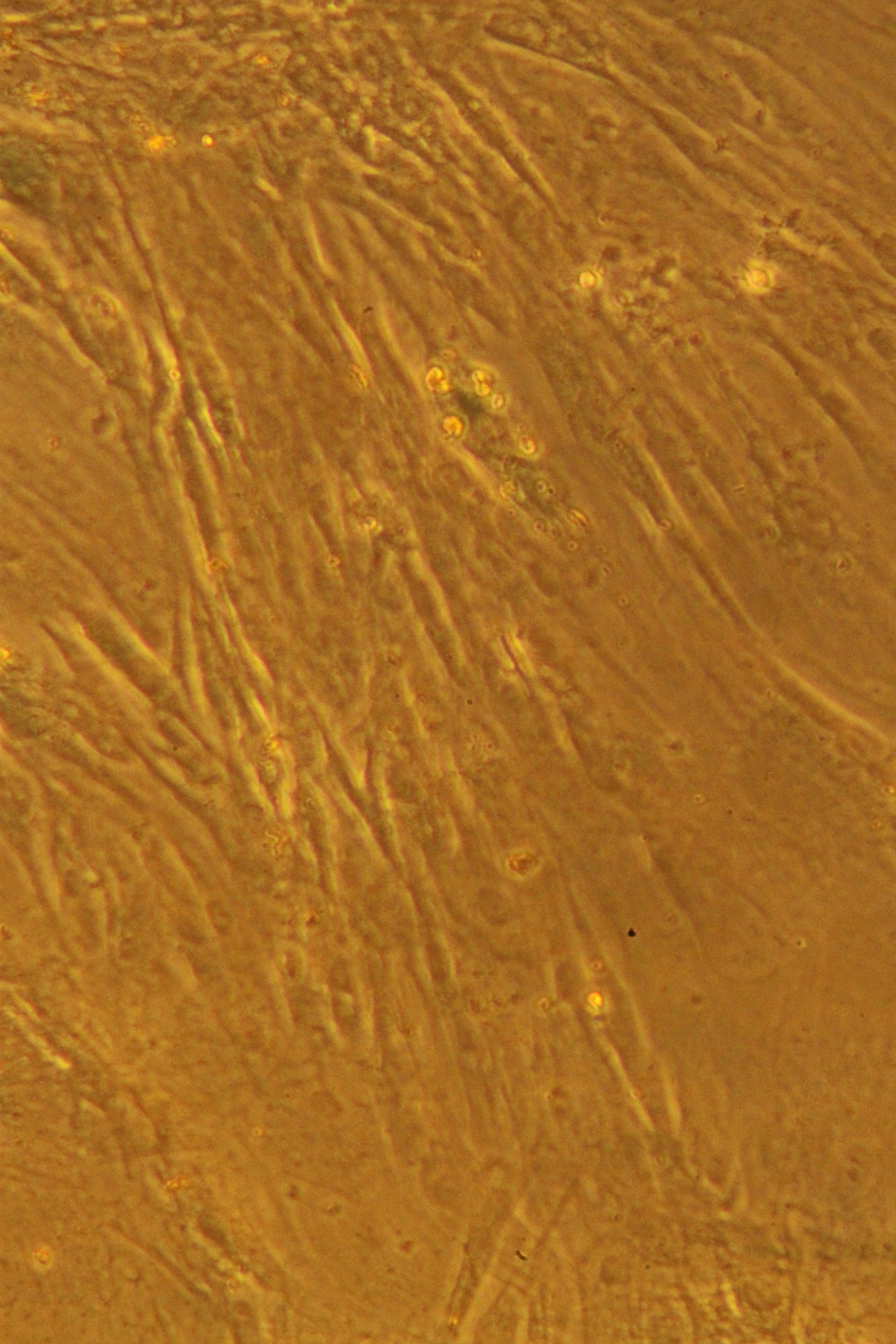

Supplement: Figure 6—source data 1. [file elife-62635-fig6-data1.zip › Figure 6-source data 1/beta galactosidase Aged Metformin/image 1 .jpg]

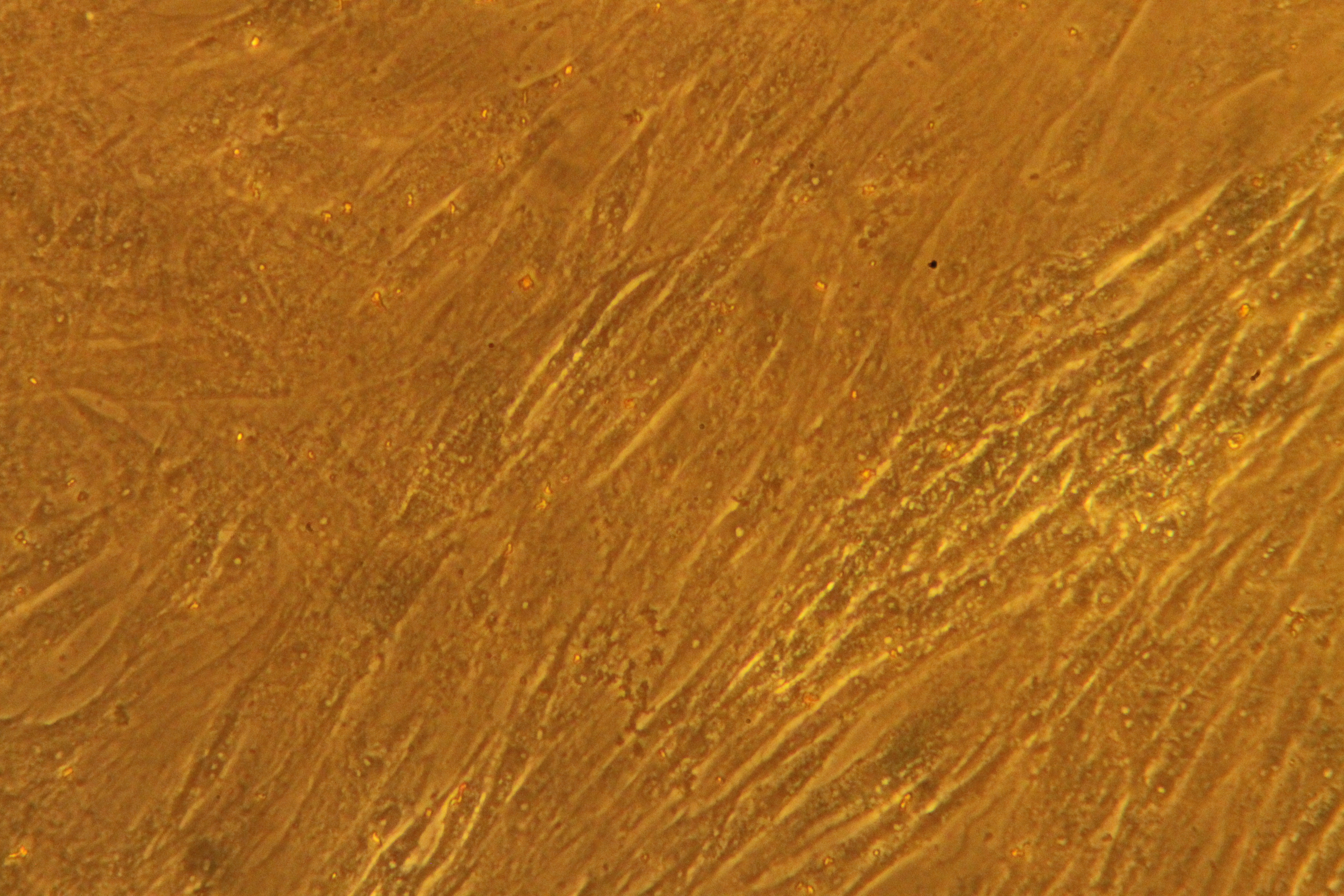

Supplement: Figure 6—source data 1. [file elife-62635-fig6-data1.zip › Figure 6-source data 1/beta galactosidase Aged Metformin/image 6.JPG]

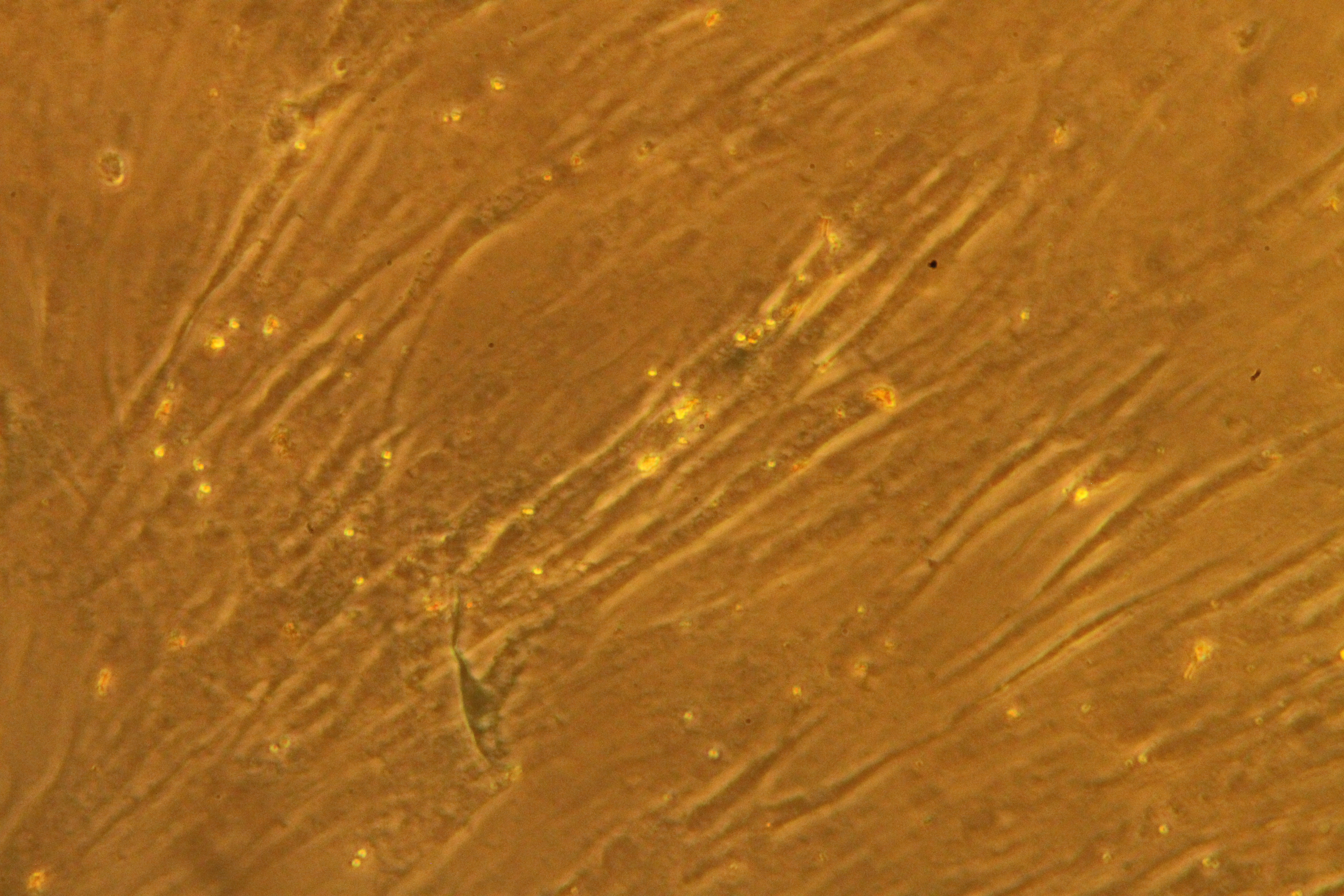

Supplement: Figure 6—source data 1. [file elife-62635-fig6-data1.zip › Figure 6-source data 1/beta galactosidase Aged Metformin/image 4.JPG]

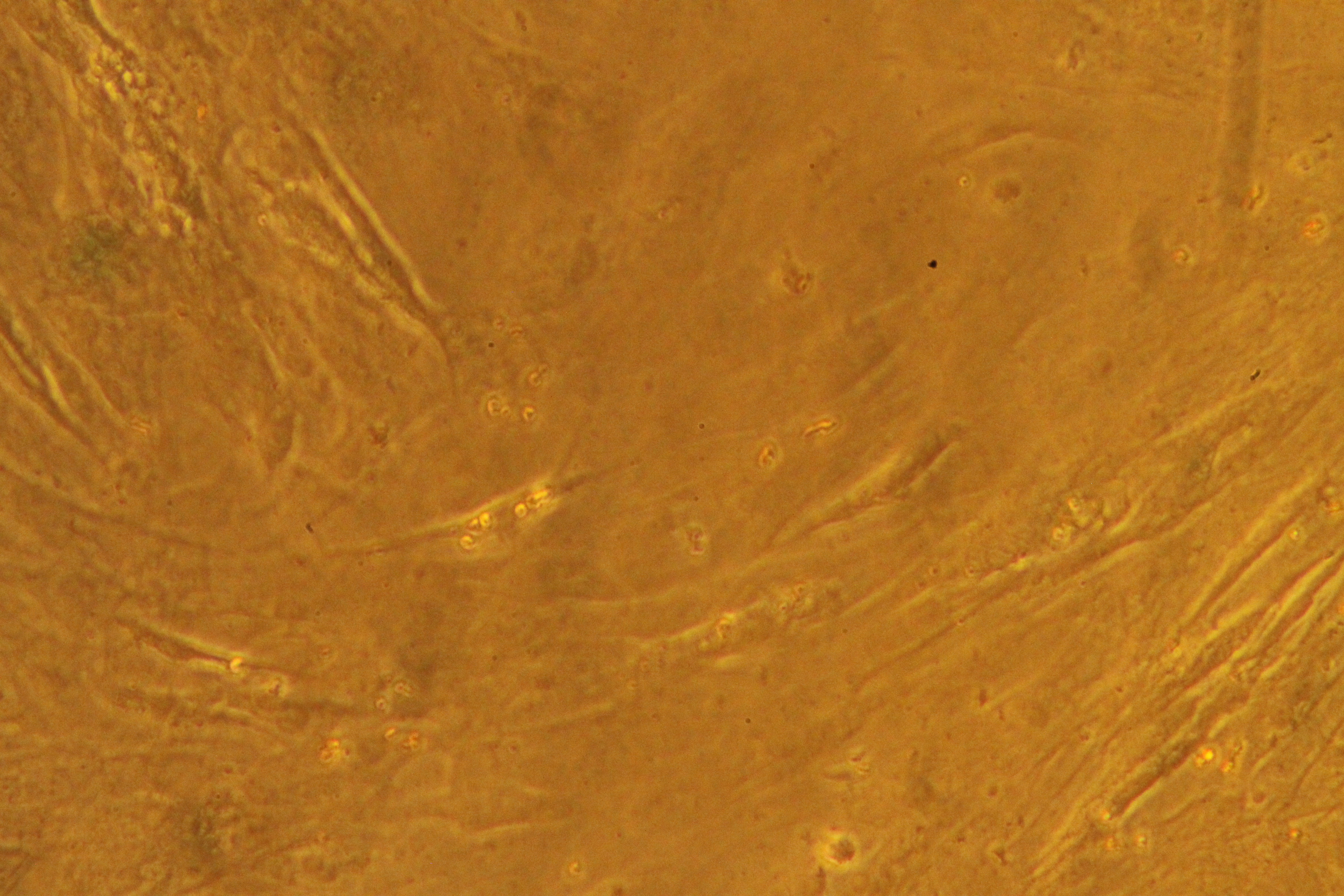

Supplement: Figure 6—source data 1. [file elife-62635-fig6-data1.zip › Figure 6-source data 1/beta galactosidase Aged Metformin/image 5.JPG]

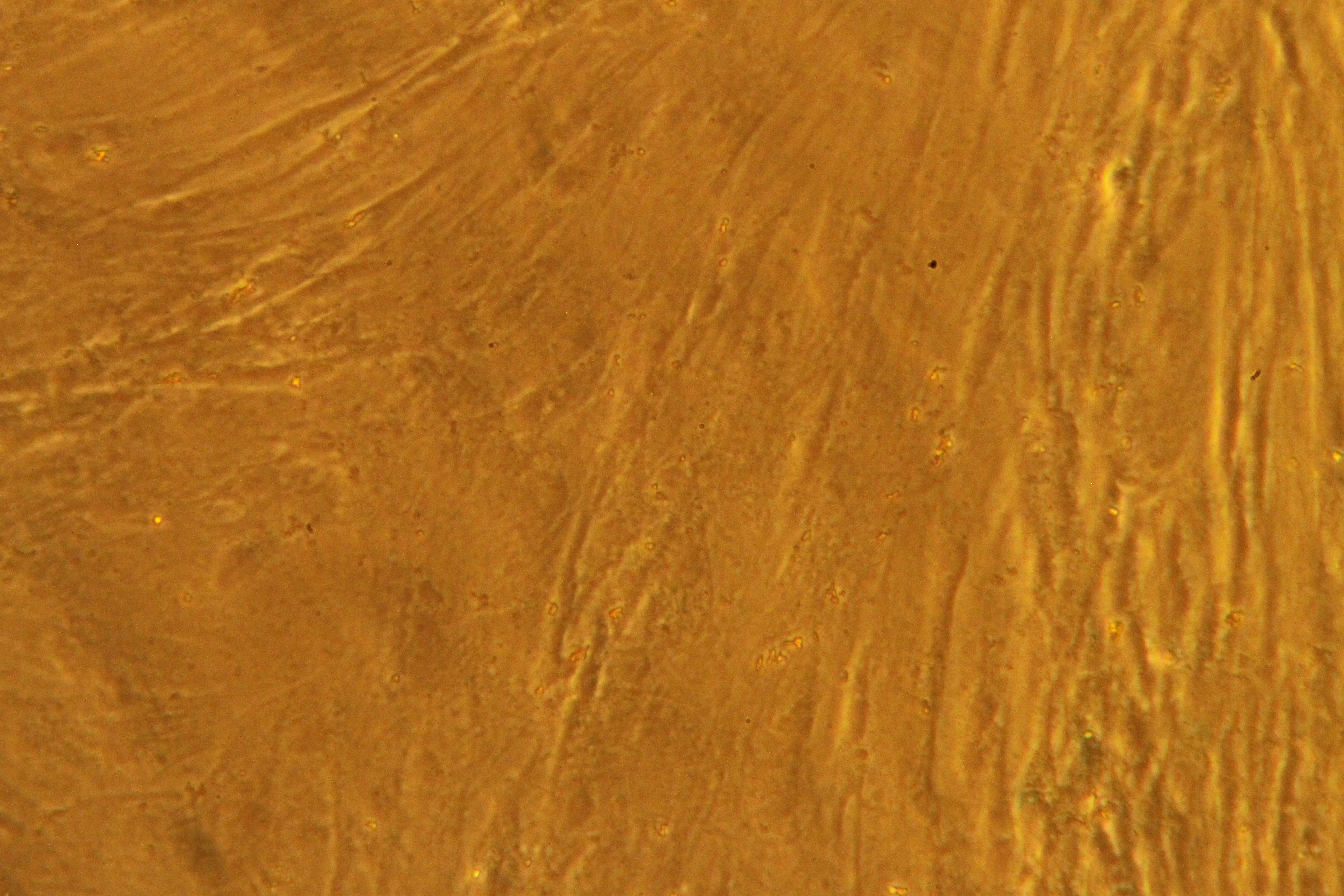

Supplement: Figure 6—source data 1. [file elife-62635-fig6-data1.zip › Figure 6-source data 1/beta galactosidase Aged Metformin/image 2.JPG]

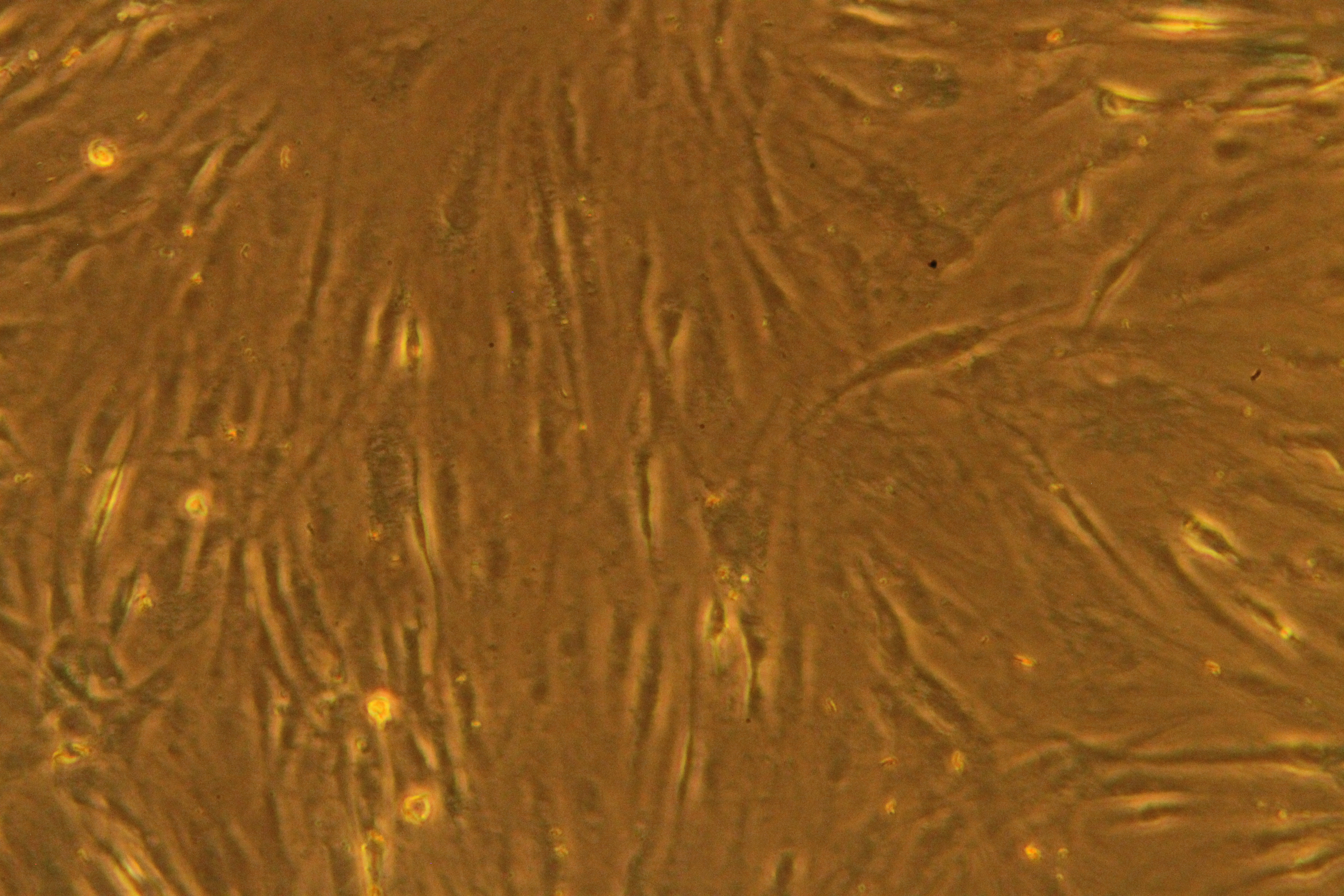

Supplement: Figure 6—source data 1. [file elife-62635-fig6-data1.zip › Figure 6-source data 1/beta galactosidase Aged Metformin/image 3.JPG]

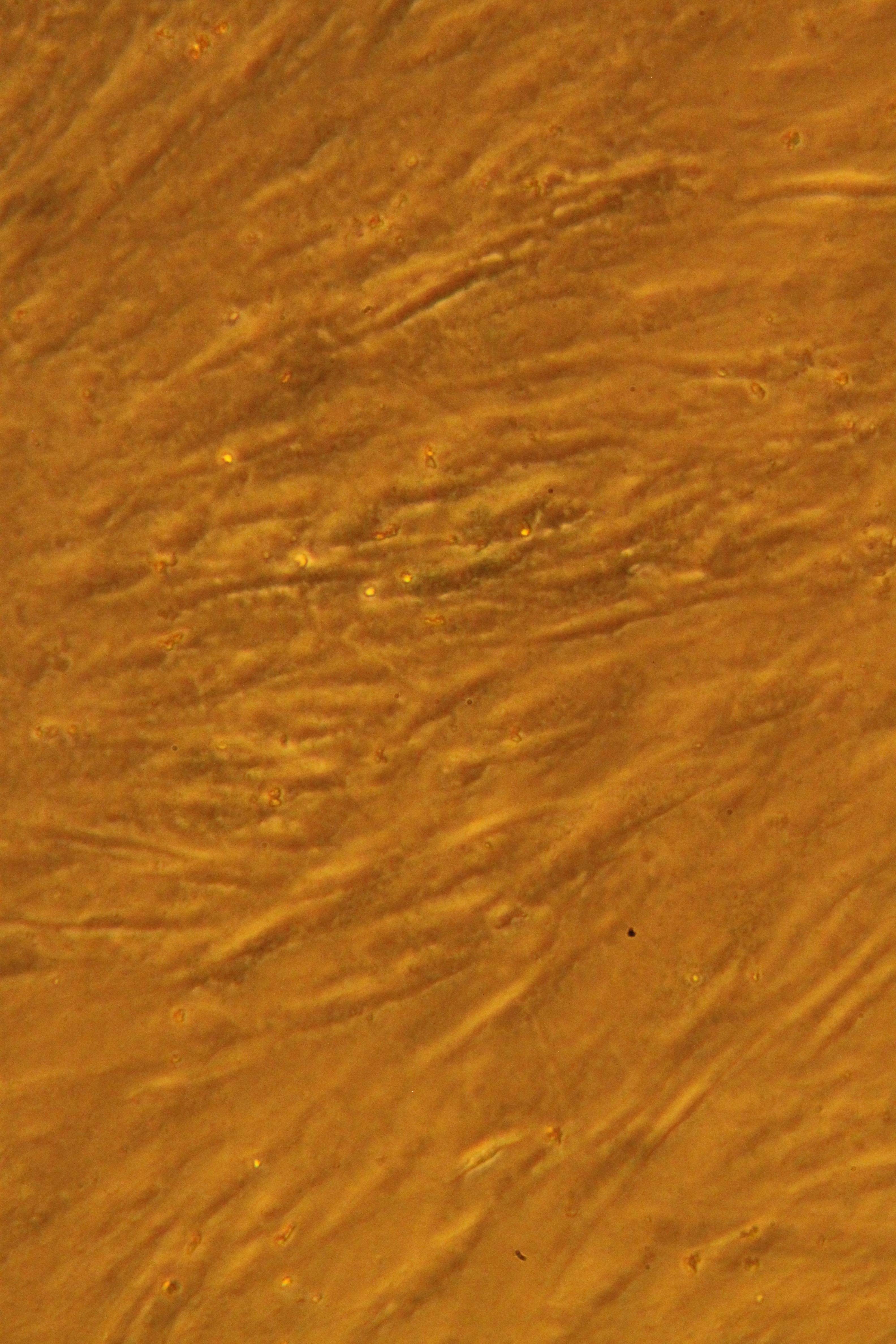

Supplement: Figure 6—source data 1. [file elife-62635-fig6-data1.zip › Figure 6-source data 1/beta galactosidase Young Metformin/image 1 .jpg]

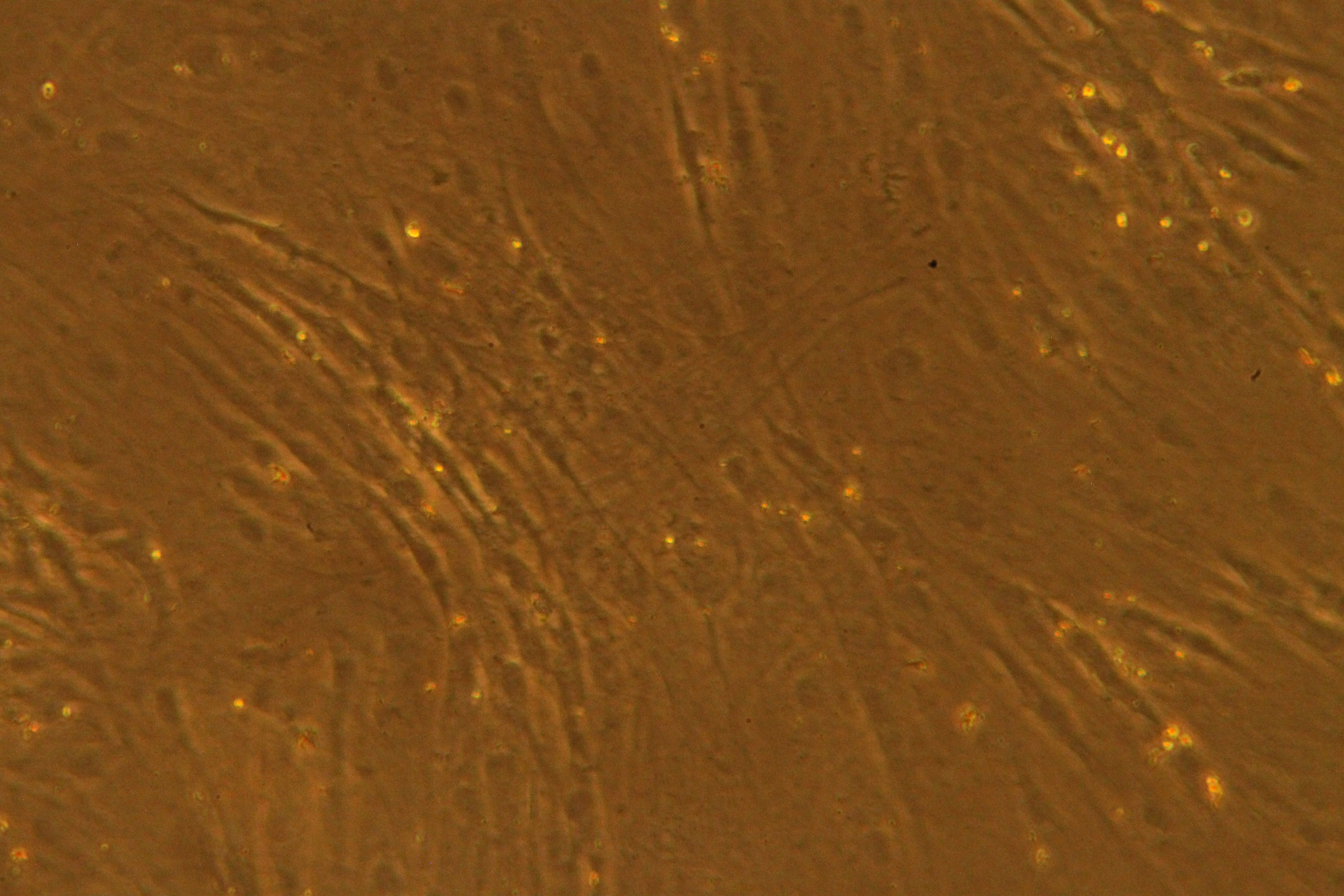

Supplement: Figure 6—source data 1. [file elife-62635-fig6-data1.zip › Figure 6-source data 1/beta galactosidase Young Metformin/image 6.JPG]

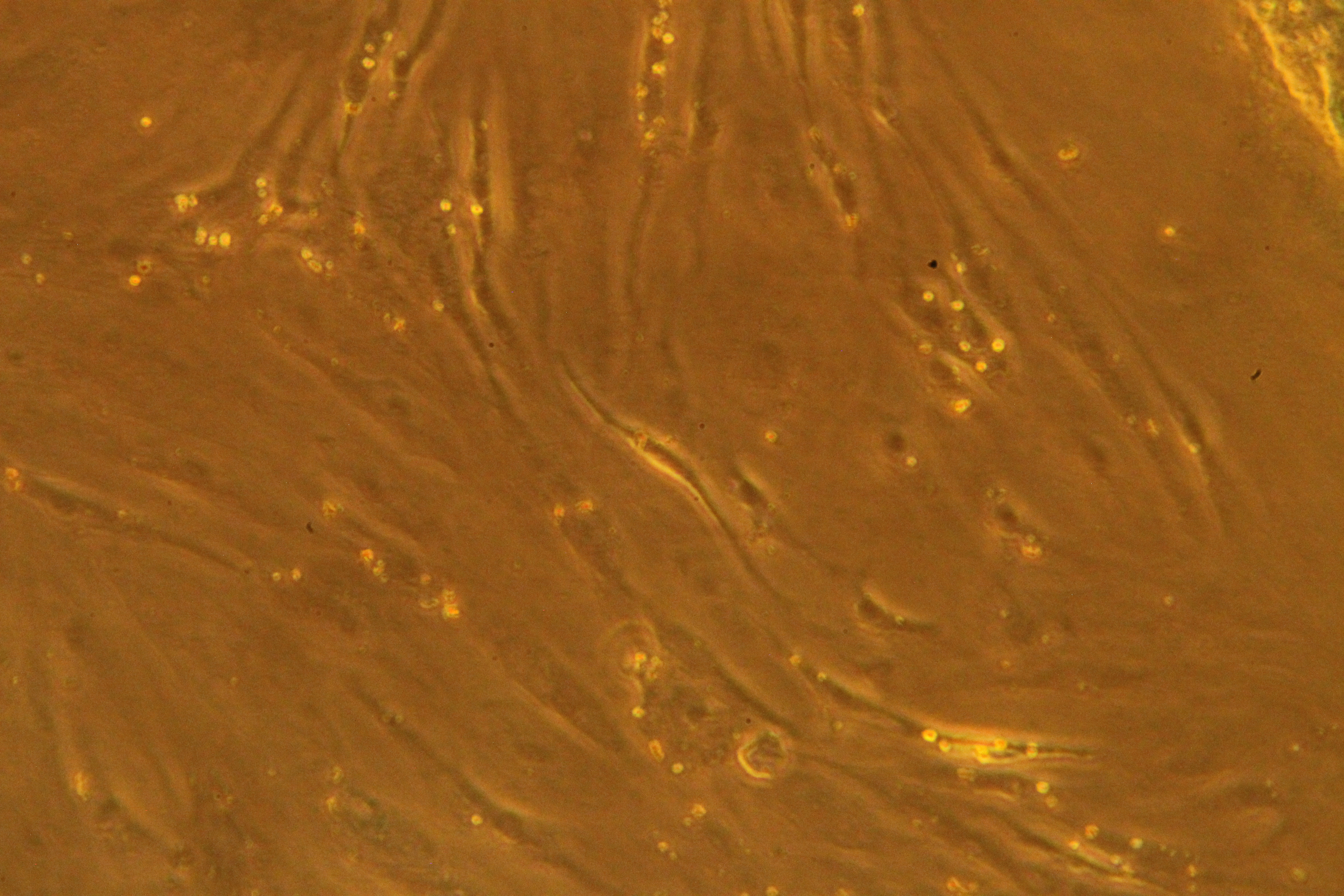

Supplement: Figure 6—source data 1. [file elife-62635-fig6-data1.zip › Figure 6-source data 1/beta galactosidase Young Metformin/image 4.JPG]

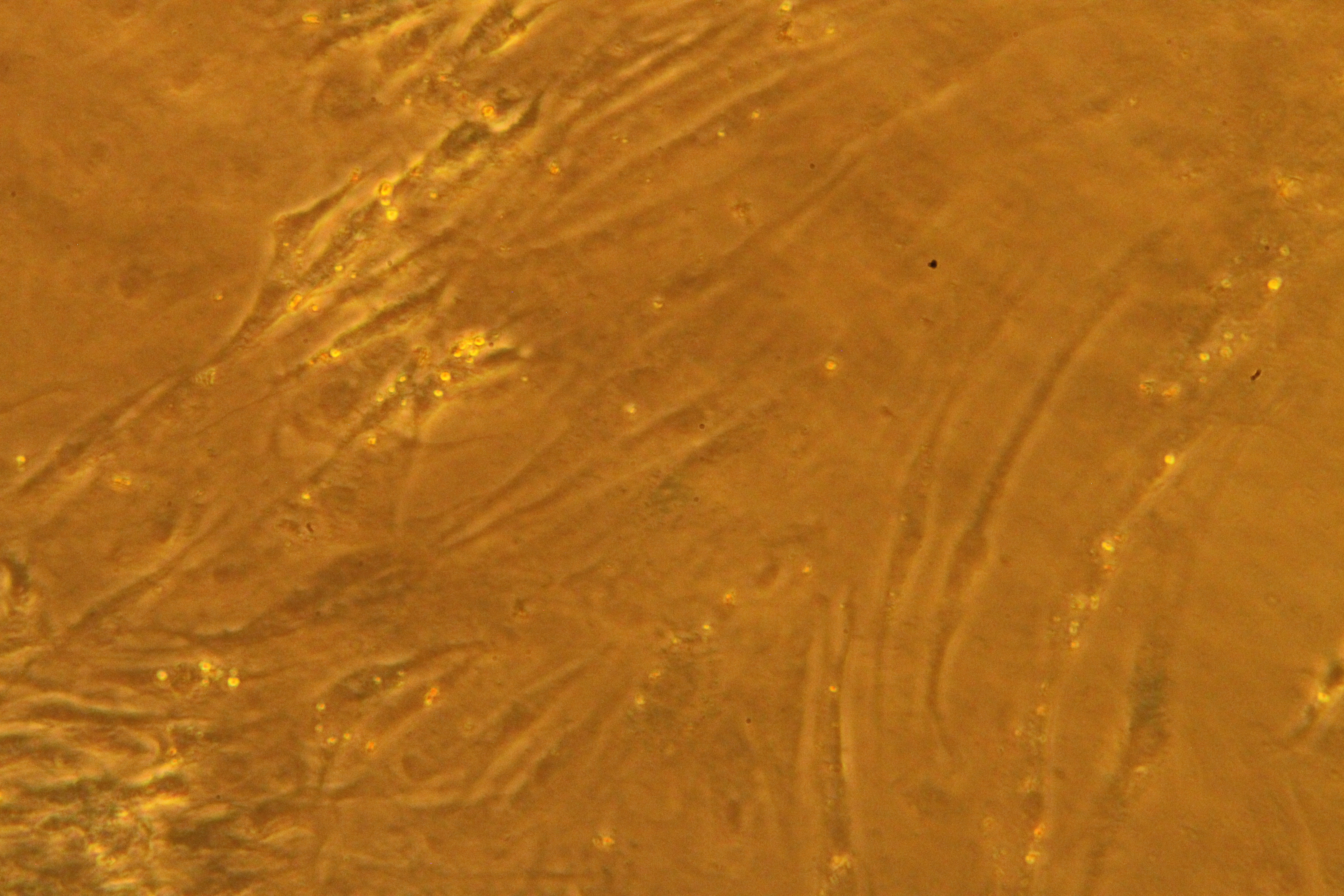

Supplement: Figure 6—source data 1. [file elife-62635-fig6-data1.zip › Figure 6-source data 1/beta galactosidase Young Metformin/image 5.JPG]

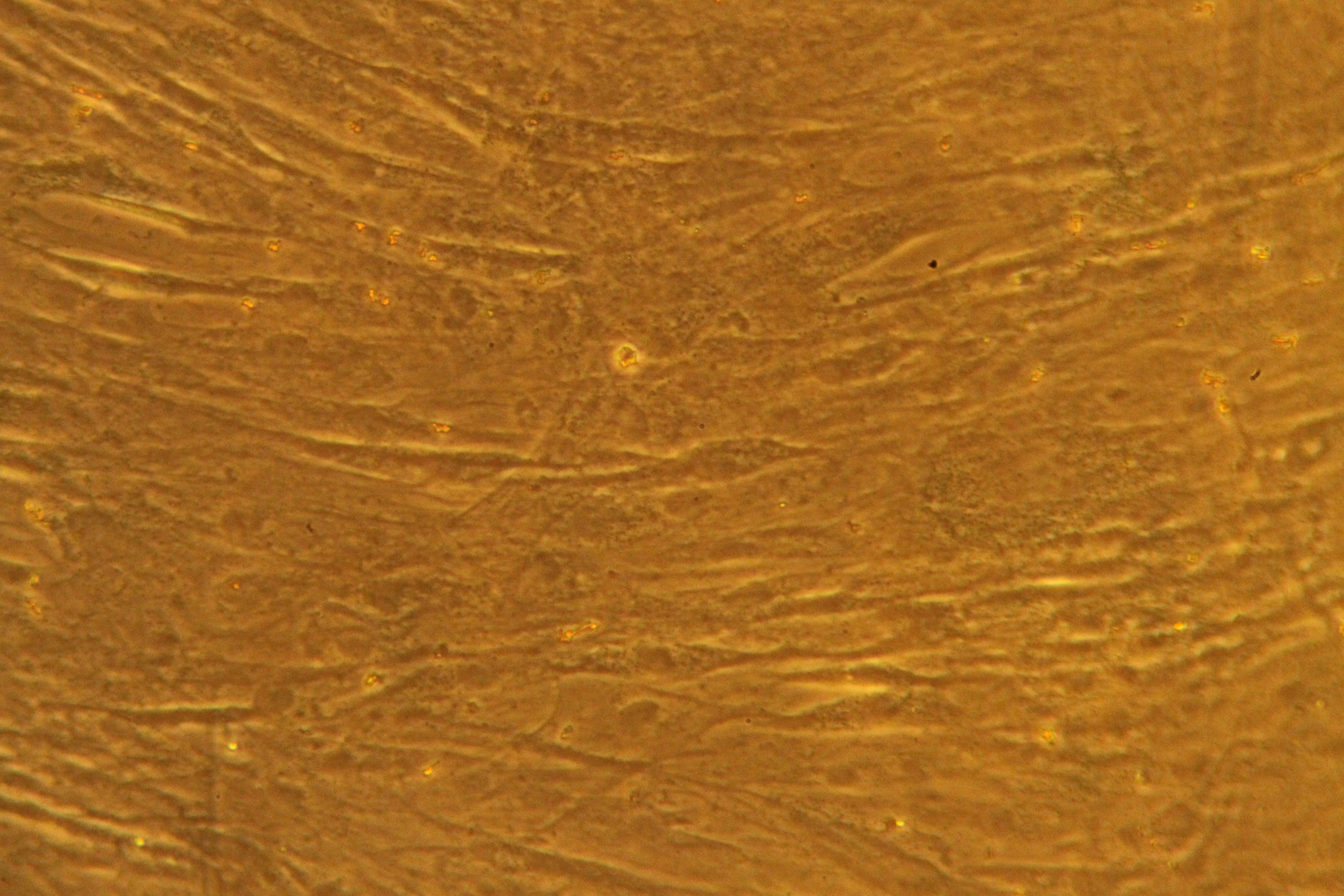

Supplement: Figure 6—source data 1. [file elife-62635-fig6-data1.zip › Figure 6-source data 1/beta galactosidase Young Metformin/image 2.JPG]

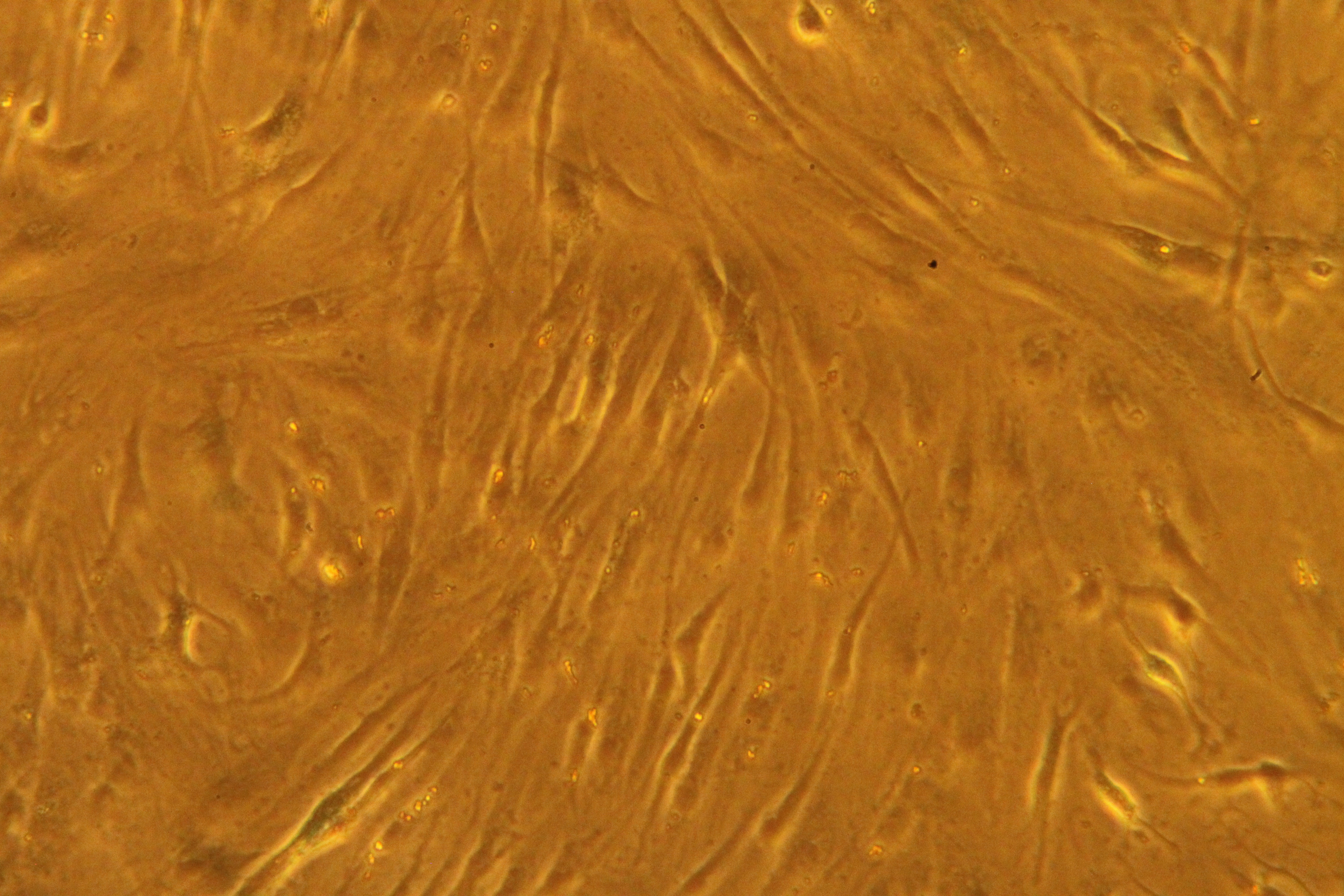

Supplement: Figure 6—source data 1. [file elife-62635-fig6-data1.zip › Figure 6-source data 1/beta galactosidase Young Metformin/image 3.JPG]

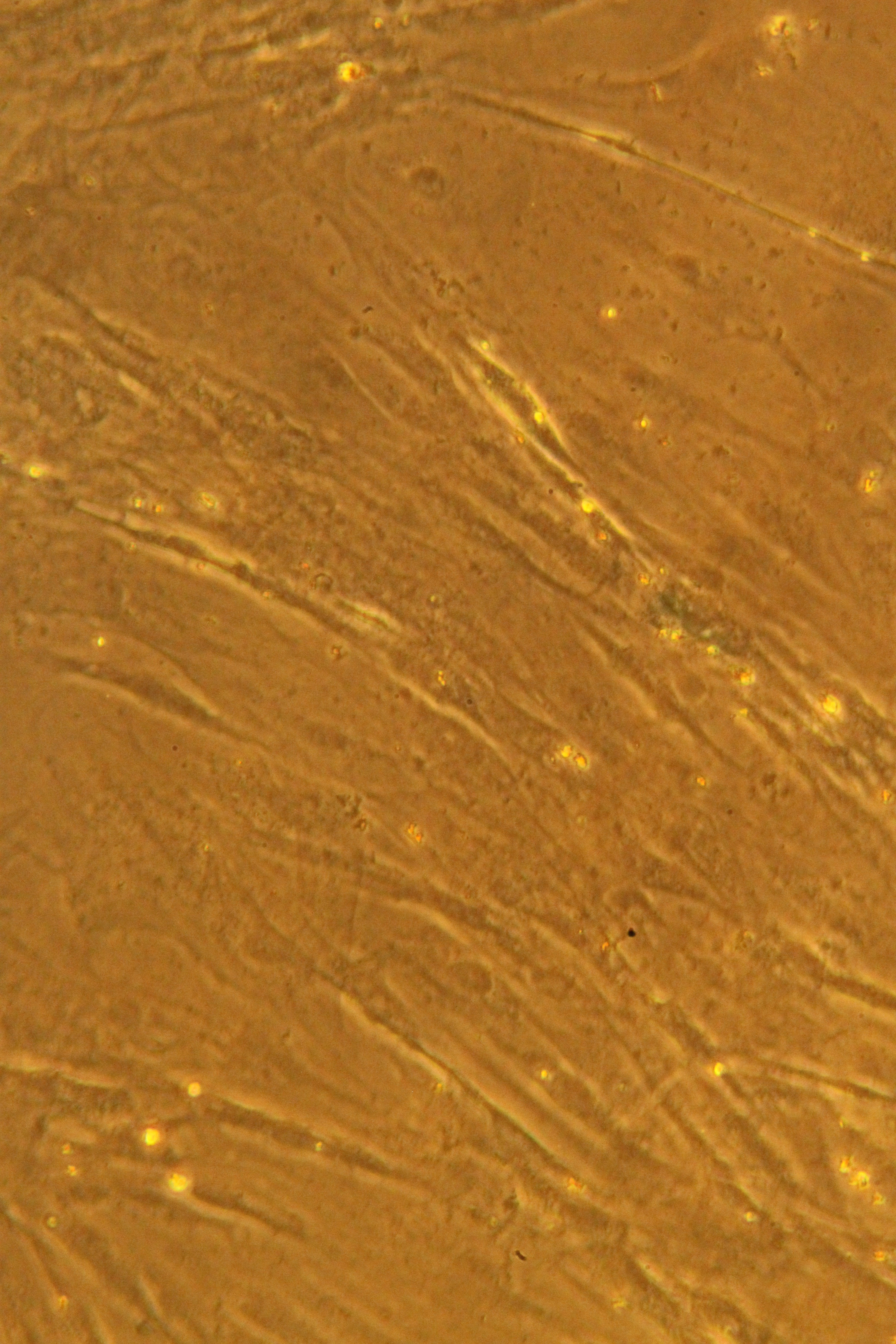

Supplement: Figure 6—source data 2. [file elife-62635-fig6-data2.zip › Figure 6-source data 2/beta galactosidase Young untreated/image 1 .jpg]

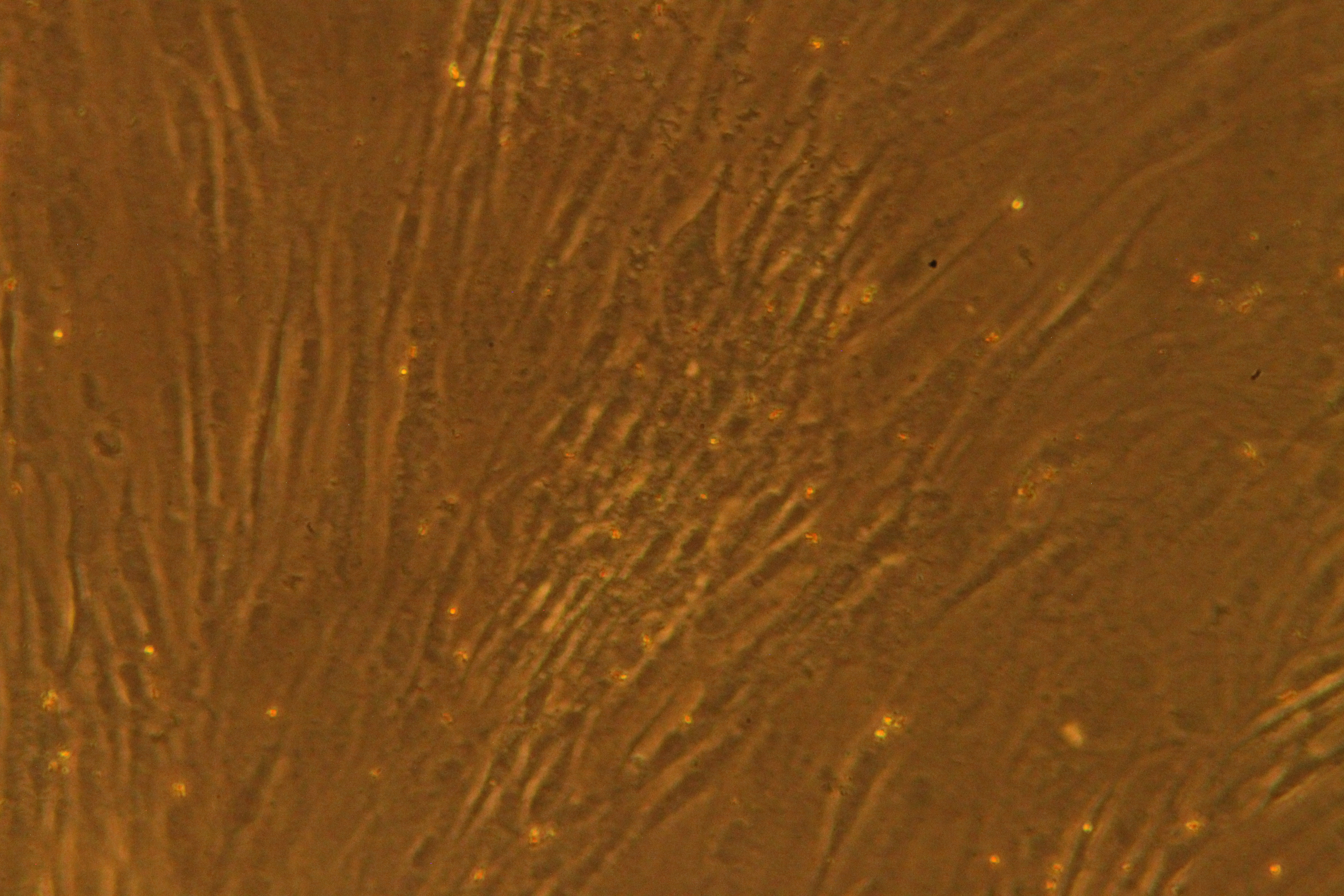

Supplement: Figure 6—source data 2. [file elife-62635-fig6-data2.zip › Figure 6-source data 2/beta galactosidase Young untreated/image 6.JPG]

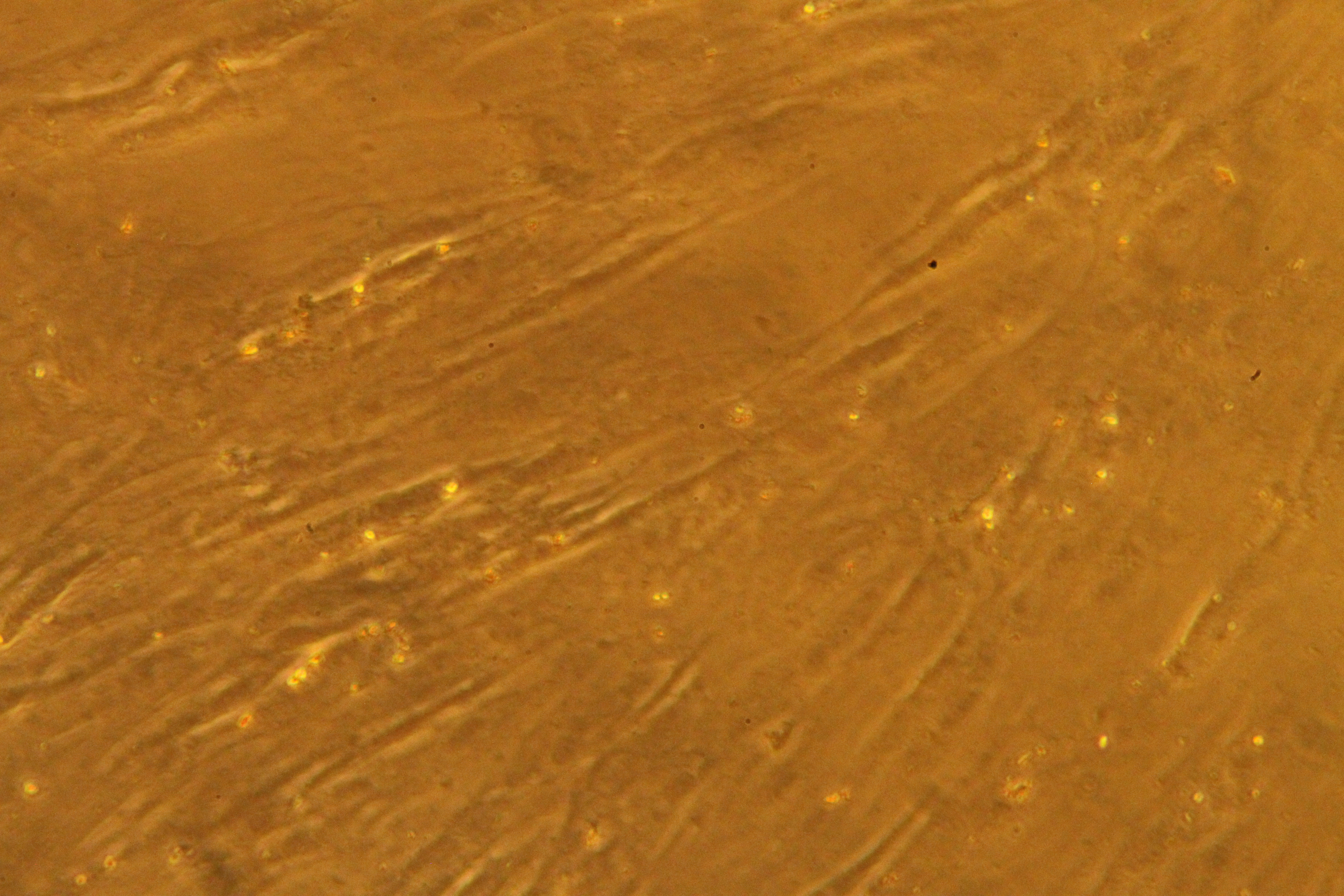

Supplement: Figure 6—source data 2. [file elife-62635-fig6-data2.zip › Figure 6-source data 2/beta galactosidase Young untreated/image 4.JPG]

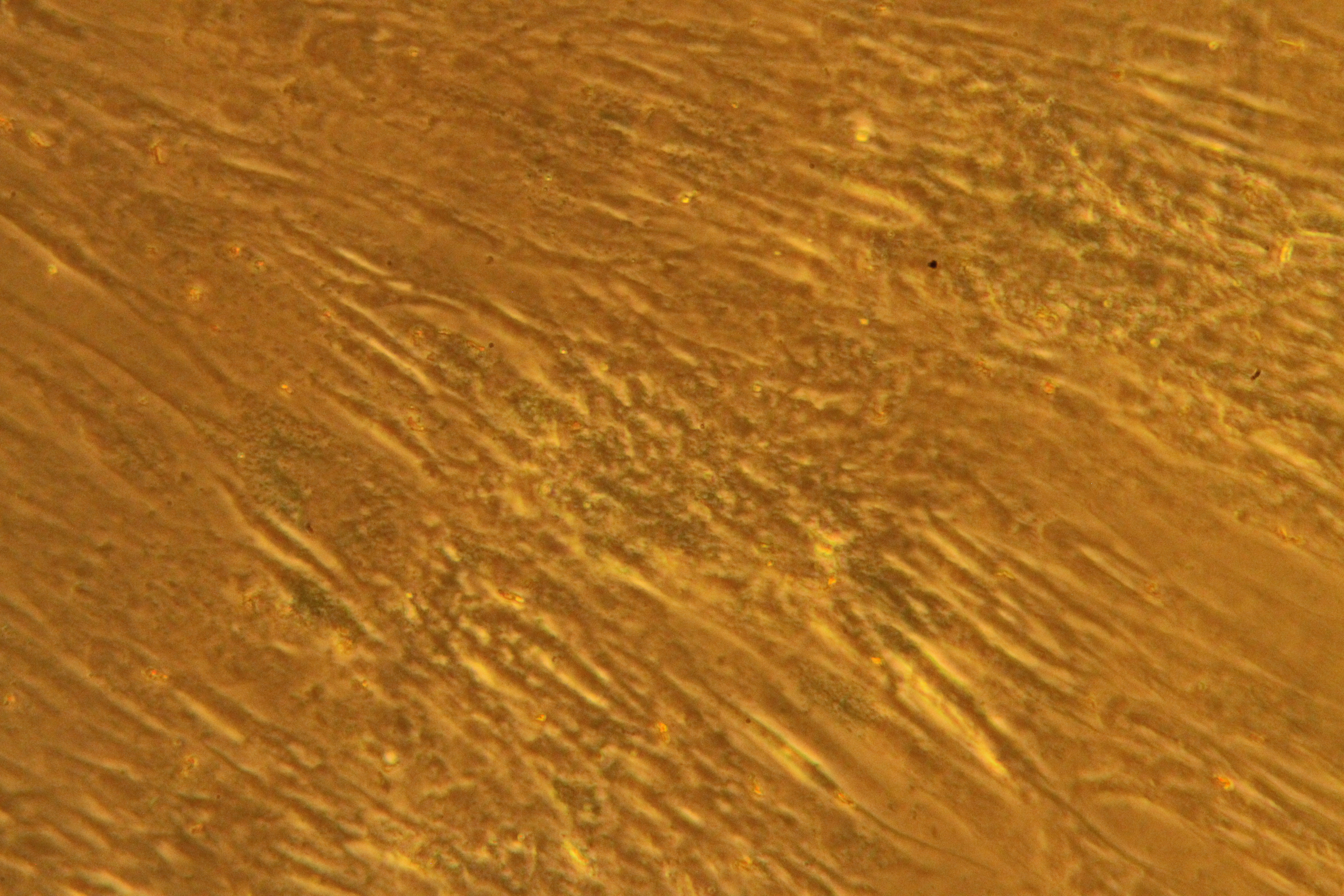

Supplement: Figure 6—source data 2. [file elife-62635-fig6-data2.zip › Figure 6-source data 2/beta galactosidase Young untreated/image 5.JPG]

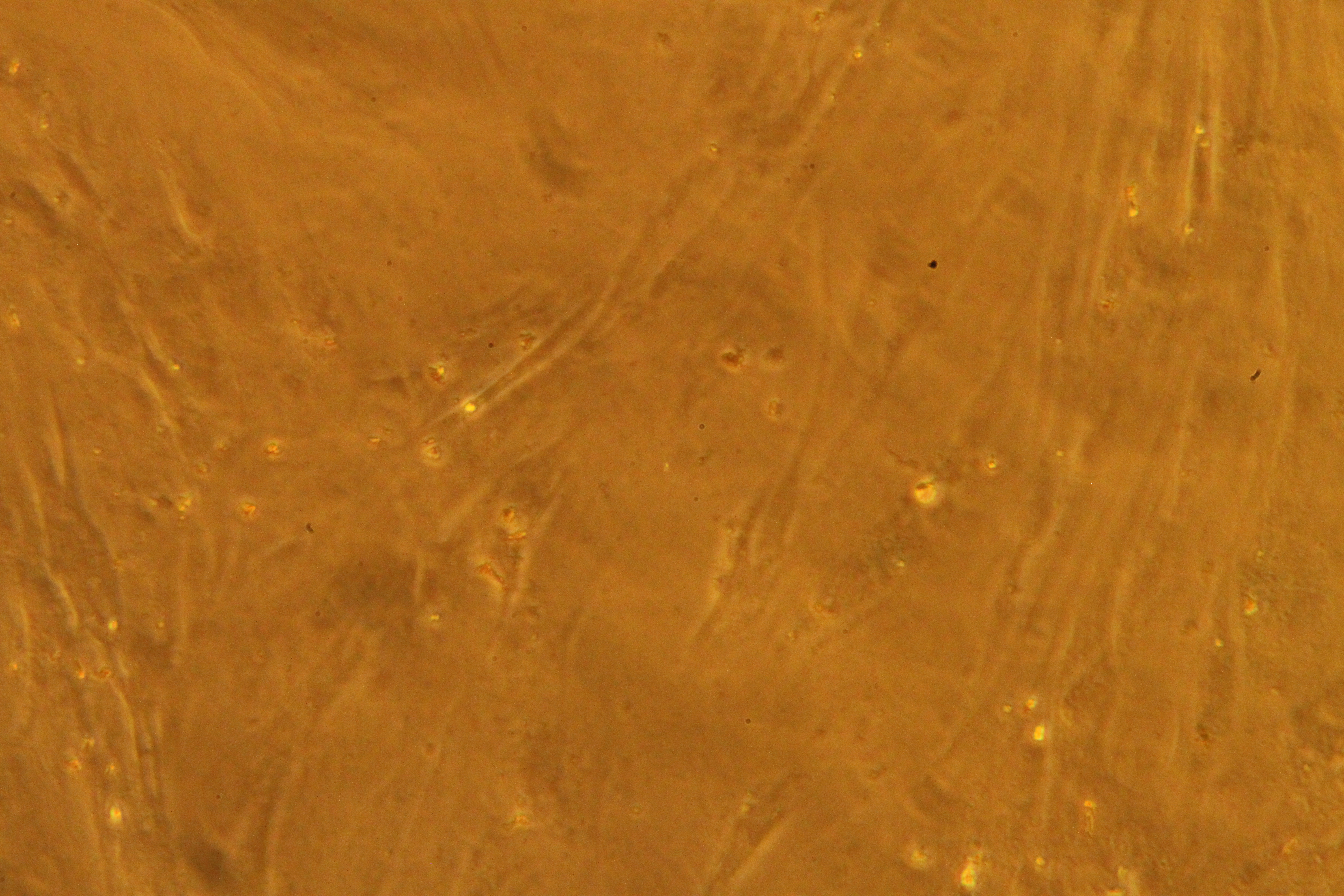

Supplement: Figure 6—source data 2. [file elife-62635-fig6-data2.zip › Figure 6-source data 2/beta galactosidase Young untreated/image 2.JPG]

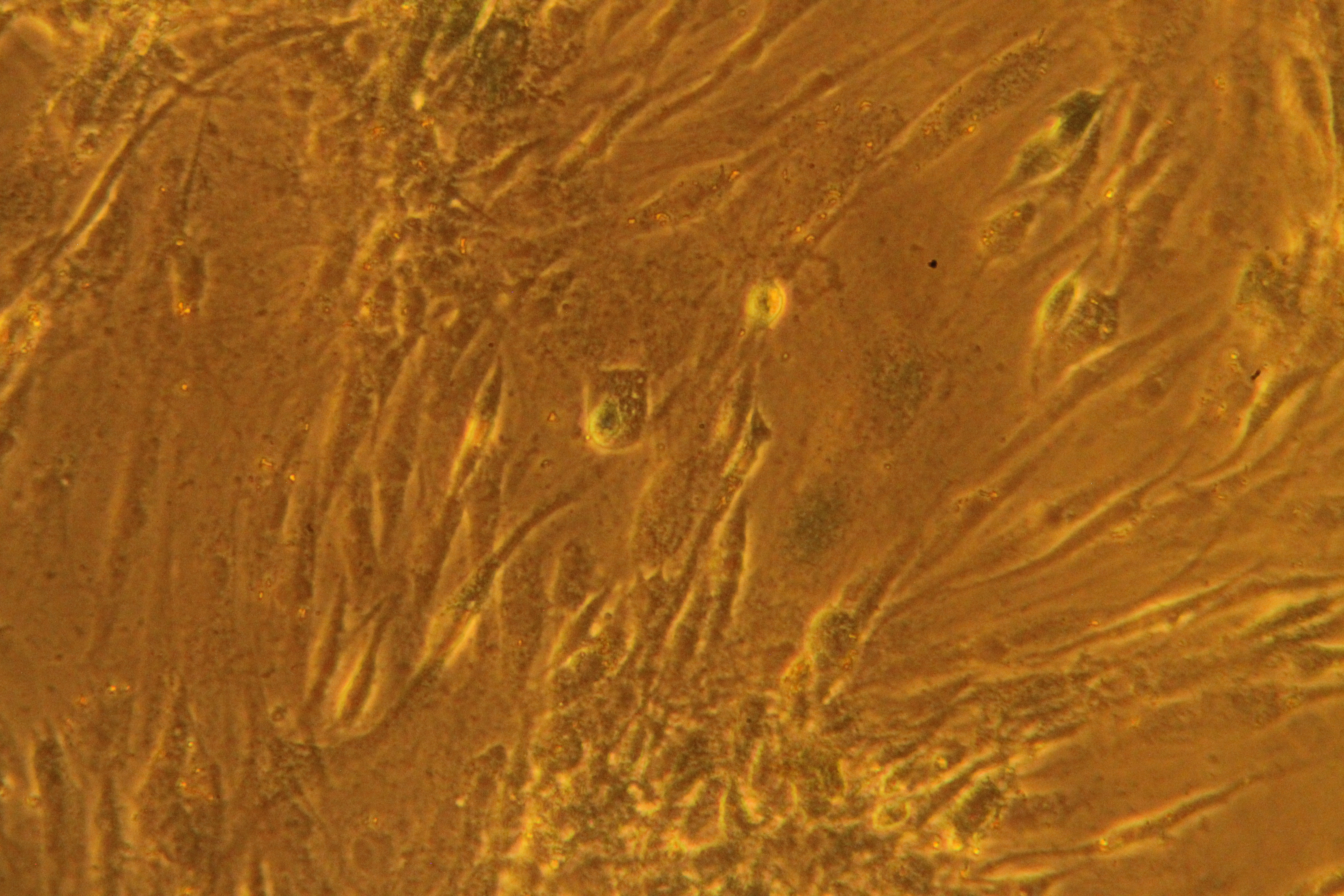

Supplement: Figure 6—source data 2. [file elife-62635-fig6-data2.zip › Figure 6-source data 2/beta galactosidase Young untreated/image 3.JPG]

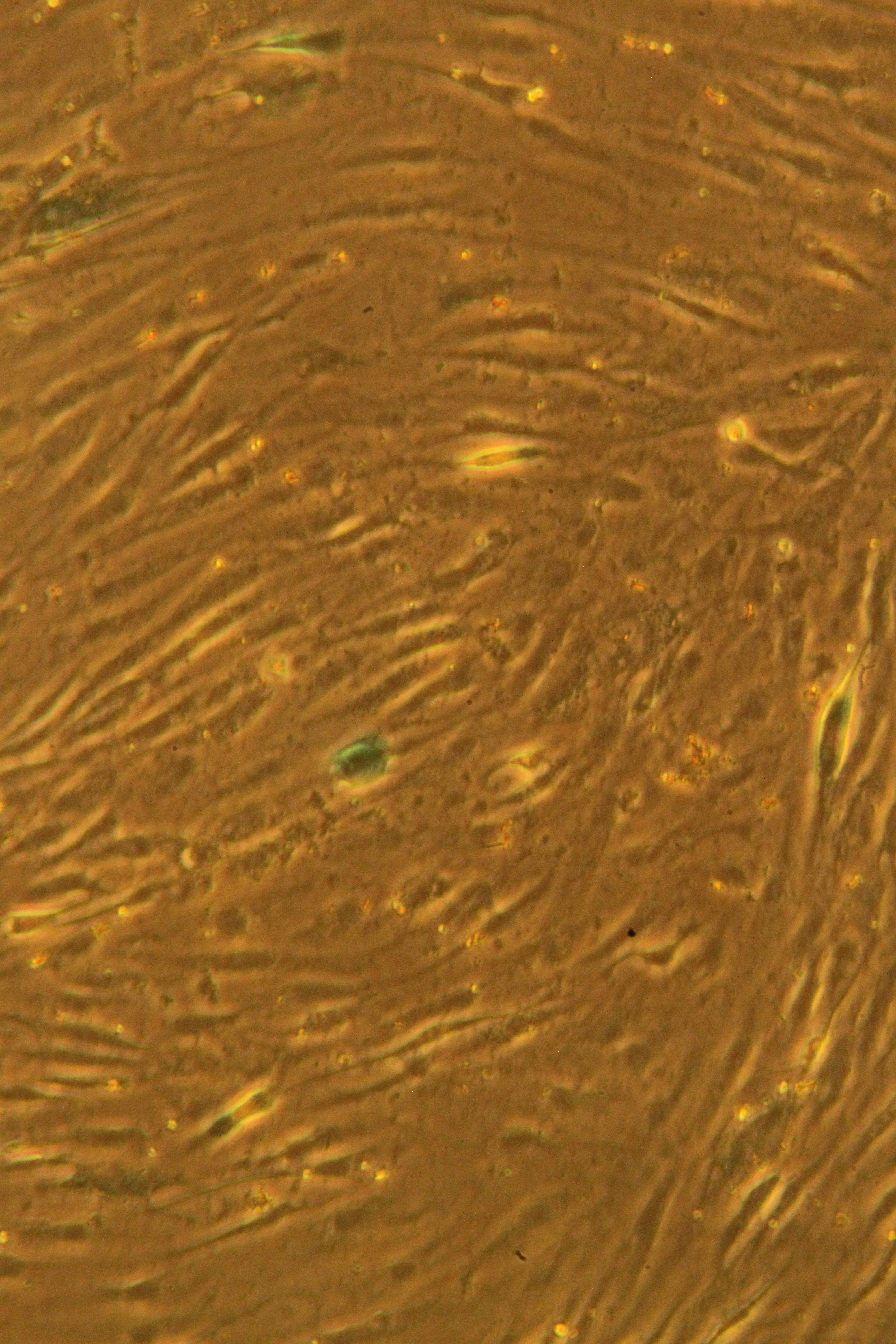

Supplement: Figure 6—source data 2. [file elife-62635-fig6-data2.zip › Figure 6-source data 2/beta galactosidase Aged untreated/image 1 .jpg]

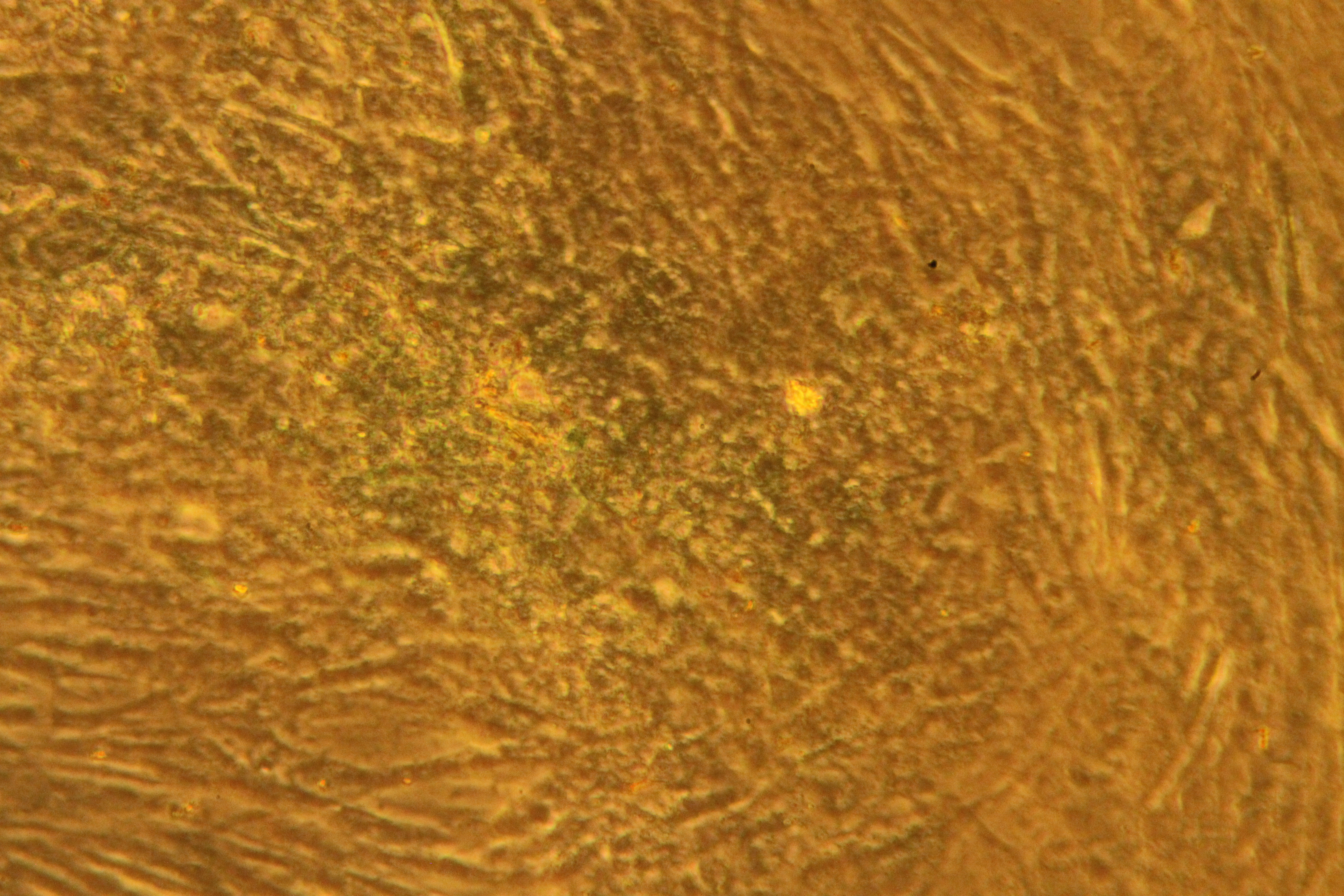

Supplement: Figure 6—source data 2. [file elife-62635-fig6-data2.zip › Figure 6-source data 2/beta galactosidase Aged untreated/image 6.JPG]

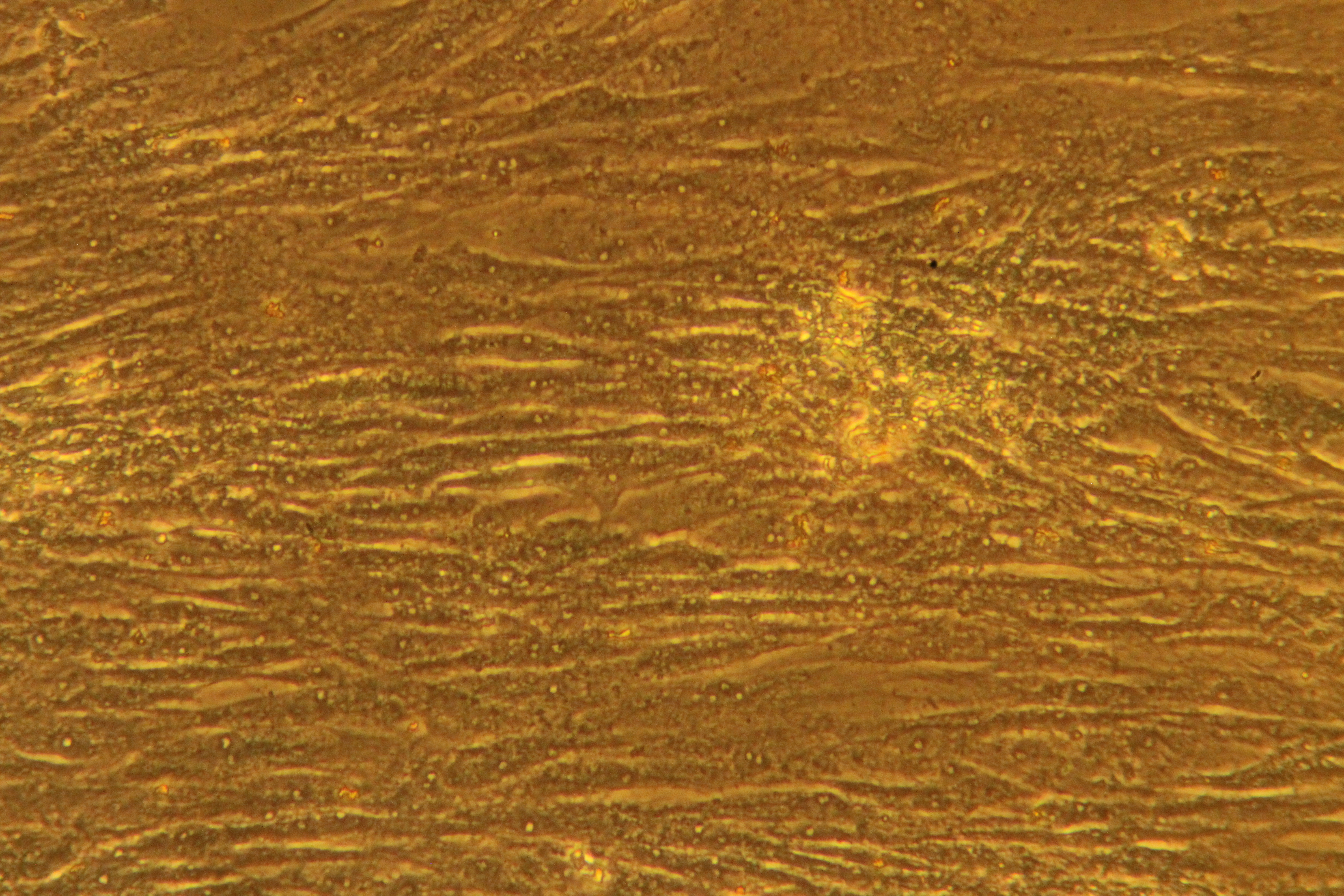

Supplement: Figure 6—source data 2. [file elife-62635-fig6-data2.zip › Figure 6-source data 2/beta galactosidase Aged untreated/image 4.JPG]

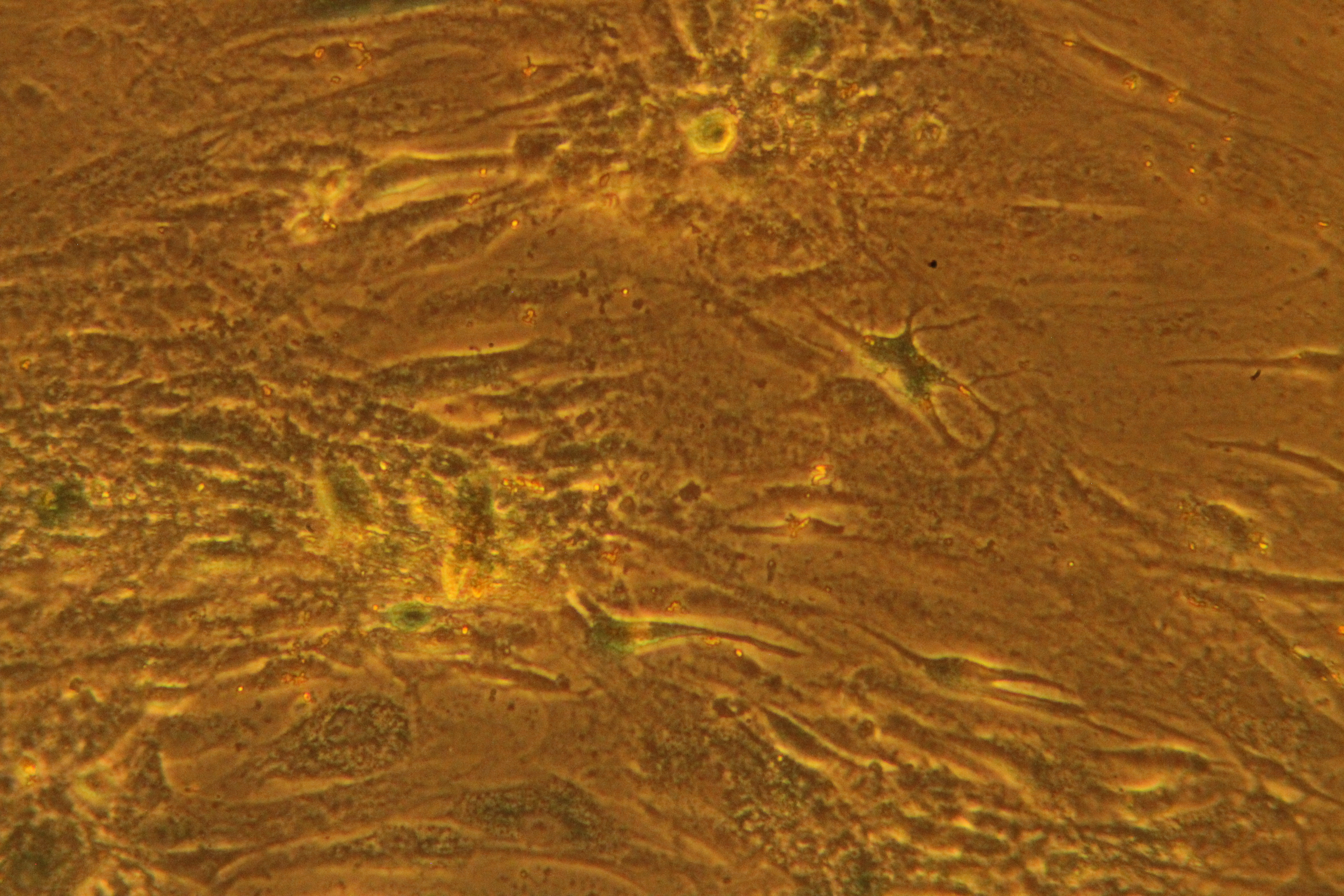

Supplement: Figure 6—source data 2. [file elife-62635-fig6-data2.zip › Figure 6-source data 2/beta galactosidase Aged untreated/image 5.JPG]

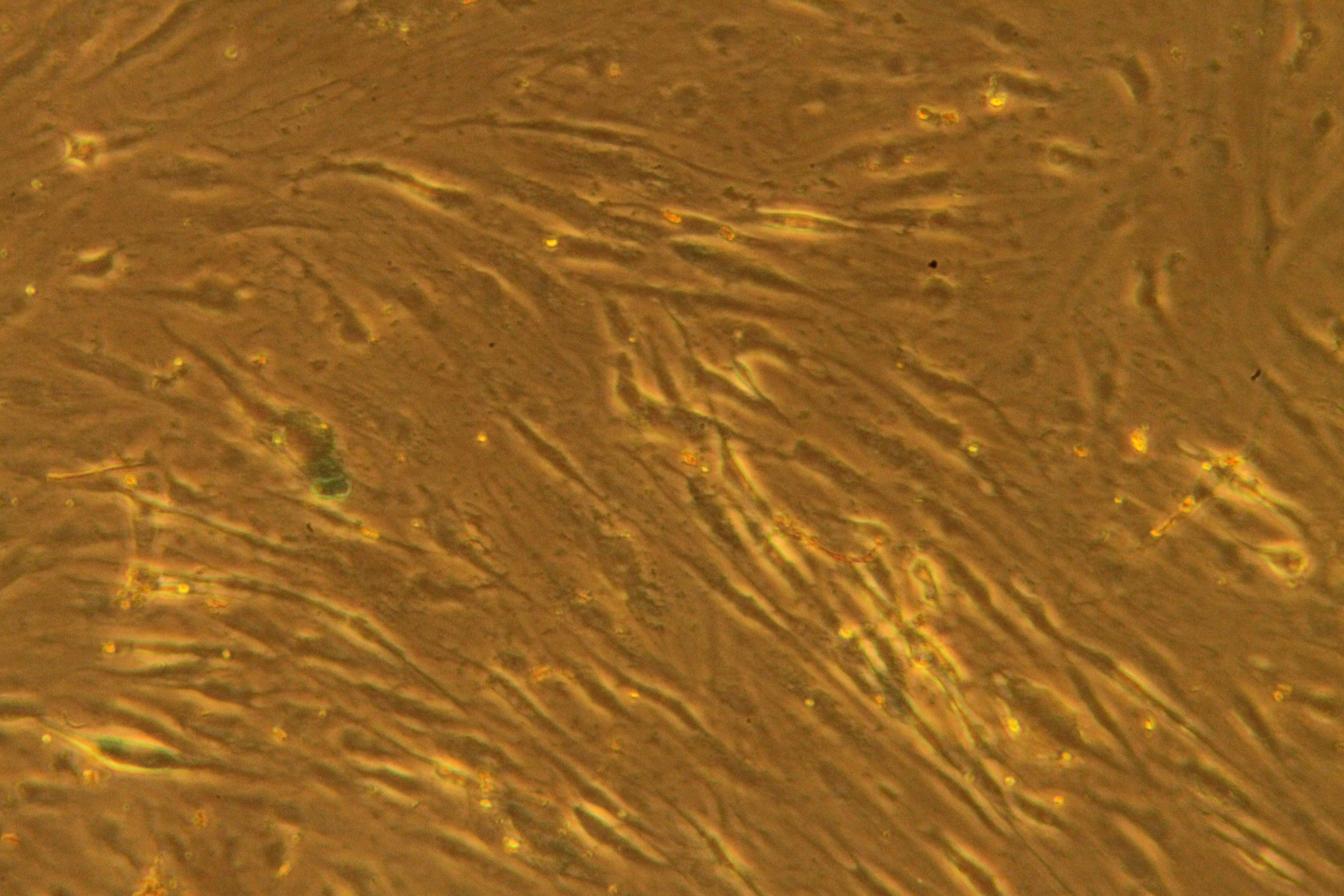

Supplement: Figure 6—source data 2. [file elife-62635-fig6-data2.zip › Figure 6-source data 2/beta galactosidase Aged untreated/image 2.JPG]

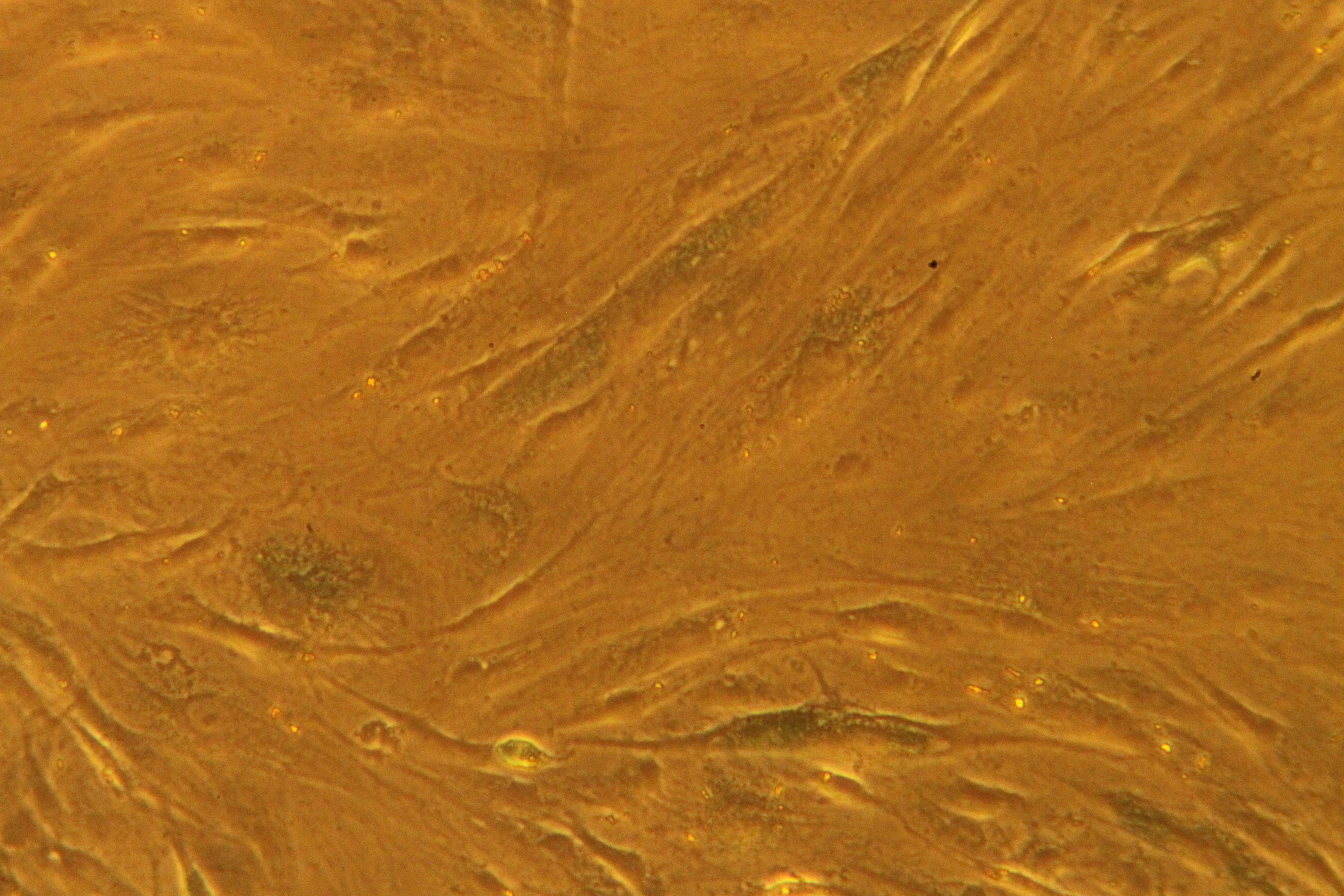

Supplement: Figure 6—source data 2. [file elife-62635-fig6-data2.zip › Figure 6-source data 2/beta galactosidase Aged untreated/image 3.JPG]

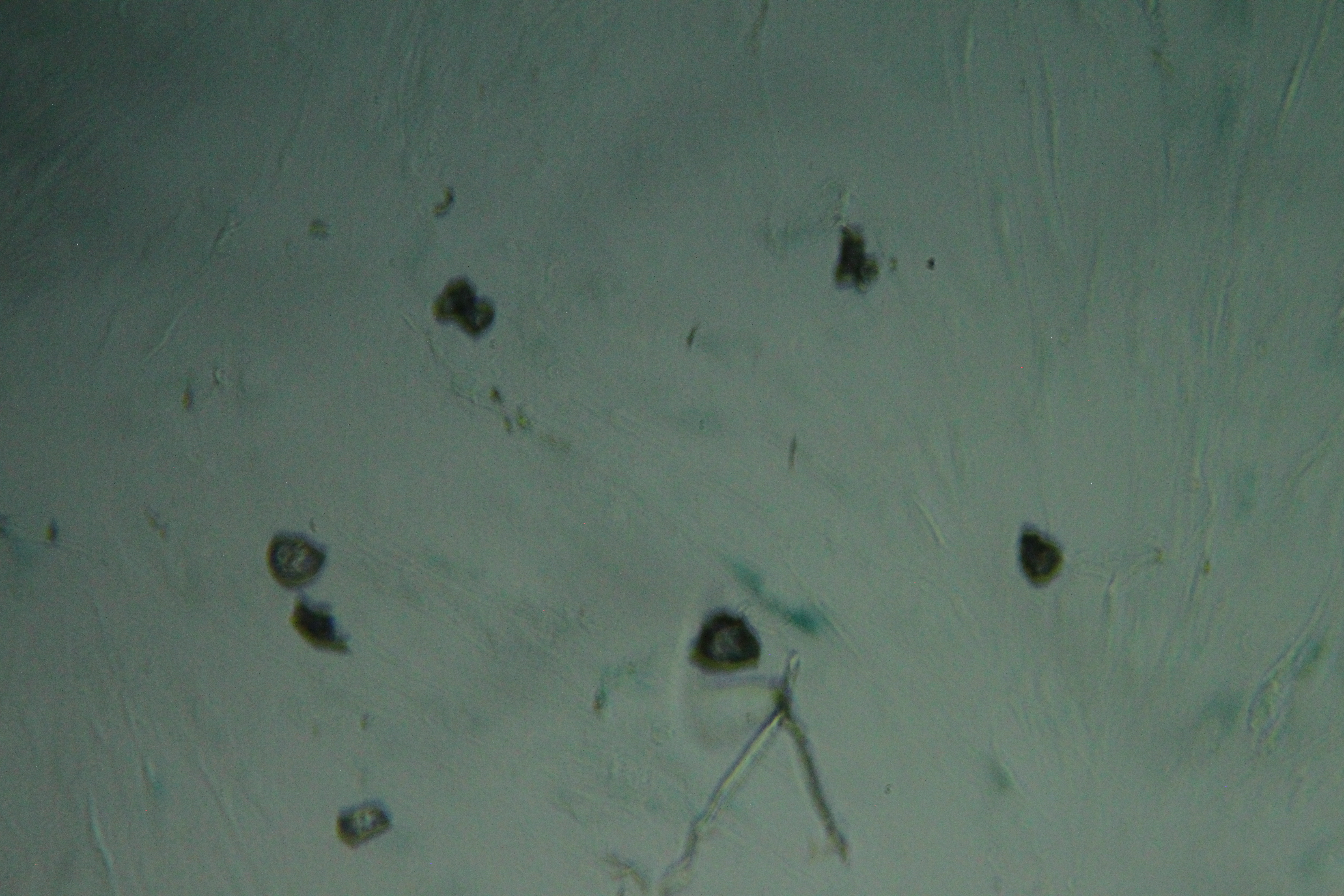

Supplement: Figure 8—source data 1. [file elife-62635-fig8-data1.zip › Figure8-source data 1/Beta galactosidase Aged/Aged Metformin/image 1.JPG]

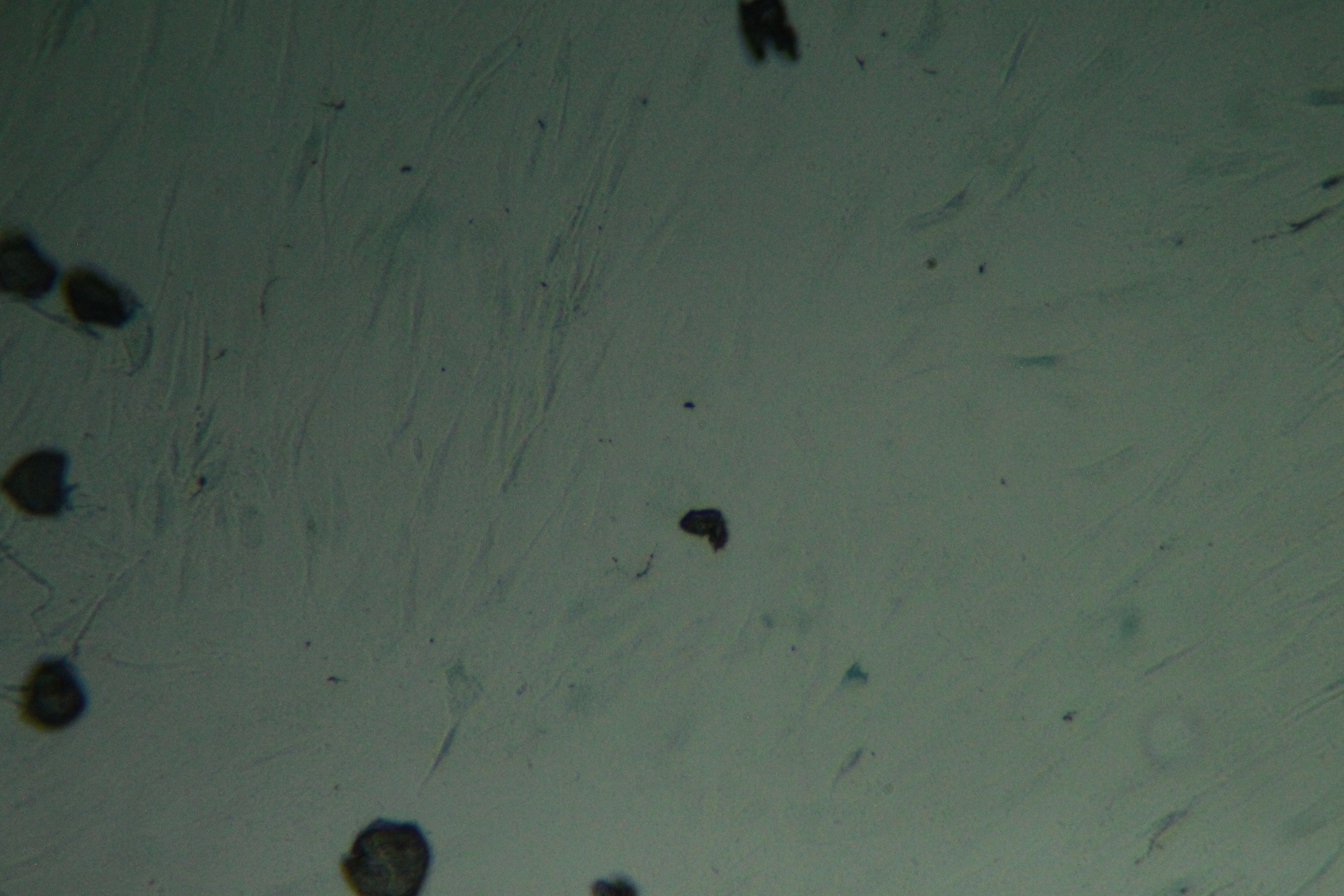

Supplement: Figure 8—source data 1. [file elife-62635-fig8-data1.zip › Figure8-source data 1/Beta galactosidase Aged/Aged Metformin/image 2.JPG]

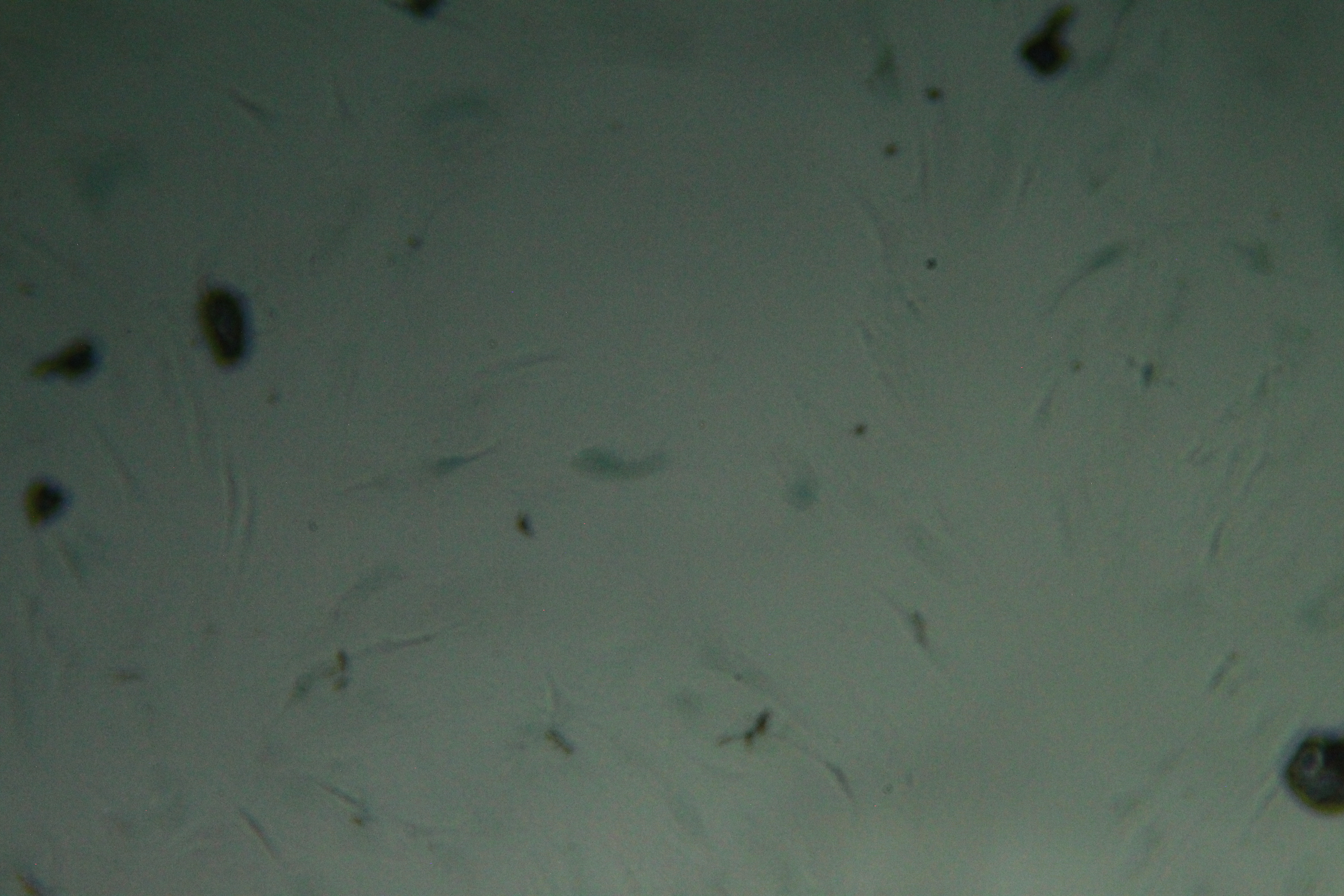

Supplement: Figure 8—source data 1. [file elife-62635-fig8-data1.zip › Figure8-source data 1/Beta galactosidase Aged/Aged Metformin/image 3.JPG]

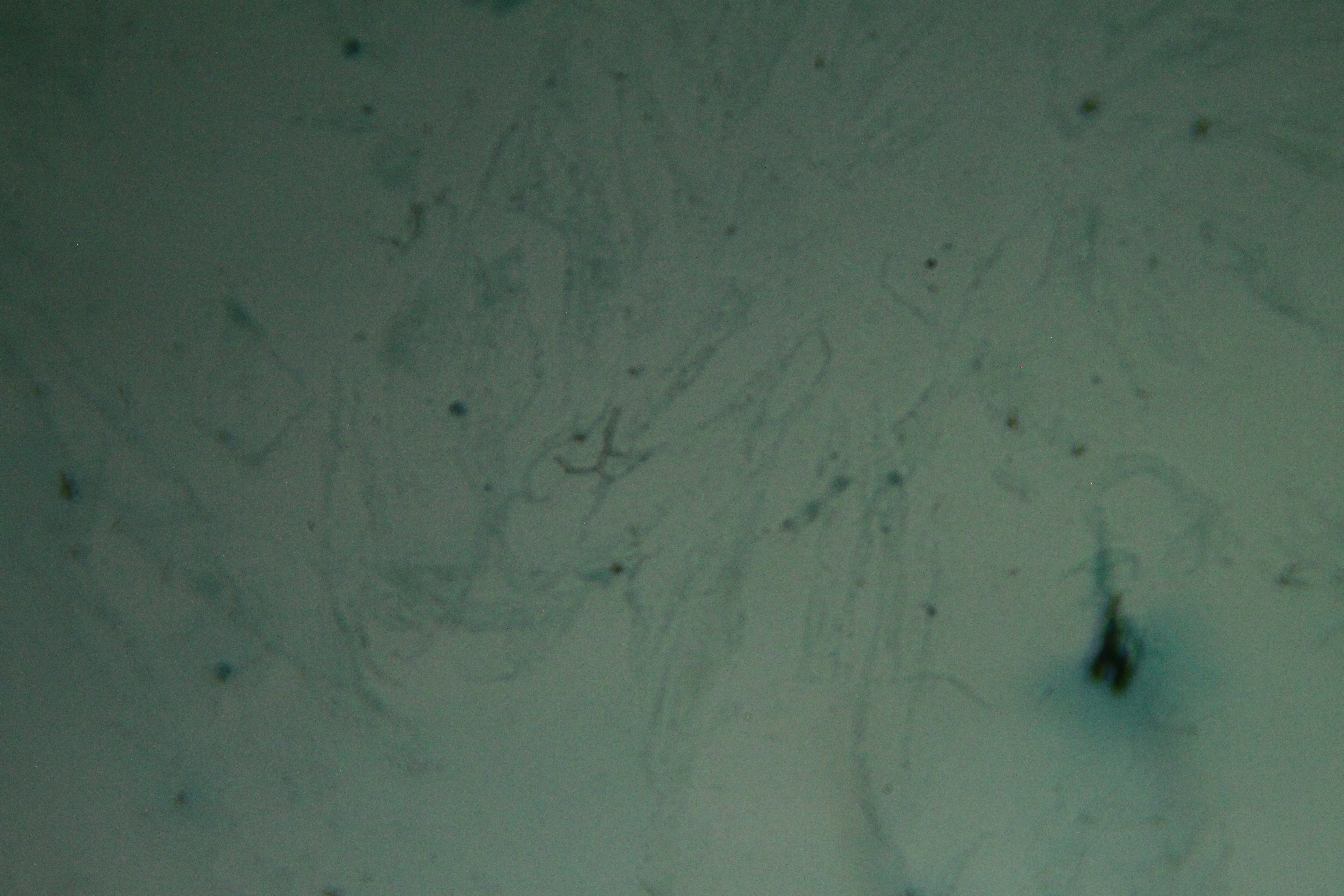

Supplement: Figure 8—source data 1. [file elife-62635-fig8-data1.zip › Figure8-source data 1/Beta galactosidase Aged/Aged Compound C/image 1.JPG]

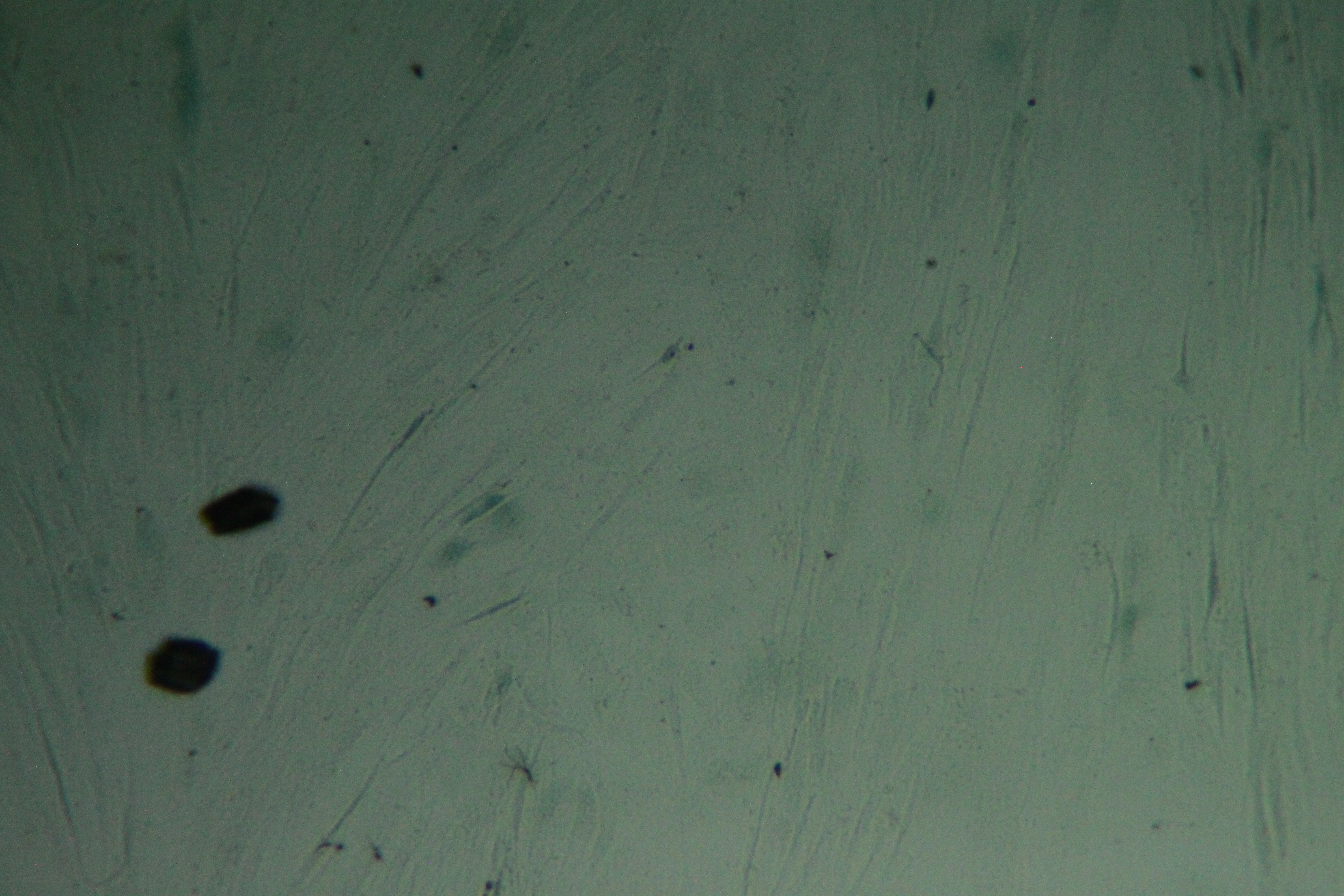

Supplement: Figure 8—source data 1. [file elife-62635-fig8-data1.zip › Figure8-source data 1/Beta galactosidase Aged/Aged Compound C/image 2.JPG]

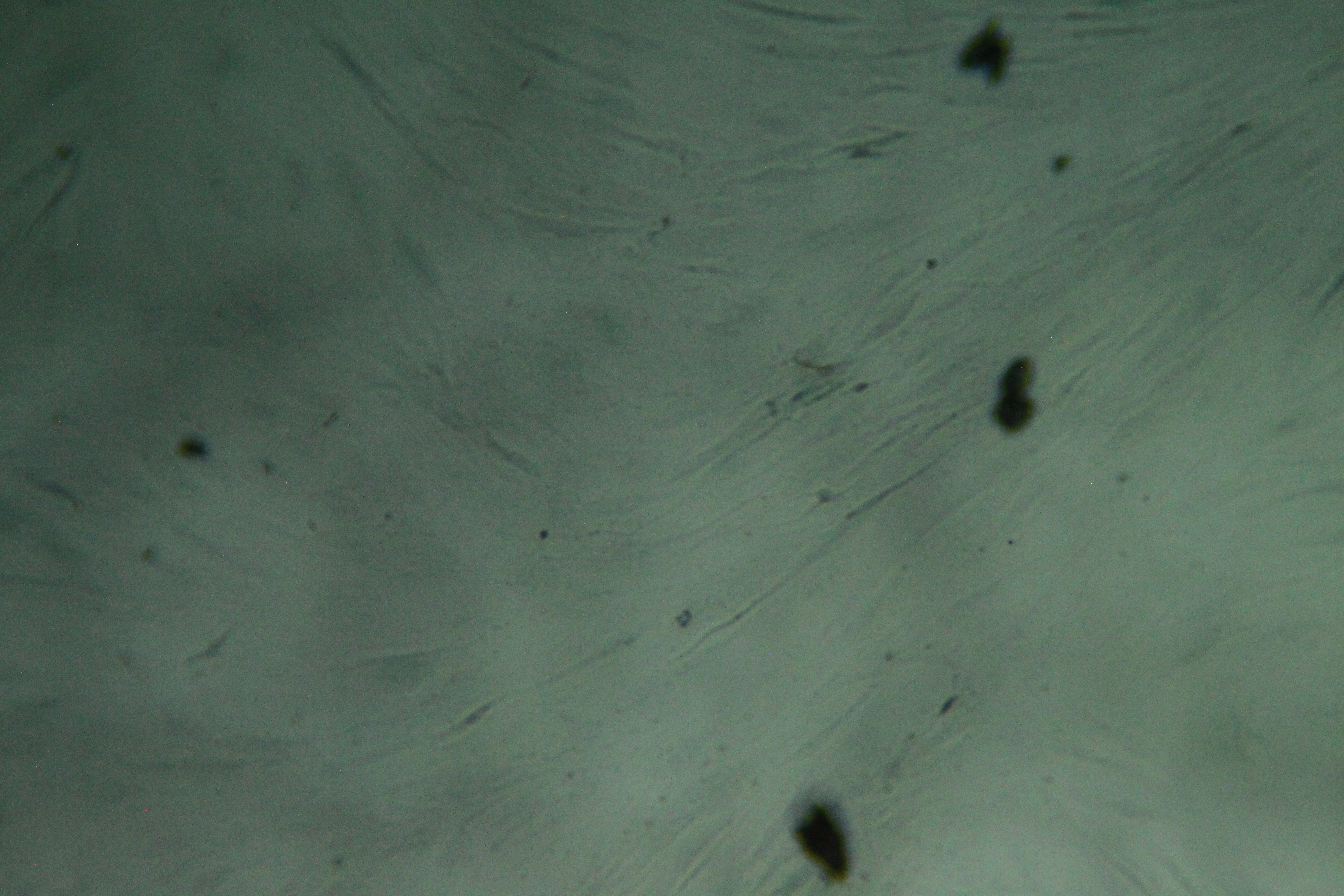

Supplement: Figure 8—source data 1. [file elife-62635-fig8-data1.zip › Figure8-source data 1/Beta galactosidase Aged/Aged Compound C/image 3.JPG]

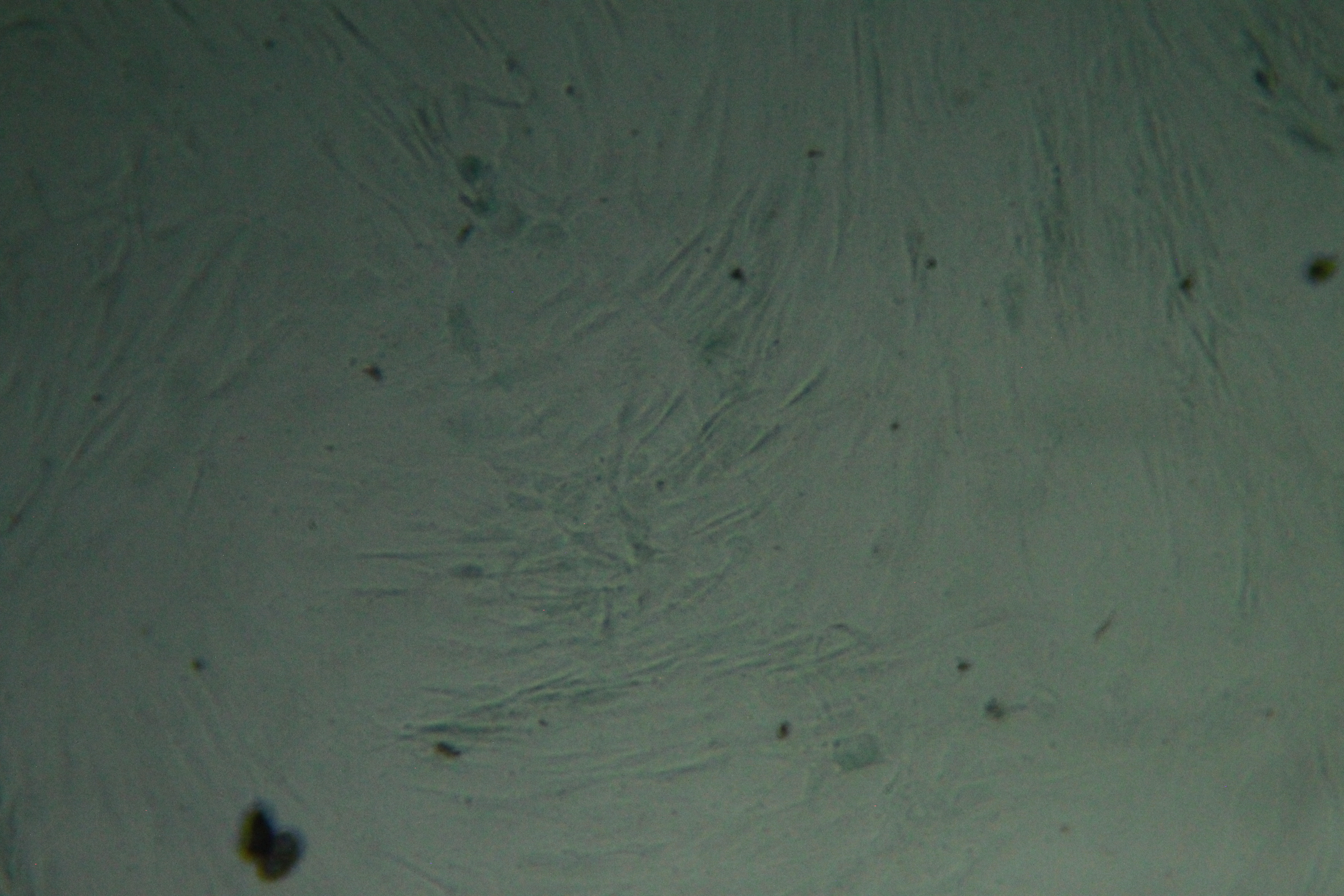

Supplement: Figure 8—source data 1. [file elife-62635-fig8-data1.zip › Figure8-source data 1/Beta galactosidase Aged/Aged Metformin Compound C/image 1.JPG]

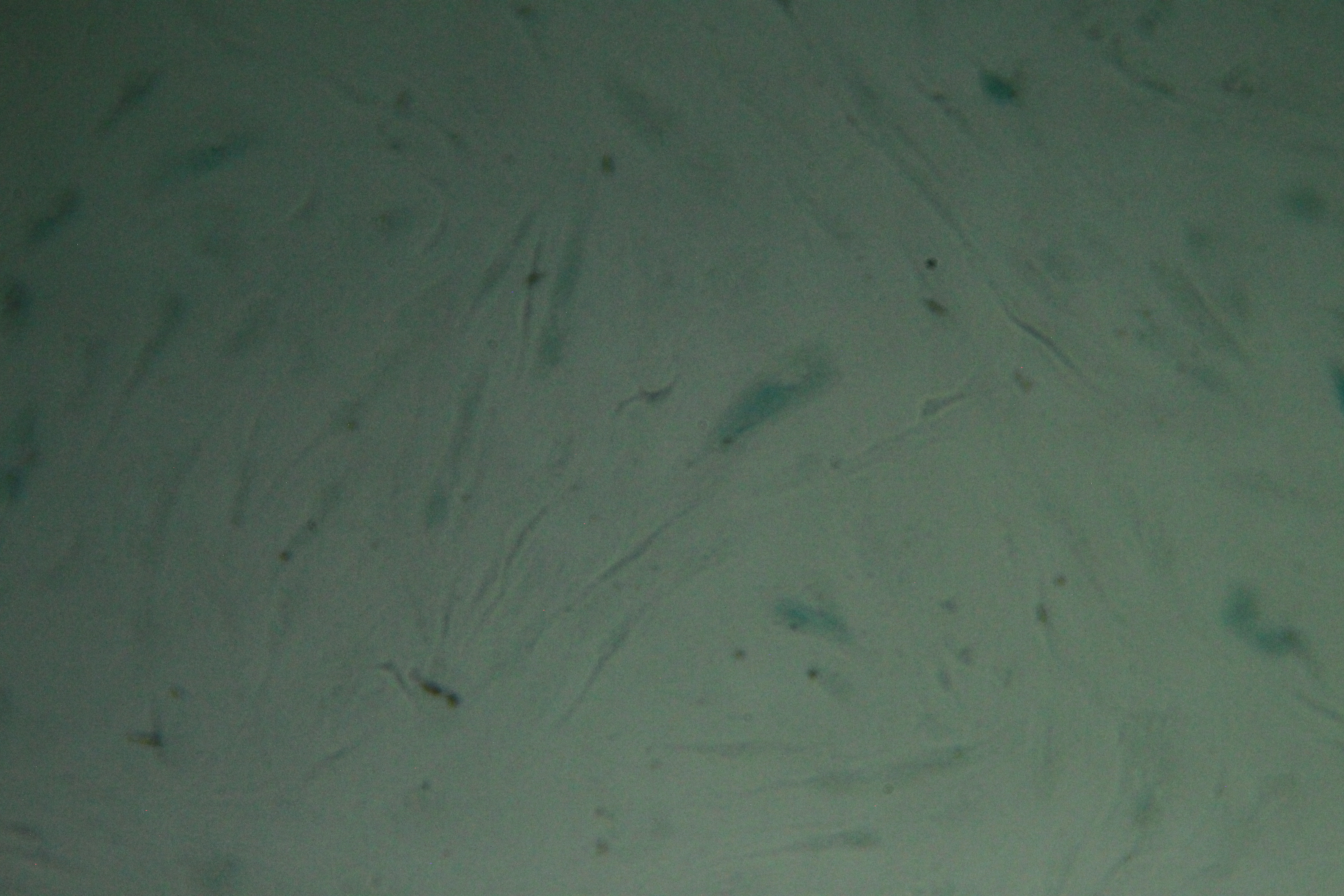

Supplement: Figure 8—source data 1. [file elife-62635-fig8-data1.zip › Figure8-source data 1/Beta galactosidase Aged/Aged Metformin Compound C/image 2.JPG]

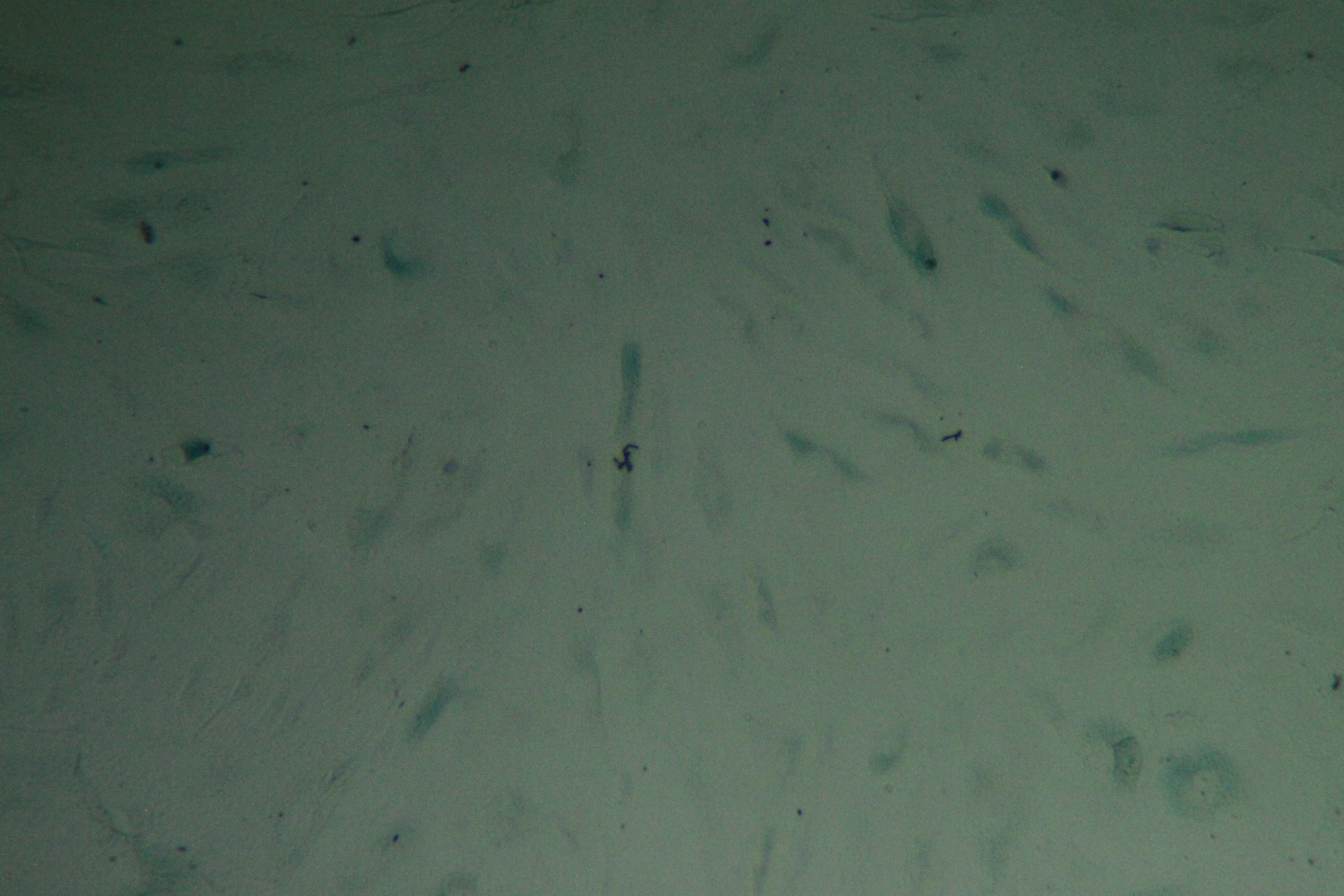

Supplement: Figure 8—source data 1. [file elife-62635-fig8-data1.zip › Figure8-source data 1/Beta galactosidase Aged/Aged Metformin Compound C/image 3.JPG]

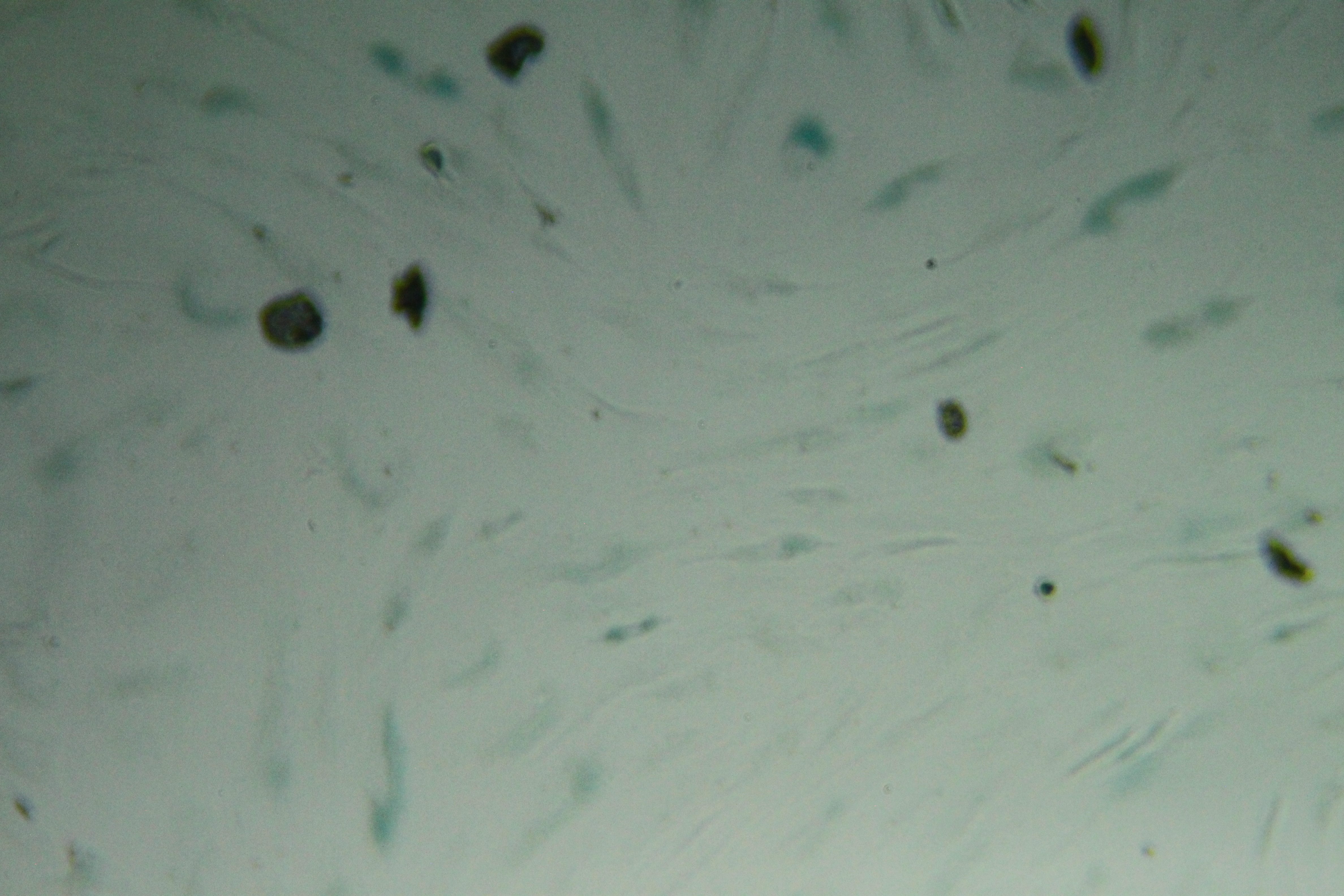

Supplement: Figure 8—source data 1. [file elife-62635-fig8-data1.zip › Figure8-source data 1/Beta galactosidase Aged/Aged untreated/image 1.JPG]

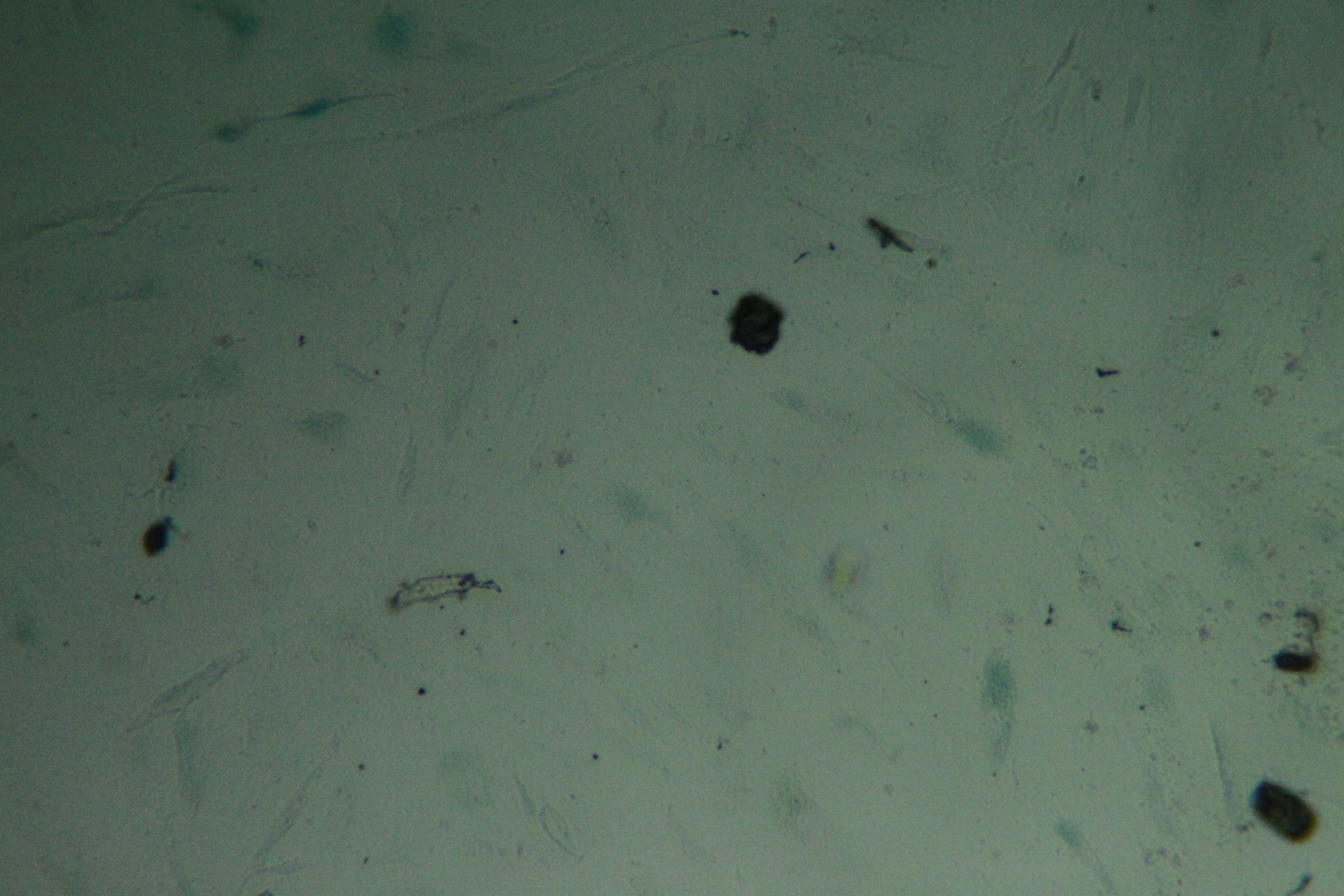

Supplement: Figure 8—source data 1. [file elife-62635-fig8-data1.zip › Figure8-source data 1/Beta galactosidase Aged/Aged untreated/image 2.JPG]

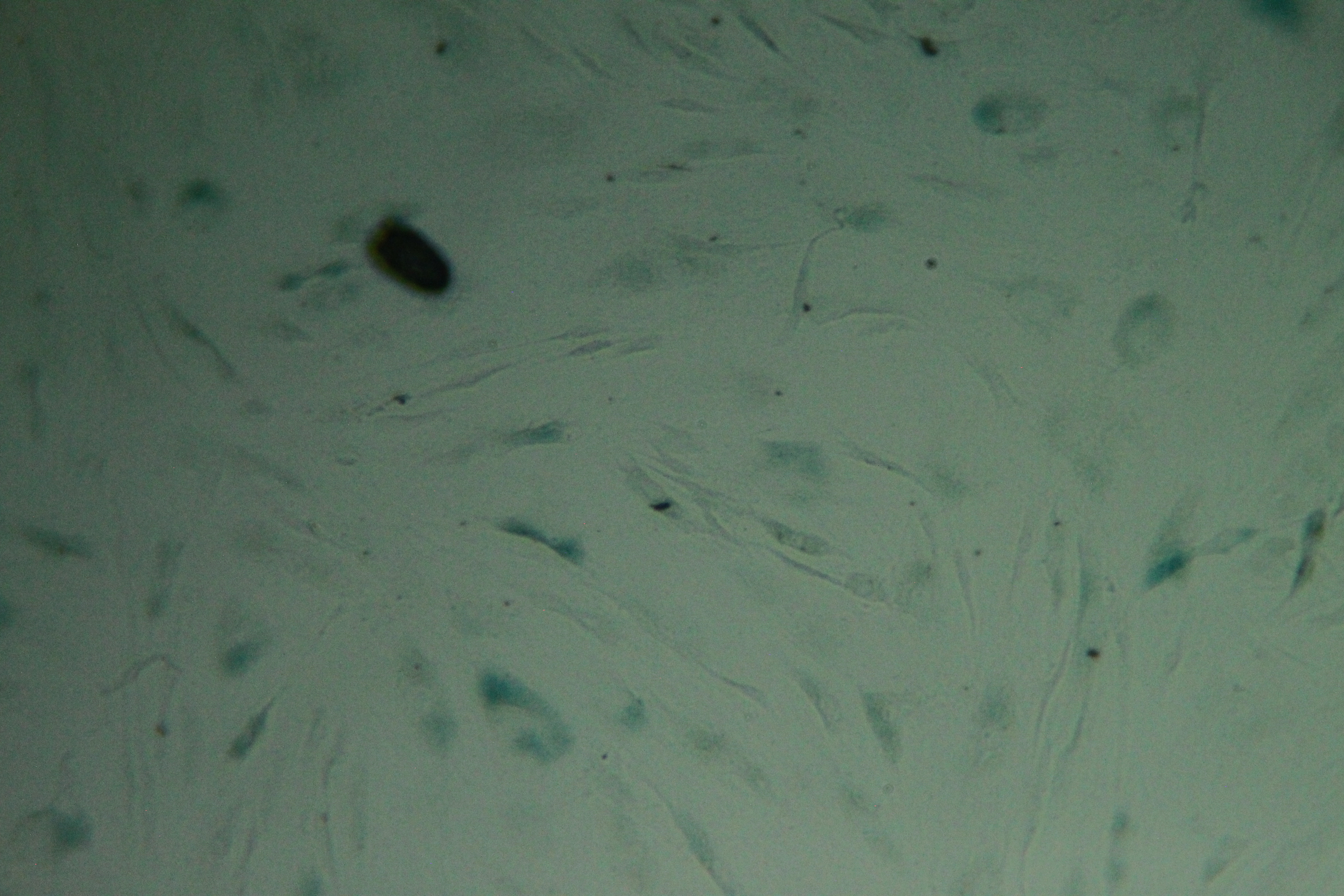

Supplement: Figure 8—source data 1. [file elife-62635-fig8-data1.zip › Figure8-source data 1/Beta galactosidase Aged/Aged untreated/image 3.JPG]

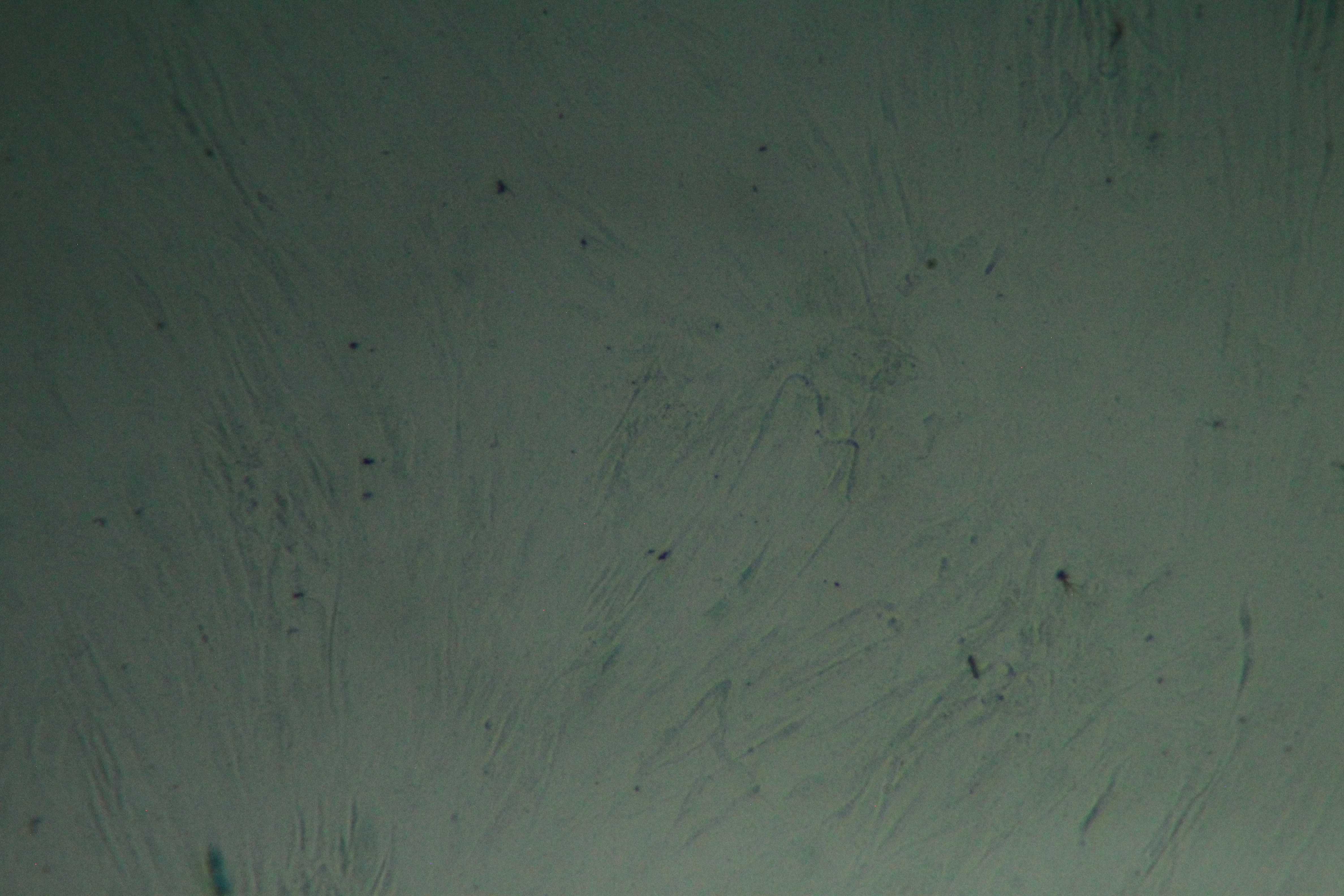

Supplement: Figure 8—source data 2. [file elife-62635-fig8-data2.zip › Figure8-source data 2/Beta galactosidase Young/Young Metformin/image 1.JPG]

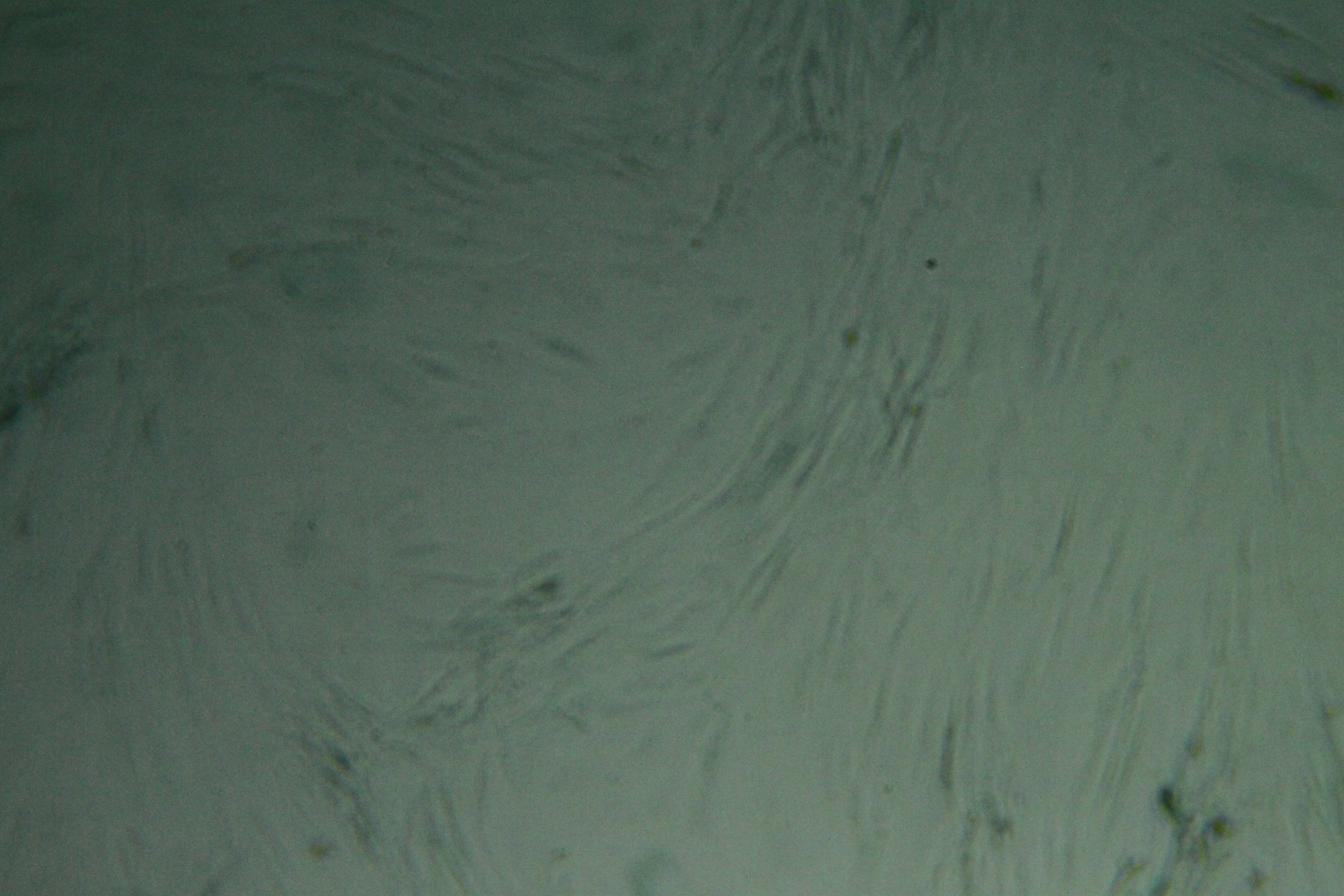

Supplement: Figure 8—source data 2. [file elife-62635-fig8-data2.zip › Figure8-source data 2/Beta galactosidase Young/Young Metformin/image 2.JPG]

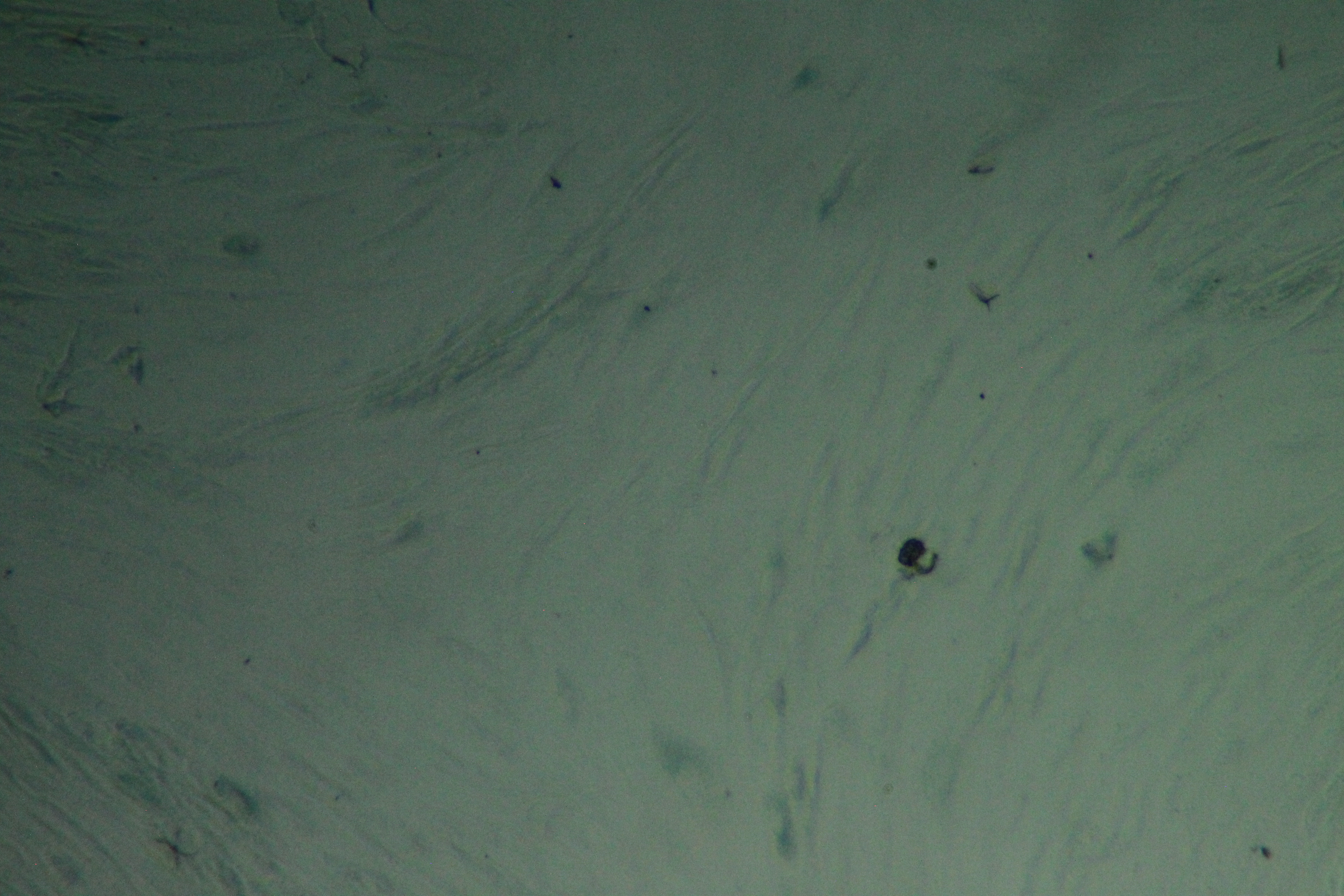

Supplement: Figure 8—source data 2. [file elife-62635-fig8-data2.zip › Figure8-source data 2/Beta galactosidase Young/Young Metformin/image 3.JPG]

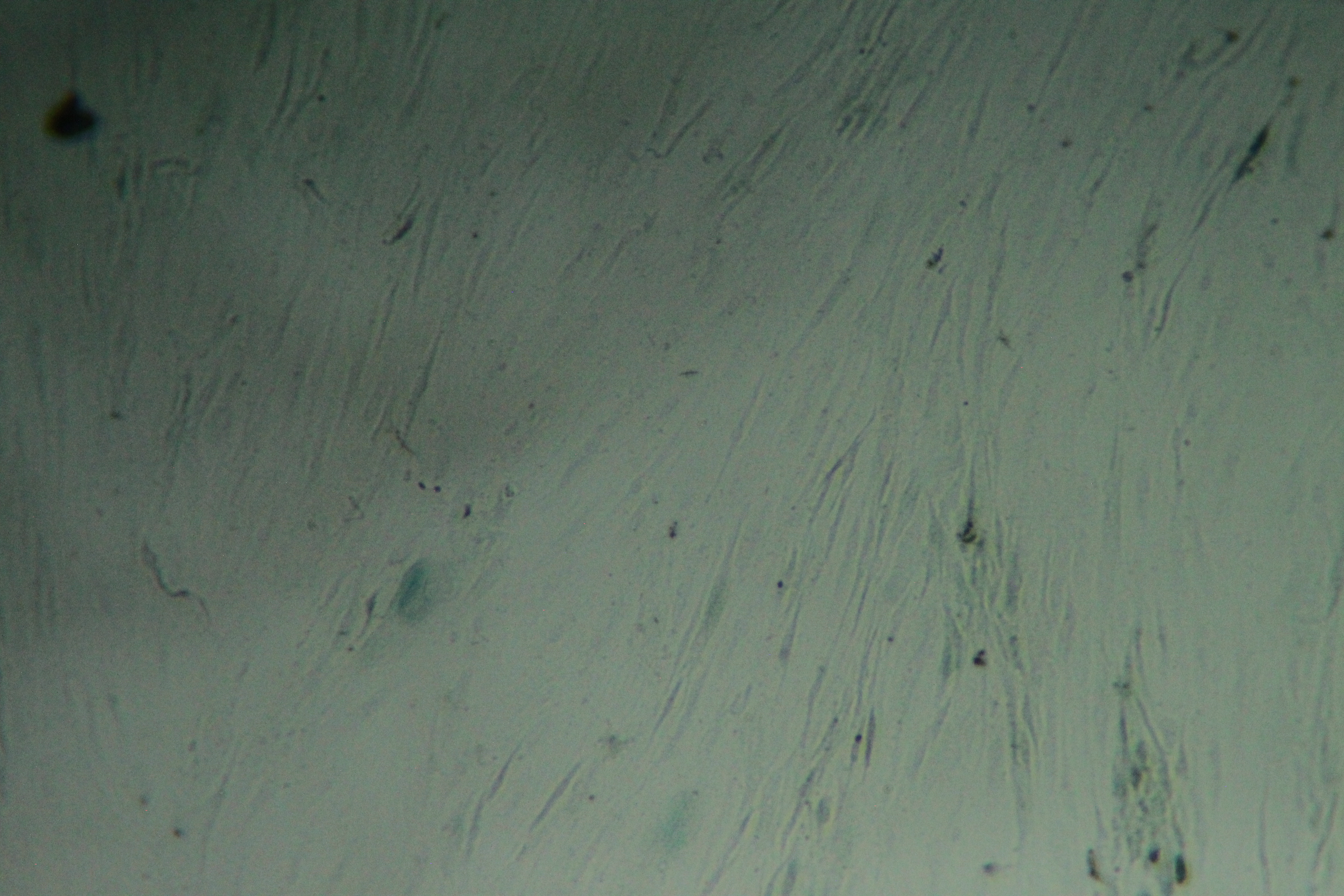

Supplement: Figure 8—source data 2. [file elife-62635-fig8-data2.zip › Figure8-source data 2/Beta galactosidase Young/Young untreated/image 1.JPG]

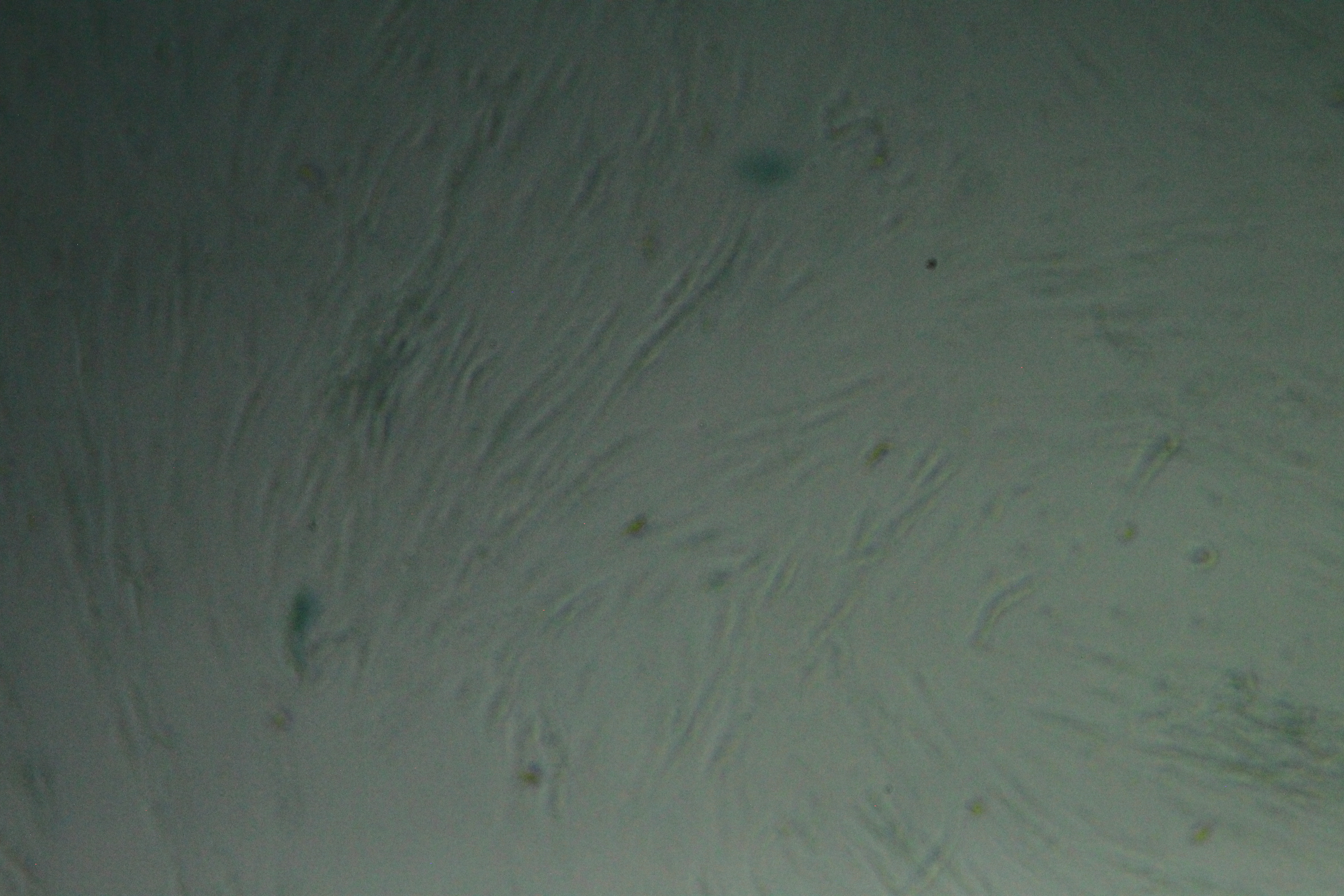

Supplement: Figure 8—source data 2. [file elife-62635-fig8-data2.zip › Figure8-source data 2/Beta galactosidase Young/Young untreated/image 2.JPG]

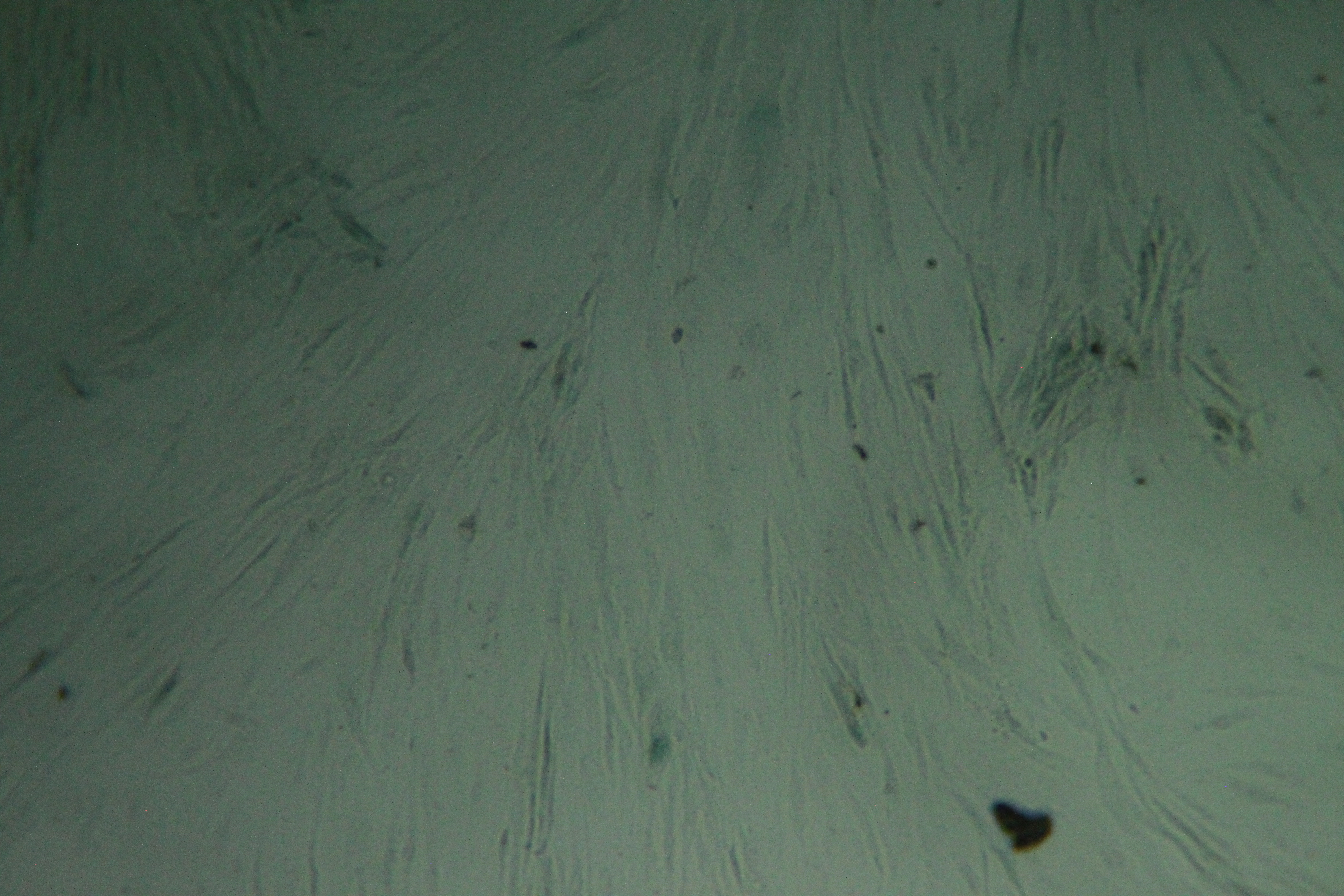

Supplement: Figure 8—source data 2. [file elife-62635-fig8-data2.zip › Figure8-source data 2/Beta galactosidase Young/Young untreated/image 3.JPG]

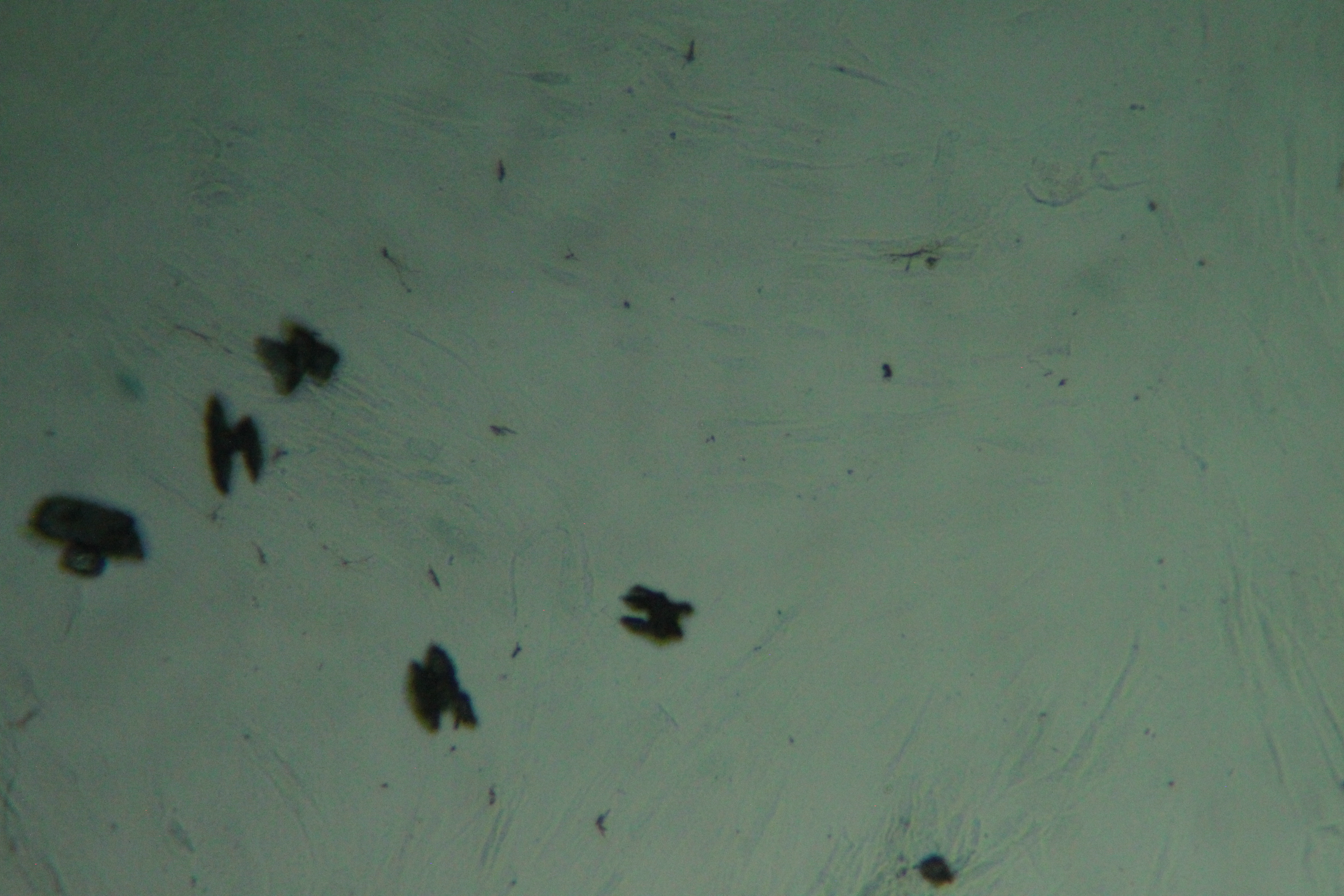

Supplement: Figure 8—source data 2. [file elife-62635-fig8-data2.zip › Figure8-source data 2/Beta galactosidase Young/Young Metformin Compound C/image 1.JPG]
