# Supplementary figures and images for: Metformin alleviates stress-induced cellular senescence of aging human adipose stromal cells and the ensuing adipocyte dysfunction (part 2 of 2)
Source: eLife. 2021 Sep 21;10:e62635. doi: 10.7554/eLife.62635 (PMC8526089; doi:10.7554/eLife.62635)

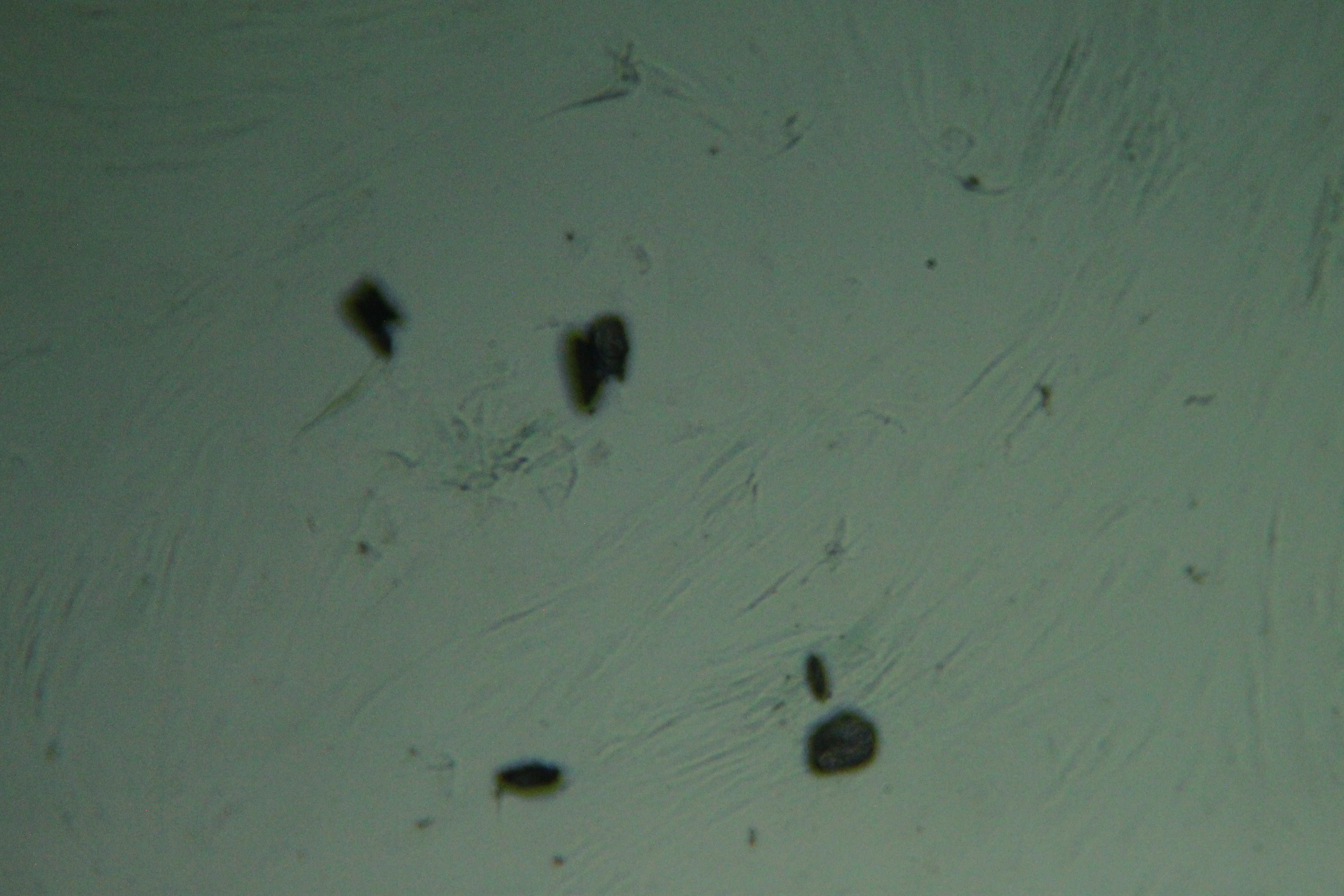

Supplement: Figure 8—source data 2. [file elife-62635-fig8-data2.zip › Figure8-source data 2/Beta galactosidase Young/Young Metformin Compound C/image 2.JPG]

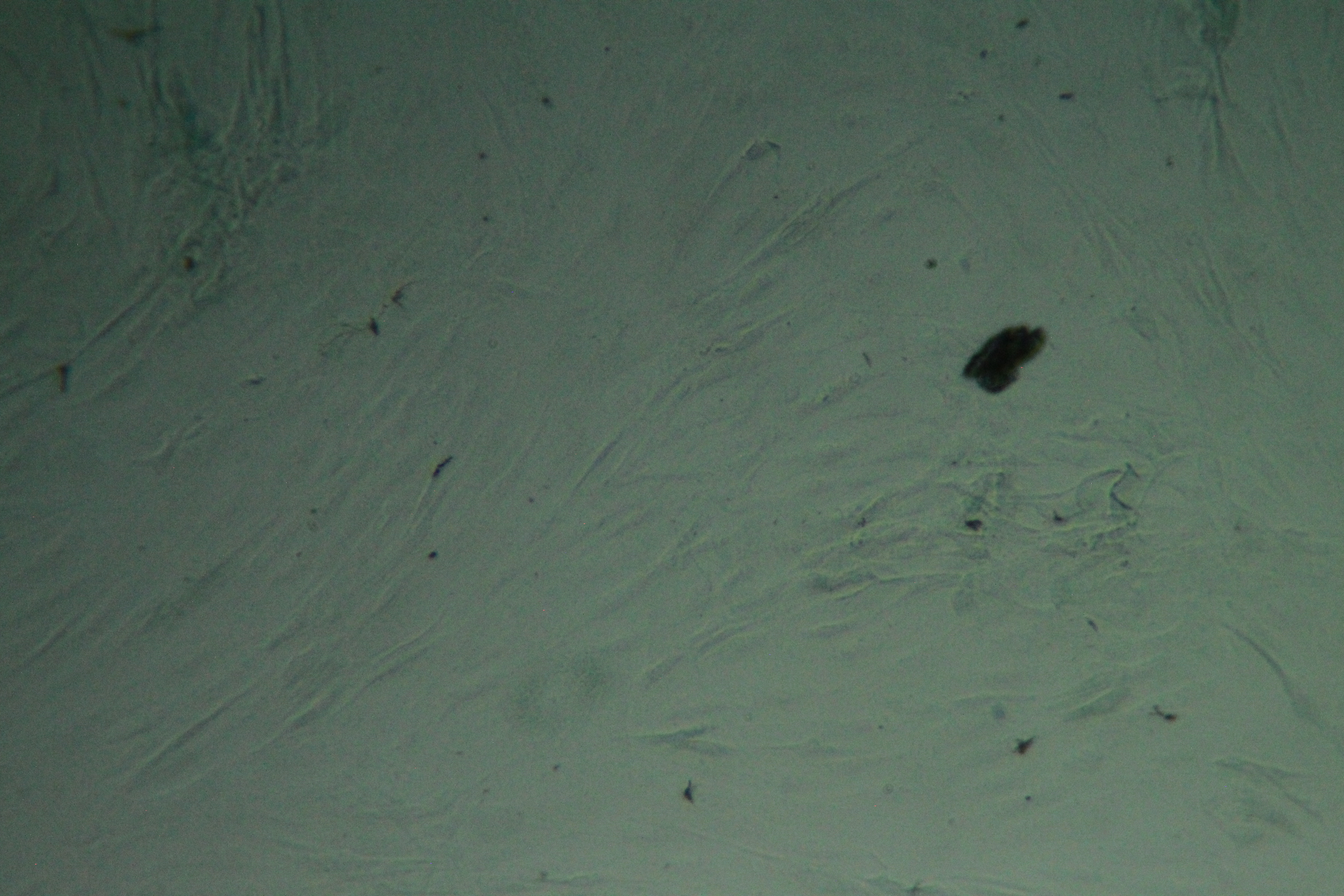

Supplement: Figure 8—source data 2. [file elife-62635-fig8-data2.zip › Figure8-source data 2/Beta galactosidase Young/Young Metformin Compound C/image 3.JPG]

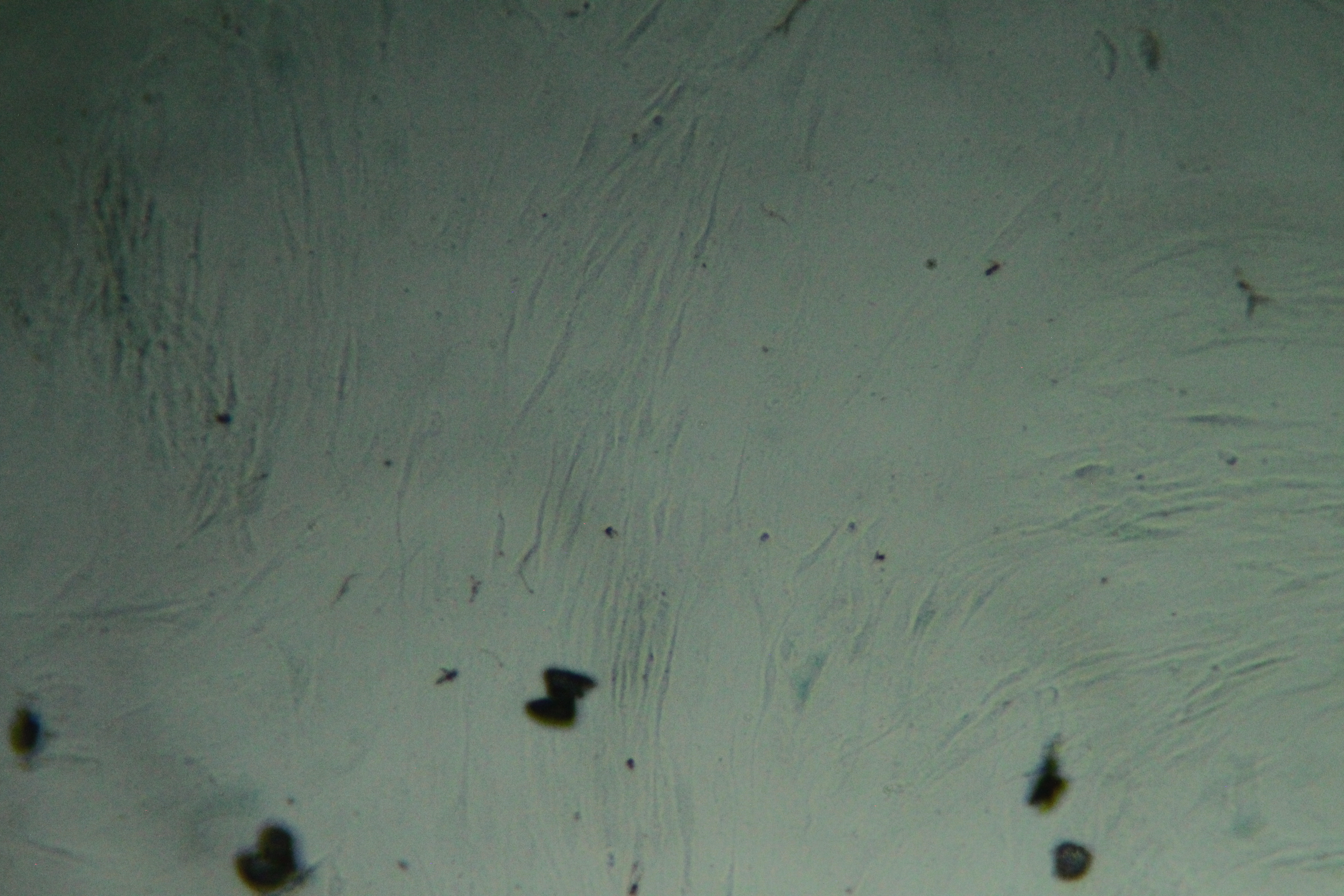

Supplement: Figure 8—source data 2. [file elife-62635-fig8-data2.zip › Figure8-source data 2/Beta galactosidase Young/Young Compound C/image 1.JPG]

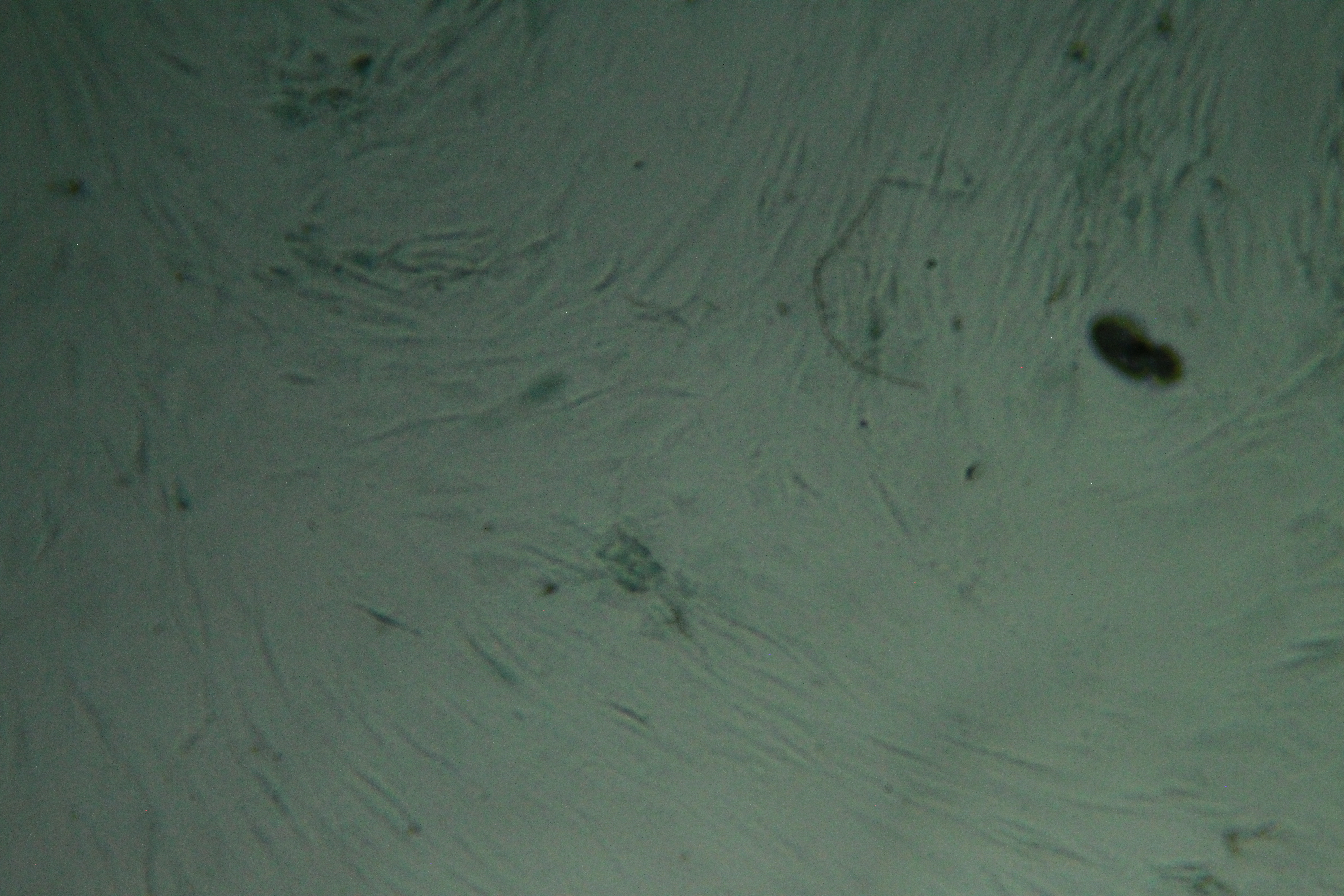

Supplement: Figure 8—source data 2. [file elife-62635-fig8-data2.zip › Figure8-source data 2/Beta galactosidase Young/Young Compound C/image 2.JPG]

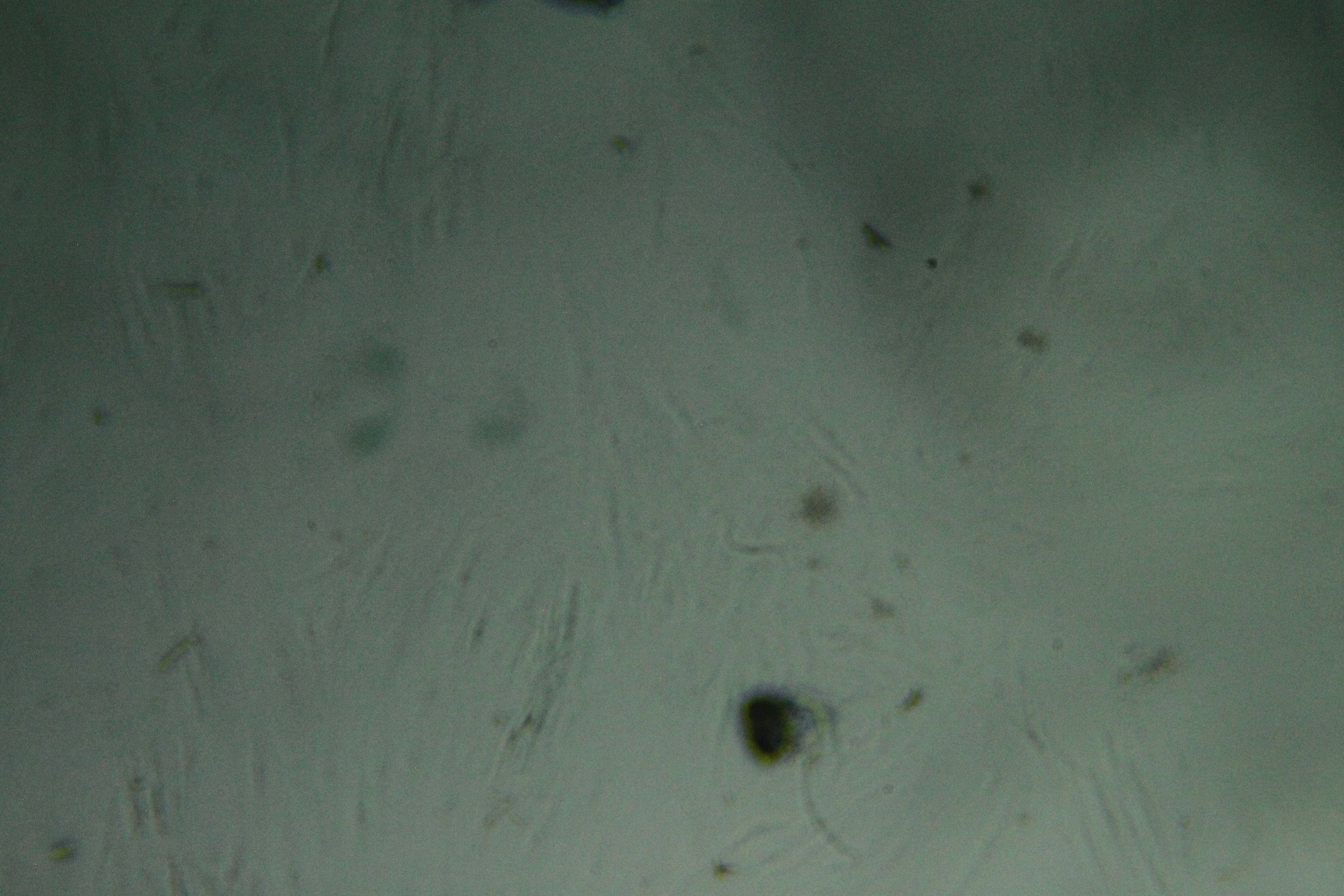

Supplement: Figure 8—source data 2. [file elife-62635-fig8-data2.zip › Figure8-source data 2/Beta galactosidase Young/Young Compound C/image 3.JPG]

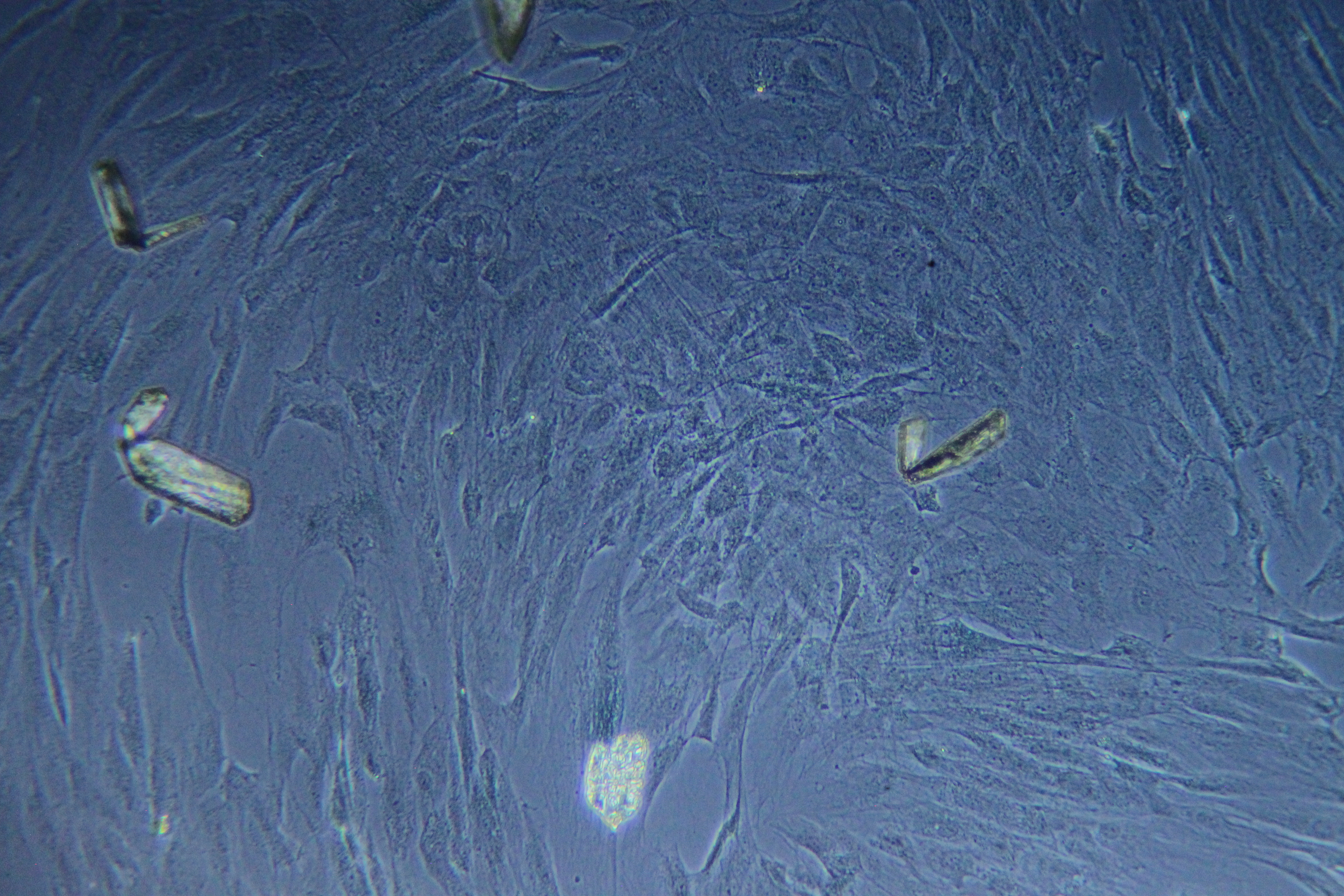

Supplement: Figure 8—figure supplement 1—source data 1. [file elife-62635-fig8-figsupp1-data1.zip › Figure 8-figure supplement 1 -Source Data 1/beta galactosidase Young untreated/image 6.JPG]

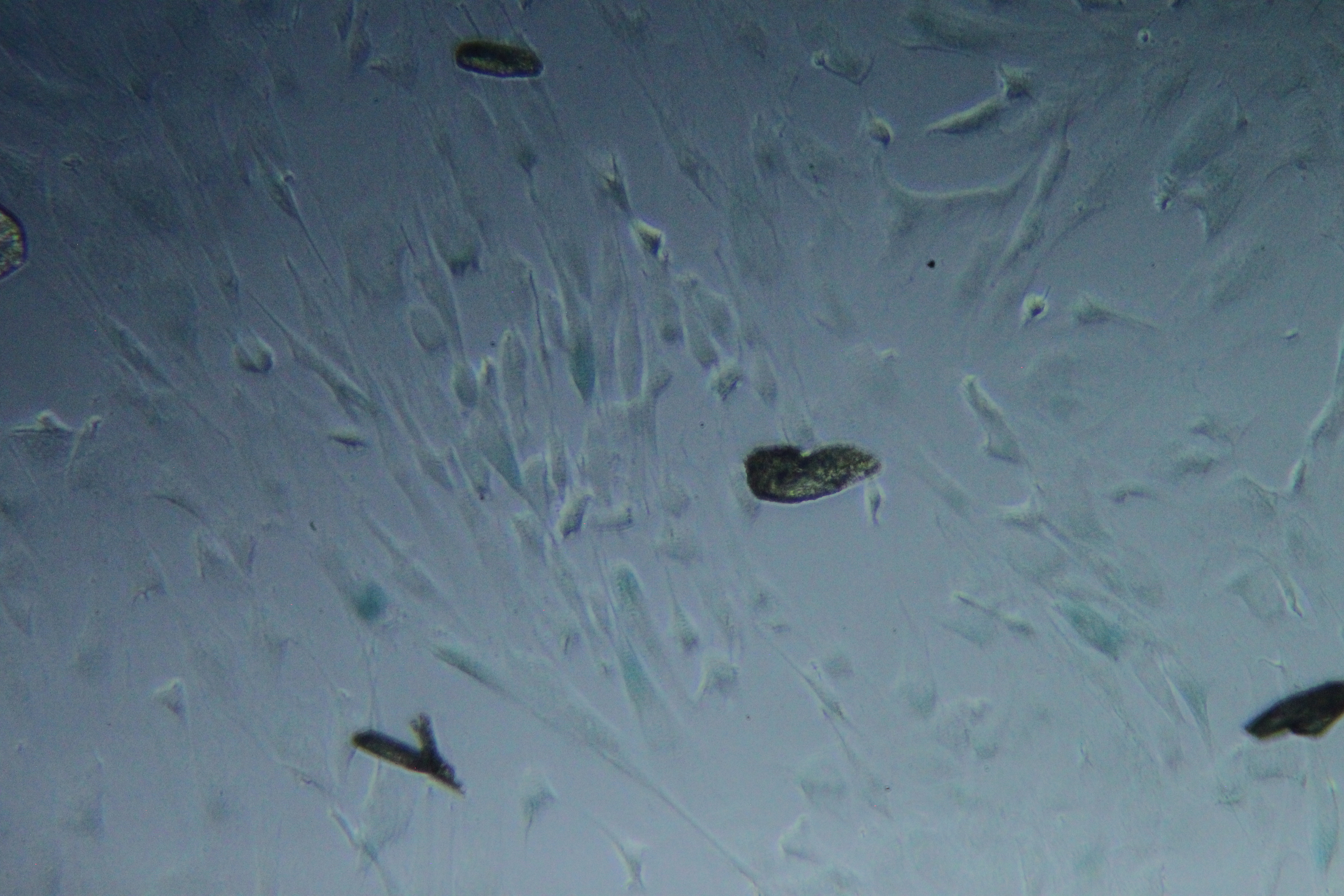

Supplement: Figure 8—figure supplement 1—source data 1. [file elife-62635-fig8-figsupp1-data1.zip › Figure 8-figure supplement 1 -Source Data 1/beta galactosidase Young untreated/image 4.JPG]

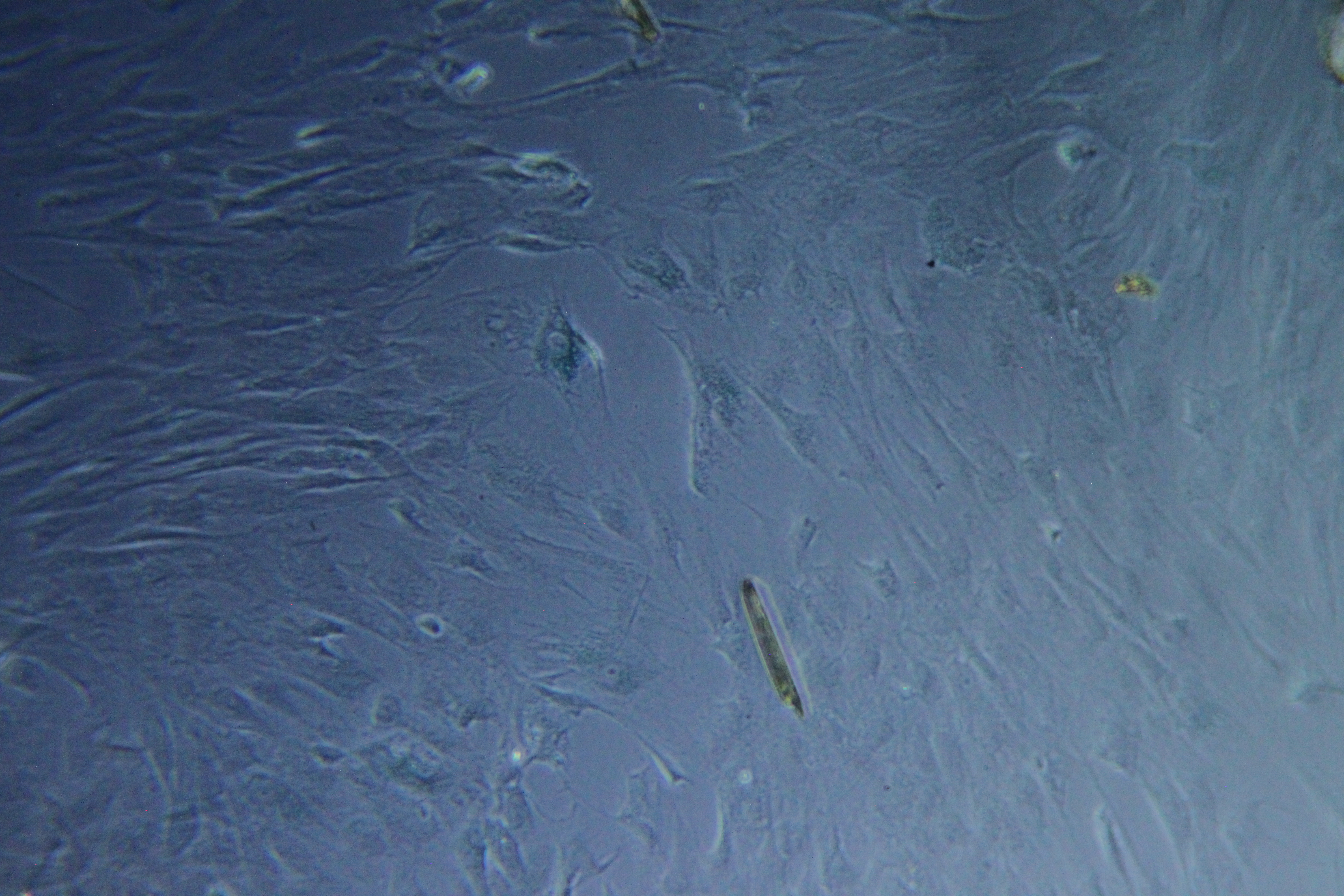

Supplement: Figure 8—figure supplement 1—source data 1. [file elife-62635-fig8-figsupp1-data1.zip › Figure 8-figure supplement 1 -Source Data 1/beta galactosidase Young untreated/image 5.JPG]

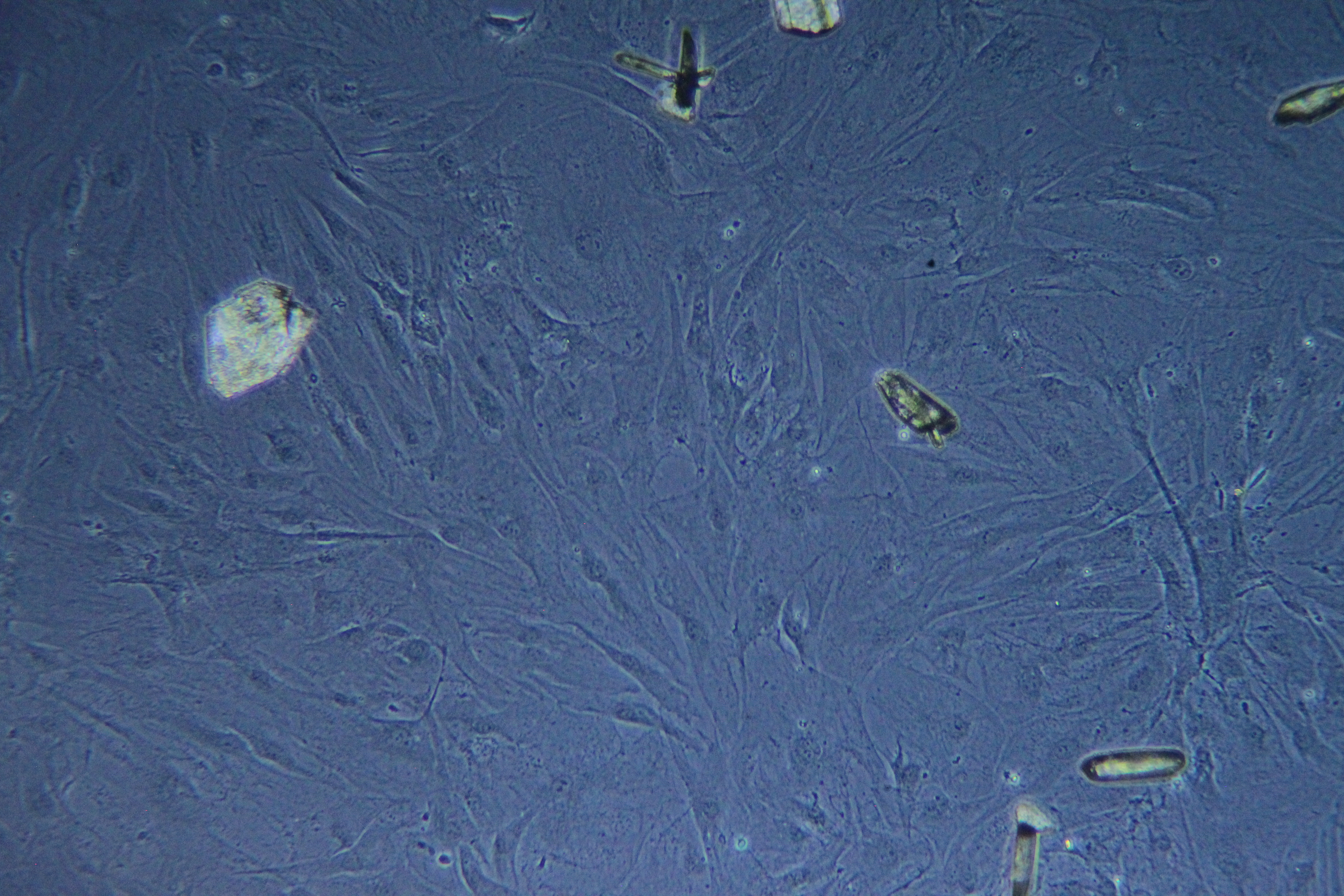

Supplement: Figure 8—figure supplement 1—source data 1. [file elife-62635-fig8-figsupp1-data1.zip › Figure 8-figure supplement 1 -Source Data 1/beta galactosidase Young untreated/image 1.JPG]

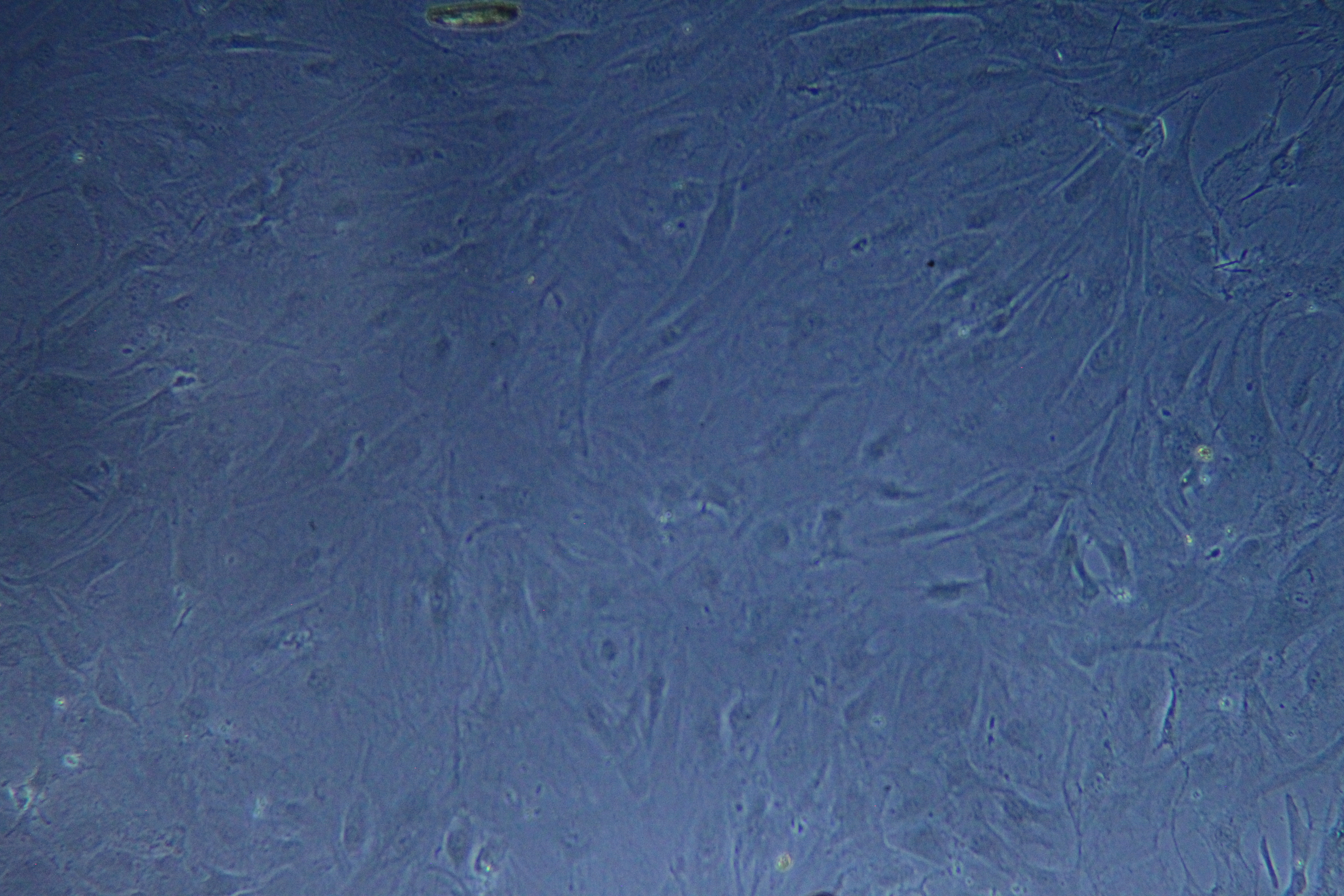

Supplement: Figure 8—figure supplement 1—source data 1. [file elife-62635-fig8-figsupp1-data1.zip › Figure 8-figure supplement 1 -Source Data 1/beta galactosidase Young untreated/image 2.JPG]

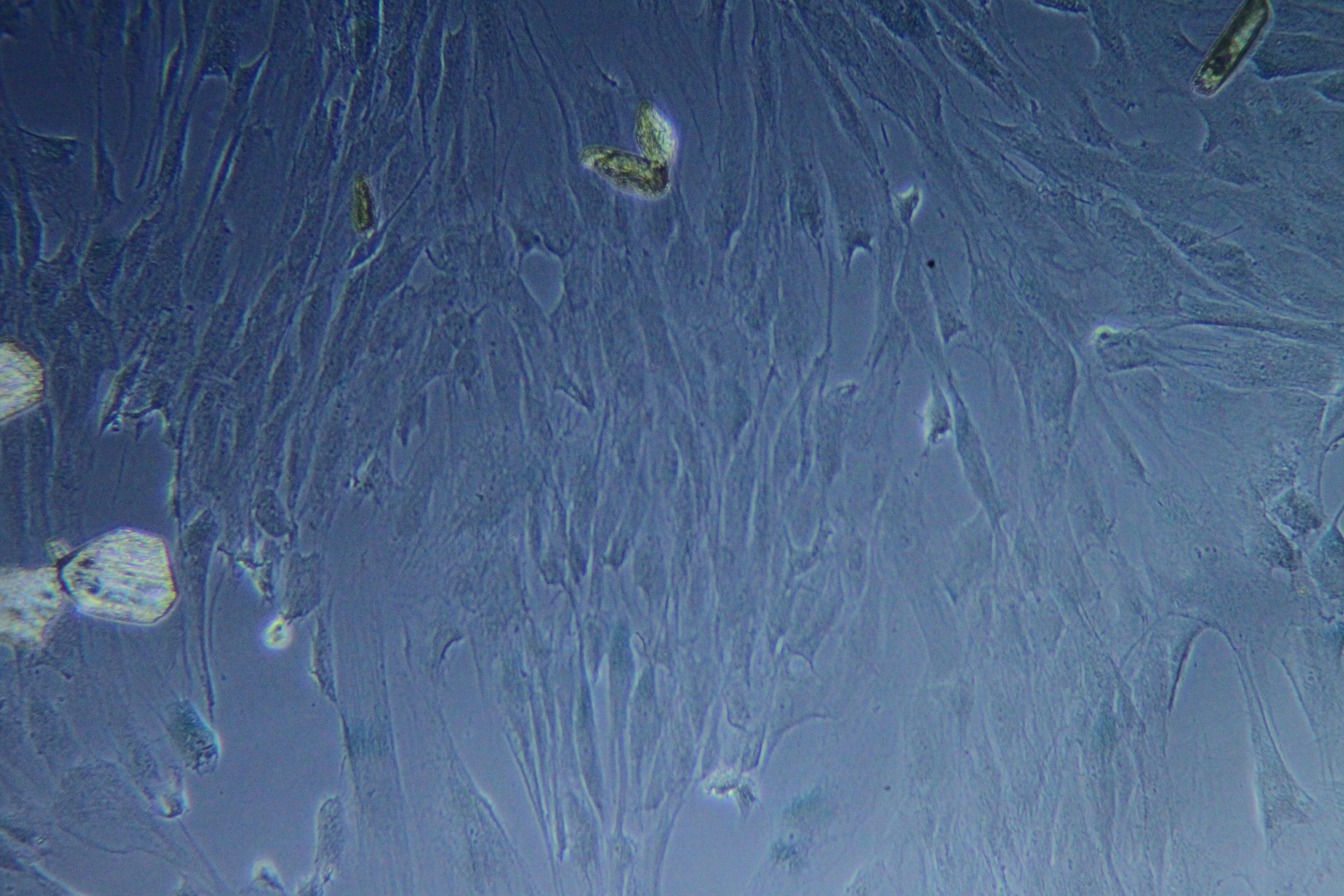

Supplement: Figure 8—figure supplement 1—source data 1. [file elife-62635-fig8-figsupp1-data1.zip › Figure 8-figure supplement 1 -Source Data 1/beta galactosidase Young untreated/image 3.JPG]

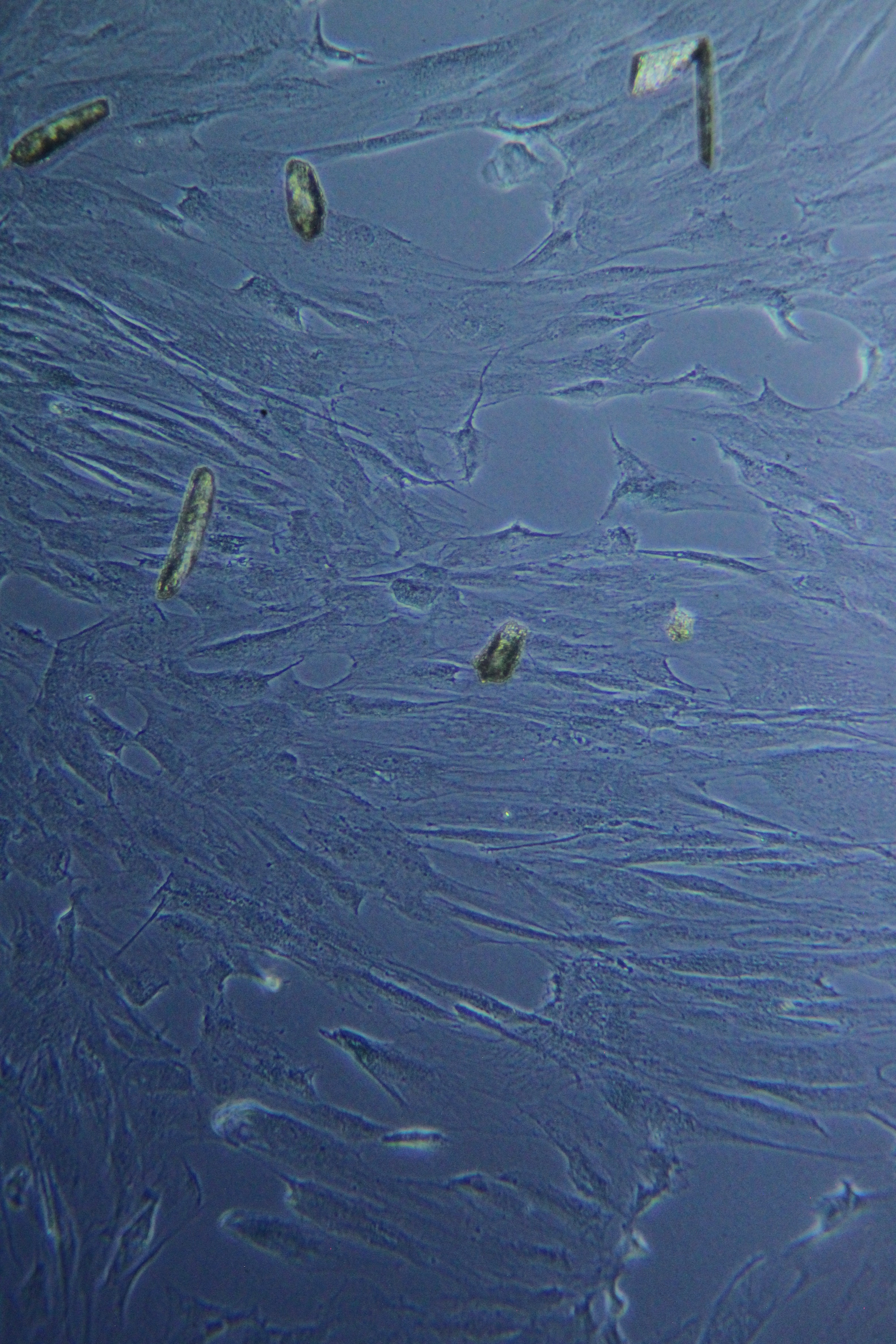

Supplement: Figure 8—figure supplement 1—source data 1. [file elife-62635-fig8-figsupp1-data1.zip › Figure 8-figure supplement 1 -Source Data 1/beta galactosidase Aged untreated/image 1 .jpg]

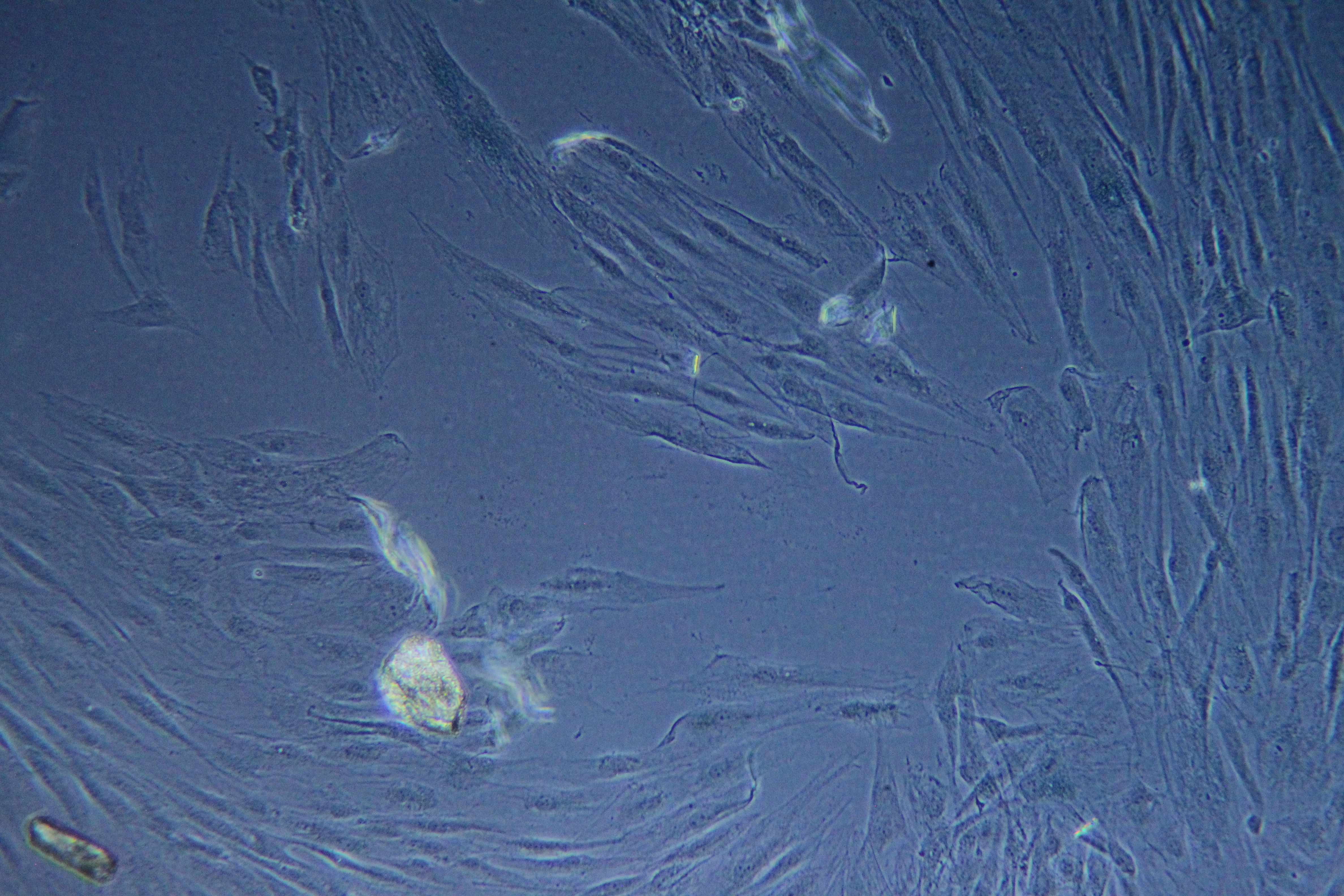

Supplement: Figure 8—figure supplement 1—source data 1. [file elife-62635-fig8-figsupp1-data1.zip › Figure 8-figure supplement 1 -Source Data 1/beta galactosidase Aged untreated/image 6.JPG]

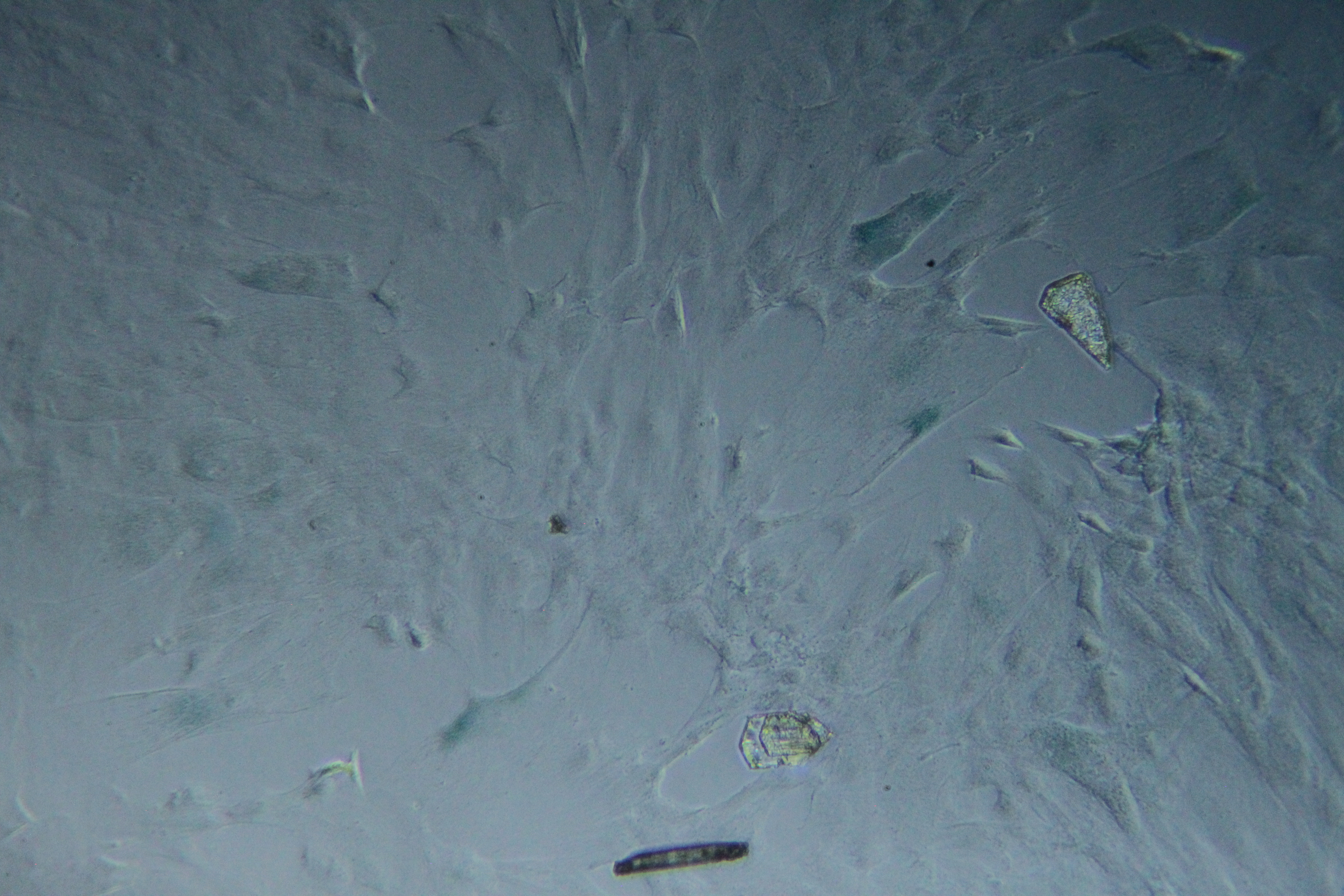

Supplement: Figure 8—figure supplement 1—source data 1. [file elife-62635-fig8-figsupp1-data1.zip › Figure 8-figure supplement 1 -Source Data 1/beta galactosidase Aged untreated/image 4.JPG]

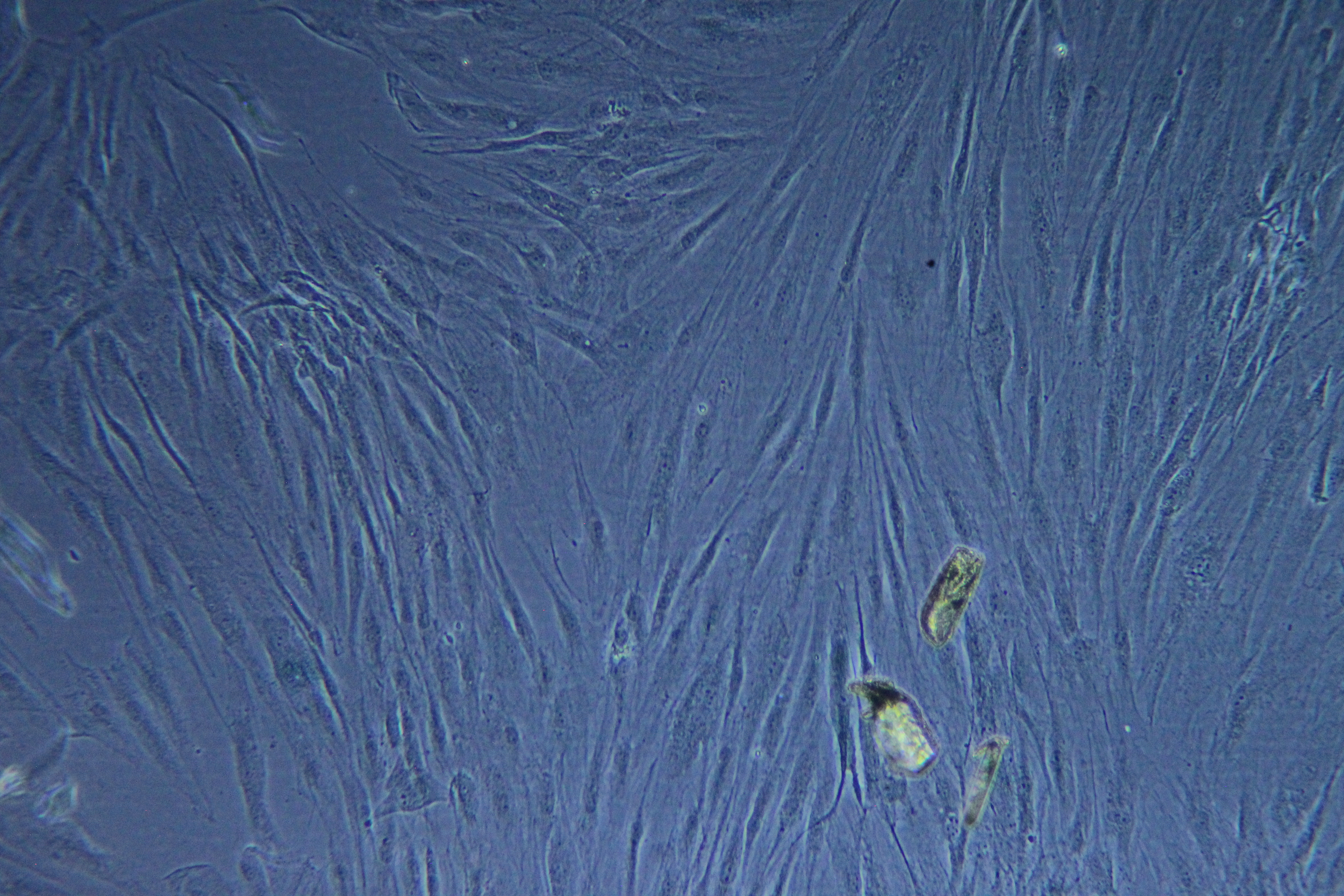

Supplement: Figure 8—figure supplement 1—source data 1. [file elife-62635-fig8-figsupp1-data1.zip › Figure 8-figure supplement 1 -Source Data 1/beta galactosidase Aged untreated/image 5.JPG]

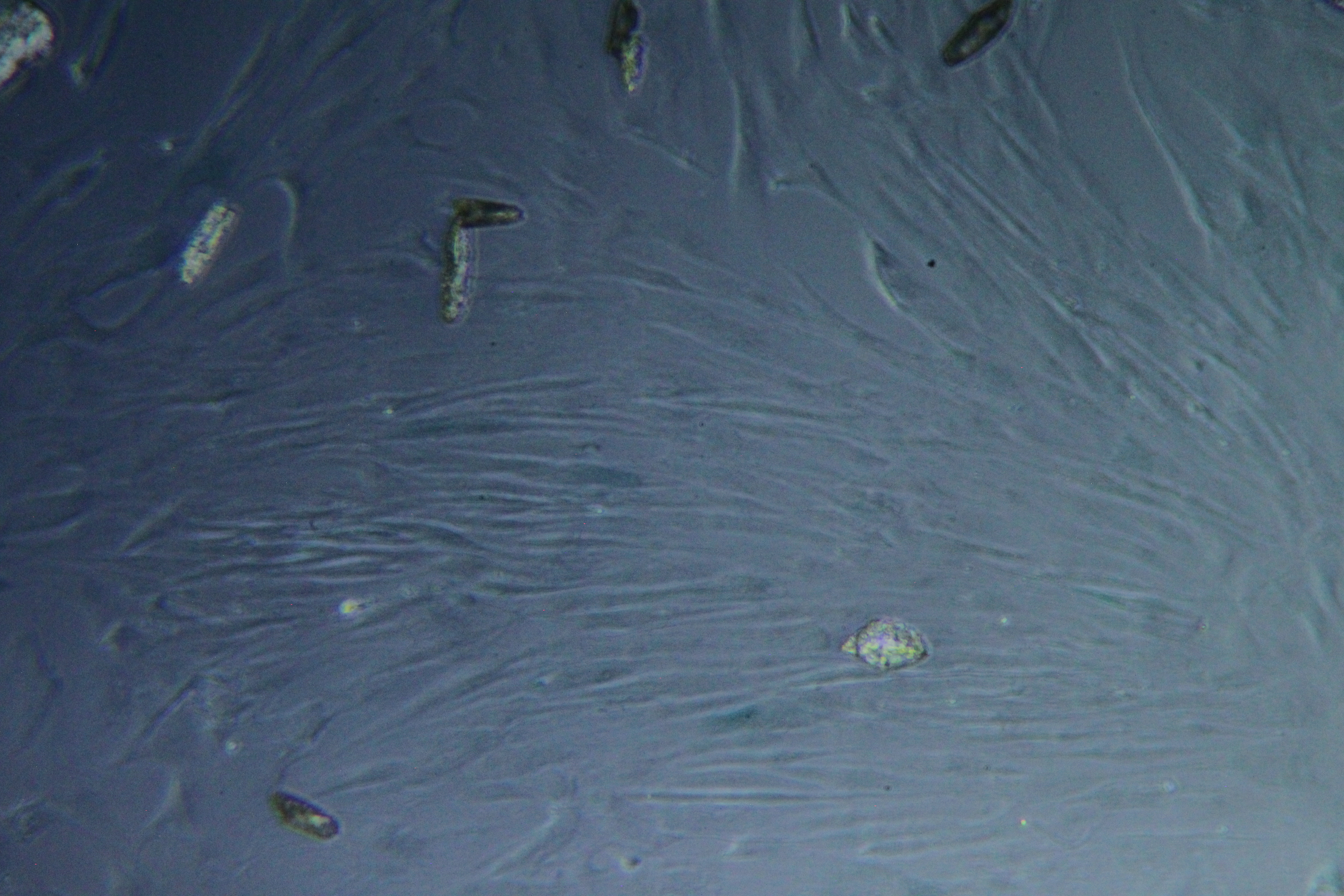

Supplement: Figure 8—figure supplement 1—source data 1. [file elife-62635-fig8-figsupp1-data1.zip › Figure 8-figure supplement 1 -Source Data 1/beta galactosidase Aged untreated/image 2.JPG]

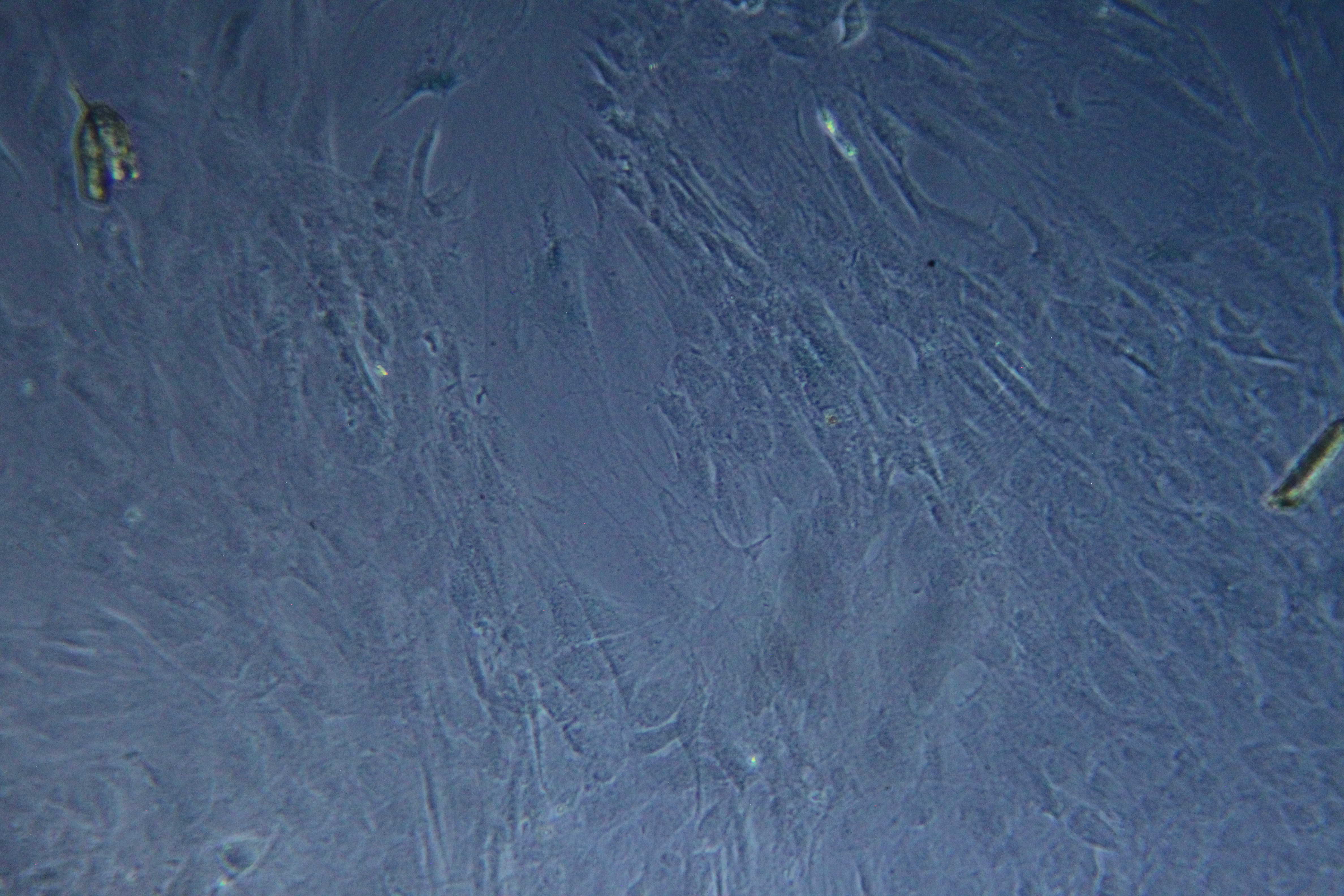

Supplement: Figure 8—figure supplement 1—source data 1. [file elife-62635-fig8-figsupp1-data1.zip › Figure 8-figure supplement 1 -Source Data 1/beta galactosidase Aged untreated/image 3.JPG]

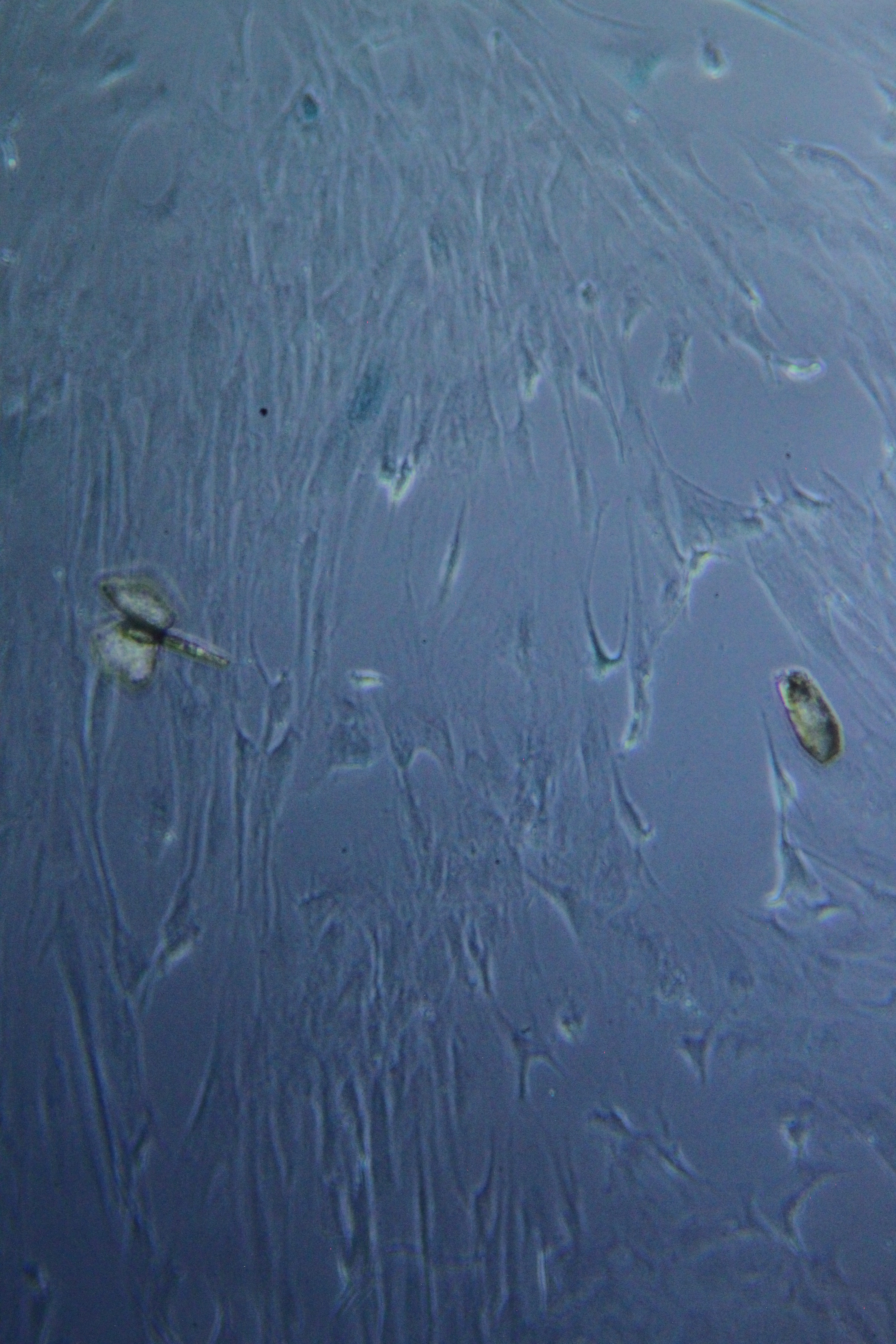

Supplement: Figure 8—figure supplement 1—source data 2. [file elife-62635-fig8-figsupp1-data2.zip › Figure 8-figure supplement 1 -Source Data 2/beta galactosidase Aged metformin/image 1 .jpg]

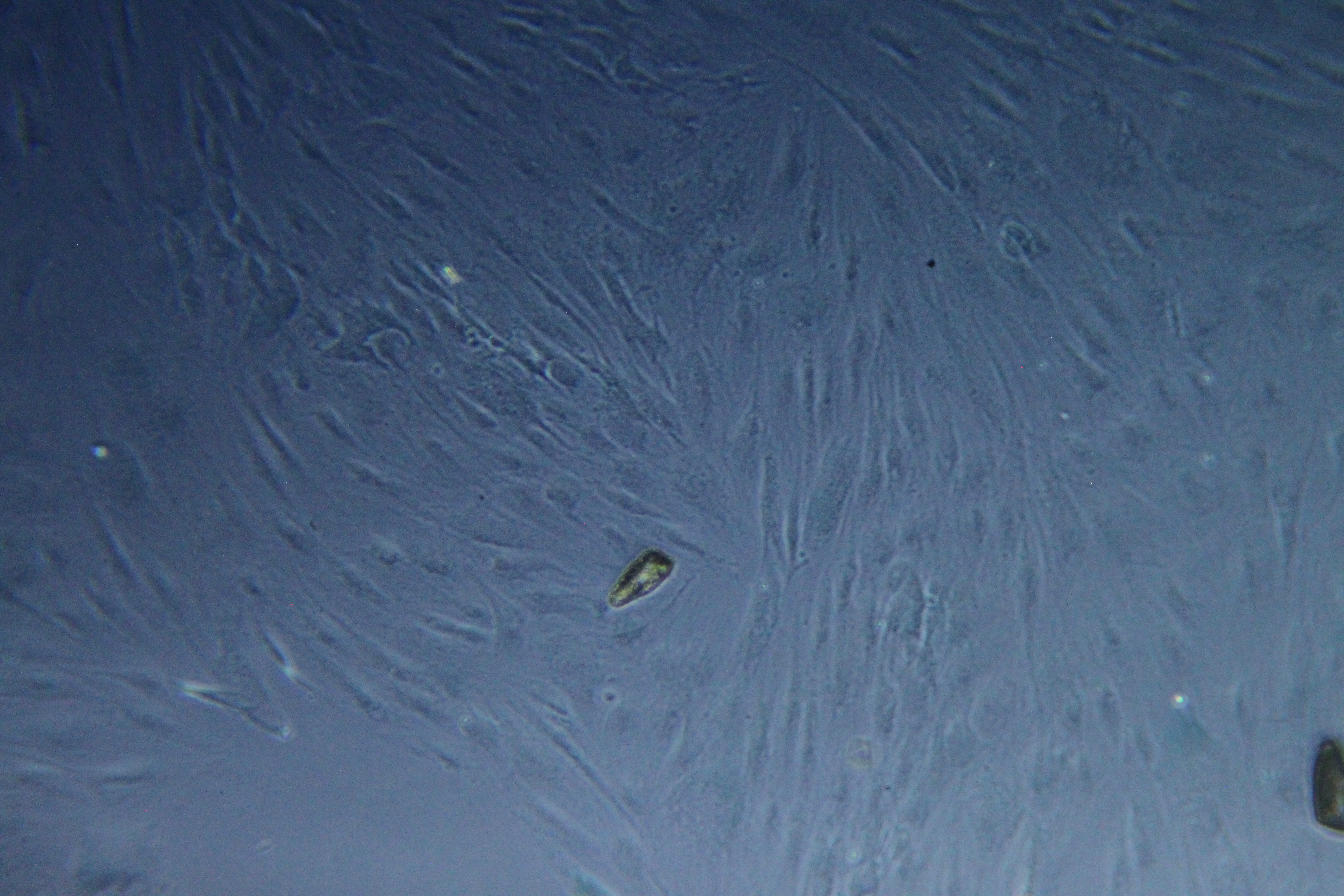

Supplement: Figure 8—figure supplement 1—source data 2. [file elife-62635-fig8-figsupp1-data2.zip › Figure 8-figure supplement 1 -Source Data 2/beta galactosidase Aged metformin/image 6.JPG]

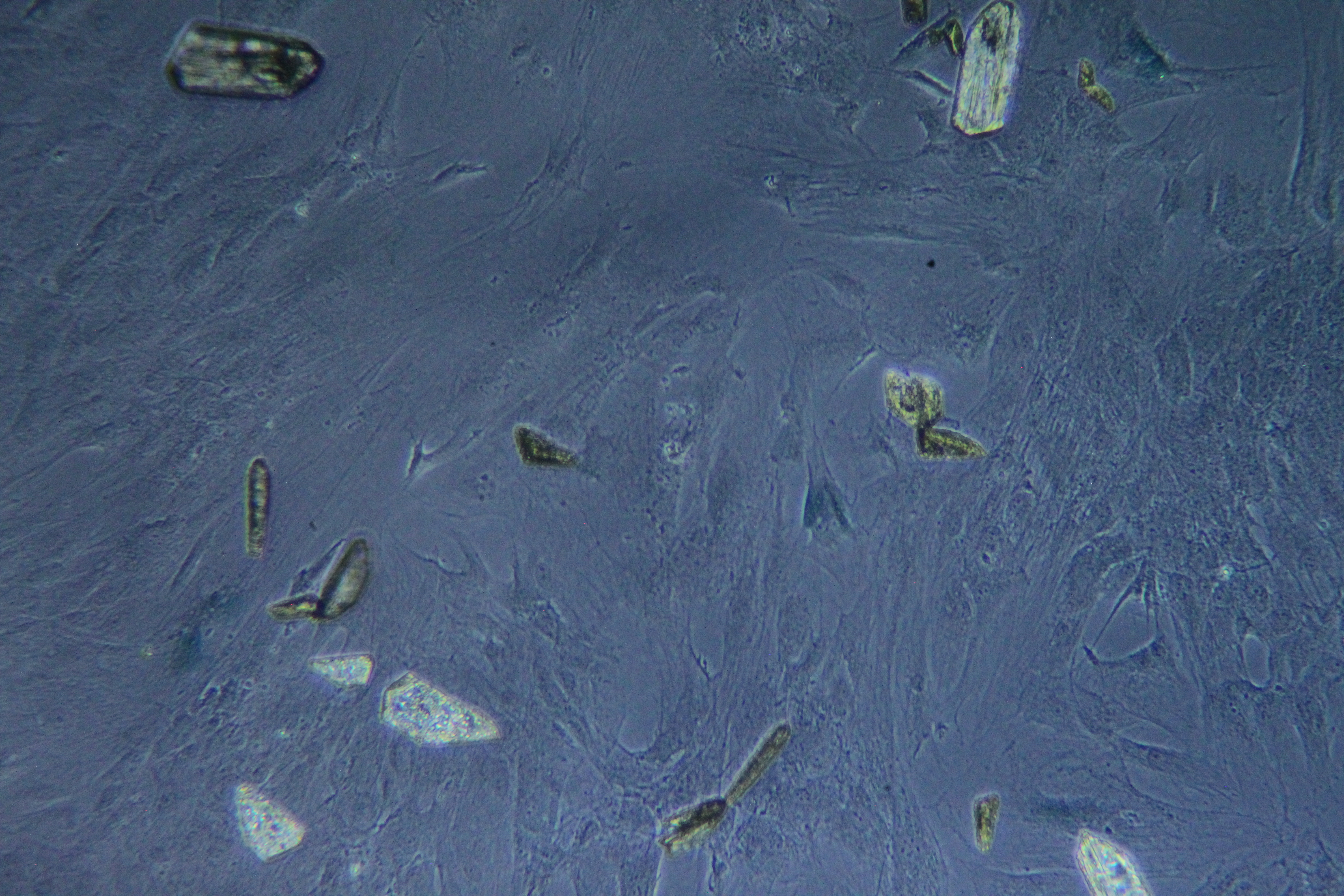

Supplement: Figure 8—figure supplement 1—source data 2. [file elife-62635-fig8-figsupp1-data2.zip › Figure 8-figure supplement 1 -Source Data 2/beta galactosidase Aged metformin/image 4.JPG]

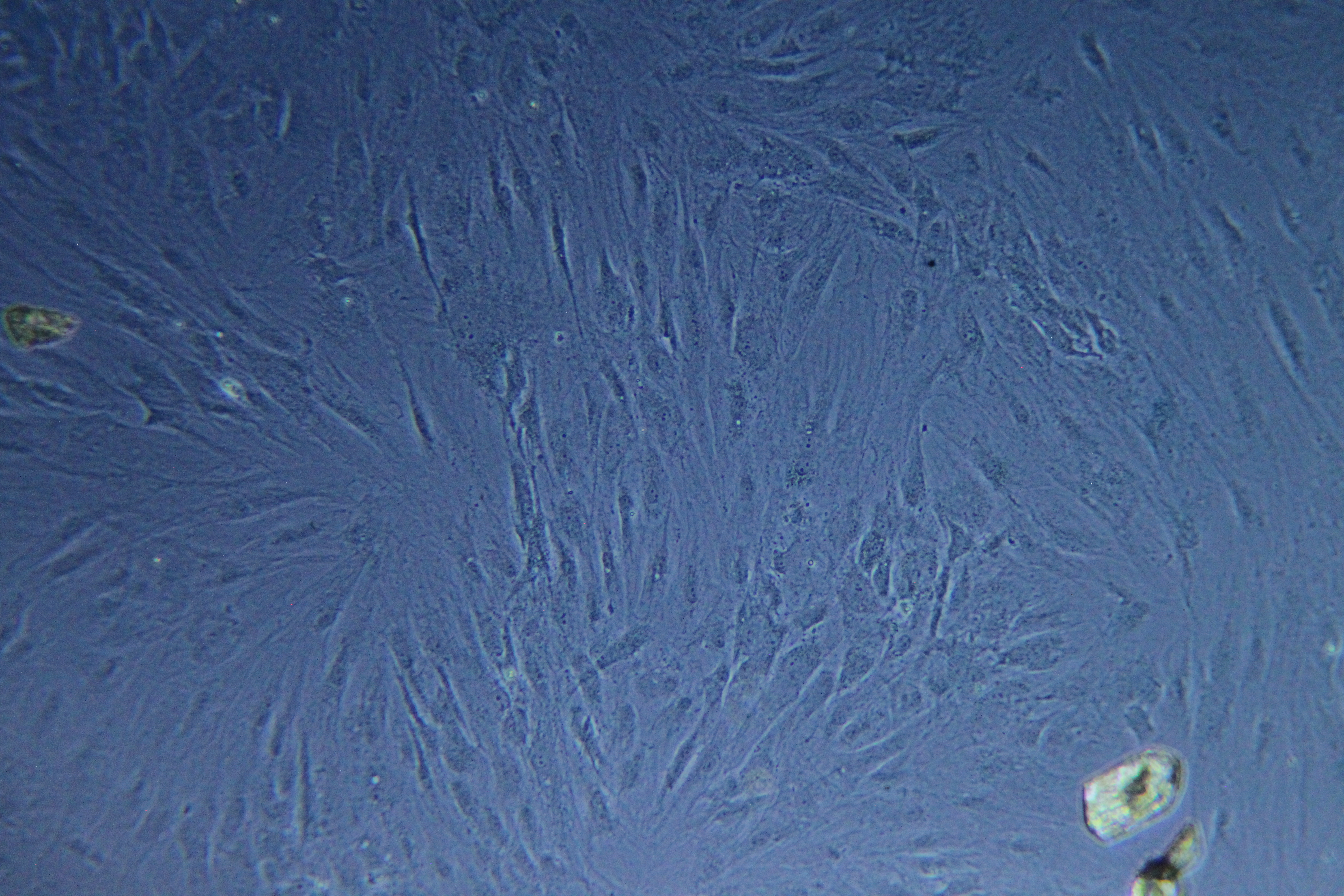

Supplement: Figure 8—figure supplement 1—source data 2. [file elife-62635-fig8-figsupp1-data2.zip › Figure 8-figure supplement 1 -Source Data 2/beta galactosidase Aged metformin/image 5.JPG]

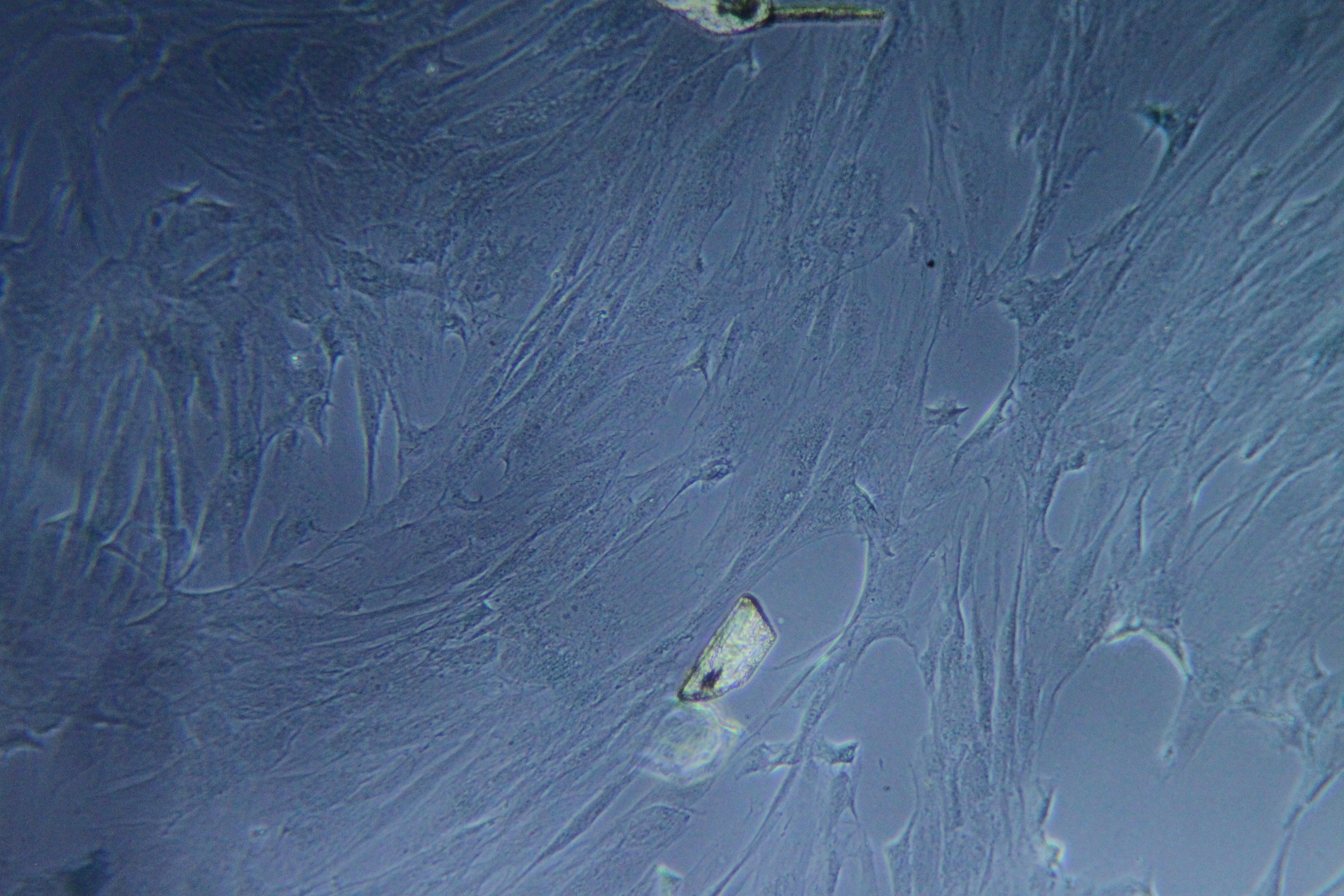

Supplement: Figure 8—figure supplement 1—source data 2. [file elife-62635-fig8-figsupp1-data2.zip › Figure 8-figure supplement 1 -Source Data 2/beta galactosidase Aged metformin/image 2.JPG]

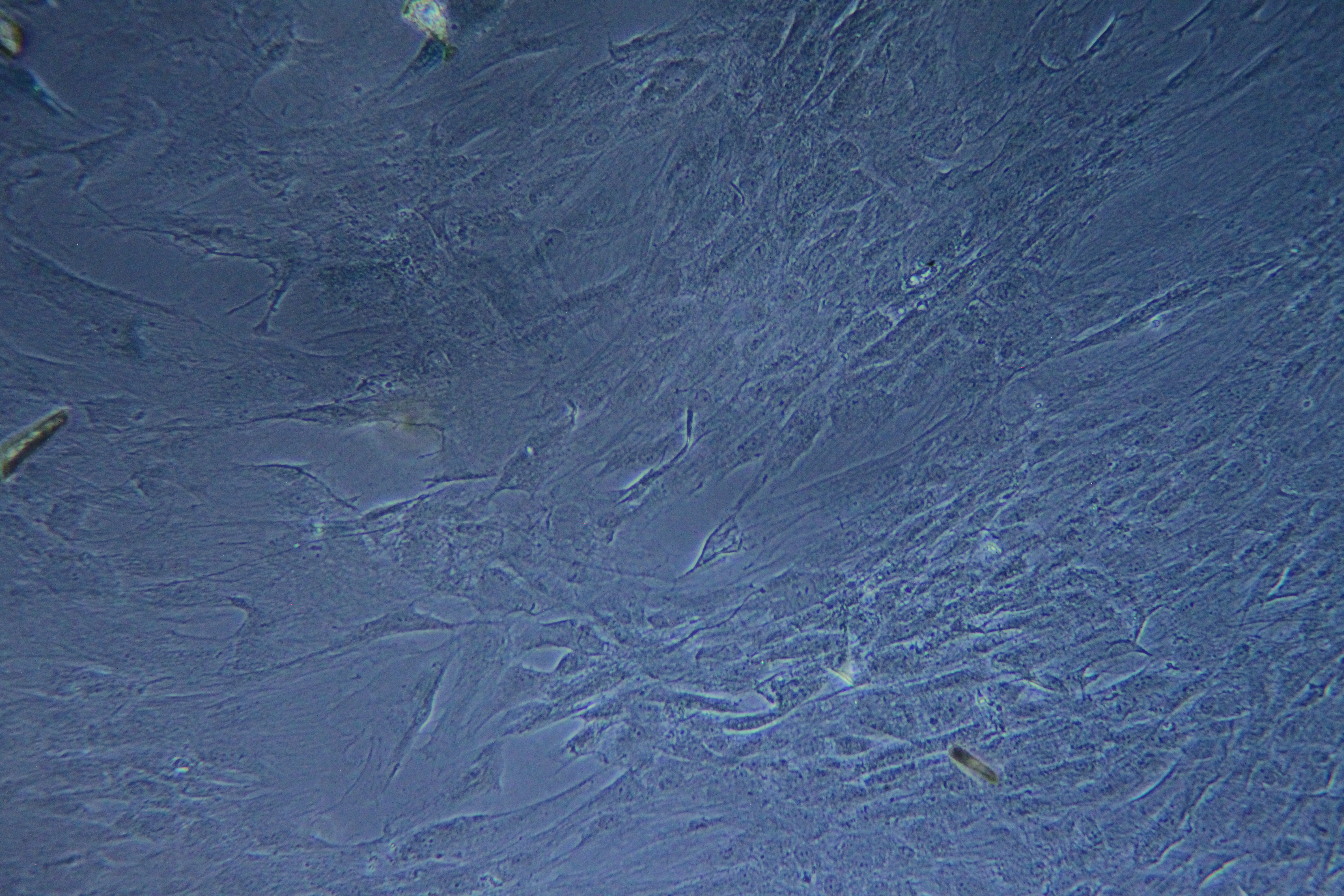

Supplement: Figure 8—figure supplement 1—source data 2. [file elife-62635-fig8-figsupp1-data2.zip › Figure 8-figure supplement 1 -Source Data 2/beta galactosidase Aged metformin/image 3.JPG]

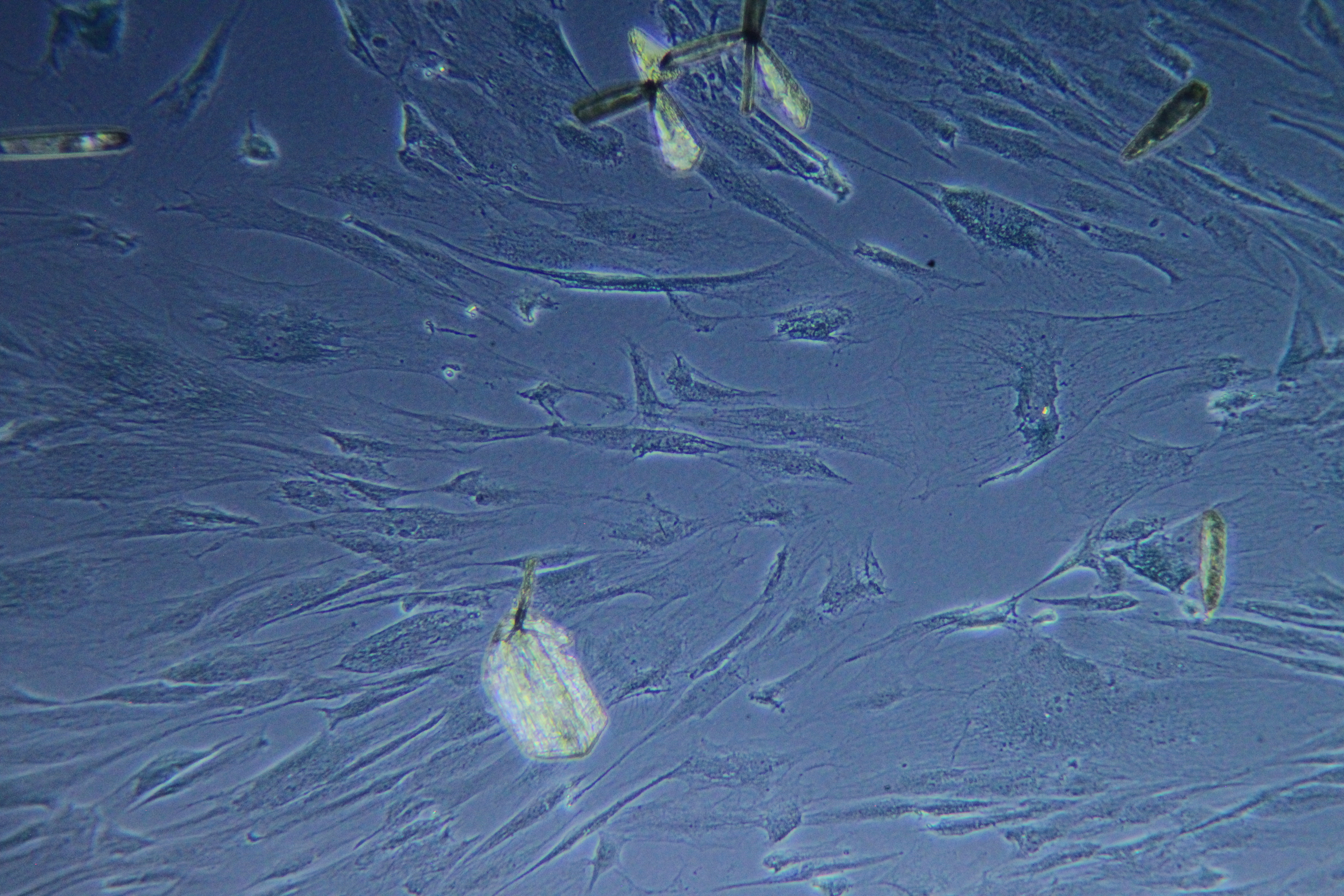

Supplement: Figure 8—figure supplement 1—source data 2. [file elife-62635-fig8-figsupp1-data2.zip › Figure 8-figure supplement 1 -Source Data 2/beta galactosidase Young metformin/image 6.JPG]

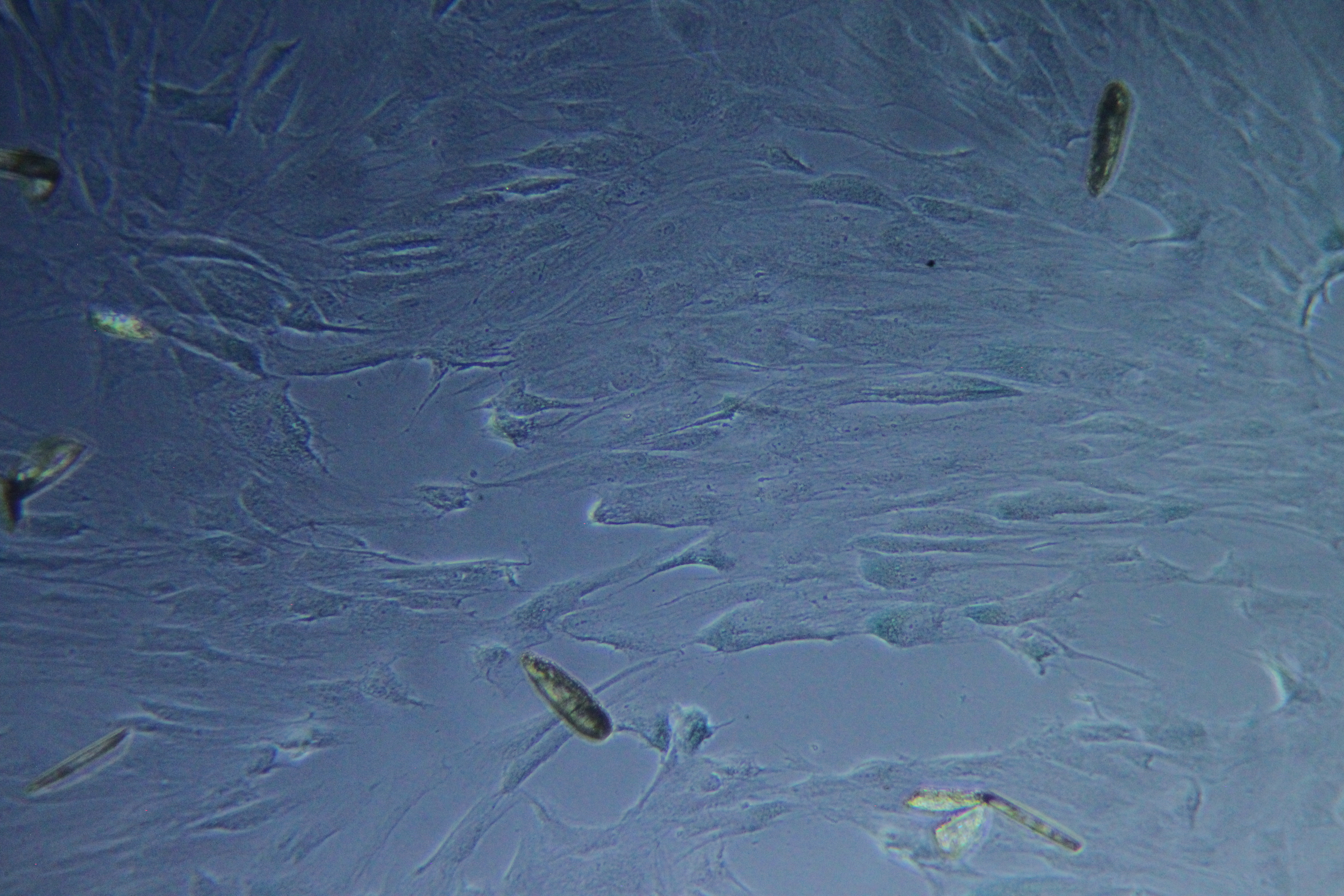

Supplement: Figure 8—figure supplement 1—source data 2. [file elife-62635-fig8-figsupp1-data2.zip › Figure 8-figure supplement 1 -Source Data 2/beta galactosidase Young metformin/image 4.JPG]

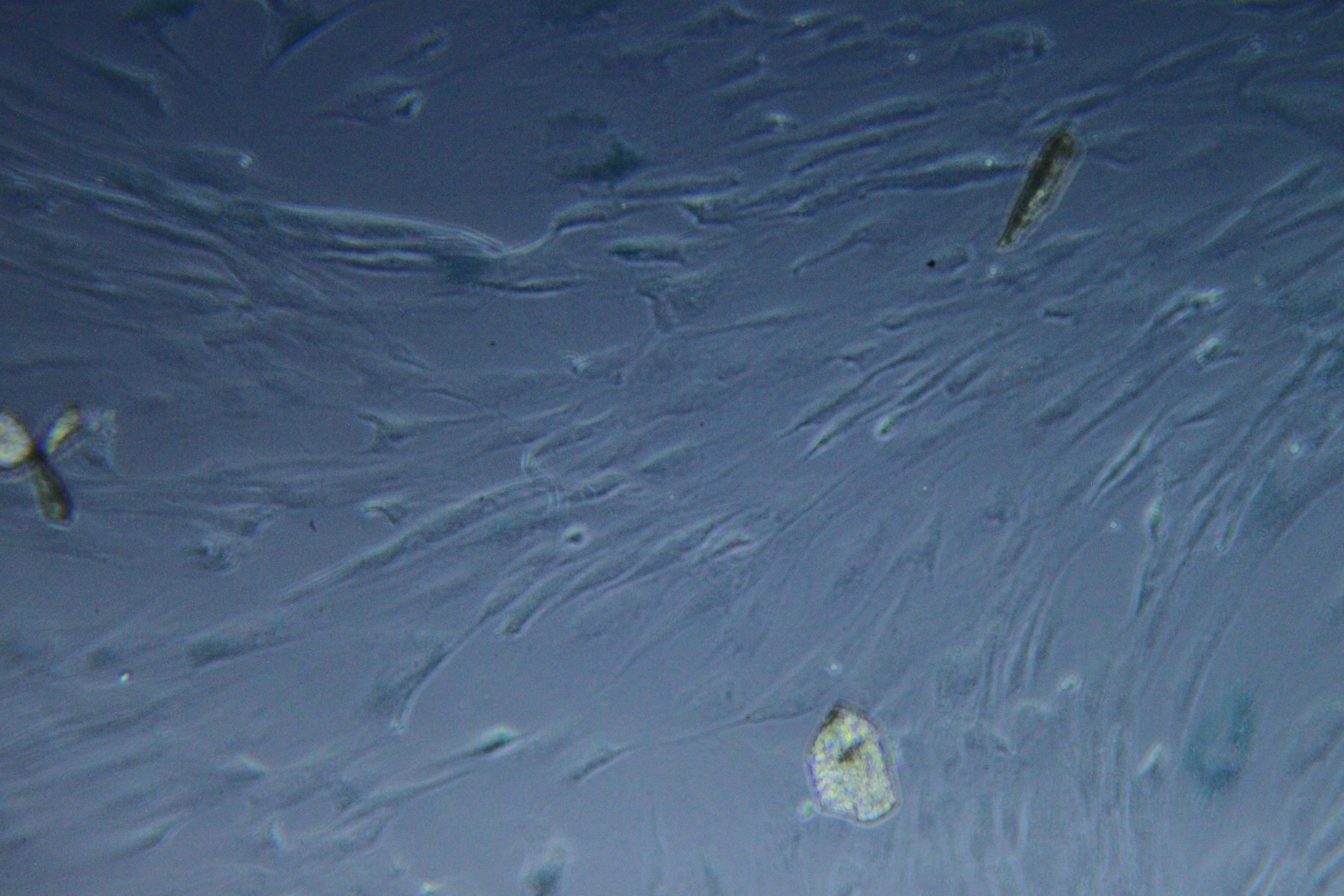

Supplement: Figure 8—figure supplement 1—source data 2. [file elife-62635-fig8-figsupp1-data2.zip › Figure 8-figure supplement 1 -Source Data 2/beta galactosidase Young metformin/image 5.JPG]

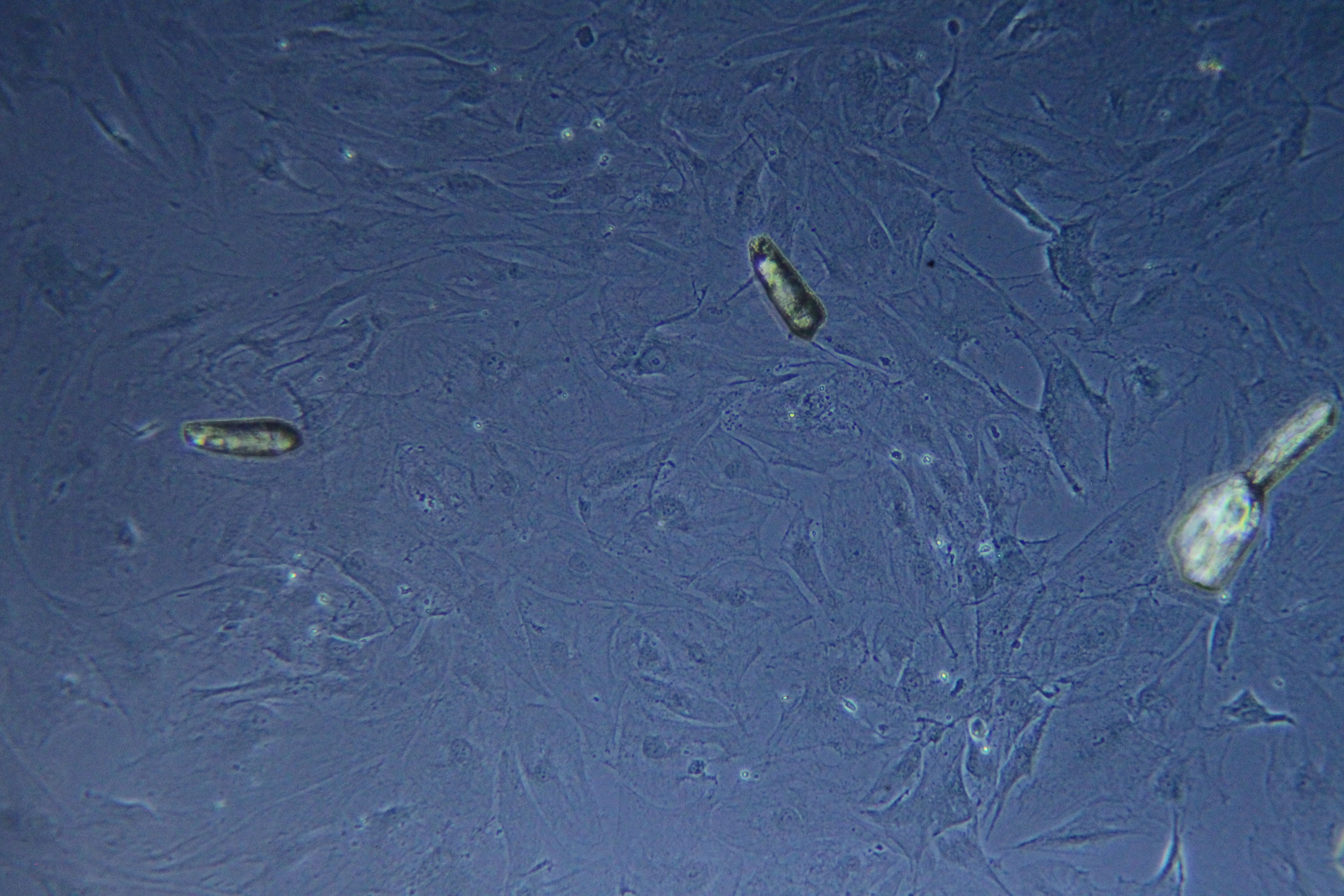

Supplement: Figure 8—figure supplement 1—source data 2. [file elife-62635-fig8-figsupp1-data2.zip › Figure 8-figure supplement 1 -Source Data 2/beta galactosidase Young metformin/image 1.JPG]

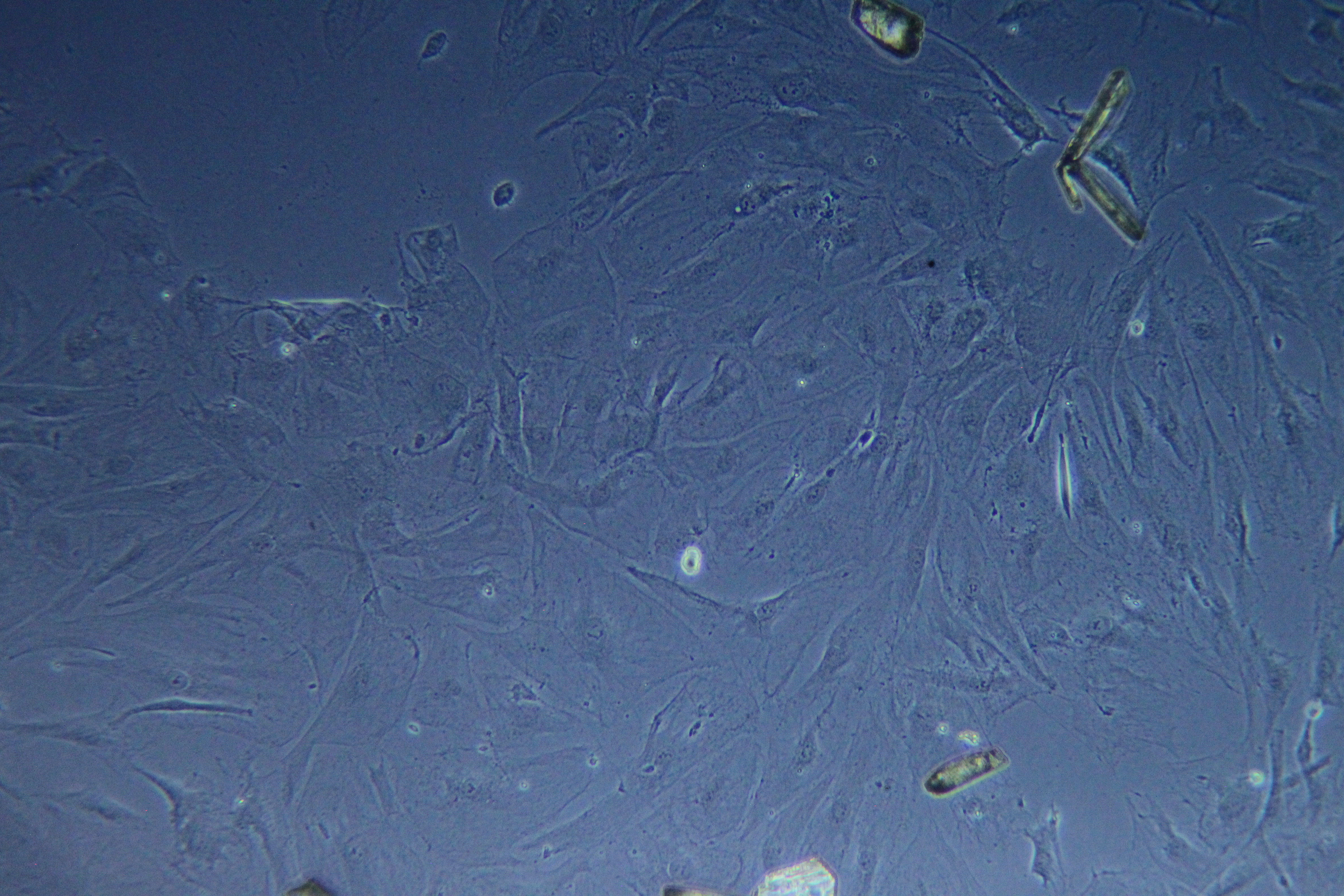

Supplement: Figure 8—figure supplement 1—source data 2. [file elife-62635-fig8-figsupp1-data2.zip › Figure 8-figure supplement 1 -Source Data 2/beta galactosidase Young metformin/image 2.JPG]

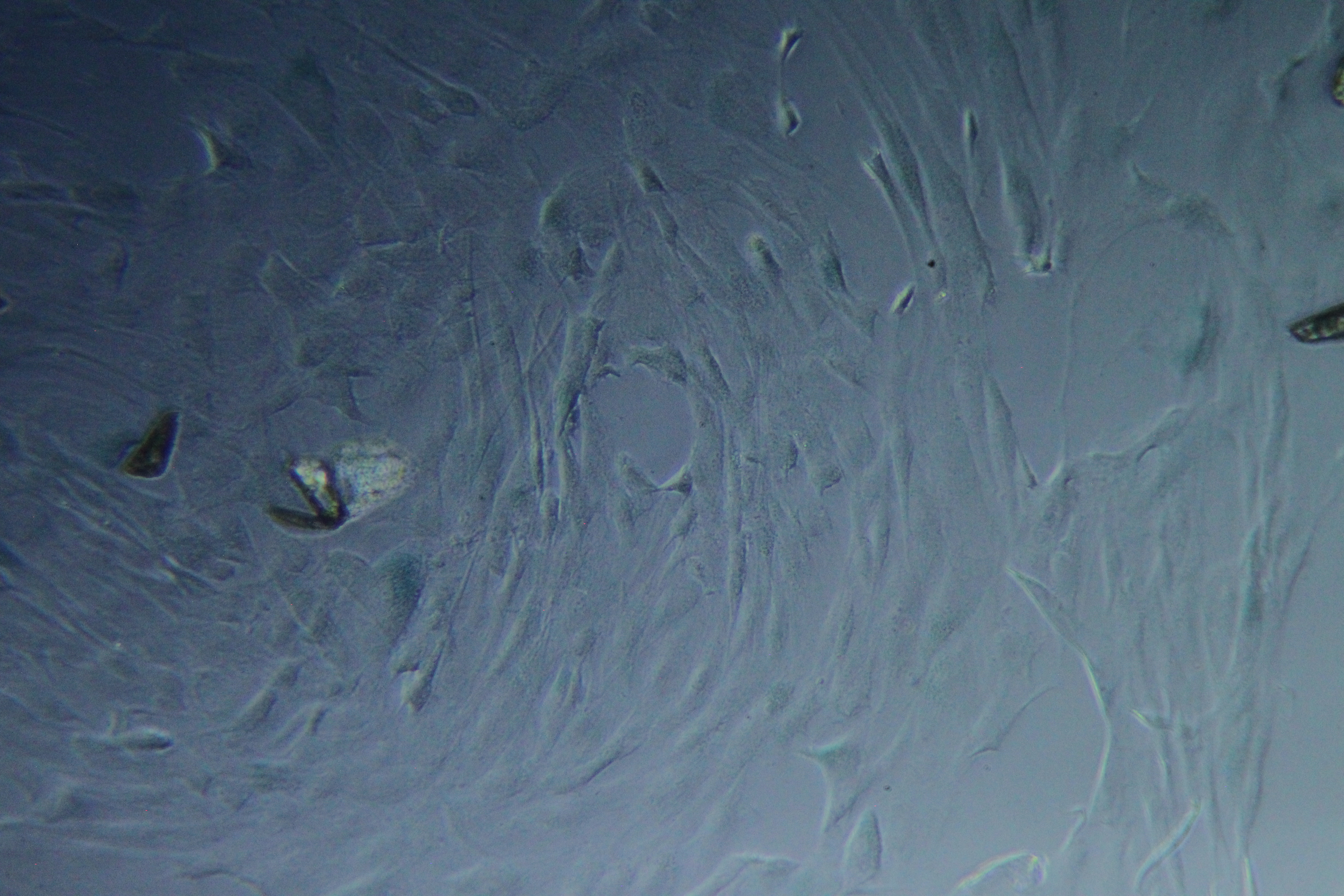

Supplement: Figure 8—figure supplement 1—source data 2. [file elife-62635-fig8-figsupp1-data2.zip › Figure 8-figure supplement 1 -Source Data 2/beta galactosidase Young metformin/image 3.JPG]

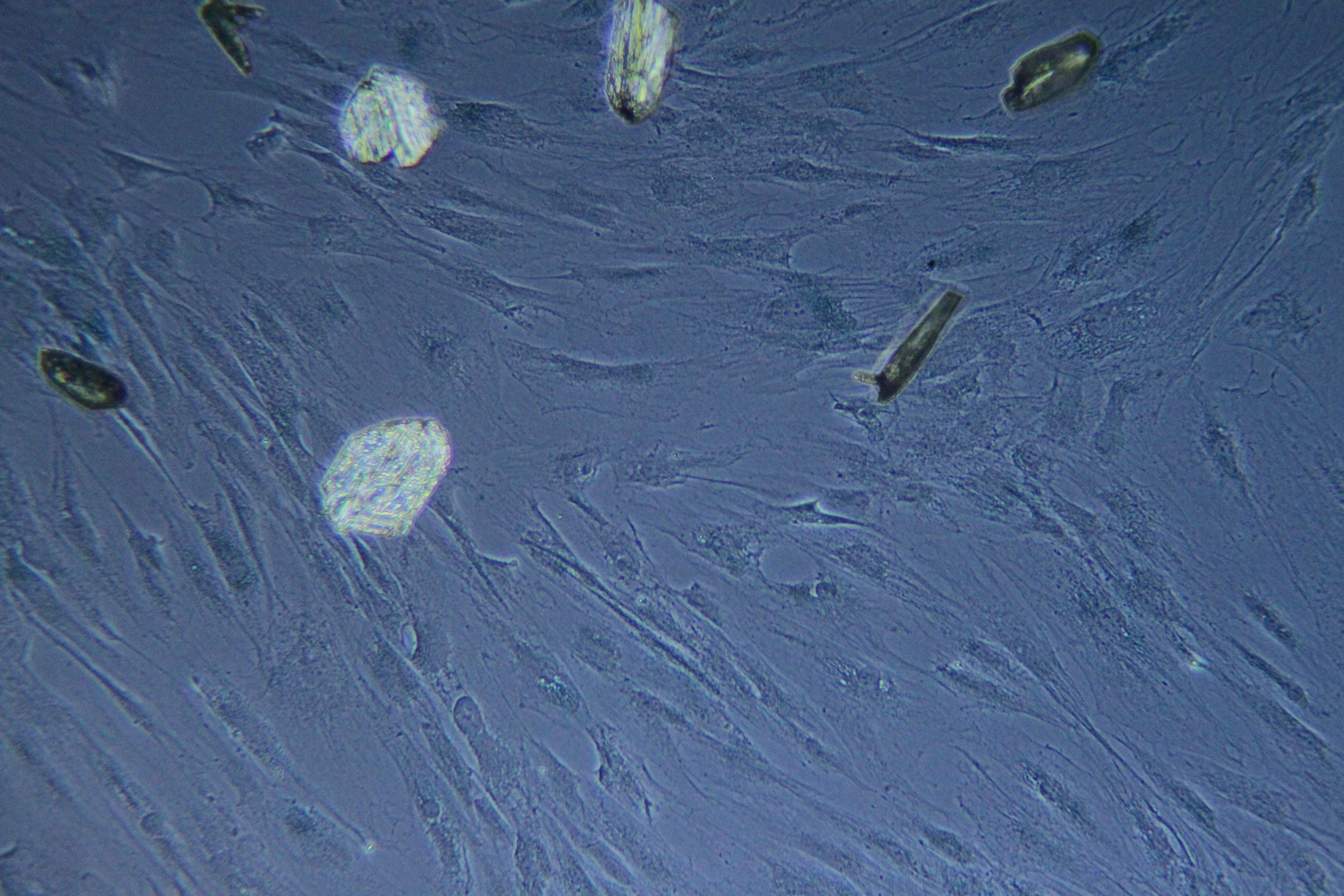

Supplement: Figure 8—figure supplement 1—source data 3. [file elife-62635-fig8-figsupp1-data3.zip › Figure 8-figure supplement 1 -Source Data 3/beta galactosidase Young AICAR/image 6.JPG]

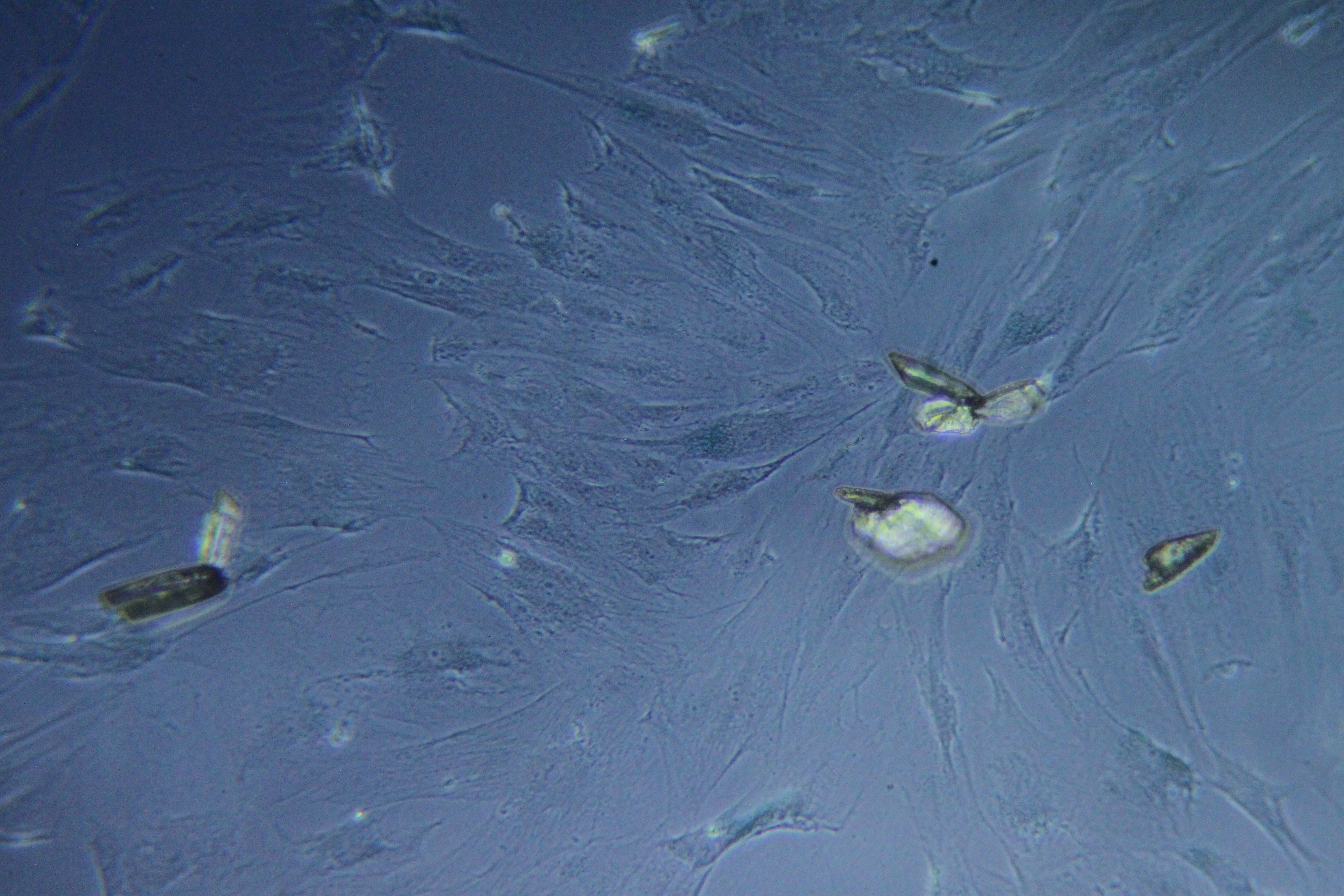

Supplement: Figure 8—figure supplement 1—source data 3. [file elife-62635-fig8-figsupp1-data3.zip › Figure 8-figure supplement 1 -Source Data 3/beta galactosidase Young AICAR/image 4.JPG]

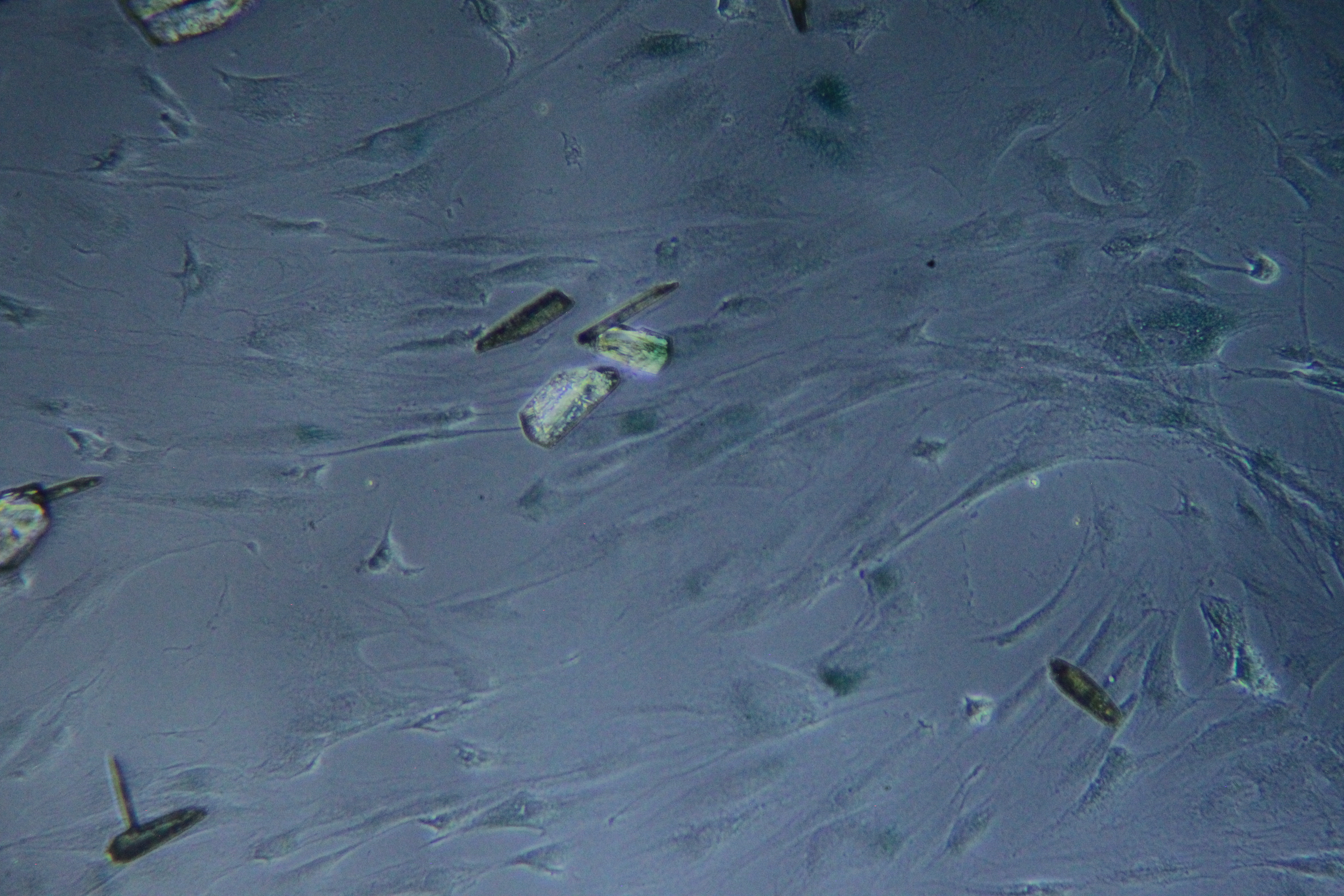

Supplement: Figure 8—figure supplement 1—source data 3. [file elife-62635-fig8-figsupp1-data3.zip › Figure 8-figure supplement 1 -Source Data 3/beta galactosidase Young AICAR/image 5.JPG]

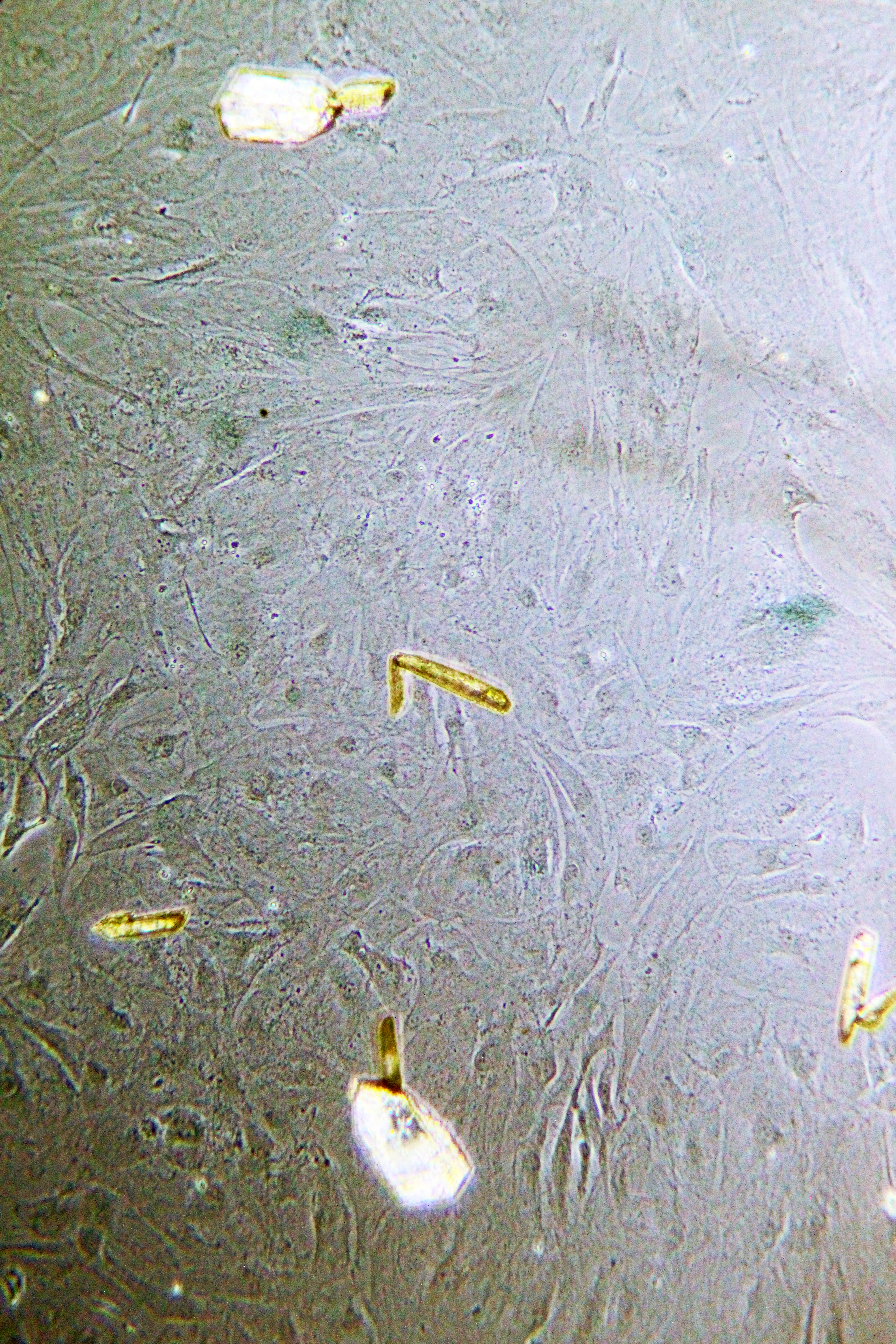

Supplement: Figure 8—figure supplement 1—source data 3. [file elife-62635-fig8-figsupp1-data3.zip › Figure 8-figure supplement 1 -Source Data 3/beta galactosidase Young AICAR/image 1.JPG]

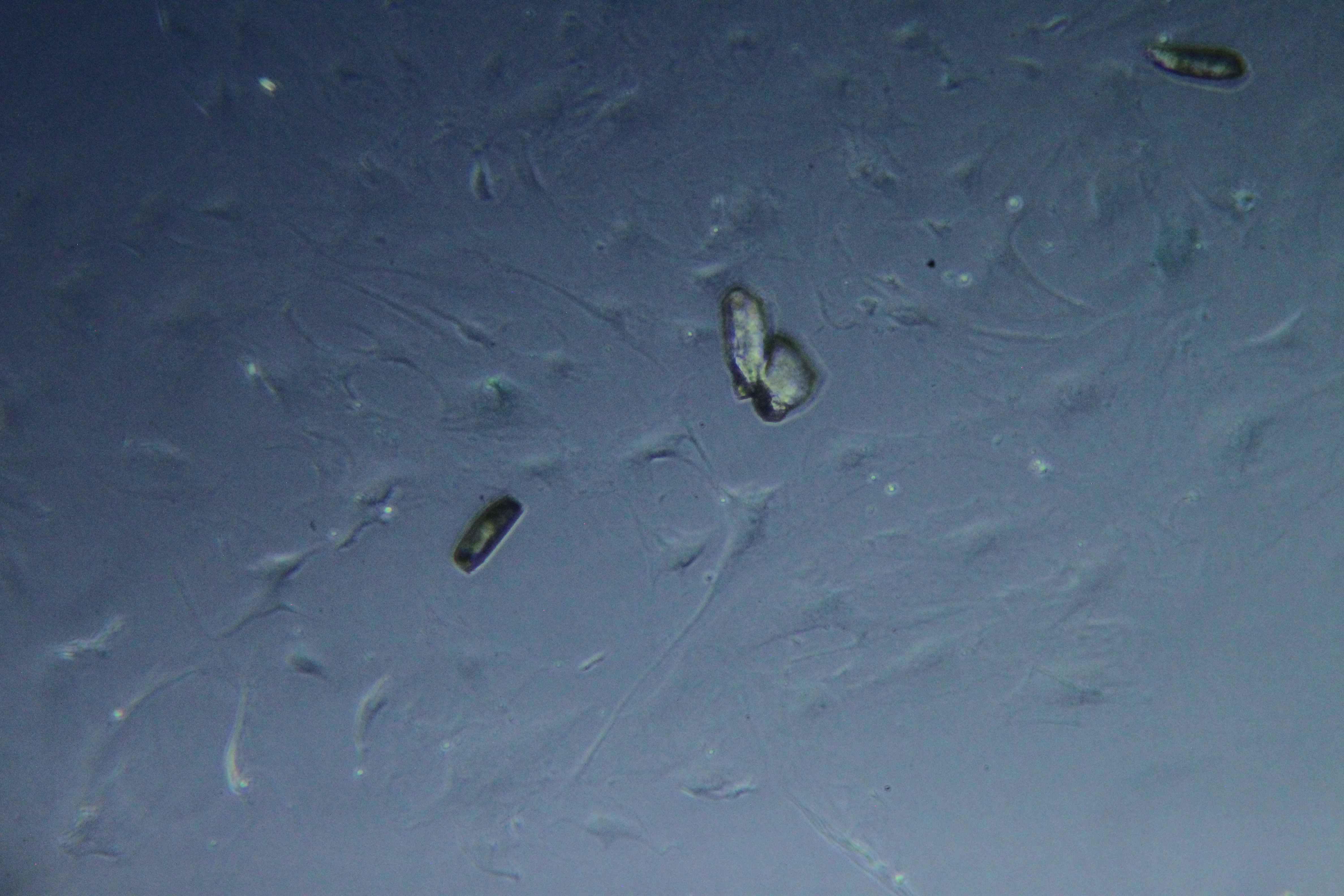

Supplement: Figure 8—figure supplement 1—source data 3. [file elife-62635-fig8-figsupp1-data3.zip › Figure 8-figure supplement 1 -Source Data 3/beta galactosidase Young AICAR/image 2.JPG]

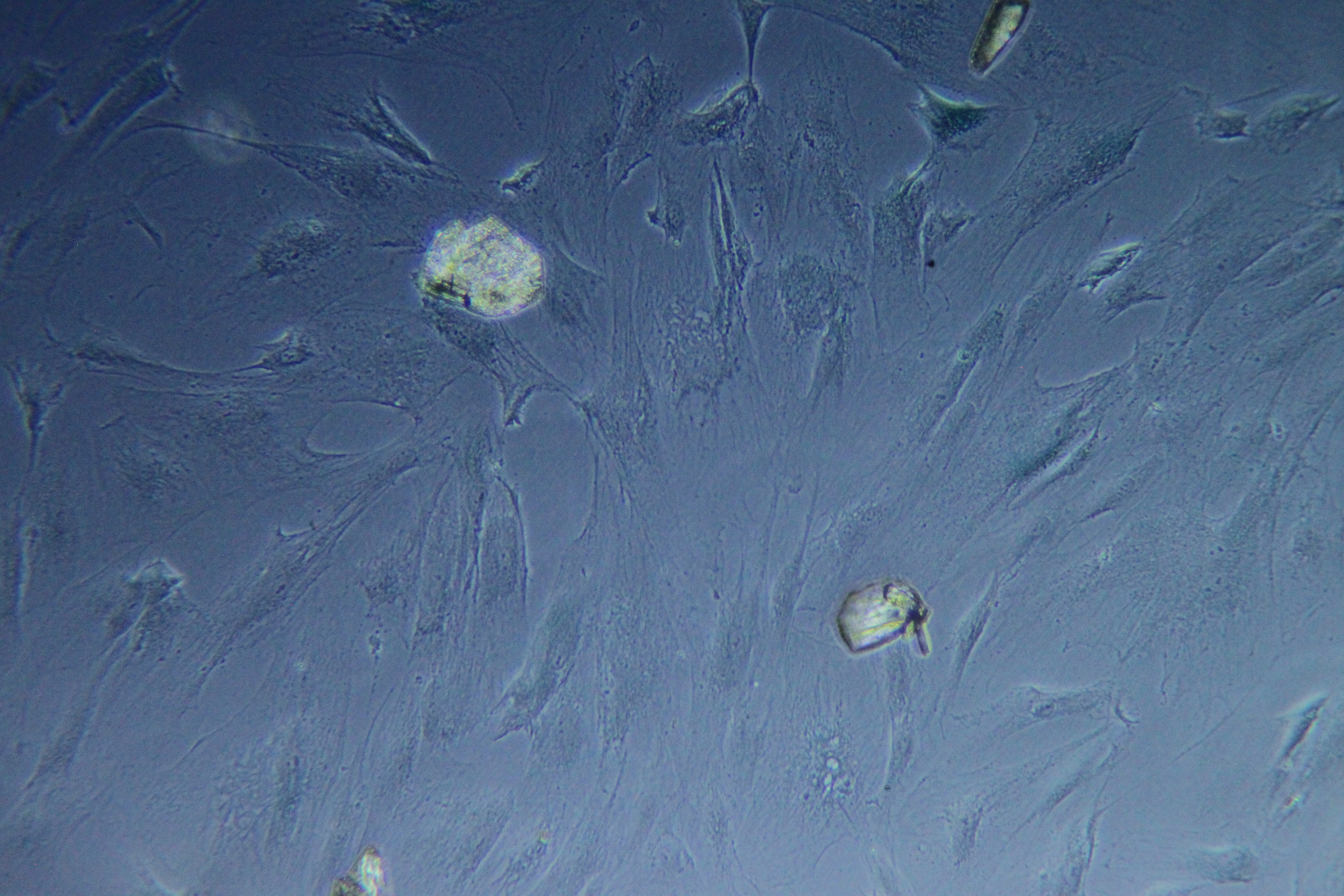

Supplement: Figure 8—figure supplement 1—source data 3. [file elife-62635-fig8-figsupp1-data3.zip › Figure 8-figure supplement 1 -Source Data 3/beta galactosidase Young AICAR/image 3.JPG]

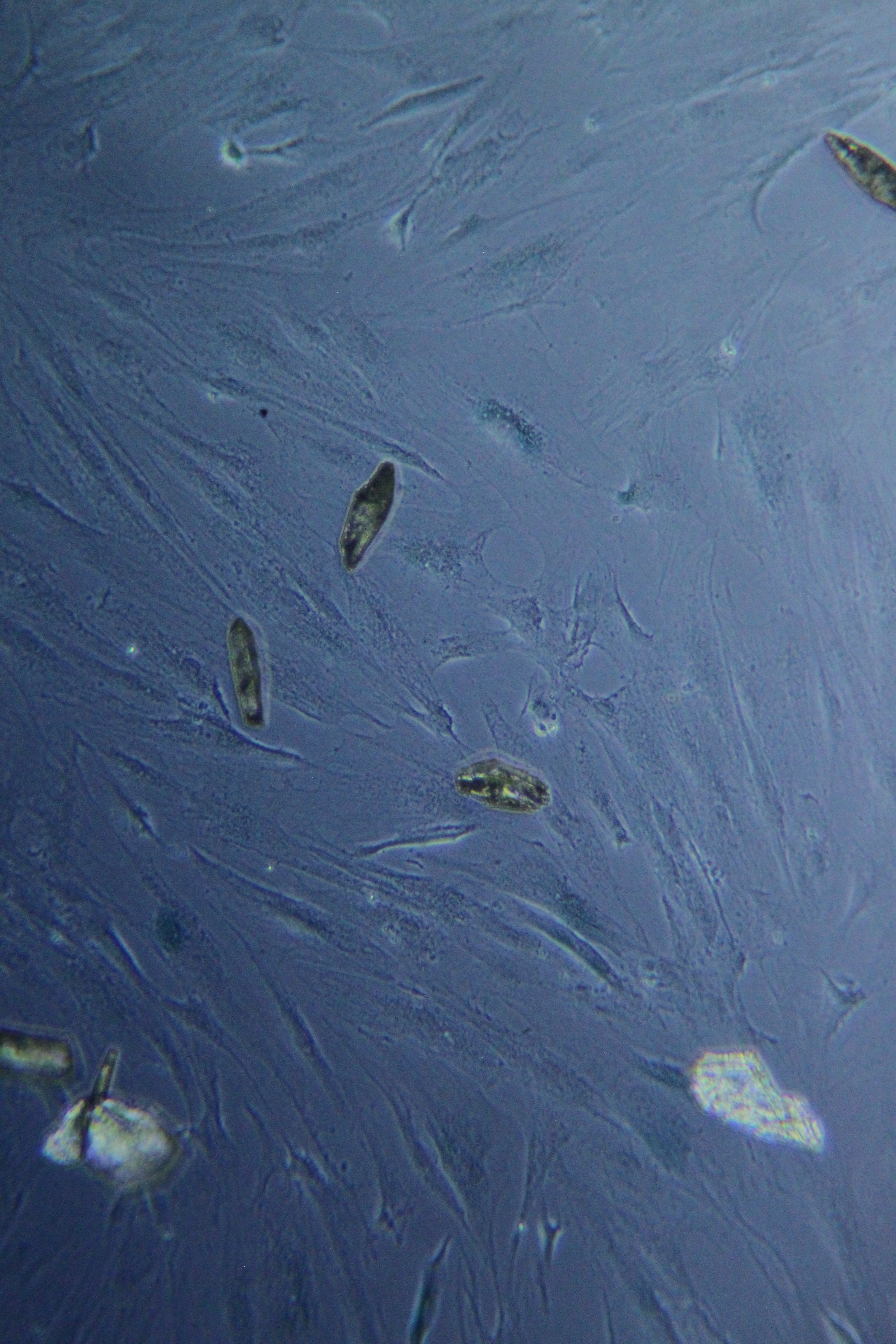

Supplement: Figure 8—figure supplement 1—source data 3. [file elife-62635-fig8-figsupp1-data3.zip › Figure 8-figure supplement 1 -Source Data 3/beta galactosidase Aged AICAR/image 1 .jpg]

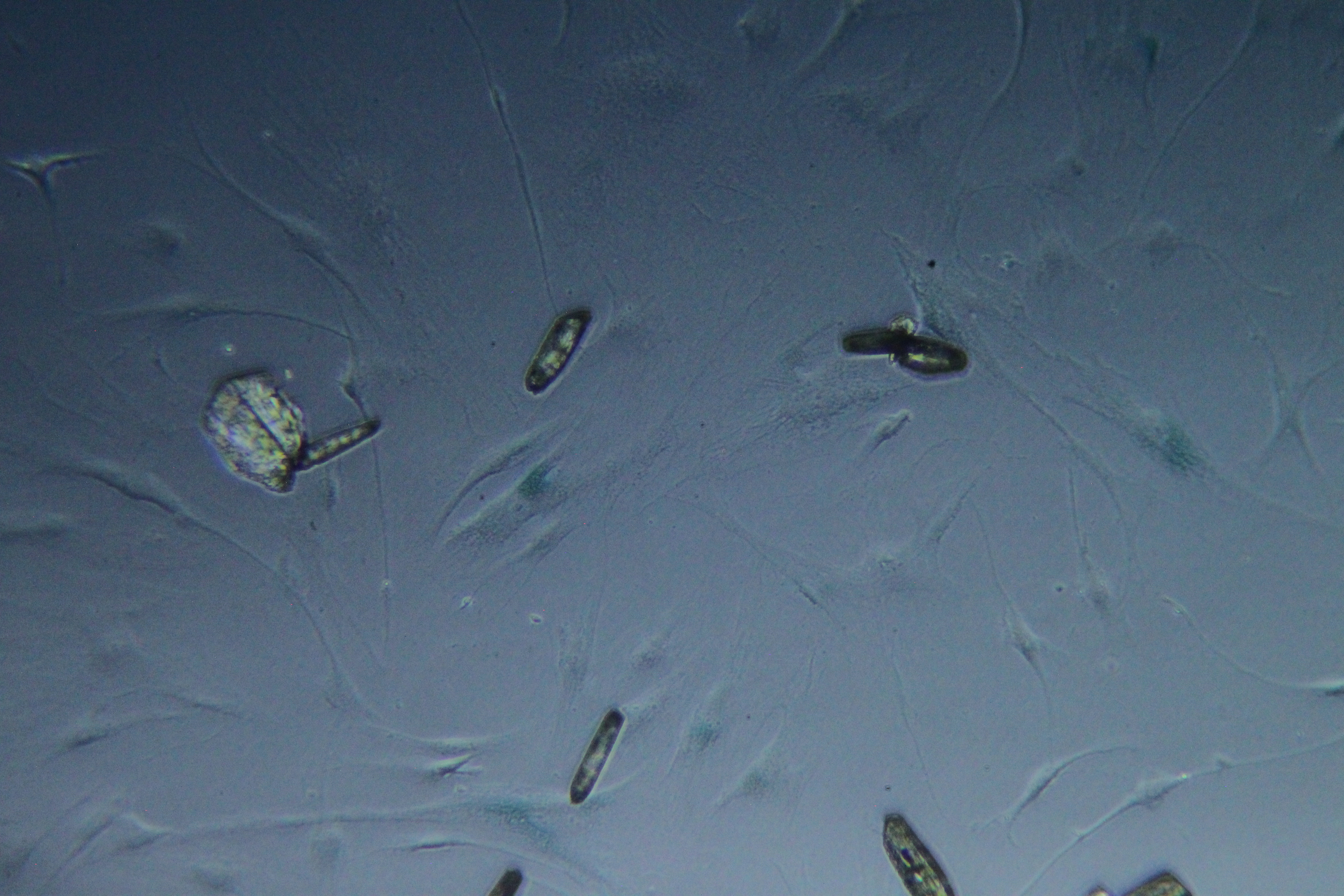

Supplement: Figure 8—figure supplement 1—source data 3. [file elife-62635-fig8-figsupp1-data3.zip › Figure 8-figure supplement 1 -Source Data 3/beta galactosidase Aged AICAR/image 6.JPG]

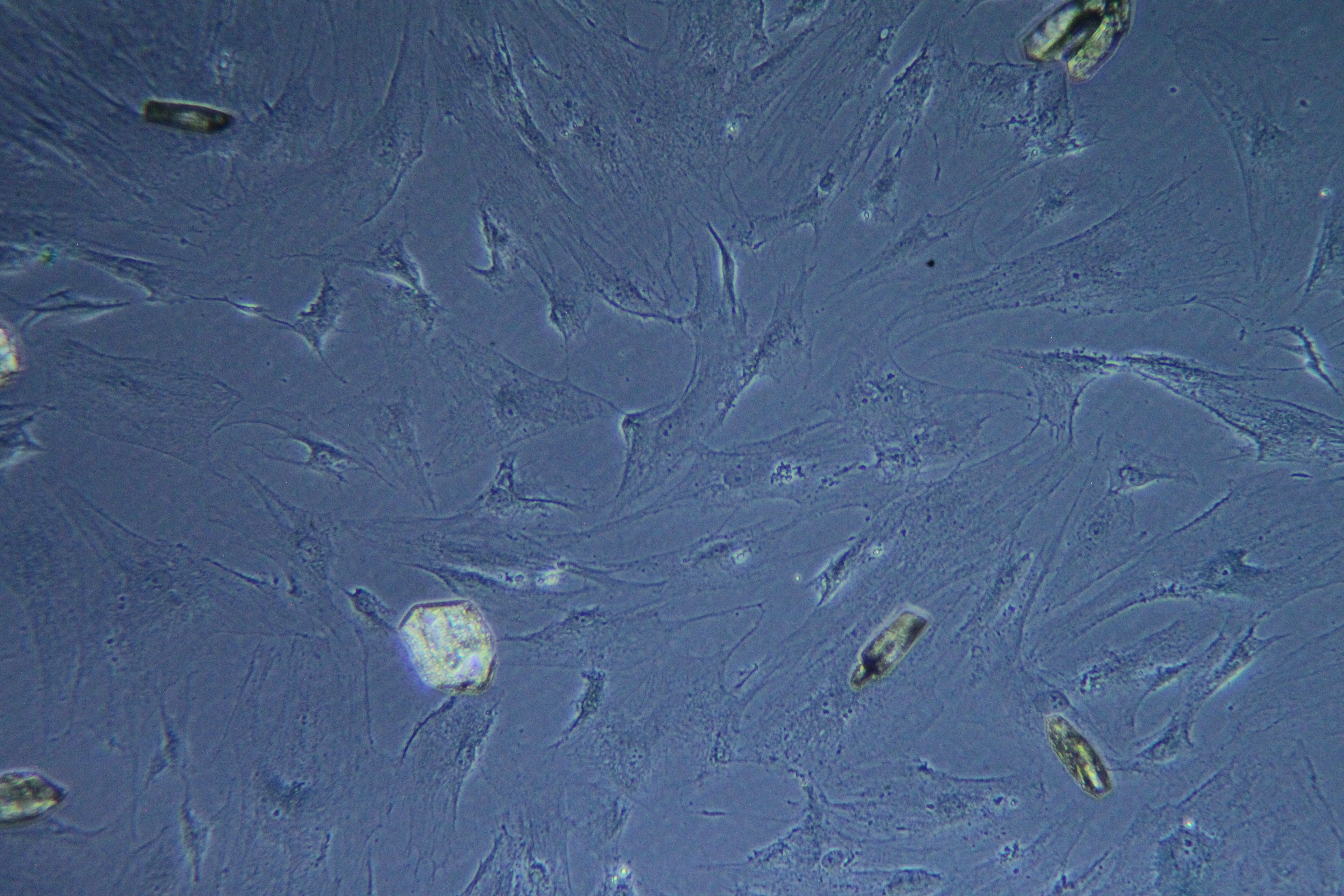

Supplement: Figure 8—figure supplement 1—source data 3. [file elife-62635-fig8-figsupp1-data3.zip › Figure 8-figure supplement 1 -Source Data 3/beta galactosidase Aged AICAR/image 4.JPG]

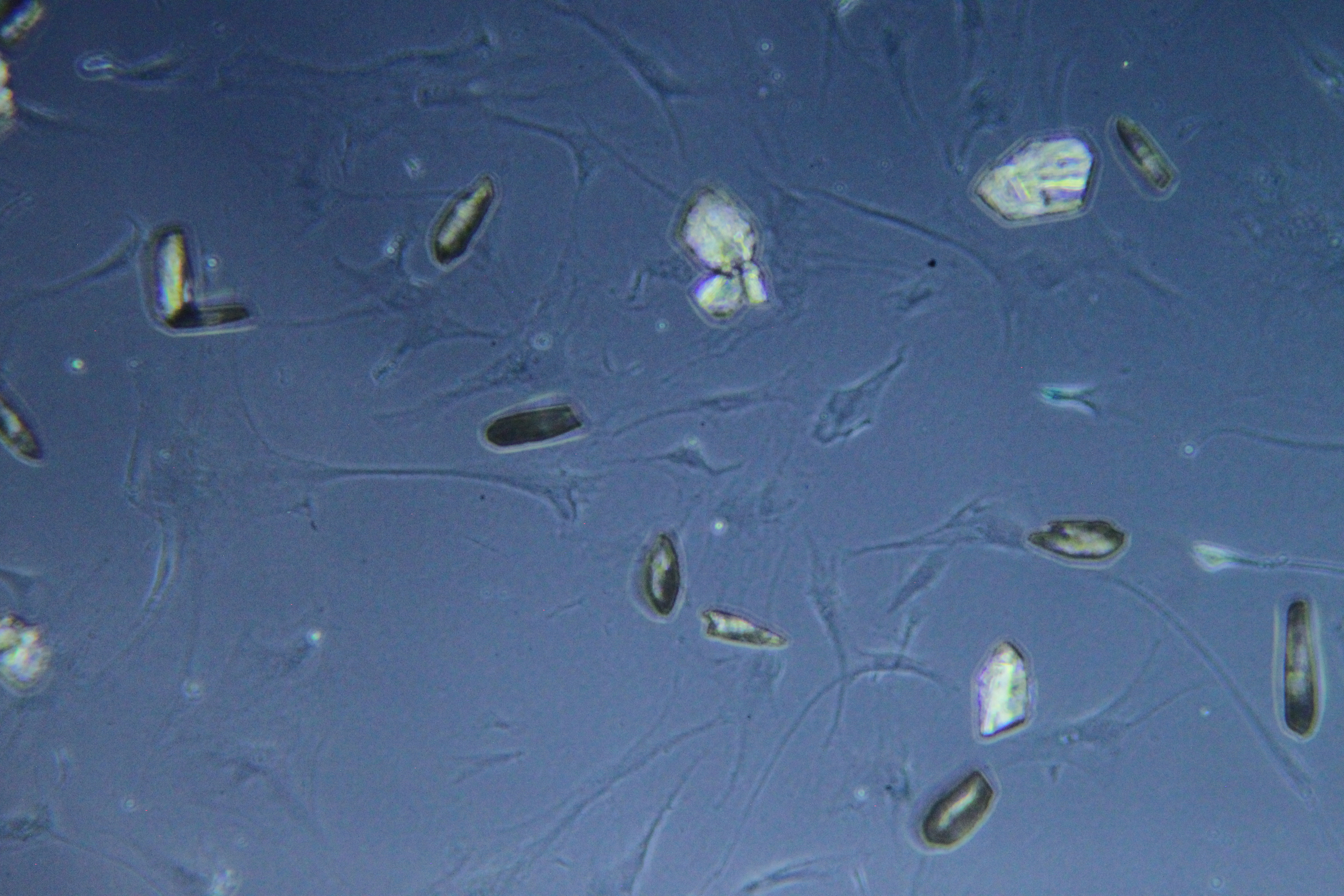

Supplement: Figure 8—figure supplement 1—source data 3. [file elife-62635-fig8-figsupp1-data3.zip › Figure 8-figure supplement 1 -Source Data 3/beta galactosidase Aged AICAR/image 5.JPG]

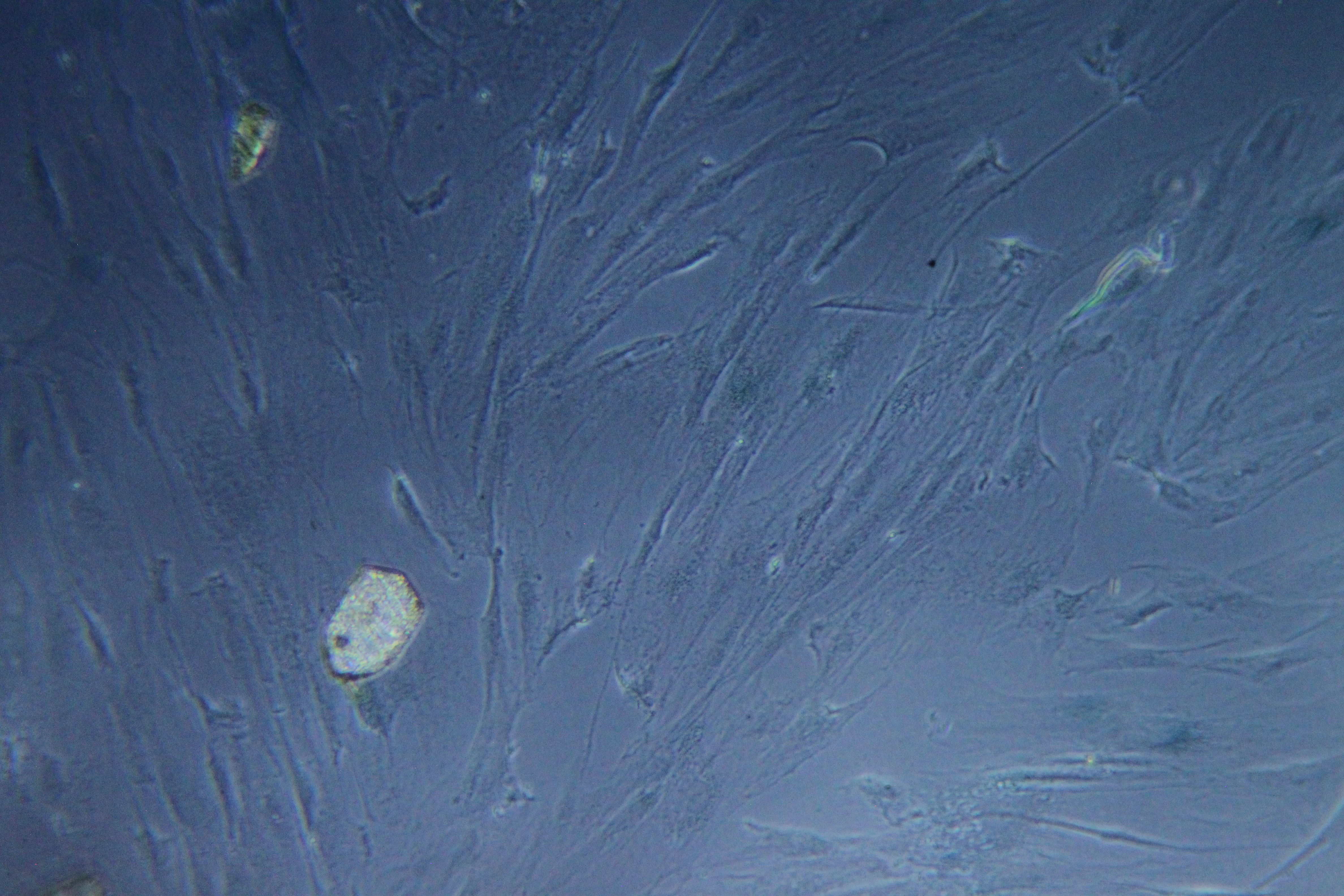

Supplement: Figure 8—figure supplement 1—source data 3. [file elife-62635-fig8-figsupp1-data3.zip › Figure 8-figure supplement 1 -Source Data 3/beta galactosidase Aged AICAR/image 2.JPG]

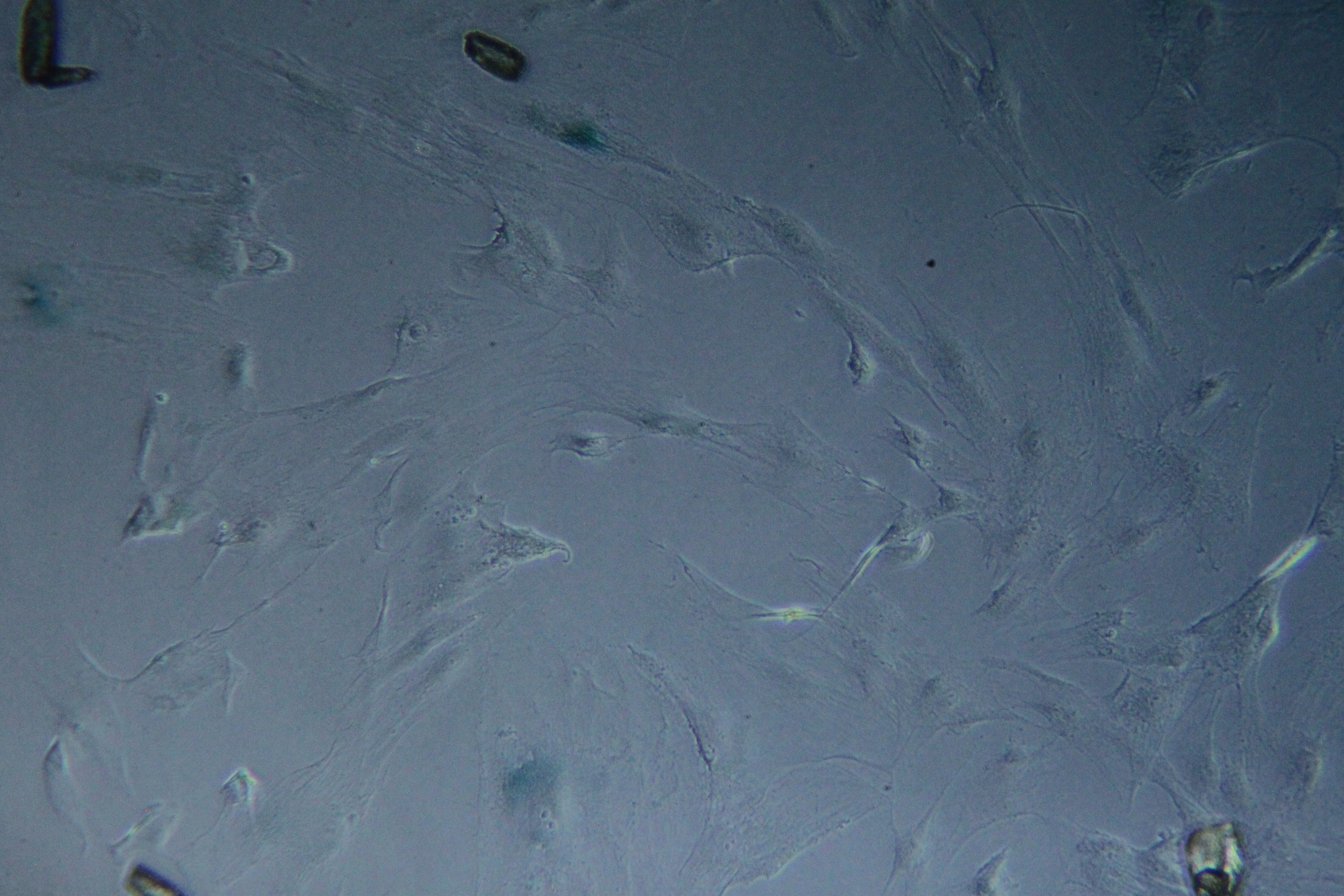

Supplement: Figure 8—figure supplement 1—source data 3. [file elife-62635-fig8-figsupp1-data3.zip › Figure 8-figure supplement 1 -Source Data 3/beta galactosidase Aged AICAR/image 3.JPG]
